# Low pH-responsive proteins revealed by a 2-DE based MS approach and related physiological responses in *Citrus* leaves

**DOI:** 10.1186/s12870-018-1413-3

**Published:** 2018-09-12

**Authors:** Jiang Zhang, Qiang Li, Yi-Ping Qi, Wei-Lin Huang, Lin-Tong Yang, Ning-Wei Lai, Xin Ye, Li-Song Chen

**Affiliations:** 1 Institute of Plant Nutritional Physiology and Molecular Biology, College of Resources and Environment, Fujian Agriculture and Forestry University (FAFU), Fuzhou, 350002 China; 2 Institute of Materia Medica, Fujian Academy of Medical Sciences, Fuzhou, 350001 China; 3 Fujian Provincial Key Laboratory of Soil Environmental Health and Regulation, College of Resources and Environment, FAFU, Fuzhou, 350002 China; 4 The Higher Educational Key Laboratory of Fujian Province for Soil Ecosystem Health and Regulation, College of Resources and Environment, FAFU, Fuzhou, 350002 China

**Keywords:** *Citrus grandis*, *Citrus sinensis*, 2-DE, Leaves, Low pH, Proteomics

## Abstract

**Background:**

Rare data are available on the molecular responses of higher plants to low pH. Seedlings of ‘Sour pummelo’ (*Citrus grandis*) and ‘Xuegan’ (*Citrus sinensis*) were treated daily with nutrient solution at a pH of 2.5, 3, or 6 (control) for nine months. Thereafter, we first used 2-dimensional electrophoresis (2-DE) to investigate low pH-responsive proteins in *Citrus* leaves. Meanwhile, we examined low pH-effects on leaf gas exchange, carbohydrates, ascorbate, dehydroascorbate and malondialdehyde. The objectives were to understand the adaptive mechanisms of *Citrus* to low pH and to identify the possible candidate proteins for low pH-tolerance.

**Results:**

Our results demonstrated that *Citrus* were tolerant to low pH, with a slightly higher low pH-tolerance in the *C. sinensis* than in the *C. grandis*. Using 2-DE, we identified more pH 2.5-responsive proteins than pH 3-responsive proteins in leaves. This paper discussed mainly on the pH 2.5-responsive proteins. pH 2.5 decreased the abundances of proteins involved in ribulose bisphosphate carboxylase/oxygenase activation, Calvin cycle, carbon fixation, chlorophyll biosynthesis and electron transport, hence lowering chlorophyll level, electron transport rate and photosynthesis. The higher oxidative damage in the pH 2.5-treated *C. grandis* leaves might be due to a combination of factors including higher production of reactive oxygen species, more proteins decreased in abundance involved in antioxidation and detoxification, and lower ascorbate level. Protein and amino acid metabolisms were less affected in the *C. sinensis* leaves than those in the *C. grandis* leaves when exposed to pH 2.5. The abundances of proteins related to jasmonic acid biosynthesis and signal transduction were increased and decreased in the pH 2.5-treated *C. sinensis* and *C. grandis* leaves, respectively.

**Conclusions:**

This is the first report on low pH-responsive proteins in higher plants. Thus, our results provide some novel information on low pH-toxicity and -tolerance in higher plants.

**Electronic supplementary material:**

The online version of this article (10.1186/s12870-018-1413-3) contains supplementary material, which is available to authorized users.

## Background

Soil acidity is a major factor limiting crop and productivity in many parts of the world, with up to 30% of the world’s ice-free land and 12% of crops affected by soil acidity [1]. What’s worse, soil pH is rapidly decreasing due to acid rain, soil leaching, intensive agriculture and monoculture, poor nutrient cycling, and the acidifying effects of nitrogen (N) fertilizer [2–5].

Usually, multiple stress factors including toxicities of H^+^, aluminum (Al) and manganese (Mn), lack of nutrients, namely N, phosphorus (P), potassium (K), magnesium (Mg), calcium (Ca) and molybdenum (Mo), decreased uptake of water, and toxic level of phenolic acids are considered to be responsible for poor growth and yield loss of crops on acidic soils [1, 6–8]. Recently, many researchers have paid attention to Al-toxicity and -tolerance, but few data are available on low pH (H^+^) damage and adaptation in plants [9–11]. Evidence shows that the adaptation of plants to H^+^ and Al are regulated by separate mechanisms [7, 8, 12]. Obviously, additional research on low pH adaptation is needed in order to a better understanding of plant adaptation to acid soils [13].

In addition to inhibiting directly or indirectly plant growth and development, low pH (high H^+^) has negative influences on cellular structure and functions, and physiological and biochemical processes, including the uptake of water and nutrients [8, 14], leaf gas exchange [8, 10, 15], chlorophyll (Chl) biosynthesis, Chl a fluorescence [8, 11, 15, 16], reactive oxygen species (ROS) production and detoxification [4, 16–18], membrane integrity [19], and cell wall structure and functions [20, 21]. Because low pH can inhibit photosynthesis and growth in some higher plants [8, 10, 15], carbohydrates should be altered by low pH. To our best knowledge, such data are very rare.

Although some workers have investigated the physiological and biochemical responses of higher plants to low pH [8, 22, 23], rare data are available on the molecular responses until recently [24]. In a study, Lager et al. investigated the effects of pH on gene expression in roots of *Arabidopsis thaliana* shifted from a nutrition solution of pH 6 to one of pH 4.5 for 1 h and 8 h, and obtained a total of 277 ‘early-responsive genes’, namely ‘1 h responsive genes’ and a total of 748 ‘late-responsive genes’, namely ‘8 h responsive genes’. The major alterations of gene expression in response to low pH were associated with Ca^2+^ signaling and cell wall modifications [24]. Howbeit these transcriptome data are very useful, great difference exists between protein level and mRNA level because the abundance of a protein is determined not only by the transcriptional rate of the gene, but also by the transcript stability, nuclear export and location, translational regulation and protein degradation [25, 26]. Because proteins are the ultimate controllers for biological processes, it is imperative to conduct a proteomic analysis in order to fully understand the molecular responses of higher plants to low pH. To our knowledge, data on low pH-responsive proteins in higher plants are very scanty.

*Citrus* can be cultivated in soils covering a wide range of pH and are tolerant to acidic soils [27]. Recently, we used sand culture to investigate the effects of pH 2.5, 3, 4, 5 and 6 on growth, nutrients, relative water content (RWC), specific leaf weight, total soluble proteins, H_2_O_2_ production, electrolyte leakage, photosynthesis and related physiological parameters in *C. grandis* and *C. sinensis* seedlings. pH 2.5 greatly inhibited seedling growth; pH 3 slightly inhibited growth; and pH 4 had almost no influence on growth. In addition, most of these parameters [i.e., leaf CO_2_ assimilation, Chl levels, ribulose bisphosphate carboxylase/oxygenase (Rubisco) activity, overwhelming majority of Chl a fluorescence parameters and specific leaf weight; root and leaf RWC and electrolyte leakage; and root, stem and leaf N and K concentrations] were altered only at pH 2.5, with slightly greater changes in the *C. grandis* seedlings than those in the *C. sinensis* seedlings. Evidently, *C. grandis* and *C. sinensis* were tolerant to low pH, and the latter was slightly more tolerant to low pH [8]. Most of soils used for *Citrus* production in China are acidic and strong acidic. Moreover, *Citrus* orchard soil pH is rapidly decreasing in the last decade [28].

In this study, we first used a 2-dimensional electrophoresis (2-DE)-based mass spectrometry (MS) approach to investigate low pH-responsive proteins in *C. sinensis* and *C. grandis* leaves. Also, we examined low pH-effects on leaf gas exchange, carbohydrates, ascorbate (ASC), dehydroascorbate (DHA) and malondialdehyde (MDA). The objectives were (*a*) to understand the adaptive mechanisms of *Citrus* to low pH and (*b*) to identify the possible candidate proteins for tolerance to low pH in *Citrus*.

## Methods

### *Citrus* seedling culture and pH treatments

Seedling culture and pH treatments were carried out according to Long et al. [8]. Briefly, four week-old uniform seedlings of ‘Xuegan’ (*C. sinensis*) and ‘Sour pummelo’ (*C. grandis*) with single stem were chosen and transplanted to 6 L pots (two seedlings per pot) filled with ~ 0.6 cm in diameter clean river sand washed thoroughly with tap water, then grown in a glasshouse under natural photoperiod at Fujian Agriculture and Forestry University (FAFU), Fuzhou (26°5’ N, 119°14′), China, until the end of the experiment. Seven weeks after transplanting, each pot was supplied daily with nutrient solution containing 2.5 mM KNO_3_, 2.5 mM Ca(NO_3_)_2_, 1 mM MgSO_4_, 0.5 mM KH_2_PO_4_, 20 μM Fe-EDTA, 10 μM H_3_BO_3_, 2 μM ZnSO_4_, 2 μM MnCl_2_, 0.5 μM CuSO_4_ and 0.065 μM (NH_4_)_6_Mo_7_O_24_ until dripping (~ 500 mL) at a pH of 6 (control), 3 or 2.5 (adjusted by 1 M H_2_SO_4_) for nine months, which were selected based on our preliminary experiment and previous study [8] and were suitable for physiological and proteomic analysis. In this study, we focused mainly on the long-term changes that allow homeostatic adjustment to low pH and on the long-term consequences of low pH because there is an opportunity to extend *Citrus* cultivation to acidic soils. No any precipitates were formed in the nutrient solution. In addition, we measured the concentrations of macroelements (N, P, K, Ca, Mg and S) in the nutrient solution. Analytic results showed that pH did not affect their solubility. Thereafter, recent fully expanded (~ 7-week-old) leaves were used for all measurements. After leaf gas exchange being determined, leaves (midribs, petioles and winged leaves removed) and leaf discs (0.6 cm in diameter) from the same seedlings were harvested at sunny noon and frozen in liquid N_2_, then stored at − 80 °C until they were used for the extract of proteins.

### Leaf nonstructural carbohydrate, ASC, DHA and malondialdehyde

Leaf fructose, glucose, sucrose and starch were assayed using enzymatic methods as previously described by Han et al. [29]. Leaf ASC and DHA were measured using enzymatic methods after being extracted with 6% (*v*/v) of HClO_4_ [30]. Leaf malondialdehyde (MDA) was measured as thiobarbituric acid-reactive substances after being extracted with 80% (v/v) of ethanol [31].

### Leaf gas exchange

Leaf gas exchange was determined with a CIARS-2 portable photosynthesis system (PP systems, Herts, UK) at a controlled CO_2_ concentration of ~ 380 μmol mol^− 1^, a controlled light intensity of ~ 1000 μmol m^− 2^ s^− 1^, a relative humidity of 64.0 ± 0.6% and a leaf temperature of 30.8 ± 0.2 °C, between 9 and 11 a.m. on a sunny day.

### Leaf protein extraction, 2-DE and image analysis

In order to reduce errors and get reliable and reproducible results, ~ 1 g frozen leaves from four seedlings (one seedling per pot, equal amount of sample per seedling) were mixed as one biological replicate. There were three replicates per treatment (a total of 12 seedlings from 12 pots). Proteins were independently extracted thrice from pH 2.5-, 3- and 6-treated samples using a phenol extraction procedure as described previously [32] and their concentrations were determined according to Bradford [33]. Both 2-DE and image analysis were performed as described by Sang et al. [34, 35] and Yang et al. [36]. Background subtraction, Gaussian fitting, gel alignment, spot detection, matching and normalization were made with PDQuest version 8.0.1 (Bio-Rad, Hercules, CA, USA). A protein spot was considered differentially abundant when it had both a *P*-value < 0.05 by ANOVA and a fold change > 1.5. All these differentially abundant protein (DAP) spots were visually checked and excised for identification by MALDI-TOF/TOF-MS.

### Protein identification by MALDI-TOF/TOF-MS and bioinformatic analysis

MALDI-TOF/TOF-MS-based protein identification was conducted on an AB SCIEX 5800 TOF/TOF plus MS (AB SCIEX, Shanghai, China) as described previously [26, 34]. All acquired spectra of samples were processed using TOF/TOF Explorer™ Software (AB SCIEX, Shanghai, China) in a default mode. The data were searched by GPS Explorer (Version 3.6) with the search engine MASCOT (Version 2.3, Matrix Science Inc., Boston, MA) against the *C. sinensis* databases (http://citrus.hzau.edu.cn/orange/index.php). The search parameters were as follows: trypsin cleavage with one missed, MS tolerance of 100 ppm, and MS/MS tolerance of 0.6 Da. At least two peptides were required to match for each protein. Protein identifications were accepted if MASCOT score was not less than 70, and the number of matched peptides (NMP) was not less than five or the sequence coverage was not less than 20% [35, 37]. Functional categories of DAPs were assigned according to Kyoto Encyclopedia of Genes and Genomes (KEGG; http://www.kegg.jp/), Uniprot (http://www.uniprot.org/) and gene ontology (GO; http://www.geneontology.org/) databases [38, 39].

### qRT-PCR analysis

About 300 mg frozen leaves from four seedlings (one seedling per pot, equal amount of sample per seedling) were pooled as one biological replicate. qRT-PCR was made with three biological and two technical replicates for each treatment (a total of 12 seedlings from 12 pots) as described by Zhou et al. [40]. Here, we randomly selected a total of 26 DAPs from the pH 2.5-treated *C. sinensis* (i.e., S1, S4, S5, S6, S7, S9, S10, S15, S23, S26, S27, S35 and S40) and *C. grandis* (i.e., G2, G5, G6, G13, G14, G15, G16, G19, G21, G36, G37, G40, and G41) leaves for qRT-PCR. Specific primers were designed from the corresponding sequences of these selected DAPs in *Citrus* genome (http://citrus.hzau.edu.cn/orange/index.php) using Primer Primier Version 5.0 (PREMIER Biosoft International, CA, USA). The sequences of the F and R primers used were listed in Additional file 1: Table S1. For the normalization of gene expression and reliability of quantitative analysis, two *Citrus* genes: *actin* (Ciclev10025866m) and *U4/U6 small nuclear ribonucleoprotein PRP31* (*PRPF31*; Ciclev10031363m), were selected as internal standards and the leaves from the pH 6-treated seedlings were used as reference sample, which was set to 1.

### Data analysis

There were 20 pots (40 seedlings) per treatment in a completely randomized design. Experiments were performed with 3–8 replicates. Results represented the mean ± SE. Significant differences among the six treatment combinations were analyzed by two (species) × three (pH levels) ANOVA, and the six means were separated by the Turkey test at *P* < 0.05.

## Results

### Physiological and biochemical responses to low pH

**Fig. 1:**
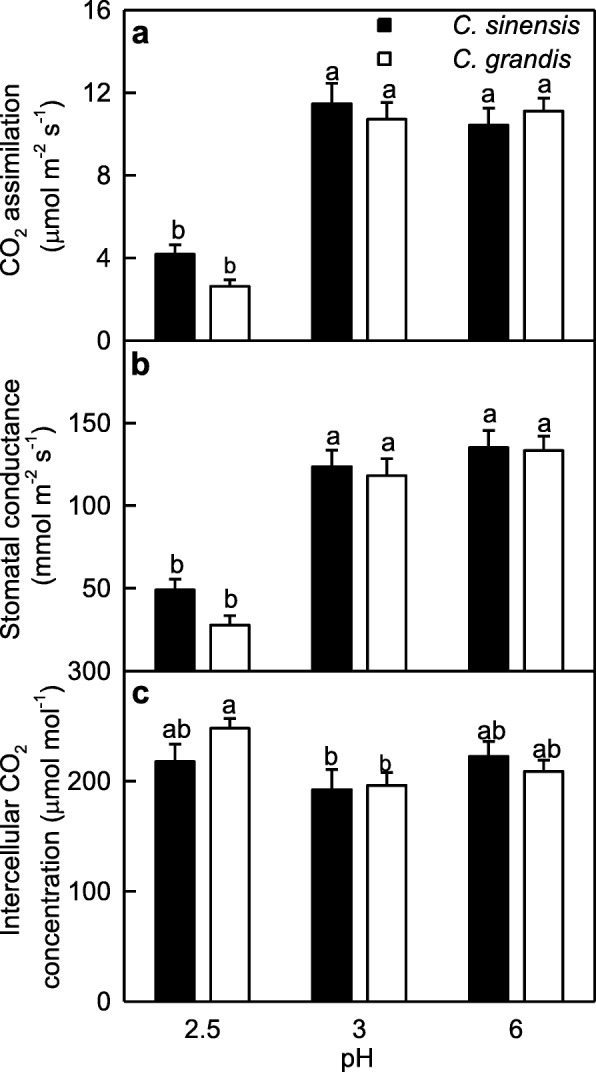
Leaf gas exchange in response to low pH***.*** **a** CO_2_ assimilation; **b** stomatal conductance; **c** intercellular CO_2_ concentration. Bars represent means ± SE (*n* = 5). Different letters above the bars indicate a significant difference at *P* < 0.05

Leaf CO_2_ assimilation and stomatal conductance were significantly lower at pH 2.5 than at pH 3 or pH 6. Intercellular CO_2_ concentration was similar among the six treatment combinations except that it was slightly higher in the 2.5-treated *C. grandis* leaves than that in the 3-treated *C. grandis* and *C. sinensis* leaves (Fig. 1). Based on the stomatal limited theory in photosynthesis [41], the pH 2.5-induced inhibition of photosynthesis was not explained alone by stomatal limitation. CO_2_ assimilation, stomatal conductance and intercellular CO_2_ concentration were similar between *C. grandis* and *C. sinensis* leaves (Fig. 1).

**Fig. 2:**
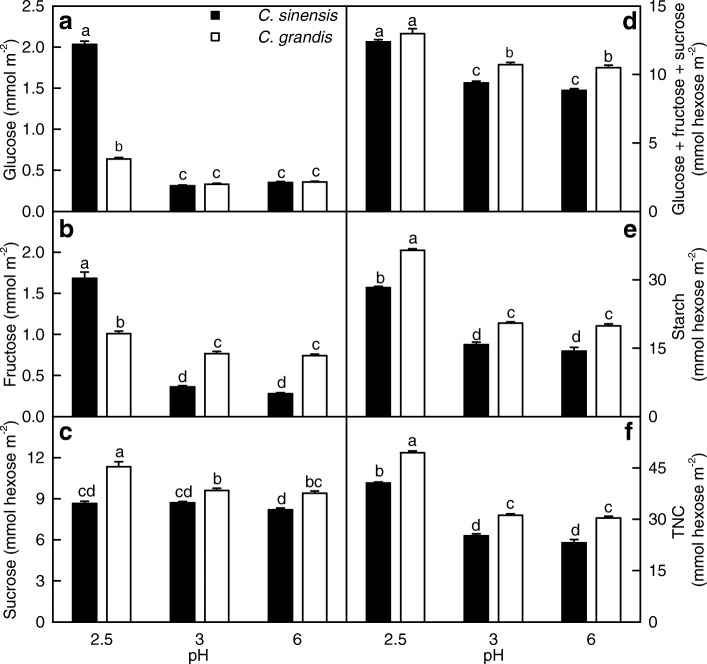
Leaf concentrations of nonstructural carbohydrates in response to low pH. **a** glucose; **b** fructose; **c** sucrose; **d** total soluble sugars (the summation of glucose, fructose and sucrose); **e** starch; **f** total nonstructural carbohydrates (TNC; the summation of glucose, fructose, sucrose and starch). Bars represent means ± SE (*n* = 8). Different letters above the bars indicate a significant difference at *P* < 0.05

As shown in Fig. 2, the levels of glucose, fructose, sucrose, total soluble sugars (the summation of glucose, fructose and sucrose), starch, and total nonstructural carbohydrates (TNC, the summation of glucose, fructose, sucrose and starch) in the C. *grandis* and *C. sinensis* leaves were elevated at pH 2.5, but unaffected at pH 3. The only exception was that sucrose levels in the *C. sinensis* leaves did not change as pH decreased from 6 to 2.5. The concentrations of all these nonstructural carbohydrates were higher in the *C. grandis* leaves than those in the *C. sinensis* leaves or similar between the two at each given pH with the exceptions that glucose and fructose concentrations were higher in the *C. sinensis* leaves than those in the *C. grandis* leaves at pH 2.5.

**Fig. 3:**
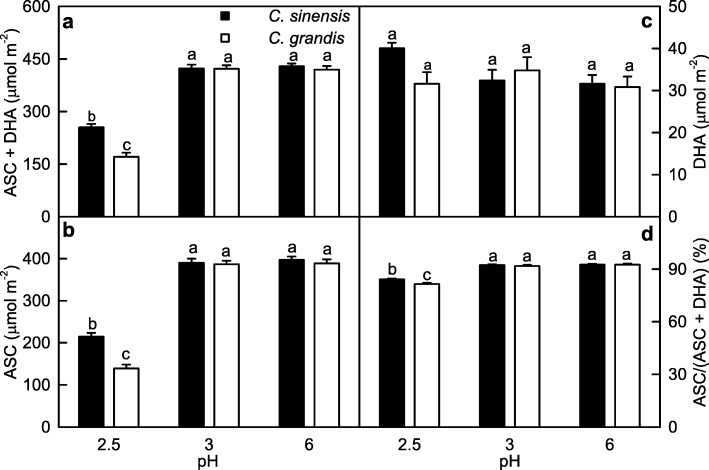
Leaf ASC + DHA, ASC and DHA concentrations, and ASC/(ASC + DHA) ratio in response to low pH*.* Bars represent means ± SE (*n* = 6–8). Different letters above the bars indicate a significant difference at *P* < 0.05

As shown in Fig. 3, only pH 2.5 decreased the levels of ASC + DHA and ASC, and the ratio of ASC/(ASC + DHA) in *C. grandis* and *C. sinensis* leaves, especially in the *C. grandis* leaves. The levels of ASC + DHA and ASC, and the ratio of ASC/(ASC + DHA) were higher in the *C. sinensis* leaves than those in the *C. grandis* leaves at pH 2.5, but similar between the two at pH 3 or pH 6. Interestingly, leaf DHA level did not significantly differ among the six treatment combinations.

**Fig. 4:**
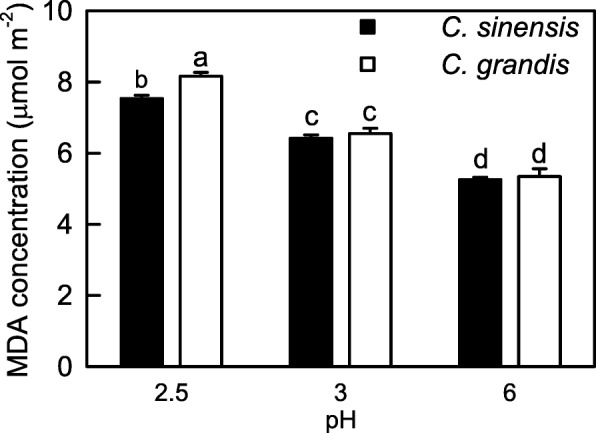
Leaf MDA concentration in response to low pH*.* Bars represent means ± SE (*n* = 7–8). Different letters above the bars indicate a significant difference at *P* < 0.05

MDA concentrations in *C. grandis* and *C. sinensis* leaves increased as pH decreased from 6 to 2.5. MDA concentrations were higher in the *C. grandis* leaves than that in the *C. sinensis* leaves at pH 2.5, but similar between the two at pH 6 or pH 3 (Fig. 4).

### Protein yield and low pH-responsive proteins in leaves

**Fig. 5:**
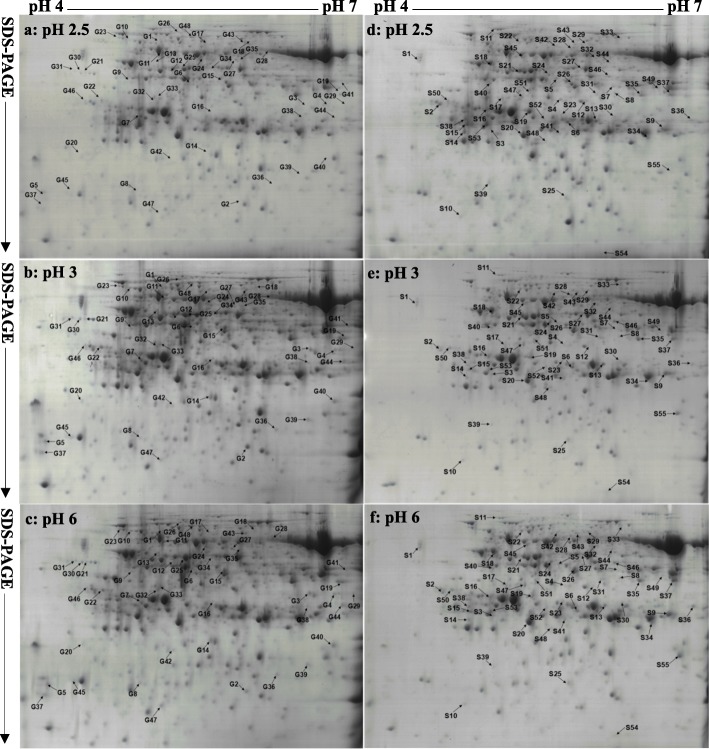
Representative 2-DE images of proteins extracted from *C. grandis* (**a**-**c**) and *C. sinensis* (**d**-**f**) leaves

To obtain reliable results, three biological replicates were conducted in this experiment (Fig. 5 and Additional file 2: Figure S1). As shown in Table 1, protein yields and the number of protein spots per gel did not significantly differ among the six treatment combinations (Table 1, Fig. 5 and Additional file 2: Figure S1).

**Table 1:** Protein yield, number of variable spots and number of identified DAP spots in *C. sinensis* and *C. grandis* leaves

	*C. sinensis*					*C. grandis*				
	pH 2.5	pH 3	pH 6			pH 2.5	pH 3	pH 6		
Protein yield (mg g^−1^ FW)	16.4 ± 0.24a	15.57 ± 0.09a	15.97 ± 0.35a			14.35 ± 0.19a	14.04 ± 0.66a	14.63 ± 0.10a		
Number of spots per gel	714 ± 12a	717 ± 4a	733 ± 14a			727 ± 13a	737 ± 9a	755 ± 11a		
	Only present at pH 2.5	Only present at pH 3		Present at both pH 2.5 and 3	Total	Only present at pH 2.5	Only present at pH 3		Present at both pH 2.5 and 3	Total
Number of DAP spots										
Increased in abundance	15	0		1	16	5	0		3	8
Decreased in abundance	29	1		6	36	14	1		23	38
Increased in abundance at pH 2.5 and decreased in abundance at pH 3				2	2				1	1
Decreased in abundance at pH 2.5 and increased in abundance at pH 3				1	1				1	1
Total	44	1		10	55	19	1		28	48
Number of identified DAP spots										
Increased in abundance	13	0		1	14	5	0		3	8
Decreased in abundance	26	0		6	32	12	1		21	34
Increased in abundance at pH 2.5 and decreased in abundance at pH 3				2	2				1	1
Decreased in abundance at pH 2.5 and increased in abundance at pH 3				1	1				1	1
Total	39	0		10	49	17	1		26	44

Data are means ± SE (*n* = 3). Different letters within a row indicate significant differences at *P* < 0.05

**Fig. 6:**
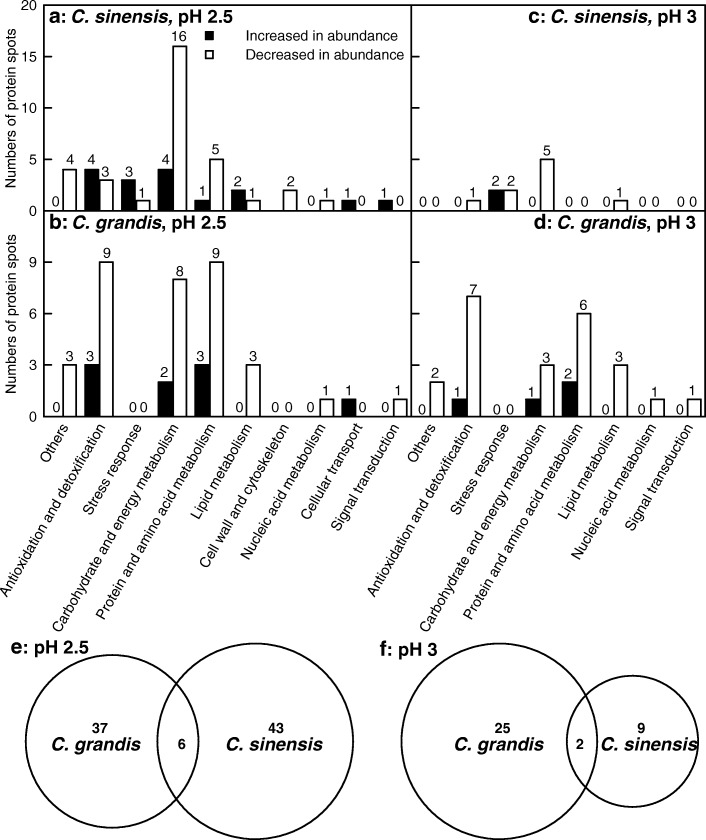
Classification of low pH-responsive proteins (**a**-**d**) and venn diagram analysis of low pH-responsive proteins (**e**-**f**). For **e,** 37 or 43 identified DAPs only presented in the pH 2.5-treated *C. grandis* or *C. sinensis* leaves, respectively, and only six identified DAPs with the same accession number were shared by the two. For **f**, 25 or 9 identified DAPs only presented in the pH 3-treated *C. grandis* or *C. sinensis* leaves, respectively, and only two identified DAPs with the same accession number were shared by the two

A protein spot having an average fold change > 1.5 and a *P*-value < 0.05 was considered as differentially abundant. Based on the two criteria, we obtained 55 and 48 DAP spots from the pH 2.5 and/or pH 3-treated *C. sinensis* and *C. grandis* leaves, respectively. After submitting these DAP spots to MALDI-TOF/TOF-MS-based identification, we identified 49 and 44 DAP spots in the pH 2.5 and/or pH 3-treated *C. sinensis* and *C. grandis* leaves, responsively (Tables 1, 2 and 3, Fig. 5 and Additional file 3: Table S2, Additional file 4: Table S3, Additional file 5: Figure S2). For *C. sinensis*, 39 identified DAP spots only presented in pH 2.5-treated leaves, and 10 identified DAP spots with the same accession number were shared by the two. For *C. grandis*, 26 identified DAP spots were shared by pH 2.5- and pH 3-treated leaves. Only 17 or one identified DAP spots presented in pH 2.5- or pH 3-treated leaves, respectively. In short, we identified 16 protein spots increased in abundance and 33 protein spots decreased in abundance, and two protein spots increased in abundance and eight protein spots decreased in abundance from the pH 2.5- and pH 3-treated *C. sinensis* leaves, respectively, and nine protein spots increased in abundance and 34 protein spots decreased in abundance, and four protein spots increased in abundance and 23 protein spots decreased in abundance from the pH 2.5- and pH 3-treated *C. grandis* leaves, respectively (Tables 1, 2 and 3 and Fig. 6a-d). The majority of these low pH-responsive proteins were identified only in the *C. sinensis* or *C. grandis* leaves, only six pH 2.5-responsive proteins (i.e., Cs7g31800, Cs3g01420, Cs1g25510, Cs8g19010, Cs3g11320 and orange1.1 t04488) and two pH 3-responsive proteins (i.e., Cs7g31800 and Cs1g25510) with the same accession number were simultaneously identified in the two species (Tables 2 and 3 and Fig. 6e-f). These low pH-responsive proteins were mainly associated with carbohydrate and energy metabolism, antioxidation and detoxification, stress response, protein and amino acid metabolisms, lipid metabolism, cellular transport, signal transduction and nucleic acid metabolism (Tables 2 and 3 and Fig. 6a-d).

**Table 2:** DAP spots and their identification by MALDI-TOF/TOF-MS in leaves from *C. sinensis* seedlings submitted to pH 2.5, pH 3 or pH 6 for 9 months

Spot No.^a^	Protein identity	Accession No.	Mr (kDa)/pI theor.	Mr (kDa)/pI exp.	Protein score	Peptide ions	NMP^b^	Ratio^c^			Covered^d^ sequence (%)	Charge
								pH 2.5	pH 3	pH 6		
Antioxidation and detoxification												
S6	L-ascorbate peroxidase 1, cytosolic	Cs8g17370.1	27.57/5.55	44.86/5.80	584	1 26	24	2.16 ± 0.09a	1.07 ± 0.12b	1.00 ± 0.03b	22	1
S4	L-ascorbate peroxidase 3, peroxisomal	Cs3g19810.2	47.33/8.59	53.31/5.73	573	144	25	2.94 ± 0.80a	1.11 ± 0.21b	1.00 ± 0.15b	23	1
S5	Probable aldo-keto reductase 1	Cs3g10670.1	38.42/5.50	62.64/5.68	295	90	15	2.41 ± 0.26a	0.84 ± 0.05b	1.00 ± 0.17b	14	1
S8	Isoflavone reductase-like protein	Cs2g16220.1	34.25/6.40	56.42/6.23	155	56	17	2.03 ± 0.29a	0.80 ± 0.16b	1.00 ± 0.11b	15	1
S1	Peroxidase 15	orange1.1 t02046.1	37.43/4.52	71.41/4.46	178	90	9	0.37 ± 0.07b	0.45 ± 0.06b	1.00 ± 0.04a	8	1
S9	Glutathione S-transferase U19	Cs5g15190.1	25.56/7.56	42.77/6.64	148	71	14	0.30 ± 0.05b	0.83 ± 0.10a	1.00 ± 0.09a	13	1
S10	Thioredoxin-2	Cs7g13660.1	21.44/8.44	21.81/4.87	178	62	7	0.47 ± 0.10b	0.87 ± 0.11a	1.00 ± 0.06a	6	1
Stress response												
S11	Heat shock protein 90–1	Cs5g03150.1	80.52/5.03	90.75/5.15	186	96	41	1.62 ± 0.19a	0.53 ± 0.09c	1.00 ± 0.02b	37	1
S12	Putative uncharacterized protein Sb02g035950	Cs1g06050.1	27.76/5.56	52.74/6.00	265	87	11	1.29 ± 0.04a	0.51 ± 0.14b	1.00 ± 0.10a	10	1
S13	Abscisic stress ripening-like protein	Cs3g21500.1	20.05/5.75	45.68/6.08	411	92	14	0.20 ± 0.05c	1.59 ± 0.11a	1.00 ± 0.06b	13	1
S17	Thiamine thiazole synthase 1, chloroplastic	Cs4g11090.1	37.60/5.40	53.52/5.22	304	95	15	1.64 ± 0.12a	0.75 ± 0.02b	1.00 ± 0.18b	14	1
S39	S-norcoclaurine synthase; Pathogenesis-related (PR)-10-related norcoclaurine synthase-like protein	Cs6g03210.1	17.29/4.89	28.52/5.12	485	90	17	4.45 ± 0.27a	2.23 ± 0.16b	1.00 ± 0.13c	15	1
Carbohydrate and energy metabolism												
S14	Chlorophyll a-b binding protein 215	Cs1g06360.1	28.93/5.13	41.21/4.90	71	56	6	2.22 ± 0.10a	0.46 ± 0.03c	1.00 ± 0.16b	5	1
S20	Chlorophyll a-b binding protein 4, chloroplastic	Cs3g06180.1	29.52/6.84	40.18/5.42	167	88	11	0.13 ± 0.02c	0.71 ± 0.07b	1.00 ± 0.11a	10	1
S16	Oxygen-evolving enhancer protein 1–1, chloroplastic	Cs1g23450.1	35.38/5.83	49.12/5.09	371	110	14	2.20 ± 0.59a	0.82 ± 0.11b	1.00 ± 0.02b	13	1
S19	Oxygen-evolving enhancer protein 1–1, chloroplastic	Cs1g23450.1	35.38/5.83	48.88/5.44	523	135	19	2.67 ± 0.76a	0.99 ± 0.19b	1.00 ± 0.03b	17	1
**S18**^**e**^	**Ribulose bisphosphate carboxylase/oxygenase activase 1, chloroplastic**	**Cs7g31800.4**	**50.90/5.33**	**68.00/5.12**	**534**	**91**	**23**	**0.30 ± 0.06b**	**0.44 ± 0.06b**	**1.00 ± 0.09a**	**21**	**1**
**S21**	**Ribulose bisphosphate carboxylase/oxygenase activase 1, chloroplastic**	**Cs7g31800.3**	**46.96/5.94**	**65.74/5.36**	**511**	**109**	**20**	**0.21 ± 0.02b**	**0.91 ± 0.02a**	**1.00 ± 0.08a**	**18**	**1**
S22	Rubisco subunit binding-protein β-2 subunit; Chaperonin 60 subunit β 1	Cs9g03300.1	64.78/5.85	81.19/5.36	798	107	39	0.33 ± 0.04b	1.10 ± 0.10a	1.00 ± 0.13a	35	1
S35	**Ferredoxin-NADP reductase, leaf isozyme, chloroplastic**	**Cs1g25510.1**	**40.48/8.68**	**55.49/6.40**	**258**	**80**	**22**	**0.45 ± 0.04c**	**0.65 ± 0.08b**	**1.00 ± 0.02a**	**20**	**1**
S37	**Ferredoxin-NADP reductase, leaf isozyme, chloroplastic**	**Cs1g25510.4**	**40.48/8.68**	**55.29/6.70**	**240**	**82**	**21**	**0.45 ± 0.03b**	**0.91 ± 0.11a**	**1.00 ± 0.13a**	**19**	**1**
S27	Phosphoglycerate kinase 1, chloroplastic	orange1.1 t03280.1	49.45/8.20	67.19/5.91	567	95	25	0.25 ± 0.10b	0.87 ± 0.06a	1.00 ± 0.11a	23	1
S29	Phosphoglycerate kinase 1, chloroplastic	orange1.1 t03280.1	49.45/8.20	80.46/5.95	346	92	13	0.06 ± 0.02b	0.95 ± 0.10a	1.00 ± 0.14a	12	1
**S23**	**2-C-methyl-D-erythritol 4-phosphate cytidylyltransferase, chloroplastic**	**Cs3g01420.1**	**32.59/8.29**	**47.43/5.72**	**415**	**121**	**14**	**0.31 ± 0.04b**	**0.80 ± 0.08a**	**1.00 ± 0.15a**	**13**	**1**
S24	Succinyl-CoA ligase [ADP-forming] subunit beta, mitochondrial	Cs5g29390.1	45.26/5.98	63.82/5.62	286	78	27	0.52 ± 0.13b	1.10 ± 0.06a	1.00 ± 0.06a	25	1
S28	Dihydrolipoyllysine-residue acetyltransferase component of pyruvate dehydrogenase complex, mitochondrial	Cs1g17930.1	59.37/8.43	77.39/5.80	390	94	23	0.30 ± 0.06c	0.77 ± 0.04b	1.00 ± 0.05a	21	1
S32	Dihydrolipoyllysine-residue succinyltransferase component of 2-oxoglutarate dehydrogenase complex 1, mitochondrial	Cs2g21190.1	51.06/9.07	72.17/6.03	181	135	11	0.19 ± 0.04b	0.86 ± 0.04a	1.00 ± 0.07a	10	1
S3	NADH dehydrogenase [ubiquinone] iron-sulfur protein 8-A, mitochondrial	Cs8g06410.1	26.11/5.68	42.59/5.12	238	48	20	2.58 ± 0.27a	1.10 ± 0.10b	1.00 ± 0.09b	18	1
S26	ATP synthase gamma chain 1, chloroplastic	Cs2g03080.1	40.62/6.08	59.62/5.82	208	103	10	0.19 ± 0.04c	0.44 ± 0.06b	1.00 ± 0.06a	9	1
S30	Probable ATP synthase 24 kDa subunit, mitochondrial	Cs1g04030.1	27.52/8.90	46.81/6.24	368	96	22	0.22 ± 0.06b	0.55 ± 0.11b	1.00 ± 0.14a	20	1
S36	Probable ATP synthase 24 kDa subunit, mitochondrial	Cs1g04030.1	27.52/8.90	45.64/6.86	543	102	27	0.29 ± 0.07b	0.68 ± 0.08ab	1.00 ± 0.24a	25	1
**S47**	**DNA-damage-repair/toleration protein DRT102; ribose-5-phosphate isomerase B**	**Cs3g11320.1**	**33.52/5.25**	**53.47/5.39**	**393**	**85**	**19**	**0.44 ± 0.07b**	**1.02 ± 0.10a**	**1.00 ± 0.16a**	**17**	**1**
Protein and amino acid metabolism												
S40	Peptidyl-prolyl cis-trans isomerase CYP37, chloroplastic	Cs1g06710.1	50.39/6.42	60.41/5.11	579	139	21	3.06 ± 0.91a	1.36 ± 0.22ab	1.00 ± 0.11b	19	1
S43	T-complex protein 1 subunit beta	Cs3g26890.2	57.19/5.56	79.99/5.91	226	73	22	0.30 ± 0.13b	1.04 ± 0.05a	1.00 ± 0.19a	20	1
S41	Proteasome subunit beta type-6-A like protein	Cs7g07630.1	25.57/5.21	40.62/5.73	254	86	12	0.23 ± 0.01c	0.68 ± 0.04b	1.00 ± 0.10a	11	1
S44	26S proteasome non-ATPase regulatory subunit 11A	Cs4g04180.1	47.05/5.79	70.34/6.16	367	97	24	0.22 ± 0.028b	0.83 ± 0.091a	1.00 ± 0.06a	22	1
S50	**Unknown protein DS12 from 2D-PAGE of leaf, chloroplastic; ACT domain-containing protein, putative, expressed**	**orange1.1 t04488.1**	**30.69/5.59**	**51.57/4.75**	**333**	**105**	**15**	**0.35 ± 0.07b**	**0.89 ± 0.02a**	**1.00 ± 0.04a**	**14**	**1**
S51	Kynurenine formamidase	Cs8g05140.1	30.62/5.32	55.58/5.48	270	75	13	0.44 ± 0.01b	1.04 ± 0.03a	1.00 ± 0.08a	12	1
Cell wall and cytoskeleton												
S45	Tubulin alpha-1 chain	Cs9g03120.1	49.75/4.99	73.13/5.41	487	85	20	0.40 ± 0.05b	0.93 ± 0.12a	1.00 ± 0.13a	18	1
S46	Caffeic acid 3-O-methyltransferase 1	orange1.1 t05216.1	29.98/5.73	63.37/6.19	194	66	11	0.53 ± 0.09b	1.18 ± 0.04a	1.00 ± 0.04a	10	1
Nucleic acid metabolism												
S48	Hypoxanthine-guanine phosphoribosyltransferase	Cs3g21990.1	20.48/5.54	38.60/5.61	143	94	8	0.41 ± 0.03b	1.07 ± 0.17a	1.00 ± 0.08a	7	1
Lipid metabolism												
S2	Plastid lipid-associated protein 2, chloroplastic; Chromoplast-specific carotenoid-associated protein, chromoplast	Cs2g02520.1	43.18/6.08	50.48/4.67	503	92	24	1.68 ± 0.09a	0.79 ± 0.18b	1.00 ± 0.02b	22	1
S7	Epoxide hydrolase 4	Cs2g06360.1	36.94/5.90	59.20/6.19	352	73	21	2.51 ± 0.27a	0.90 ± 0.10b	1.00 ± 0.18b	19	1
S31	Cinnamoyl-CoA reductase 1	Cs8g20610.1	35.48/5.57	55.34/6.06	220	77	14	0.31 ± 0.02b	0.66 ± 0.21ab	1.00 ± 0.12a	13	1
Cellular transport												
S15	Ferritin-1, chloroplastic	Cs6g09150.2	28.97/5.46	43.81/4.92	88	64	8	1.97 ± 0.34a	0.69 ± 0.09b	1.00 ± 0.11b	7	1
Signal transduction												
S38	14–3-3-like protein GF14 kappa; General regulatory factor 8	Cs1g20220.1	27.69/4.87	45.64/4.90	339	106	20	4.71 ± 0.32a	1.20 ± 0.15b	1.00 ± 0.06b	18	1
Others												
S52	Putative uncharacterized protein Sb02g003450	Cs5g09380.2	27.69/7.84	45.27/5.58	193	60	14	0.38 ± 0.11b	1.00 ± 0.09a	1.00 ± 0.12a	13	1
**S42**	**Putative uncharacterized protein Sb09g010000**	**Cs8g19010.1**	**55.68/7.66**	**79.38/5.66**	**248**	**102**	**19**	**0.19 ± 0.06b**	**0.73 ± 0.13a**	**1.00 ± 0.22a**	**17**	**1**
S34	Flavoprotein WrbA	Cs4g11860.1	22.29/5.75	40.45/6.51	256	136	10	0.44 ± 0.05b	0.67 ± 0.07ab	1.00 ± 0.14a	9	1
S49	NAD(P)H-dependent 6′-deoxychalcone synthase	orange1.1 t00001.2	67.34/9.06	57.67/6.61	247	100	20	0.46 ± 0.07b	0.67 ± 0.11b	1.00 ± 0.07a	18	1
Unidentified protein spots												
S33	Probable phosphoglucomutase, cytoplasmic 1	orange1.1 t05474.1	16.20/5.32	84.77/6.25	100	91	4	0.40 ± 0.05b	0.77 ± 0.08ab	1.00 ± 0.17a	4	1
S25	Glucose-6-phosphate 1-dehydrogenase 1, chloroplastic	Cs7g11110.1	66.95/7.68	25.37/5.76	65	23	21	0.46 ± 0.04b	1.34 ± 0.08a	1.00 ± 0.23b	19	1
S53	Endo-1,3;1,4-beta-D-glucanase	Cs9g05910.3	28.00/7.07	43.84/5.09	43	/	12	3.32 ± 0.29a	1.02 ± 0.07b	1.00 ± 0.09b	11	1
S54	E3 ubiquitin-protein ligase MARCH9	Cs9g05100.7	32.53/8.56	16.93/6.1	44	/	12	4.26 ± 0.19a	0.45 ± 0.02b	1.00 ± 0.18b	11	1
S55	Cytochrome b6-f complex iron-sulfur subunit, chloroplastic	Cs2g22650.3	22.32/8.76	30.95/8.76	52	36	6	0.33 ± 0.04c	0.75 ± 0.05b	1.00 ± 0.09a	5	1

^a^Spot number corresponds to the 2-DE imagines in Fig. 5

^b^NMP means the number of matched peptides

^c^Ratio means the ratio of pH 2.5 and pH 3.0 to pH 6.0; Different letters within a row indicate significant differences at *P* < 0.05

^d^Covered sequence (%) means the ratio of the number of amino of the matched peptides to the number of amino acids of the full-length protein

^e^Low pH-responsive proteins shared by the two *Citrus* species were highlighted in bold

**Table 3:** DAP spots and their identification by MALDI-TOF/TOF-MS in leaves from *C. grandis* seedlings submitted to pH 2.5, pH 3 or pH 6 for 9 months

Spot No.^a^	Protein identity	Accession No.	Mr (kDa)/pI theor.	Mr (kDa)/pI exp.	Protein score	Peptide ions	NMP^b^	Ratio^c^			Covered^d^ sequence (%)	Charge
								pH 2.5	pH 3	pH 6		
Antioxidation and detoxification												
G2	Copper/zinc superoxide dismutase 2, chloroplastic	Cs8g15520.1	26.00/6.52	22.72/6.05	341	119	8	2.51 ± 0.06a	2.42 ± 0.24a	1.00 ± 0.17b	7	1
G11	Aldehyde dehydrogenase family 2 member B4, mitochondrial	Cs5g05240.1	58.91/7.11	75.4/5.31	409	115	27	2.48 ± 0.20a	0.33 ± 0.06c	1.00 ± 0.08b	25	1
G40	Apolipoprotein D	Cs4g01600.1	21.52/6.33	32.64/6.84	264	106	11	1.57 ± 0.22a	0.62 ± 0.04b	1.00 ± 0.10b	10	1
G15	Phosphomannomutase 1	orange1.1 t00331.1	28.16/6.19	59.09/5.85	561	122	25	0.48 ± 0.05b	1.01 ± 0.02a	1.00 ± 0.05a	23	1
G1	NADP-dependent alkenal double bond reductase P2	Cs5g21010.1	36.04/6.16	82.02/5.3	435	116	18	0.18 ± 0.04b	0.38 ± 0.07b	1.00 ± 0.29a	16	1
G3	Thioredoxin-like protein CDSP32, chloroplastic	Cs3g26690.1	39.02/8.59	49.64/6.62	472	106	18	0.36 ± 0.05c	0.66 ± 0.09b	1.00 ± 0.08a	16	1
G4	Thioredoxin-like protein CDSP32, chloroplastic	Cs3g26690.1	39.02/8.59	49.46/6.85	477	106	20	0.22 ± 0.01b	0.83 ± 0.15a	1.00 ± 0.22a	18	1
G8	Ferredoxin-thioredoxin reductase catalytic chain, chloroplastic	Cs6g20130.1	16.57/6.29	24.68/5.07	130	61	7	0.25 ± 0.05b	0.42 ± 0.10b	1.00 ± 0.08a	6	1
G23	Protein disulfide isomerase-like 1–1	Cs3g19790.1	55.62/4.84	83.51/4.91	625	95	31	0.43 ± 0.06b	0.80 ± 0.09ab	1.00 ± 0.16a	28	1
G39	Annexin A13	Cs3g18360.1	36.10/5.55	28.12/6.6	422	82	23	0.18 ± 0.05c	0.52 ± 0.04b	1.00 ± 0.07a	21	1
G36	Nucleoside diphosphate kinase II, chloroplastic	Cs5g06840.1	25.53/9.35	25.98/6.32	467	106	13	0.07 ± 0.02b	0.25 ± 0.02b	1.00 ± 0.15a	12	1
G5	Betaine aldehyde dehydrogenase 1, chloroplastic	Cs5g04880.1	54.95/5.40	24.08/4.24	236	116	12	0.10 ± 0.05c	0.52 ± 0.10b	1.00 ± 0.01a	11	1
Carbohydrate and energy metabolism												
G7	RuBisCO large subunit-binding protein subunit alpha, chloroplastic; Chaperonin 60 subunit alpha 1	Cs8g16040.1	61.50/5.23	46.22/5.1	215	77	23	2.75 ± 0.28a	1.58 ± 0.11b	1.00 ± 0.06b	21	1
G10	RuBisCO large subunit-binding protein subunit alpha, chloroplastic; Chaperonin 60 subunit α 1	Cs8g16040.1	61.50/5.23	82.35/5.02	783	126	37	0.35 ± 0.09b	0.99 ± 0.13a	1.00 ± 0.09a	34	1
**G12**^e^	**Ribulose bisphosphate carboxylase/oxygenase activase 1, chloroplastic**	**Cs7g31800.2**	**41.38/5.07**	**64.04/5.4**	**219**	**97**	**10**	**0.14 ± 0.02b**	**0.31 ± 0.05b**	**1.00 ± 0.08a**	**9**	**1**
**G19**	**Ferredoxin--NADP reductase, leaf isozyme, chloroplastic**	**Cs1g25510.1**	**40.48/8.68**	**54.2/6.92**	**289**	**70**	**23**	**0.44 ± 0.05b**	**0.53 ± 0.06b**	**1.00 ± 0.12a**	**21**	**1**
G13	Rhodanese-like domain-containing protein 4A, chloroplastic	orange1.1 t00475.2	46.75/5.59	69.08/5.26	507	130	24	0.41 ± 0.06b	1.00 ± 0.12a	1.00 ± 0.18a	22	1
**G16**	**2-C-methyl-D-erythritol 4-phosphate cytidylyltransferase, chloroplastic**	**Cs3g01420.1**	**32.59/8.29**	**46.72/5.75**	**321**	**95**	**12**	**0.34 ± 0.06b**	**0.88 ± 0.13a**	**1.00 ± 0.15a**	**11**	**1**
G17	Probable 6-phosphogluconolactonase 2	orange1.1 t02542.1	35.38/6.24	81.27/5.72	454	133	15	0.34 ± 0.04b	2.61 ± 0.45a	1.00 ± 0.01b	14	1
G18	Dihydroxyacetone kinase 2	orange1.1 t02644.1	61.75/5.74	81.59/6.13	342	95	19	2.30 ± 0.56a	1.66 ± 0.14ab	1.00 ± 0.04b	17	1
G9	Probable fructokinase-1	Cs5g22920.1	35.11/4.98	59.41/5.02	718	137	22	0.49 ± 0.10b	0.58 ± 0.05b	1.00 ± 0.07a	20	1
**G33**	**DNA-damage-repair/toleration protein DRT102; ribose-5-phosphate isomerase B**	**Cs3g11320.1**	**33.52/5.25**	**52.44/5.28**	**279**	**104**	**15**	**0.27 ± 0.06b**	**0.79 ± 0.05a**	**1.00 ± 0.20a**	**14**	**1**
Protein and amino acid metabolism												
G26	Peptidyl-prolyl cis-trans isomerase FKBP62	Cs7g16620.3	63.83/5.19	87.75/5.46	344	81	21	0.23 ± 0.02c	0.79 ± 0.07b	1.00 ± 0.03a	19	1
G21	Peptidyl-prolyl cis-trans isomerase CYP38, chloroplastic	Cs2g28260.1	48.02/5.00	64.55/4.61	231	80	18	0.20 ± 0.06c	0.47 ± 0.04b	1.00 ± 0.05a	16	1
G30	Ankyrin repeat domain-containing protein 2	orange1.1 t02270.1	64.18/5.29	64.12/4.57	261	110	16	0.16 ± 0.04b	0.72 ± 0.09a	1.00 ± 0.11a	15	1
G31	Ankyrin repeat domain-containing protein 2	orange1.1 t02270.1	64.18/5.29	65.14/4.51	228	120	13	0.30 ± 0.13b	1.13 ± 0.16a	1.00 ± 0.21a	12	1
G20	SKP1-like protein 10	Cs3g26510.1	17.82/4.56	33.74/4.58	105	60	11	0.16 ± 0.02b	0.27 ± 0.04b	1.00 ± 0.13a	10	1
G29	Subtilisin-like protease SDD1	Cs1g17350.1	82.90/5.86	49.48/6.95	311	107	12	0.25 ± 0.06b	0.07 ± 0.01b	1.00 ± 0.09a	11	1
G24	26S protease regulatory subunit 6B homolog	Cs1g08770.1	46.49/5.42	72.36/5.68	425	87	27	1.24 ± 0.12a	0.42 ± 0.05b	1.00 ± 0.12a	25	1
G6	Proline iminopeptidase	Cs8g03250.1	44.67/5.70	60.15/5.58	463	116	23	3.44 ± 0.42a	2.21 ± 0.41b	1.00 ± 0.05c	21	1
G25	Glutamine synthetase cytosolic isozyme	Cs9g05680.1	47.86/6.29	65.88/5.51	181	82	16	0.07 ± 0.02c	0.61 ± 0.12b	1.00 ± 0.14a	15	1
G34	Dihydropyrimidine dehydrogenase [NADP(+)]	orange1.1 t02649.1	46.01/6.29	66.1/5.75	189	96	16	0.28 ± 0.04b	0.50 ± 0.02b	1 ± 0.17a	15	1
G27	Argininosuccinate synthase, chloroplastic	Cs5g07120.1	54.38/6.50	70.63/5.94	371	112	26	2.36 ± 0.13a	0.90 ± 0.14b	1.00 ± 0.18b	24	1
G28	3-isopropylmalate dehydratase large subunit 1	Cs5g35290.1	55.66/6.68	76.63/6.29	124	72	15	3.06 ± 0.05a	1.55 ± 0.12b	1.00 ± 0.08c	14	1
**G22**	**Unknown protein DS12 from 2D-PAGE of leaf, chloroplastic; ACT domain-containing protein, putative, expressed**	**orange1.1 t04488.3**	**26.98/4.94**	**51.32/4.76**	**318**	**89**	**16**	**0.41 ± 0.11b**	**1.08 ± 0.26a**	**1.00 ± 0.02a**	**15**	**1**
Signal transduction												
G32	Plasma membrane-associated cation-binding protein 1	Cs2g12010.1	23.34/5.03	51.44/5.25	113	37	12	0.19 ± 0.04b	0.39 ± 0.04b	1.00 ± 0.09a	11	1
Nucleic acid metabolism												
G35	Adenylosuccinate synthetase 1, chloroplastic	orange1.1 t03395.1	53.23/6.40	70.64/6.01	568	101	25	0.25 ± 0.01b	0.42 ± 0.09b	1 ± 0.22a	23	1
Lipid metabolism												
G37	Lipase/lipooxygenase, PLAT/LH2 family protein	Cs1g01370.1	19.62/4.80	22.28/4.22	213	134	7	0.15 ± 0.03c	0.46 ± 0.07b	1 ± 0.03a	6	1
G38	Lipase/lipooxygenase, PLAT/LH2 family protein	Cs1g01370.1	19.62/4.80	44.79/6.6	222	137	7	0.33 ± 0.07b	0.48 ± 0.06b	1 ± 0.003a	6	1
G14	Allene oxide cyclase 1, chloroplastic	Cs6g18900.1	27.51/8.63	34.12/5.73	371	83	13	0.19 ± 0.06c	0.64 ± 0.03b	1.00 ± 0.11a	12	1
Cellular transport												
G41	V-type proton ATPase catalytic subunit A isoform 1	Cs1g10270.1	68.68/5.29	59.4/6.96	635	100	40	1.50 ± 0.16a	0.65 ± 0.07b	1.00 ± 0.08b	36	1
Others												
G42	Putative uncharacterized protein Sb01g045410	Cs4g06170.1	26.81/8.90	31.73/5.39	228	82	11	0.13 ± 0.04b	0.22 ± 0.03b	1.00 ± 0.14a	10	1
**G43**	**Putative uncharacterized protein Sb09g010000**	**Cs8g19010.1**	**55.68/7.66**	**80.33/6.05**	**285**	**118**	**20**	**0.46 ± 0.05c**	**1.18 ± 0.06a**	**1.00 ± 0.02b**	**18**	**1**
G44	Uncharacterized protein At2g37660, chloroplastic	Cs6g06590.1	36.01/9.43	45/6.92	485	136	14	0.19 ± 0.03c	0.58 ± 0.07b	1.00 ± 0.15a	13	1
Unidentified protein spots												
G45	Kinesin-like protein KIF21A	Cs3g17220.3	108.17/6.47	25.06/4.49	52	/	28	0.27 ± 0.07b	0.39 ± 0.07b	1.00 ± 0.25a	25	1
G46	Actinidain	Cs3g23180.1	51.42/5.37	52.44/4.62	55	55	2	0.35 ± 0.01b	0.97 ± 0.25a	1.00 ± 0.13a	2	1
G47	Peptide methionine sulfoxide reductase B2, chloroplastic	Cs8g03090.1	17.59/10.55	20.63/5.27	51	24	8	0.10 ± 0.009b	0.34 ± 0.14b	1.00 ± 0.13a	7	1
G48	Putative uncharacterized protein	Cs7g03150.1	15.56/6.22	85.49/5.56	55	29	7	0.35 ± 0.06b	0.85 ± 0.05a	1.00 ± 0.13a	6	1

^a^Spot number corresponds to the 2-DE imagines

^b^NMP means the number of matched peptides in Fig. 5

^c^Ratio means the ratio of pH 2.5 and pH 3 to pH 6.0; Different letters within a row indicate significant differences at *P* < 0.05

^d^Covered sequence (%) means the ratio of the number of amino of the matched peptides to the number of amino acids of the full-length protein

^e^Low pH-responsive proteins shared by the two *Citrus* species were highlighted in bold

### qRT-PCR analysis of genes for some low pH-responsive proteins

**Fig. 7:**
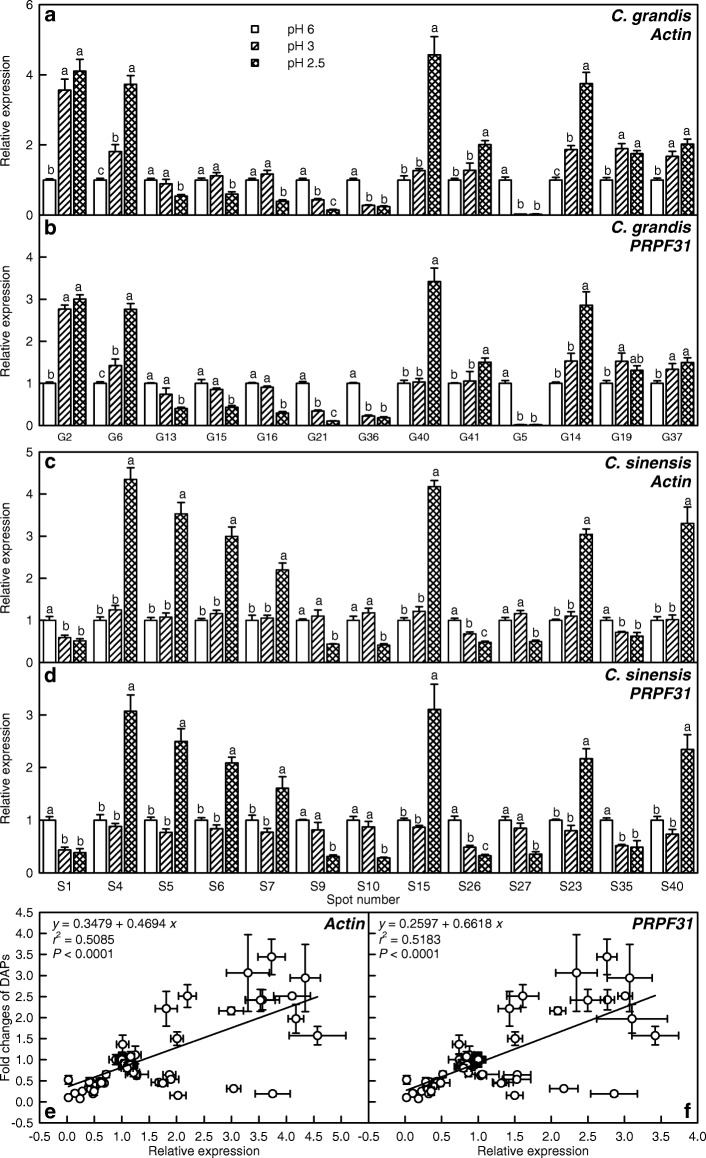
qRT-PCR analysis of 26 low-pH responsive protein genes. Relative expression levels of genes encoding 26 low-pH responsive proteins identified in *C. grandis* (**a-b**) and *C. sinensis* (**c-d**) leaves using *actin* (**a**, **c**) and *PRPF31* (**b**, **d**) as internal standards, and the correlation analysis of qRT-PCR results and 2-DE data (**e-f**). For **a-d**, bars represent means ± SE (*n* = 3). For the same genes, different letters above the bars indicate a significant difference at *P* < 0.05. For **e** and **f**, 2-DE data from Tables 2 and 3

To understand the correlation between gene expression levels and 2-DE data, we used qRT-PCR to assay the transcript levels of genes for a total of 26 DAPs from the *C. sinensis* (i.e., S1, S4, S5, S6, S7, S9, S10, S15, S23, S26, S27, S35 and S40) and *C. grandis* (i.e., G2, G5, G6, G13, G14, G15, G16, G19, G21, G36, G37, G40 and G41) leaves. *Actin* and *PRPF31* were selected as the internal standards (Fig. 7). The transcript levels of all these genes with the exceptions of G5, G14, G19, G37, S23, S35 and S40 matched well with our 2-DE data, regardless of which gene was used as the internal standard (Tables 2 and 3). In addition, there was a positive linear correlation between qRT-PCR results and 2-DE data, regardless of *actin* or *PRPF31* was used as the internal standard (Fig. 7e-f). Thus, it is reasonable to assume that these DAPs were mainly regulated in the transcriptional level.

## Discussion

### *Citrus sinensis* and *C. grandis* were tolerant to low pH

Our results clearly showed that only pH 2.5 led to significant decreases in leaf CO_2_ assimilation and stomatal conductance (Fig. 1a-b), ASC + DHA and ASC concentrations, and ASC/(ASC + DHA) ratio (Fig. 3a, b and d), and significant increases in leaf levels of nonstructural carbohydrates (Fig. 2). Based on these results, we concluded that *C. sinensis and C. grandis* seedlings were tolerant to low pH. Similar results have been obtained in grafted *Citrus unshiu* plants [27] and *C. sinensis* and *C. grandis seedlings* (seedlings should be normal type) [8]. Thus, *Citrus* are ideal materials for studying low pH-tolerance of higher plants.

We found that the pH 2.5-induced decreases of both ASC + DHA and ASC concentrations and ASC/(ASC + DHA) ratio (Fig. 3a, b and d), and increases of MDA (Fig. 4), sucrose, starch and TNC concentrations (Fig. 2c, e and f) were greater in the *C. grandis* leaves than those in the *C. sinensis* leaves. This agrees with our previous finding that *C. sinensis* seedlings were slightly tolerant to low pH than *C. grandis* ones [8]. We identified slightly more pH 2.5-responsive proteins in the *C. sinensis* leaves (49) than those in the *C. grandis* leaves (43), but much more pH 3-responsive proteins in the *C. grandis* leaves (27) than those in the *C. sinensis* leaves (11) (Tables 1, 2 and 3 and Fig. 6). The observed fewer pH 3-responsive proteins in the *C. sinensis* leaves could be explained by the slightly higher low pH-tolerance.

As shown in Tables 1, 2 and 3 and Fig. 5, we identified more pH 2.5-responsive proteins than pH 3-responsive proteins in the *C. sinensis* and *C. grandis* leaves. This agrees with our results that only pH 2.5 significantly affected leaf gas exchange, ratio of ASC/(ASC + DHA), and levels of ASC + DHA, ASC, nonstructural carbohydrates and MDA (Figs. 1, 2, 3 and 4), and the previous report that most of the physiological and biochemical indexes were altered only at pH 2.5, but almost unaltered at pH 3 or more [8]. Here, we focused mainly on the effects of pH 2.5 on *Citrus* leaf protein profiles in this paper.

### Low pH-responsive proteins related to carbohydrate and energy metabolism

As shown in Figs. 1a and 2, pH 2.5 significantly inhibited leaf photosynthesis and increased leaf accumulation of nonstructural carbohydrates. Thus, the abundances of proteins involved in carbohydrate and energy metabolism might be altered at pH 2.5. As expected, we identified four DAPs increased in abundance and 16 DAPs decreased in abundance, and two DAPs increased in abundance and eight DAPs decreased in abundance in the pH 2.5-treated *C. sinensis* and *C. grandis* leaves, respectively. Also, we obtained five DAPs increased in abundance from the pH 3-treated *C. sinensis* leaves, and one DAP increased in abundance and three DAPs decreased in abundance from the *C. grandis* leaves (Tables 2 and 3 and Fig. 6a-d). Evidently, low pH-responsive proteins related to carbohydrate and energy metabolism greatly differed between the two *Citrus* species.

In higher plants, Chl a/b-binding protein (CAB) binds to Chl and forms light harvesting complex (LHC), which functions as a light receptor. Lhca proteins are associated with the photosystem I (PSI) light-harvesting complexes (LHCI) and the Lhcb proteins are associated with the LHCII. Damkjær et al. observed that the maximum photosystem II (PSII) efficiency of dark-adapted leaves (F_v_/F_m_) dropped more in the *A. thaliana* T-DNA knockout plants lacking Lhcb3 (koLhcb3) than that in the wild type, indicating the involvement of Lhcb3 in photoacclimation [42]. We found that the abundance of Chl a-b binding protein 215 (Lhcb3, S14) was increased in the pH 2.5-treated *C. sinensis* leaves, but not in the pH 2.5-treated *C. grandis* leaves (Tables 2 and 3). This agrees with our report that the decreases of both F_v_/F_m_ and the electron transport rate (ETR) through PSII in response to pH 2.5 was slightly lower in the *C. sinensis* leaves than that in the *C. grandis* leaves [8]. Similarly, the abundances of oxygen-evolving enhancer protein 1–1 (PSBO2, S16 and 19) were enhanced only in the pH 2.5-treated *C. sinensis* leaves (Tables 2 and 3). Previous studies showed that PSBO2 was necessary for the stability of Mn cluster, the primary site of water splitting [43], and played a role in D1 dephosphorylation and turnover [44]. The increased abundances of PSBO2 might contribute to the stability of oxygen evolving complexes (OEC), as indicated by the less pronounced ΔK-band (a specific indicator of OEC) [45] in the pH 2.5-treated *C. sinensis* leaves than that in the pH 2.5-treated *C. grandis* leaves [8]. However, the abundance of Chl a-b binding protein 4 (S20, Lhca3) was decreased in the pH 2.5-treated *C. sinensis* leaves (Table 2).

The abundances of Rubisco activase 1 (S18 and S21) catalyzed the activation of Rubisco and of Rubisco subunit binding-protein β-2 subunit (60 kDa chaperonin 1, S22) involved in protein folding and stabilization were decreased in the pH 2.5-treated *C. sinensis* leaves (Table 2). Transgenic plants showed that the activation of Rubisco by Rubisco activase is necessary for CO_2_ assimilation at atmospheric CO_2_ concentrations [46–48]. Suzuki et al. observed that moderate decrease of plastid chaperonin Cpn60 level led to impaired plastid division and reduced Chl level, suggesting that plastid chaperonins Cpn60α and Cpn60β were necessary for plastid division in *A. thaliana* [49]. The observed decreases in the abundances of Rubisco activase 1 (S18 and 21) and chaperonin 60 subunit beta 1 (S21) agrees with our report that pH 2.5 decreased Rubisco activity, photosynthesis, and Chl a and Chl b levels in the *C. sinensis* leaves [8]. Similarly, Rubisco activase 1 (G12) and chaperonin 60 subunit α 1 (G10) abundances (Table 3), Rubisco activity, photosynthesis, and Chl a and Chl levels [8] were decreased in the pH 2.5-treated *C. grandis* leaves.

The abundances of ferredoxin-NADP reductase, leaf isozyme, chloroplastic (FNR2; S35 and S37) in the *C. sinensis* leaves (Table 2), and the abundances of FNR2 (G19) and Rhodanese-like domain-containing protein 4A, chloroplastic (TROL; G13) in the *C. grandis* leaves (Table 3) were decreased at pH 2.5. FNR mediates the final step of line electron flow by transferring electron from reduced ferredoxin and NADP^+^, providing NADPH for a number of reactions, including carbon fixation, Chl biosynthesis and stromal redox regulation. In *A. thaliana*, FNR exists as two isoforms: AtLFNR1 and AtLFNR2. The *Arabidopsis fnr2* RNAi mutants had lower levels of Chl and photosynthetic thylakoid proteins, decreased rate of carbon fixation than the wild type (WT) plants [50]. TROL is necessary for the maintenance of efficient linear electron flow via mediating the binding of FNR to the thylakoids. The TROL-deficient *Arabidopsis* plants had decreased ETR at high-light intensities accompanied with increased non-photochemical quenching (NPQ) [51]. The decreased abundance of TROL in the pH 2.5-treated *C. grandis* leaves agrees with the report that pH 2.5 led to decreased ETR, and increased NPQ in the *C. grandis* leaves [8]. In addition, the abundances of phosphoglycerate kinase 1 (PGK1) (S27 and S29) involved in Calvin cycle and 2-C-methyl-D-erythritol 4-phosphate cytidylyltransferase (MECT) (S23 and G16) involved in chloroplast development [52] were decreased in the pH 2.5-treated *C. sinensis* and *C. grandis* leaves (Tables 2 and 3). Based on these results, we concluded that pH 2.5 decreased the abundances of proteins related to Rubisco activation, Calvin cycle, carbon fixation, chloroplast development, Chl biosynthesis and electron transport, thus lowering Chl level, ETR and photosynthesis. Here, we first found that pH 2.5 increased the accumulation of nonstructural carbohydrates in the *C. grandis* and *C. sinensis* leaves despite decreased CO_2_ assimilation due to the prevented sink growth caused by blocked export with the exception that pH did not significantly alter sucrose level in the *C. sinensis* leaves (Figs. 1a and 2). High levels of soluble sugars, particularly hexoses, can inhibit the expression of photosynthetic genes, especially of the nuclear-encoded small subunit of Rubisco, thus lowering Rubisco level and photosynthesis [53]. The pH 2.5-induced increases of glucose and fructose levels were greater in the *C. sinensis* leaves than those in the *C. grandis* leaves, and sucrose level only increased in the pH 2.5-treated *C. grandis* leaves (Fig. 2a-c), while both CO_2_ assimilation and Rubisco activity were similar between the pH 2.5-treated *C. grandis* and *C. sinensis* leaves [8]. Thus, the pH 2.5-induced decreases of leaf Rubisco activity and CO_2_ assimilation could not explained alone by the pH 2.5-induced accumulation of soluble sugars. Interestingly, the pH 2.5-induced accumulation of starch was higher in the *C. grandis* leaves than that in the *C. sinensis* leaves (Fig. 2e). Excessive accumulation of starch can damage chloroplastic structure, thus resulting in lower CO_2_ assimilation and Chl level [54]. This agrees with the report that the pH 2.5-induced decreases of Chl a and Chl b levels were greater in the *C. grandis* leaves than those in the *C. sinensis* leaves, and that mottled bleached leaves occurred only in some pH 2.5-treated *C. grandis* leaves [8].

We found that the abundances of proteins related to tricarboxylic acid cycle (S24 and S32), glycolysis (S28) and ATP biosynthesis (S26, S30 and S36) were decreased in the pH 2.5-treated *C. sinensis* leaves (Table 2), suggesting that ATP production might be reduced in these leaves, thus resulting in a decrease in energy (ATP) level. In addition to producing ATP from ADP via utilizing proton gradient formed by photosynthetic electron transport, chloroplastic ATP synthase can catalyze ATP hydrolysis, when the transmembrane electrochemical potential gradient is small [55]. Thus, the observed lower abundance of chloroplastic ATP synthase might be of advantage to the maintenance of ATP homeostasis.

### Low pH-responsive proteins related to antioxidation and detoxification

Under high light, the amount of excess absorbed light energy was greater in the low pH-treated *Citrus* leaves, because these leaves used only less of the absorbed light energy in electron transport due to decreased ETR and CO_2_ assimilation [8]. Excess absorbed light energy can potentially trigger ROS generation. Indeed, the production of ROS (H_2_O_2_) was elevated in the low pH-treated *C. sinensis* and *C. grandis* leaves, especially in the latter [8]. To scavenge the increased production of ROS, the abundances of some proteins related to the scavenging of ROS might be increased in these leaves. Here, the abundances of four protein spots [i..e., L-ascorbate peroxidase (APX) 1, cytosolic (S6), L-ascorbate peroxidase 3, peroxisomal (S4), probable aldo-keto reductase 1 (AKR1, S5) and isoflavone reductase-like protein (IRL, S8), and of three protein spots [i.e., copper/zinc superoxide dismutase (Cu/Zn SOD) 2, chloroplastic (G2), aldehyde dehydrogenase family 2 member B4, mitochondrial (G11) and apolipoprotein D (G40)] involved in antioxidation and detoxification were increased in the pH 2.5-treated *C. sinensis* and *C. grandis* leaves, respectively (Tables 2 and 3). However, MDA concentration (Fig. 4) and electrolyte leakage [8] were elevated in the pH 2.5-treated *C. grandis* and *C. sinensis* leaves, especially in the former. Obviously, the antioxidant and detoxification system as a whole did not provide considerable protection to the pH 2.5-treated *C. grandis* and *C. sinensis* leaves against oxidative damage, which was greater in the former. This is also supported by our data that the pH 2.5-induced decrease of ASC/(ASC + DHA) ratio was greater in the *C. grandis* leaves than that in the *C. sinensis* leaves (Fig. 3d), because the ratio of ASC/(ASC + DHA) decreases in higher plants when exposed to oxidative stress [56–58]. The greater oxidative damage in the *C. grandis* leaves than that in the *C. sinensis* leaves might be related to the findings that the pH 2.5-induced production of H_2_O_2_ was greater in the *C. grandis* leaves than that in the *C. sinensis* leaves [8], that the abundances of more protein species involved in antioxidation and detoxification were decreased by pH 2.5 in the *C. grandis* (G15, G1, G3, G4, G8, G23, G39, G36 and G5) leaves than those in the *C. sinensis* leaves (S1, S9 and S10) (Tables 2 and 3), and that ASC level was lower in the pH 2.5-treated *C. grandis* leaves than that in the pH 2.5-treated *C. sinensis* leaves (Fig. 4b). As shown in Table 3, the abundance of phosphomannomutase (PMM) 1, which plays a crucial role in ASC biosynthesis in plants, was decreased in the pH 2.5-treated *C. grandis* leaves. Studies showed that ASC level in *Nicotiana benthamiana* and *Arabidopsis* leaves were decreased or increased by suppressing or overexpressing *PMM*, respectively [59, 60]. Thus, ASC biosynthesis might be impaired in the pH 2.5-treated *C. grandis* leaves. This is supported by our data that DHA + ASC and ASC levels were substantially decreased in the pH 2.5-treated *C. grandis* and *C. sinensis* leaves, especially in the former (Fig. 3a, b).

### Low pH-responsive proteins related to protein and amino acid metabolism

As shown in Tables 2 and 3, we identified one DAP increased in abundance (S40) and one DAP decreased in abundance (S43) involved in protein folding and stability, and two DAPs decreased in abundance (S41and S44) involved in proteolytic degradation in the pH 2.5-treated *C. sinensis* leaves; and five DAPs decreased in abundance involved in protein folding and stability (G23, G26 and G21) and mainly in mediating protein-protein interactions (G30 and G31), and two DAPs decreased in abundance (G20 and G29) and two DAPs increased in abundance (G24 and G6) involved in proteolytic degradation in the pH 2.5-treated *C. grandis* leaves. Evidently, pH 2.5 affected protein metabolism more in the *C. grandis* leaves than that in the *C. sinensis* leaves. This is also support by our report that the pH 2.5-induced decrease of total soluble protein level was greater in the *C. grandis* leaves than that in the *C. sinensis* leaves [8]. Similarly, amino acid metabolism was more affected by pH 2.5 in the *C. grandis* leaves than that in the *C. sinensis* leaves, as indicated by more pH 2.5-responsive proteins isolated from the *C. grandis* leaves (G25, G27, G28, G34 and G22) than those from the *C. sinensis* leaves (S50 and S51; Tables 2 and 3 and Fig. 6a-b).

### Low pH-responsive proteins related to cellular transport

Chloroplastic ferritin-1 (FER1) plays an important role in Fe homeostasis because of its ability to store large amounts of free Fe in a non-toxic form. *FER1* and *FER2*, two nuclear genes of *Clamydomonas reinhardtii* were upregulated when its cells were shifted to Fe-deficient conditions [61]. The increased abundance of FER1 (S15) in the pH 2.5-treated *C. sinensis* leaves (Table 2) might contribute to the Fe homeostasis. This is also supported by our result that pH 2.5 decreased Fe level in the *C. grandis* leaves, but not in the *C. sinensis* leaves [8]. The increased abundance of FER1 in the pH 2.5-treated *C. sinensis* leaves also agrees with the reports that *AtFER1* was induced in P-deficient *Arabidopsis* roots and leaves [62], because P level was decreased in the pH 2.5-treated *C. sinensis* leaves [8]. We found that the abundance of V-type proton ATPase (V-ATPase) catalytic subunit A isoform 1 (G41) was increased in the pH 2.5-treated *C. grandis* leaves (Table 2) accompanied by greatly decreased N, P, Ca and Mg levels and slightly decreased K level [8], as found in the P-deficient *C. grandis* and *C. sinensis* roots [63]. Transport across the tonoplast is energized by two proton pumps, the V-ATPase and the vacuolar H^+^-pyrophosphatase. Evidence shows that V-ATPase is a key regulator of intracellular ion homeostasis [64–66]. Therefore, the pH 2.5-induced increases of FER1 and V-ATPase abundances might contribute to the tolerance of *Citrus* plants to H^+^-toxicity.

### Low pH-responsive proteins related to signal transduction and jasmonic acid biosynthesis

As shown in Tables 2 and 3, the abundances of 14–3-3-like protein GF14 kappa (GRF8; S38) and plasma membrane-associated cation-binding protein 1 PCAP1 (G32) involved in signal transduction were increased and decreased in the pH 2.5-treated *C. sinensis* and *C. grandis* leaves. Similarly, we found one DAP increased in abundance and three DAPs decreased in abundance involved in jasmonic acid (JA) biosynthesis in the pH 2.5-treated *C. sinensis* (S2) and *C. grandis* (G37, G38 and G14) leaves, respectively (Tables 2 and 3). Thus, the pH 2.5-induced alterations of signal transduction and JA biosynthesis might differ between *C. sinensis* and *C. grandis* leaves.

## Conclusions

Our results demonstrated that *C. sinensis* and *C. grandis* were tolerant to low pH, with a slightly higher low pH-tolerance in the former. We first used 2-DE to investigate low pH-responsive proteins in *Citrus* leaves and identified 49 and 44 DAP spots in the pH 2.5- and/or pH 3-treated *C. sinensis* and *C. grandis* leaves, respectively. These DAPs are mainly involved in carbohydrate and energy metabolism, antioxidation and detoxification, stress response, protein and amino acid metabolisms, lipid metabolism, cellular transport, signal transduction and nucleic acid metabolism. Further analysis showed that pH 2.5 decreased the abundances of proteins related to Rubisco activation, Calvin cycle, carbon fixation, chloroplast development, Chl biosynthesis and electron transport, hence lowering Chl level, ETR and photosynthesis. The higher oxidative damage in the pH 2.5-treated *C. grandis* leaves might be due to a combination of factors including higher production of ROS, more proteins decreased in abundance involved in antioxidation and detoxification, and lower level of ASC. Protein and amino acid metabolisms were less affected in the pH 2.5-treated *C. sinensis* leaves than those in the pH 2.5-treated *C. grandis* leaves. The abundances of proteins related to JA biosynthesis and signal transduction were increased and decreased in pH 2.5-treated *C. sinensis* and *C. grandis* leaves, respectively. However, the abundances of cellular transport-related proteins: FER1 in the *C. sinensis* leaves and of V-ATPase in the *C. grandis* leaves, were enhanced at pH 2.5. Our investigation of low pH-responsive proteins and related physiological responses in *Citrus* leaves will increase our understanding of the mechanisms on low pH-toxicity and -tolerance in higher plants.

## Additional files

Additional file 1: **Table S1**. Specific primer pairs used for qRT-PCR analysis. (DOC 57 kb)

Additional file 2: **Figure S1**. Two-DE images of proteins extracted from pH 2.5- (**a**, **d**, **g**, **j**), pH 3.0- (**b**, **e**, **h**, **k**) and pH 6.0-treated (**c**, **f**, **i**, **l**) *C. grandis* (**a**-**f**) and *C. sinensis* (**g**-**l**) leaves for the other two replicates. (PDF 317 kb)

Additional file 3: **Table S2**. Master list of proteins identified in MALDI TOF/TOF MS from pH 2.5 and/or pH 3-treated *C. sinensis* leaves using 2DE and DIGE experiments. (DOC 1635 kb)

Additional file 4: **Table S3**. Master list of proteins identified in MALDI TOF/TOF MS from pH 2.5 and/or pH 3-treated *C. grandis* leaves using 2DE and DIGE experiments. (DOC 1467 kb)

Additional file 5: **Figure S2**. Close-up views of 22 DAP spots in pH 2.5, pH 3 and pH 6-treated *C. grandis* and *C. sinensis* leaves. (PDF 132 kb)

## Abbreviations

2-DE: 2-dimensional electrophoresis
AKR: Aldo-keto reductase
APX: ASC peroxidase
ASC: Ascorbate
CAB: Chl a/b-binding protein
Chl: Chlorophyll
Cpn: Chaperonin
DAP: Differentially abundant protein
DHA: Dehydroascorbate
ETR: Electron transport rate
FER1: Ferritin-1
FNR: Ferredoxin-NADP reductase
F_v_/F_m_: Maximum PSII efficiency of dark-adapted leaves
GRF8: 14–3-3-like protein GF14 kappa
IRL: Isoflavone reductase-like protein
JA: Jasmonic acid
LHC: Light harvesting complex
MDA: Malondialdehyde
MECT: 2-C-methyl-D-erythritol 4-phosphate cytidylyltransferase
MS: Mass spectrometry
NPQ: Non-photochemical quenching
OEC: Oxygen evolving complexes
PGK: Phosphoglycerate kinase
PMM: Phosphomannomutase
PRPF31: U4/U6 small nuclear ribonucleoprotein PRP31
PSBO2: Oxygen-evolving enhancer protein 1–1
PSI: Photosystem I
PSII: Photosystem II
ROS: Reactive oxygen species
Rubisco: Ribulose bisphosphate carboxylase/oxygenase
RWC: Relative water content
SOD: Superoxide dismutase
TNC: Total nonstructural carbohydrates
TROL: Rhodanese-like domain-containing protein 4A, chloroplastic

## References

[1] von Uexküll HR, Mutert E (1995). Global extent, development and economic impact of acid soils. Plant Soil.

[2] Ferguson B, Lin MH, Gresshoff PM (2013). Regulation of legume nodulation by acidic growth conditions. Plant Signal Behav.

[3] Guo JH, Liu XJ, Zhang Y, Shen JL, Han WX, Zhang WF, Christie P, Goulding KWT, Vitousek PM, Zhang FS (2010). Significant acidification in major Chinese croplands. Science.

[4] Shi QH, Zhu ZJ, Juan LI, Qian QQ (2006). Combined effects of excess Mn and low pH on oxidative stress and antioxidant enzymes in cucumber roots. Agri Sci China.

[5] Yang LT, Qi YP, Jiang HX, Chen LS (2013). Roles of organic acid anion secretion in aluminum tolerance of higher plants. Biomed Res Int.

[6] Bian M, Zhou M, Sun D, Li C (2013). Molecular approaches unravel the mechanism of acid soil tolerance in plants. Crop J.

[7] Kidd PS, Proctor J (2001). Why plants grow poorly on very acid soils: are ecologists missing the obvious?. J Exp Bot.

[8] Long A, Zhang J, Yang LT, Ye X, Lai NW, Tan LL, Lin D, Chen LS (2017). Effects of low pH on photosynthesis, related physiological parameters and nutrient profile of *Citrus*. Front Plant Sci.

[9] Koyama H, Toda T, Hara T (2001). Brief exposure to low-pH stress causes irreversible damage to the growing root in *Arabidopsis thaliana*: pectin-ca interaction may play an important role in proton rhizotoxicity. J Exp Bot.

[10] Yang M, Tan L, Xu Y, Zhao Y, Cheng F, Ye S, Jiang W (2015). Effect of low pH and aluminum toxicity on the photosynthetic characteristics of different fast-growing *Eucalyptus* vegetatively propagated clones. PLoS One.

[11] Martins N, Osório ML, Gonçalves S, Osório J, Palma T, Romano A (2013). Physiological responses of *Plantago algarbiensis* and *P. almogravensis* shoots and plantlets to low pH and aluminum stress. Acta Physiol Plant.

[12] Lazof DB, Holland MJ (1999). Evaluation of the aluminium-induced root growth inhibition in isolation from low pH effects in *Glycine max*, *Pisum sativum* and *Phaseolus vulgaris*. Aust J Plant Physiol.

[13] Samac DA, Tesfaye M (2003). Plant improvement for tolerance to aluminum in acid soils: a review. Plant Cell Tissue Organ Cult.

[14] Kamaluddin M, Zwiazek JJ (2004). Effects of root medium pH on water transport in paper birch (*Betula papyrifera*) seedlings in relation to root temperature and abscisic acid treatments. Tree Physiol.

[15] Zhang CP, Meng P, Li JZ, Wan XC (2014). Interactive effects of soil acidification and phosphorus deficiency on photosynthetic characteristics and growth in *Juglans regia* seedlings. Chin J Plant Ecol.

[16] Martins N, Osório ML, Gonçalves S, Osório J, Romano A (2013). Differences in Al tolerance between *Plantago algarbiensis* and *P. almogravensis* reflect their ability to respond to oxidative stress. Biometals.

[17] Martins N, Gonçalves S, Palma T, Romano A (2011). The influence of low pH on in vitro growth and biochemical parameters of *Plantago almogravensis* and *P. Algarbiensis*. Plant Cell Tissue Organ Cult.

[18] Martins N, Gonçalves S, Romano A (2013). Metabolism and aluminum accumulation in *Plantago almogravensis* and *P. algarbiensis* in response to low pH and aluminum stress. Biol Plant.

[19] Yang M, Huang SX, Fang SZ, Huang XL (2011). Response of seedling growth of four *Eucalyptus* clones to acid and aluminum stress. Plant Nutr Fert Sci.

[20] Graças JP, Ruiz-Romero R, Figueiredo LD, Mattiello L, Peres LEP, Vitorello VA (2016). Root growth restraint can be an acclimatory response to low pH and is associated with reduced cell mortality: a possible role of class III peroxidases and NADPH oxidases. Plant Biol.

[21] Kobayashi Y, Ohyama Y, Kobayashi Y, Ito H, Iuchi S, Fujita M, Zhao CR, Tanveer T, Ganesan M, Kobayashi M, Koyama H (2014). STOP2 activates transcription of several genes for Al- and low pH-tolerance that are regulated by STOP1 in *Arabidopsis*. Mol Plant.

[22] Anugoolprasert O, Kinoshita S, Naito H, Shimizu M, Ehara H (2012). Effect of low pH on the growth, physiological characteristics and nutrient absorption of sago palm in a hydroponic system. Plant Prod Sci.

[23] Shavrukov Y, Hirai Y (2016). Good and bad protons: genetic aspects of acidity stress responses in plants. J Exp Bot.

[24] Lager IDA, Andréasson O, Dunbar TL, Andreasson E, Escobar MA, Rasmusson AG (2010). Changes in external pH rapidly alter plant gene expression and modulate auxin and elicitor responses. Plant Cell Environ.

[25] Wang ZF, Wang ZH, Shi L, Wang LJ, Xu FS (2010). Proteomic alterations of *Brassica napus* root in response to boron deficiency. Plant Mol Biol.

[26] Peng HY, Qi YP, Lee J, Yang LT, Guo P, Jiang HX, Chen LS (2015). Proteomic analysis of *Citrus sinensis* roots and leaves in response to long-term magnesium-deficiency. BMC Genomics.

[27] Yuda E, Okamoto S (1965). The effect of soil reaction on the growth of young citrus plants. I. Forms of nitrogen fertilizer and kinds of pH adjusting agent. J Jap Soc Hort Sci.

[28] Li Y, Han MQ, Lin F, Ten Y, Lin J, Zhu DH, Guo P, Weng YB, Chen LS (2015). Soil chemical properties, ‘Guanximiyou’ pummelo leaf mineral nutrient status and fruit quality in the southern region of Fujian province, China. J Soil Sci Plant Nutr.

[29] Han S, Chen LS, Jiang HX, Smith BR, Yang LT, Xie CY (2008). Boron deficiency decreases growth and photosynthesis, and increases starch and hexoses in leaves of *Citrus* seedlings. J Plant Physiol.

[30] Chen LS, Qi YP, Liu XH (2005). Effects of aluminum on light energy utilization and photoprotective systems in *Citrus* leaves. Ann Bot.

[31] Hodges DM, DeLong JM, Forney CF, Prange RK (1999). Improving the thiobarbituric acid-reactive-substances assay for estimating lipid peroxidation in plant tissues containing anthocyanin and other interfering compounds. Planta.

[32] You X, Yang LT, Lu YB, Li H, Zhang SQ, Chen LS (2014). Proteomic changes of citrus roots in response to long-term manganese toxicity. Trees Struct Funct.

[33] Bradford MM (1976). A rapid and sensitive method for quantitation of microgram and quantities of protein utilizing the principle of protein-dye binding. Anal Biochem.

[34] Sang W, Huang ZR, Qi YP, Yang LT, Guo P, Chen LS (2015). An investigation of boron-toxicity in leaves of two citrus species differing in boron-tolerance using comparative proteomics. J Proteome.

[35] Sang W, Huang ZR, Yang LT, Guo P, Ye X, Chen LS (2017). Effects of high toxic boron concentration on protein profiles in roots of two citrus species differing in boron-tolerance revealed by a 2-DE based MS approach. Front Plant Sci.

[36] Yang LT, Liu JW, Wu YM, Qi YP, Wang JL, Lai NW, Ye X, Chen LS (2018). Proteome profile analysis of boron-induced alleviation of aluminum-toxicity in *Citrus grandis* roots. Ecotoxicol Environ Saf.

[37] Figueiredo A, Martins J, Sebastiana M, Guerreiro A, Silva A, Matos AR, Monteiro F, Pais MS, Roepstorff P, Coelho AV (2017). Specific adjustments in grapevine leaf proteome discriminating resistant and susceptible grapevine genotypes to *Plasmopara viticola*. J Proteome.

[38] Yang LT, Qi YP, Lu YB, Guo P, Sang W, Feng H, Zhang HX, Chen LS (2013). iTRAQ protein profile analysis of *Citrus sinensis* roots in response to long-term boron-deficiency. J Proteome.

[39] Guo P, Qi YP, Huang WL, Yang LT, Huang ZR, Lai NW, Chen LS (2018). Aluminum-responsive genes revealed by RNA-Seq and related physiological responses in leaves of two *Citrus* species with contrasting aluminum-tolerance. Ecotoxicol Environ Saf.

[40] Zhou CP, Qi YP, You X, Yang LT, Guo P, Ye X, Zhou XX, Ke FJ, Chen LS (2013). Leaf cDNA-AFLP analysis of two citrus species differing in manganese tolerance in response to long-term manganese-toxicity. BMC Genomics.

[41] Jones HG (1973). Limiting factors in photosynthesis. New Phytol.

[42] Damkjær JT, Kereiche S, Johnson MP, Kovacs L, Kiss AZ, Boekema EJ, Ruban AV, Horton P, Jansson S (2009). The photosystem II light-harvesting protein Lhcb3 affects the macrostructure of photosystem II and the rate of state transitions in *Arabidopsis*. Plant Cell.

[43] Yi X, McChargue M, Laborde S, Frankel LK, Bricker TM (2005). The manganese-stabilizing protein is required for photosystem II assembly/stability and photoautotrophy in higher plants. J Biol Chem.

[44] Lundin B, Hansson M, Schoefs B, Vener AV, Spetea C (2007). The *Arabidopsis* PsbO2 protein regulates dephosphorylation and turnover of the photosystem II reaction Centre D1 protein. Plant J.

[45] Srivastava A, Guisse B, Greppin H, Strasser RJ (1997). Regulation of antenna structure and electron transport in photosystem II of *Pisum sativum* under elevated temperature probed by the fast polyphasic chlorophyll a fluorescence transient: OKJIP. Biochim Biophys Bioenergetics.

[46] Salvucci ME, Portis AR, Ogren WL (1985). A soluble chloroplast protein catalyzes ribulose bisphosphate carboxylase/oxygenase activation *in vivo*. Photosynth Res.

[47] Mate CJ, von Caemmerer S, Evans JR, Hudson GS, Andrews TJ (1996). The relationship between CO_2_-assimilation rate, Rubisco carbamylation and Rubisco activase content in activase-deficient transgenic tobacco suggests a simple model of activase action. Planta.

[48] Portis AR (2003). Rubisco activase-Rubisco’s catalytic chaperone. Photosynth Res.

[49] Suzuki K, Nakanishi H, Bower J, Yoder DW, Osteryoung KW, Miyagishima SY (2009). Plastid chaperonin proteins Cpn60α and Cpn60β are required for plastid division in *Arabidopsis thaliana*. BMC Plant Biol.

[50] Lintala M, Allahverdiyeva Y, Kangasjärvi S, Lehtimäki N, Keränen M, Rintamäki E, Aro EM, Mulo P (2009). Comparative analysis of leaf-type ferredoxin-NADP^+^ oxidoreductase isoforms in *Arabidopsis thaliana*. Plant J.

[51] Jurić S, Hazler-Pilepić K, Tomašić A, Lepeduš H, Jeličić B, Puthiyaveetil S, Bionda T, Vojta L, Allen JF, Schleiff E, Fulgosi H (2009). Tethering of ferredoxin: NADP^+^ oxidoreductase to thylakoid membranes is mediated by novel chloroplast protein TROL. Plant J.

[52] Hsieh HM, Chang CY, Hsu SJ, Chen JJ (2008). Chloroplast localization of methylerythritol 4-phosphate pathway enzymes and regulation of mitochondrial genes in *ispD* and *ispE* albino mutants in *Arabidopsis*. Plant Mol Biol.

[53] Sheen J (1994). Feedback control of gene expression. Photosynth Res.

[54] Cave G, Tolley LC, Stain BR (1981). Effect of carbon dioxide enrichment on chlorophyll content, starch content and starch grain structure in *Trifolium subterraneum* leaves. Physiol Plant.

[55] Hisabori T, Konno H, Ichimura H, Strotmann H, Bald D (2002). Molecular devices of chloroplast F_1_-ATP synthase for the regulation. Biochim Biophys Acta Bioenerg.

[56] Gossett DR, Millhollon EP, Lucas MC (1994). Antioxidant responses to NaCl stress in salt-tolerant and salt-sensitive cultivars of cotton. Crop Sci.

[57] Guo P, Qi YP, Cai YT, Yang TY, Yang LT, Huang ZR, Chen LS. Aluminum effects on photosynthesis, reactive oxygen species and methylglyoxal detoxification in two *Citrus* species differing in aluminum tolerance. Tree Physiol. 2018; 10.1093/treephys/tpy035. 10.1093/treephys/tpy035 29718474

[58] Guo P, Li Q, Qi YP, Yang LT, Ye X, Chen HH, Chen LS (2017). Sulfur-mediated-alleviation of aluminum-toxicity in *Citrus grandis* seedlings. Int J Mol Sci.

[59] Hoeberichts FA, Vaeck E, Kiddle G, Coppens E, van de Cotte B, Adamantidis A, Ormenese S, Foyer CH, Zabeau M, Inzé D, Périlleux C, Breusegem FV, Vuylsteke M (2008). A temperature-sensitive mutation in the *Arabidopsis thaliana* phosphomannomutase gene disrupts protein glycosylation and triggers cell death. J Biol Chem.

[60] Qian W, Yu C, Qin H, Liu X, Zhang A, Johansen IE, Wang D (2007). Molecular and functional analysis of phosphomannomutase (PMM) from higher plants and genetic evidence for the involvement of PMM in ascorbic acid biosynthesis in *Arabidopsis* and *Nicotiana benthamiana*. Plant J.

[61] Busch A, Rimbauld B, Naumann B, Rensch S, Hippler M (2008). Ferritin is required for rapid remodeling of the photosynthetic apparatus and minimizes photo-oxidative stress in response to iron availability in *Chlamydomonas reinhardtii*. Plant J.

[62] Bournier M, Tissot N, Mari S, Boucherez J, Lacombe E, Briat JF, Gaymard F (2013). *Arabidopsis* ferritin 1 (*AtFer1*) gene regulation by the phosphate starvation response 1 (AtPHR1) transcription factor reveals a direct molecular link between iron and phosphate homeostasis. J Biol Chem.

[63] Yang LT, Jiang HX, Qi YP, Chen LS (2012). Differential expression of genes involved in alternative glycolytic pathways, phosphorus scavenging and recycling in response to aluminum and phosphorus interactions in *Citrus* roots. Mol Biol Rep.

[64] Gaxiola RA, Palmgren MG, Schumacher K (2007). Plant proton pumps. FEBS Let.

[65] Krebs M, Beyhl D, Görlich E, Al-Rasheid KAS, Marten I, Stierhof YD, Hedrich R, Schumacher K (2010). *Arabidopsis* V-ATPase activity at the tonoplast is required for efficient nutrient storage but not for sodium accumulation. Proc Natl Acad Sci U S A.

[66] Tang RJ, Liu H, Yang Y, Yang L, Gao XS, Garcia VJ, Luan S, Zhang HX (2012). Tonoplast calcium sensors CBL2 and CBL3 control plant growth and ion homeostasis through regulating V-ATPase activity in *Arabidopsis*. Cell Res.

