# Supplementary material for: Low pH-responsive proteins revealed by a 2-DE based MS approach and related physiological responses in Citrus leaves
Source: BMC Plant Biol. 2018 Sep 12;18:188. doi: 10.1186/s12870-018-1413-3 (PMC6134590; doi:10.1186/s12870-018-1413-3)
Supplement: Supplementary file 1 — Table S1. Specific primer pairs used for qRT-PCR analysis. (DOC 57 kb) [file 12870_2018_1413_MOESM1_ESM.doc]

**Additional file 1: Table S1.** Specific primer pairs used for qRT-PCR analysis.

| Spot No. | Genes | Accession No. | Forward primers (5´→3´) | Reverse primers (5´→3´) |
| --- | --- | --- | --- | --- |
| S1 | *Peroxidase 15* | orange1.1t02046.1 | CATCATTCTGGGTGTTCT | CGCTGTCTATGCTCGT |
| S4 | *L-ascorbate peroxidase 3, peroxisomal* | Cs3g19810.2 | AGACCTGAACGAAGTGG | GTAGGCAGCACGAGTAA |
| S5 | *Probable aldo-keto reductase 1* | Cs3g10670.1 | CCCATAGAGGAAACCAT | ACGACCAAGAGGACAGT |
| S6 | *L-ascorbate peroxidase 1, cytosolic* | Cs8g17370.1 | CACTGTTAGCGAGGATTA | AGGTCAGCGTAGGAGAT |
| S7 | *Epoxide hydrolase 4* | Cs2g06360.1 | CTGGCTTCAGGGCTAT | AACGGCACTCCTAATGT |
| S9 | *Glutathione S-transferase U19* | Cs5g15190.1 | GAGTGCCCCAAGTTCATAGC | AACAACCATCACAAGCCAAA |
| S10 | *Thioredoxin-2* | Cs7g13660.1 | GTCTTCTCCAACTCCACTT | GGAACTCACTTTCGGTAAT |
| S15 | *Ferritin-1, chloroplastic* | Cs6g09150.2 | ATTGGGTGGTATTTTGC | CATTGATCTGCTCGTTG |
| S26 | *ATP synthase gamma chain 1, chloroplastic* | Cs2g03080.1 | GCTGCTGAGGATGAGTT | CTGCCTGTTGTAGACGAT |
| S27 | *Phosphoglycerate kinase 1, chloroplastic* | orange1.1t03280.1 | AAACAAGTGACGGCATA | GGAGGGGCGTAGTAAA |
| S23 | *2-C-methyl-D-erythritol 4-phosphate cytidylyltransferase, chloroplastic* | Cs3g01420.1 | TGTTTACAGCGGACTTCAGG | AGTTTTGCGGTCCAGGGT |
| S35 | *Ferredoxin-NADP reductase, leaf isozyme, chloroplastic* | Cs1g25510.1 | TCCACTTCGCTTCCAACC | CATCTGCCAATGTAAGGTGTCT |
| S40 | *Peptidyl-prolyl cis-trans isomerase CYP37, chloroplastic* | Cs1g06710.1 | CCATTCTCCCTCACAAG | AAGTCCCAGCCAACCA |
| G2 | *Copper/zinc superoxide dismutase 2, chloroplastic* | Cs8g15520.1 | TCCCTCGCTTCACTCT | TAGGACCGCCATCTTC |
| G6 | *Proline iminopeptidase* | Cs8g03250.1 | AGGTCCAGGAGGAGGCACTA | CCAAGCAAGCATGAGGGGTA |
| G13 | *Rhodanese-like domain-containing protein 4A, chloroplastic* | orange1.1t00475.2 | ATGGCGGTTTAGTGCTGTTATC | AACTGGGCACTCGCATCGT |
| G15 | *Phosphomannomutase 1* | orange1.1t00331.1 | CAGCCAAGAAGAAAGGGACG | CTGTTCGCTCGGATTCAAAA |
| G16 | *2-C-methyl-D-erythritol 4-phosphate cytidylyltransferase, chloroplastic* | Cs3g01420.1 | TGTTTACAGCGGACTTCAGG | AGTTTTGCGGTCCAGGGT |
| G21 | *Peptidyl-prolyl cis-trans isomerase CYP38, chloroplastic* | Cs2g28260.1 | ACGATGGCGATGGCTAGA | TCCGTCACATAACCAAACACT |
| G36 | *Nucleoside diphosphate kinase II, chloroplastic* | Cs5g06840.1 | GTGGTCTGGTTGGCGAAAT | TGAACAGCAAGATCCCCTC |
| G40 | *Apolipoprotein D* | Cs4g01600.1 | ACTCATTCTGATAAACCCAA | CAAAGTGTAAGTAGCCCTC |
| G41 | *V-type proton ATPase catalytic subunit A isoform 1* | Cs1g10270.1 | CAAGTCTGTTTGGATGATGCG | GGTTCTCCTTCTGCTGGGTC |
| G5 | *Betaine aldehyde dehydrogenase 1, chloroplastic* | Cs5g04880.1 | AGATCGTCGGTGATATTCCAGC | CGTTTCAAGGTTTGCCAGTTC |
| G14 | *Allene oxide cyclase 1, chloroplastic* | Cs6g18900.1 | GCTTATCTTCGCTTGAG | AACCCGCTGTTATTCC |
| G19 | *Ferredoxin--NADP reductase, leaf isozyme, chloroplastic* | Cs1g25510.1 | TCCACTTCGCTTCCAACC | CATCTGCCAATGTAAGGTGTCT |
| G37 | *Lipase/lipooxygenase, PLAT/LH2 family protein* | Cs1g01370.1 | GTGACGCTCTACGATGCTGT | CCCGCTGAAAATGTCCAAA |
|  | *Actin* | Ciclev10025866m | AGAACTATGAACTGCCTGATGGC | GCTTGGAGCAAGTGCTGTGATT |
|  | *PRPF31* | Ciclev10031363m | ACTCATGGGAACGGCTGGTGGTC | TCGGCAGGCACGCATCCTTAGAG |
