# Supplementary material for: Low pH-responsive proteins revealed by a 2-DE based MS approach and related physiological responses in Citrus leaves
Source: BMC Plant Biol. 2018 Sep 12;18:188. doi: 10.1186/s12870-018-1413-3 (PMC6134590; doi:10.1186/s12870-018-1413-3)
Supplement: Supplementary file 2 — Figure S1. Two-DE images of proteins extracted from pH 2.5- (a, d, g, j), pH 3.0- (b, e, h, k) and pH 6.0-treated (c, f, i, l) C. grandis (a-f) and C. sinensis (g-l) leaves for the other two replicates. (PDF 317 kb) [file 12870_2018_1413_MOESM2_ESM.pdf]

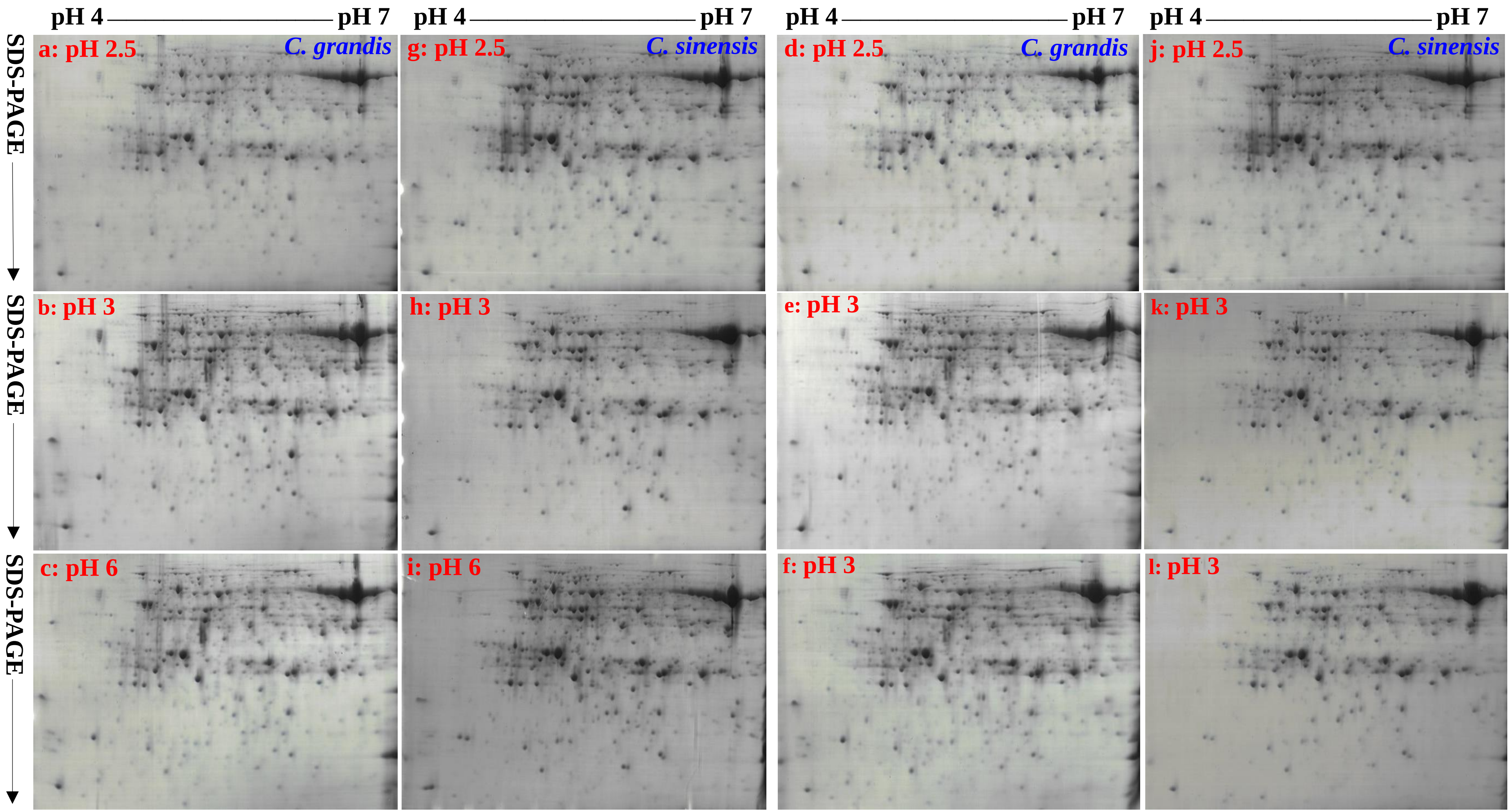

Additional file 2: Figure S1. Two-DE images of proteins extracted from pH 2.5- (**a, d, g, j**), pH 3.0- (**b, e, h, k**) and pH 6.0-treated (**c, f, i, l**) *C. grandis* (**a-f**) and *C. sinensis* (**g-l**) leaves for the other two replicates.
