# Supplementary material for: Low pH-responsive proteins revealed by a 2-DE based MS approach and related physiological responses in Citrus leaves
Source: BMC Plant Biol. 2018 Sep 12;18:188. doi: 10.1186/s12870-018-1413-3 (PMC6134590; doi:10.1186/s12870-018-1413-3)
Supplement: Supplementary file 3 — Table S2. Master list of proteins identified in MALDI TOF/TOF MS from pH 2.5 and/or pH 3-treated C. sinensis leaves using 2DE and DIGE experiments. (DOC 1635 kb) [file 12870_2018_1413_MOESM3_ESM.doc]

**Additional file 3: Table S2.** Master list of proteins identified in MALDI TOF/TOF MS from pH 2.5 and/or pH 3-treated *C. sinensis* leaves using 2DE and DIGE experiments

| | **S6** [**Cs8g17370.1**](http://zhangyang-pc/mascot/cgi/protein_view.pl?file=../data/20140118/F012093.dat&hit=1) **Mass: 27553 Score: 584 Expect: 1.8e-054 Matches: 24** | | --- | |
| --- | --- |
| | Observed | Mr(expt) | Mr(calc) | ppm | Start |  | End | Miss | Ions | Peptide | | --- | --- | --- | --- | --- | --- | --- | --- | --- | --- | | 746.3005 | 745.2933 | 745.4374 | -193.37 | 80 | - | 85 | 0 | --- | R.LLEPFK.E | | 839.2682 | 838.2610 | 838.4046 | -171.26 | 136 | - | 142 | 0 | --- | K.QGNDHLR.Q | | 883.2792 | 882.2720 | 882.4195 | -167.22 | 123 | - | 130 | 0 | --- | K.AEPPQEGR.L | | 883.2792 | 882.2720 | 882.4195 | -167.22 | 123 | - | 130 | 0 | 45 | K.AEPPQEGR.L | | 939.2778 | 938.2705 | 938.4280 | -167.87 | 53 | - | 61 | 0 | --- | K.TGGPFGTMR.L + Oxidation (M) | | 939.2778 | 938.2705 | 938.4280 | -167.87 | 53 | - | 61 | 0 | 27 | K.TGGPFGTMR.L + Oxidation (M) | | 1085.4161 | 1084.4088 | 1084.5764 | -154.54 | 200 | - | 209 | 0 | --- | K.DGLLQLPSDK.A | | 1215.3698 | 1214.3625 | 1214.5455 | -150.67 | 4 | - | 13 | 0 | --- | K.NYPTVSEDYK.K | | 1241.3837 | 1240.3764 | 1240.5684 | -154.73 | 120 | - | 130 | 1 | --- | R.DDKAEPPQEGR.L | | 1295.4873 | 1294.4800 | 1294.6994 | -169.41 | 155 | - | 167 | 0 | --- | K.DIVALSGGHTLGR.C | | 1343.4342 | 1342.4269 | 1342.6405 | -159.05 | 4 | - | 14 | 1 | --- | K.NYPTVSEDYKK.A | | 1363.4458 | 1362.4385 | 1362.7004 | -192.17 | 131 | - | 142 | 1 | --- | R.LPDAKQGNDHLR.Q | | 1611.6543 | 1610.6470 | 1610.9032 | -159.02 | 210 | - | 223 | 0 | --- | K.ALLDDPVFRPLVEK.Y | | 1611.6543 | 1610.6470 | 1610.9032 | -159.02 | 210 | - | 223 | 0 | 59 | K.ALLDDPVFRPLVEK.Y | | 1849.6619 | 1848.6546 | 1848.9442 | -156.61 | 62 | - | 79 | 0 | --- | R.LAAEQAHSANNGLDIAVR.L | | 1849.6619 | 1848.6546 | 1848.9442 | -156.61 | 62 | - | 79 | 0 | 126 | R.LAAEQAHSANNGLDIAVR.L | | 2046.5779 | 2045.5706 | 2045.9007 | -161.31 | 224 | - | 241 | 0 | --- | K.YAADEDAFFADYAEAHLK.L | | 2046.5779 | 2045.5706 | 2045.9007 | -161.31 | 224 | - | 241 | 0 | 78 | K.YAADEDAFFADYAEAHLK.L | | 2100.6985 | 2099.6912 | 2100.0171 | -155.17 | 136 | - | 154 | 1 | --- | K.QGNDHLRQVFGAQMGLSDK.D | | 2100.6985 | 2099.6912 | 2100.0415 | -166.80 | 182 | - | 199 | 0 | 55 | R.NPLIFDNSYFTELLTGEK.D | | 2245.8198 | 2244.8125 | 2245.1136 | -134.10 | 31 | - | 50 | 1 | --- | K.NCAPLMLRIAWHSAGTYDVK.T | | 2572.8816 | 2571.8743 | 2572.3068 | -168.12 | 143 | - | 167 | 1 | --- | R.QVFGAQMGLSDKDIVALSGGHTLGR.C + Oxidation (M) | | 72.8816 | 2571.8743 | 2572.3068 | -168.12 | 143 | - | 167 | 1 | 81 | R.QVFGAQMGLSDKDIVALSGGHTLGR.C + Oxidation (M) | | 3629.2029 | 3628.1956 | 3628.8202 | -172.13 | 86 | - | 119 | 0 | --- | K.EQFPTISYADLYQLAGVVGVEVTGGPDIPFHPGR.D | |
| | No match to: 700.2787, 701.2362, 705.2333, 712.2100, 713.2929, 716.2867, 718.2720, 731.2608, 734.3513, 750.3434, 752.3029, 757.3054, 758.3065, 806.3060, 822.2418, 842.3558, 844.3281, 848.3312, 850.3760, 860.9263, 861.2739, 876.8926, 897.3004, 899.3257, 905.2863, 948.3162, 955.2696, 1006.3021, 1040.3163, 1040.3163, 1045.4004, 1048.2860, 1050.3075, 1051.3121, 1052.3116, 1052.3116, 1059.3997, 1064.3134, 1066.2993, 1068.3091, 1074.3080, 1087.3479, 1091.3185, 1092.3207, 1108.3818, 1120.3485, 1122.3314, 1129.4197, 1148.3666, 1150.3260, 1206.3539, 1208.3236, 1224.3478, 1246.3883, 1351.4507, 1379.4287, 1403.4236, 1413.3818, 1431.4684, 1487.4292, 1519.4456, 1569.4768, 1610.6250, 1625.6531, 1633.6201, 1649.6049, 1660.6201, 1671.5533, 1848.6399, 1921.7328, 2028.5659, 2068.5623, 2072.6584, 2084.5305, 2122.6897, 2176.7119, 2238.7671, 2491.8684, 2508.9331, 2538.8975, 2555.8616, 2555.8616, 2571.8533, 2588.8757, 2771.8831, 3346.0938 | | | --- | --- | | **S4** [**Cs3g19810.2**](http://zhangyang-pc/mascot/cgi/protein_view.pl?file=../data/20140118/F012145.dat&hit=1) **Mass: 47301 Score: 573 Expect: 2.2e-053 Matches: 25** |  | |
| | Observed | Mr(expt) | Mr(calc) | ppm | Start |  | End | Miss | Ions | Peptide | | --- | --- | --- | --- | --- | --- | --- | --- | --- | --- | | 745.2509 | 744.2436 | 744.3878 | -193.70 | 145 | - | 152 | 0 | --- | R.GGANASLR.F | | 904.2396 | 903.2323 | 903.4086 | -195.12 | 345 | - | 352 | 0 | --- | K.DYAEAHAK.L | | 920.2404 | 919.2331 | 919.4076 | -189.72 | 297 | - | 303 | 0 | --- | K.FDNSYFK.D | | 920.2404 | 919.2331 | 919.4076 | -189.72 | 297 | - | 303 | 0 | 50 | K.FDNSYFK.D | | 947.2820 | 946.2748 | 946.5236 | -262.90 | 72 | - | 79 | 0 | --- | R.FSPLISQR.R | | 947.2820 | 946.2748 | 946.5236 | -262.90 | 72 | - | 79 | 0 | --- | R.FSPLISQR.R | | 954.3567 | 953.3494 | 953.5909 | -253.28 | 171 | - | 178 | 1 | --- | K.LIQPIKDK.Y | | 1178.4496 | 1177.4423 | 1177.6567 | -182.06 | 159 | - | 170 | 0 | --- | K.HAANAGLVNALK.L | | 1247.3344 | 1246.3271 | 1246.5506 | -179.27 | 335 | - | 344 | 0 | --- | K.YAEDQEAFFK.D | | 1247.3344 | 1246.3271 | 1246.5506 | -179.27 | 335 | - | 344 | 0 | 68 | K.YAEDQEAFFK.D | | 1309.4945 | 1308.4872 | 1308.7150 | -174.04 | 250 | - | 262 | 0 | --- | K.EIVALSGAHTVGR.S | | 1412.5133 | 1411.5060 | 1411.7572 | -177.90 | 225 | - | 238 | 0 | --- | R.LPAAGPPSPAEHLR.N | | 1412.5133 | 1411.5060 | 1411.7572 | -177.90 | 225 | - | 238 | 0 | 144 | R.LPAAGPPSPAEHLR.N | | 1416.4373 | 1415.4300 | 1415.6602 | -162.59 | 382 | - | 394 | 1 | --- | K.YSSGKSELSEAMK.Q | | 1432.4073 | 1431.4000 | 1431.6551 | -178.18 | 382 | - | 394 | 1 | --- | K.YSSGKSELSEAMK.Q + Oxidation (M) | | 1490.5288 | 1489.5215 | 1489.7889 | -179.45 | 145 | - | 158 | 1 | --- | R.GGANASLRFEVELK.H | | 1769.5265 | 1768.5192 | 1768.8519 | -188.10 | 360 | - | 376 | 0 | --- | K.FDPPEGIVLDDGAAPEK.F | | 1769.5265 | 1768.5192 | 1768.8519 | -188.10 | 360 | - | 376 | 0 | 82 | K.FDPPEGIVLDDGAAPEK.F | | 1967.5961 | 1966.5888 | 1967.0258 | -222.17 | 244 | - | 262 | 1 | --- | R.MGLNDKEIVALSGAHTVGR.S | | 1983.6632 | 1982.6559 | 1983.0208 | -183.98 | 244 | - | 262 | 1 | --- | R.MGLNDKEIVALSGAHTVGR.S + Oxidation (M) | | 1983.6632 | 1982.6559 | 1983.0208 | -183.98 | 244 | - | 262 | 1 | 76 | R.MGLNDKEIVALSGAHTVGR.S + Oxidation (M) | | 2262.7188 | 2261.7115 | 2262.1308 | -185.32 | 310 | - | 329 | 0 | --- | R.DEDLLVLPTDAVLFEDPSFK.V | | 2418.8032 | 2417.7959 | 2418.2319 | -180.27 | 309 | - | 329 | 1 | --- | R.RDEDLLVLPTDAVLFEDPSFK.V | | 2418.8032 | 2417.7959 | 2418.2319 | -180.27 | 309 | - | 329 | 1 | 86 | R.RDEDLLVLPTDAVLFEDPSFK.V | | 2558.7778 | 2557.7705 | 2558.2540 | -189.00 | 179 | - | 203 | 0 | --- | K.YSGVTYADLFQLASATAIEEAGGPK.I | |
| | No match to: 700.2349, 713.2725, 716.2277, 724.3108, 734.3439, 750.3563, 768.3775, 804.2778, 834.3056, 842.3463, 848.3326, 850.3707, 868.3744, 882.3910, 942.2233, 944.2852, 958.2595, 959.2759, 959.2759, 969.2976, 972.2994, 973.2903, 974.3409, 975.2936, 981.2457, 999.2881, 1003.2971, 1005.3125, 1051.4945, 1059.3704, 1113.3185, 1115.3346, 1128.3433, 1131.3854, 1138.4314, 1138.4314, 1149.3903, 1151.4302, 1165.5050, 1188.3982, 1229.3802, 1232.4089, 1244.3942, 1245.3933, 1261.3851, 1266.3539, 1269.3234, 1278.3469, 1292.4302, 1315.4054, 1334.4651, 1428.4385, 1430.4255, 1434.4635, 1464.4888, 1465.5094, 1467.4408, 1507.5011, 1513.4064, 1517.3918, 1556.4176, 1574.3798, 1574.3798, 1588.3999, 1590.4058, 1619.6011, 1646.6085, 1648.5852, 1791.5090, 1812.6440, 1918.7137, 1919.6357, 1979.5887, 1999.6714, 2002.6737, 2096.6653, 2220.7283, 2224.7290, 2238.7405, 2245.7883, 2337.7991, 2860.9109, 2949.8770, 2967.8225, 3346.0356 | | --- | | **S5** [**Cs3g10670.1**](http://zhangyang-pc/mascot/cgi/protein_view.pl?file=../data/20140118/F012165.dat&hit=2) **Mass: 38398 Score: 295 Expect: 1.4e-025 Matches: 15** | |
| | Observed | Mr(expt) | Mr(calc) | ppm | Start |  | End | Miss | Ions | Peptide | | --- | --- | --- | --- | --- | --- | --- | --- | --- | --- | | 821.2564 | 820.2491 | 820.3967 | -179.90 | 326 | - | 332 | 0 | --- | R.YPDALDK.T | | 868.3689 | 867.3616 | 867.3578 | 4.35 | 113 | - | 120 | 0 | --- | R.SCCEASLR.R | | 964.2665 | 963.2593 | 963.4410 | -188.63 | 240 | - | 247 | 0 | --- | R.FNGENLDR.N | | 1114.3364 | 1113.3291 | 1113.5343 | -184.22 | 55 | - | 64 | 0 | --- | K.GITFFDTADK.Y | | 1367.4447 | 1366.4374 | 1366.7132 | -201.81 | 65 | - | 76 | 0 | --- | K.YGPYTNEILLGK.A | | 1403.4921 | 1402.4848 | 1402.7708 | -203.85 | 223 | - | 235 | 0 | --- | K.AVVESVPLDSFLK.F | | 1421.4711 | 1420.4638 | 1420.7198 | -180.17 | 159 | - | 171 | 0 | --- | K.YIGLSEASPDTIR.R | | 1421.4711 | 1420.4638 | 1420.7198 | -180.17 | 159 | - | 171 | 0 | 90 | K.YIGLSEASPDTIR.R | | 1663.5066 | 1662.4993 | 1662.8022 | -182.16 | 135 | - | 149 | 0 | --- | R.VDTSVPIEETIGEMK.K + Oxidation (M) | | 1726.5432 | 1725.5359 | 1725.8362 | -174.00 | 122 | - | 134 | 0 | --- | R.LDVEYIDLYYQHR.V | | 1726.5432 | 1725.5359 | 1725.8362 | -174.00 | 122 | - | 134 | 0 | 73 | R.LDVEYIDLYYQHR.V | | 1882.5979 | 1881.5906 | 1881.9373 | -184.22 | 121 | - | 134 | 1 | --- | R.RLDVEYIDLYYQHR.V | | 1882.5979 | 1881.5906 | 1881.9373 | -184.22 | 121 | - | 134 | 1 | 53 | R.RLDVEYIDLYYQHR.V | | 2389.6841 | 2388.6768 | 2389.1285 | -189.05 | 311 | - | 332 | 1 | --- | K.EISDAVPTEEVAGDRYPDALDK.T | | 2389.6841 | 2388.6768 | 2389.1285 | -189.05 | 311 | - | 332 | 1 | 43 | K.EISDAVPTEEVAGDRYPDALDK.T | |
| | No match to: 706.2463, 713.2891, 716.1478, 725.2515, 734.3337, 736.3187, 743.2224, 747.2499, 750.3845, 757.2969, 768.3635, 783.2018, 836.3063, 842.3472, 848.3567, 850.3522, 860.8978, 875.2459, 882.3821, 947.2542, 982.3628, 995.2977, 998.3220, 1017.4405, 1059.3768, 1067.3038, 1081.3717, 1087.3057, 1119.3340, 1142.3977, 1158.3970, 1174.3157, 1179.3843, 1196.3517, 1233.3776, 1233.3776, 1234.3701, 1235.3668, 1266.4446, 1293.3925, 1293.3925, 1295.4054, 1308.4070, 1309.4194, 1315.4014, 1319.4628, 1337.3882, 1359.4191, 1389.4462, 1434.5365, 1436.4379, 1478.5439, 1485.5454, 1485.5454, 1506.4537, 1541.5089, 1559.5271, 1559.5271, 1578.5518, 1580.4833, 1581.5171, 1582.4618, 1599.4794, 1642.5518, 1642.5518, 1653.4755, 1658.5461, 1772.5731, 1865.6556, 1886.6257, 1887.6442, 1888.6354, 1904.5560, 1908.7184, 1932.5256, 1960.6196, 1964.6638, 1994.5900, 1997.7147, 2000.7150, 2010.6729, 2010.6729, 2014.6611, 2172.6877, 2186.6677, 2189.5217, 2192.6724, 2217.7485, 2232.7346, 2235.7329, 2238.7136, 2377.6604, 2388.7092, 2411.7090, 3001.8938 | | --- | | **S8** [**Cs2g16220.1**](http://zhangyang-pc/mascot/cgi/protein_view.pl?file=../data/20140118/F012115.dat&hit=1) **Mass: 34230 Score: 155 Expect: 1.4e-011 Matches: 17** | |
| | Observed | Mr(expt) | Mr(calc) | ppm | Start |  | End | Miss | Ions | Peptide | | --- | --- | --- | --- | --- | --- | --- | --- | --- | --- | | 712.2056 | 711.1984 | 711.3439 | -204.63 | 129 | - | 134 | 0 | --- | K.STYDVK.A | | 892.3458 | 891.3385 | 891.5066 | -188.53 | 19 | - | 26 | 0 | --- | K.FIVEASVK.A | | 987.3510 | 986.3437 | 986.5185 | -177.24 | 46 | - | 53 | 0 | --- | K.SQLLDHFK.N | | 1096.4377 | 1095.4304 | 1095.6189 | -172.05 | 27 | - | 36 | 0 | --- | K.AGHPTFVLVR.E | | 1178.4641 | 1177.4568 | 1177.6707 | -181.58 | 7 | - | 18 | 0 | --- | K.ILSIGGTGYIGK.F | | 1237.4137 | 1236.4064 | 1236.6237 | -175.74 | 242 | - | 251 | 0 | --- | K.EYVSEEQLLK.N | | 1429.4045 | 1428.3972 | 1428.6310 | -163.64 | 108 | - | 119 | 0 | --- | R.FFPSEFGNDVDR.V | | 1429.4045 | 1428.3972 | 1428.6310 | -163.64 | 108 | - | 119 | 0 | 35 | R.FFPSEFGNDVDR.V | | 1491.4194 | 1490.4121 | 1490.6929 | -188.37 | 298 | - | 309 | 0 | --- | K.YTTVDEYLNQFV.- | | 1630.5057 | 1629.4984 | 1629.8362 | -207.27 | 120 | - | 134 | 1 | --- | R.VHGAVEPTKSTYDVK.A | | 1713.5602 | 1712.5529 | 1712.8621 | -180.51 | 187 | - | 201 | 1 | --- | K.AVFNKEDDIGTYTIK.A | | 1713.5602 | 1712.5529 | 1712.8621 | -180.51 | 187 | - | 201 | 1 | --- | K.AVFNKEDDIGTYTIK.A | | 1878.6185 | 1877.6112 | 1877.9411 | -175.65 | 281 | - | 297 | 0 | --- | K.IEPSFGVEASQLYPDVK.Y | | 1878.6185 | 1877.6112 | 1877.9411 | -175.65 | 281 | - | 297 | 0 | 56 | K.IEPSFGVEASQLYPDVK.Y | | 2021.7524 | 2020.7451 | 2021.1157 | -183.37 | 76 | - | 94 | 0 | --- | K.QVDVVISTVGHTLIADQVK.I | | 2066.7578 | 2065.7505 | 2066.1160 | -176.91 | 54 | - | 72 | 0 | --- | K.NLGVNFLVGDVLNHESLVK.A | | 2799.9114 | 2798.9041 | 2799.4020 | -177.86 | 212 | - | 234 | 1 | --- | K.NFYIQPPGNIYSFNDLVSLWERK.I | |
| | No match to: 700.2657, 705.2568, 706.2521, 713.2783, 716.2333, 720.2239, 727.2487, 730.2582, 733.2313, 734.3519, 738.2461, 744.2362, 752.2395, 758.2497, 761.3115, 768.3749, 807.2845, 825.2732, 834.3217, 842.3544, 848.3318, 850.3696, 860.9185, 868.3812, 876.8917, 882.4052, 996.4020, 1011.4013, 1025.2749, 1051.4762, 1059.3871, 1126.3794, 1127.4360, 1127.4360, 1147.4370, 1149.3923, 1151.4492, 1152.4257, 1159.4056, 1165.4305, 1165.4305, 1179.4609, 1187.4069, 1251.4303, 1251.4303, 1273.3981, 1286.3782, 1304.4276, 1316.3979, 1348.4603, 1424.4098, 1426.3953, 1434.5630, 1439.4565, 1443.3765, 1444.3873, 1444.3873, 1448.4066, 1458.3993, 1464.4789, 1466.4108, 1498.5265, 1513.4248, 1529.3981, 1536.4567, 1600.4534, 1637.5854, 1637.5854, 1651.5859, 1735.5475, 1831.6248, 1900.6089, 1916.6057, 1960.6643, 1988.7035, 2002.7108, 2004.7223, 2053.7070, 2110.6375, 2115.7363, 2220.7214, 2238.7537, 2245.7825, 2328.7195, 2346.7290, 2346.7290, 2360.7510, 2397.8572, 2397.8572, 2411.8386, 2535.8662, 2536.8643, 3729.1853 | | | | --- | --- | --- | |  | **S1** [**orange1.1t02046.1**](http://zhangyang-pc/mascot/cgi/protein_view.pl?file=../data/20140118/F012156.dat&hit=1) **Mass: 37410 Score: 178 Expect: 7e-014 Matches: 9** |  | |
| | Observed | Mr(expt) | Mr(calc) | ppm | Start |  | End | Miss | Ions | Peptide | | --- | --- | --- | --- | --- | --- | --- | --- | --- | --- | | 773.3194 | 772.3121 | 772.4807 | -218.18 | 57 | - | 63 | 0 | --- | R.ITASLIR.L | | 1011.2755 | 1010.2682 | 1010.4743 | -203.93 | 104 | - | 112 | 0 | --- | R.GFEVVDAMK.A + Oxidation (M) | | 1114.2937 | 1113.2864 | 1113.5026 | -194.11 | 203 | - | 211 | 0 | --- | R.AQCQFFSQR.L | | 1605.5028 | 1604.4955 | 1604.8192 | -201.69 | 311 | - | 325 | 0 | --- | R.MGNLSLLTGTQGEIR.S + Oxidation (M) | | 2013.6719 | 2012.6646 | 2013.0782 | -205.47 | 160 | - | 177 | 0 | --- | R.SLADQNLPTPFQTLDLLK.G | | 2013.6719 | 2012.6646 | 2013.0782 | -205.47 | 160 | - | 177 | 0 | 71 | R.SLADQNLPTPFQTLDLLK.G | | 2418.7170 | 2417.7097 | 2418.1928 | -199.77 | 180 | - | 202 | 0 | --- | R.FTNVGLNDNTDLVALSGAHTFGR.A | | 2418.7170 | 2417.7097 | 2418.1928 | -199.77 | 180 | - | 202 | 0 | 90 | R.FTNVGLNDNTDLVALSGAHTFGR.A | | 2456.6589 | 2455.6516 | 2455.3071 | 140 | 156 | - | 177 | 1 | --- | R.TANRSLADQNLPTPFQTLDLLK.G | |
| | No match to: 705.2402, 713.2695, 716.2217, 727.2257, 729.2399, 730.2282, 734.3507, 750.3426, 757.2557, 768.3620, 795.2520, 801.3309, 812.2311, 813.2569, 822.2461, 832.2284, 838.2470, 842.3346, 848.3359, 850.3637, 860.9049, 864.3199, 868.3604, 876.8737, 882.2466, 883.2374, 940.2236, 1033.2782, 1051.4613, 1059.3513, 1063.2638, 1073.3793, 1074.3942, 1076.2646, 1079.2760, 1080.3054, 1080.3054, 1096.3019, 1097.3105, 1102.2957, 1125.3040, 1128.2969, 1129.2794, 1130.2882, 1130.2882, 1151.4636, 1169.2764, 1171.3068, 1171.3068, 1179.3807, 1186.1935, 1187.2667, 1187.2667, 1201.3394, 1202.3164, 1203.2950, 1209.2788, 1224.3428, 1315.3877, 1319.3884, 1334.5570, 1342.4575, 1342.4575, 1503.4159, 1940.5389, 1957.5460, 1993.6124, 2002.6687, 2035.6680, 2051.6531, 2056.6208, 2205.6130, 2220.6863, 2238.6924, 2245.7383, 2297.4382, 2313.4536, 2374.7017, 2383.4980, 2400.7410, 2400.7410, 2403.7329, 2415.6716, 2417.7212, 2434.7134, 2440.6934, 2476.7356, 2524.5981, 2565.7317, 2613.6887, 2629.6511, 2681.7222, 2710.7468, 2711.7468, 2711.7468, 2775.6677, 2775.6677, 2791.6838, 2809.6851, 3108.6658, 3346.0010 | | --- | | **S9** [**Cs5g15190.1**](http://zhangyang-pc/mascot/cgi/protein_view.pl?file=../data/20140118/F012158.dat&hit=1) **Mass: 25542 Score: 148 Expect: 7e-011 Matches: 14** | |
| | Observed | Mr(expt) | Mr(calc) | ppm | Start |  | End | Miss | Ions | Peptide | | --- | --- | --- | --- | --- | --- | --- | --- | --- | --- | | 911.2974 | 910.2901 | 910.4800 | -208.54 | 127 | - | 133 | 0 | --- | K.EFFEVLK.T | | 1085.3361 | 1084.3288 | 1084.5376 | -192.46 | 205 | - | 213 | 0 | --- | K.VFEFVSAMR.T | | 1101.3354 | 1100.3281 | 1100.5325 | -185.68 | 205 | - | 213 | 0 | --- | K.VFEFVSAMR.T + Oxidation (M) | | 1213.4117 | 1212.4044 | 1212.6325 | -188.09 | 204 | - | 213 | 1 | --- | K.KVFEFVSAMR.T | | 1216.3652 | 1215.3579 | 1215.5659 | -171.08 | 30 | - | 38 | 1 | --- | K.YEYKEEDLK.N | | 1229.4010 | 1228.3937 | 1228.6274 | -190.21 | 204 | - | 213 | 1 | --- | K.KVFEFVSAMR.T + Oxidation (M) | | 1256.4301 | 1255.4228 | 1255.6561 | -185.75 | 82 | - | 92 | 0 | --- | K.APLLPSDPYQR.A | | 1256.4301 | 1255.4228 | 1255.6561 | -185.75 | 82 | - | 92 | 0 | 71 | K.APLLPSDPYQR.A | | 1392.5118 | 1391.5045 | 1391.7595 | -183.20 | 41 | - | 52 | 0 | --- | K.SPLLLQMNPVHK.K + Oxidation (M) | | 1499.5186 | 1498.5113 | 1498.7780 | -177.91 | 80 | - | 92 | 1 | --- | K.DKAPLLPSDPYQR.A | | 1499.5186 | 1498.5113 | 1498.7780 | -177.91 | 80 | - | 92 | 1 | 57 | K.DKAPLLPSDPYQR.A | | 2124.6023 | 2123.5950 | 2124.0536 | -215.91 | 1 | - | 18 | 0 | --- | -.MANEVVLLDFWPSMFGIR.V | | 2125.6838 | 2124.6765 | 2124.0536 | 293 | 1 | - | 18 | 0 | --- | -.MANEVVLLDFWPSMFGIR.V | | 2125.6838 | 2124.6765 | 2124.0536 | 293 | 1 | - | 18 | 0 | --- | -.MANEVVLLDFWPSMFGIR.V | |
| | No match to: 718.2645, 731.2885, 741.2589, 767.2781, 774.1531, 809.2476, 823.2211, 842.3402, 848.3061, 943.3265, 1018.4283, 1065.3651, 1073.3326, 1082.2903, 1095.3665, 1129.3597, 1129.3597, 1145.3464, 1201.4077, 1204.4044, 1212.3959, 1249.3794, 1255.4070, 1261.4674, 1278.4053, 1458.4884, 1488.4777, 1527.5006, 1539.4967, 1539.4967, 1549.5050, 1555.4919, 1561.4928, 1577.4584, 1660.5990, 1661.6013, 1661.6013, 1683.5800, 1703.5310, 1723.7000, 1744.6372, 1766.6316, 1853.5530, 1865.5465, 1904.5638, 1921.6941, 1921.6941, 1937.6340, 1966.6495, 1969.7275, 1971.5664, 1983.5452, 1992.6620, 1999.5831, 2002.6720, 2004.6482, 2020.6394, 2021.6016, 2033.5653, 2038.6495, 2052.6106, 2056.6355, 2056.6355, 2061.6057, 2062.5313, 2063.5618, 2066.5776, 2068.6248, 2068.6248, 2072.6274, 2075.5913, 2078.5383, 2083.6211, 2084.6257, 2088.6248, 2090.5437, 2094.5730, 2098.6509, 2106.5747, 2109.5281, 2111.6035, 2138.6074, 2153.5178, 2164.6172, 2198.6252, 2215.6956, 2216.6909, 2216.6909, 2230.6338, 2232.6348, 2238.7222, 2435.8091, 2987.0063, 2999.0620, 2999.7839, 3353.0837 | | --- | | **S10** [**Cs7g13660.1**](http://zhangyang-pc/mascot/cgi/protein_view.pl?file=../data/20140118/F012091.dat&hit=1) **Mass: 21429 Score: 178 Expect: 7e-014 Matches: 7** | |
| | Observed | Mr(expt) | Mr(calc) | ppm | Start |  | End | Miss | Ions | Peptide | | --- | --- | --- | --- | --- | --- | --- | --- | --- | --- | | 774.3032 | 773.2959 | 773.4395 | -185.72 | 169 | - | 175 | 1 | --- | R.REGAITK.L | | 1015.3178 | 1014.3105 | 1014.4730 | -160.18 | 160 | - | 168 | 0 | --- | K.NGQEVPESR.R | | 1015.3178 | 1014.3105 | 1014.4730 | -160.18 | 160 | - | 168 | 0 | 47 | K.NGQEVPESR.R | | 1263.5627 | 1262.5554 | 1262.7638 | -165.05 | 149 | - | 159 | 0 | --- | K.VYGLPTLILFK.N | | 1263.5627 | 1262.5554 | 1262.7638 | -165.05 | 149 | - | 159 | 0 | 56 | K.VYGLPTLILFK.N | | 1684.5475 | 1683.5402 | 1683.8104 | -160.44 | 135 | - | 148 | 0 | --- | K.IDHDANPQLIEEYK.V | | 1684.5475 | 1683.5402 | 1683.8104 | -160.44 | 135 | - | 148 | 0 | 62 | K.IDHDANPQLIEEYK.V | |
| | No match to: 701.1859, 704.2697, 705.2588, 707.2249, 709.2194, 713.2740, 715.2167, 716.2744, 721.2363, 730.2612, 734.3104, 742.1957, 745.2084, 752.2740, 753.2836, 780.2242, 782.2895, 818.3308, 825.2789, 835.2576, 837.2610, 842.3641, 848.3245, 864.3115, 868.3394, 951.2968, 951.2968, 996.3502, 1016.3114, 1029.3417, 1034.3553, 1059.3759, 1075.4156, 1075.4156, 1089.3828, 1168.3634, 1192.4160, 1198.4579, 1224.4659, 1245.4933, 1285.5084, 1316.4376, 1358.5585, 1359.5227, 1404.3870, 1485.5345, 1560.5662, 1666.5349, 1706.5216, 1722.5212, 1745.5927, 1757.6074, 1802.6442, 1818.6267, 1820.6409, 1820.6409, 1829.6382, 1830.6434, 1831.6432, 1832.6416, 1832.6416, 1836.6572, 1846.6360, 1848.6320, 1848.6320, 1852.6326, 1860.6390, 1863.6216, 1865.6177, 1872.6400, 1886.6204, 1889.6455, 1891.6473, 1898.6318, 1901.6077, 1916.6565, 1929.6517, 1957.6506, 1972.6860, 1984.6459, 1986.6643, 1988.6119, 1993.5780, 1996.6342, 2001.6416, 2002.7101, 2004.7153, 2010.6652, 2022.6846, 2022.6846, 2030.6932, 2036.6833, 2044.6738, 2060.6545, 2079.6960, 2079.6960, 2097.6602, 2102.7124, 2117.6514, 2220.7368, 2238.7585, 2245.7944, 2451.7712 | | --- | | **S11** [**Cs5g03150.1**](http://zhangyang-pc/mascot/cgi/protein_view.pl?file=../data/20140118/F012184.dat&hit=5) **Mass: 80473 Score: 186 Expect: 1.1e-014 Matches: 20** | |
| | Observed | Mr(expt) | Mr(calc) | ppm | Start |  | End | Miss | Ions | Peptide | | --- | --- | --- | --- | --- | --- | --- | --- | --- | --- | | 700.2553 | 699.2480 | 699.4279 | -257.16 | 433 | - | 438 | 0 | --- | K.LADLLR.F | | 734.3273 | 733.3200 | 733.4738 | -209.70 | 179 | - | 184 | 0 | --- | K.ITLFLK.E | | 1020.2684 | 1019.2611 | 1019.4295 | -165.13 | 525 | - | 532 | 0 | --- | K.LEEDEEEK.K | | 1020.2684 | 1019.2611 | 1019.4295 | -165.13 | 525 | - | 532 | 0 | --- | K.LEEDEEEK.K | | 1081.3240 | 1080.3167 | 1080.5240 | -191.84 | 321 | - | 329 | 0 | --- | R.APFDLFDTR.K | | 1081.3240 | 1080.3167 | 1080.5240 | -191.84 | 321 | - | 329 | 0 | 39 | R.APFDLFDTR.K | | 1161.3745 | 1160.3672 | 1160.5826 | -185.55 | 475 | - | 484 | 0 | --- | K.AVENSPFLER.L | | 1168.3561 | 1167.3488 | 1167.5632 | -183.64 | 421 | - | 430 | 0 | --- | K.LGIHEDSQNR.A | | 1201.4072 | 1200.3999 | 1200.6503 | -208.49 | 62 | - | 71 | 0 | --- | K.LDAQPELFIR.I | | 1237.3954 | 1236.3881 | 1236.6251 | -191.65 | 320 | - | 329 | 1 | --- | R.RAPFDLFDTR.K | | 1237.3954 | 1236.3881 | 1236.6251 | -191.65 | 320 | - | 329 | 1 | 53 | R.RAPFDLFDTR.K | | 1256.4636 | 1255.4563 | 1255.6884 | -184.84 | 93 | - | 104 | 0 | --- | K.ADLVNNLGTIAR.S | | 1256.4636 | 1255.4563 | 1255.6884 | -184.84 | 93 | - | 104 | 0 | 76 | K.ADLVNNLGTIAR.S | | 1289.4242 | 1288.4169 | 1288.6775 | -202.22 | 474 | - | 484 | 1 | --- | R.KAVENSPFLER.L | | 1320.4047 | 1319.3974 | 1319.6510 | -192.15 | 302 | - | 312 | 0 | --- | K.HFSVEGQLEFK.A | | 1527.5188 | 1526.5115 | 1526.7365 | -147.36 | 289 | - | 301 | 0 | --- | K.SLTNDWEDHLAVK.H | | 1527.5188 | 1526.5115 | 1526.7365 | -147.36 | 289 | - | 301 | 0 | 5 | K.SLTNDWEDHLAVK.H | | 1748.6169 | 1747.6096 | 1747.7869 | -101.42 | 590 | - | 605 | 1 | --- | K.AQALRDSSMSSYMSSK.K | | 1748.6169 | 1747.6096 | 1747.7869 | -101.42 | 590 | - | 605 | 1 | --- | K.AQALRDSSMSSYMSSK.K | | 1847.5117 | 1846.5044 | 1846.8988 | -213.56 | 274 | - | 288 | 1 | --- | R.KPEEVTKEEYASFYK.S | |
| | No match to: 713.2690, 714.2902, 716.2282, 724.3184, 750.3489, 766.2210, 768.3619, 787.3397, 797.2162, 835.2664, 842.3379, 848.3348, 850.3486, 858.2963, 868.3638, 876.8698, 882.3692, 907.2427, 931.2752, 947.2944, 961.3038, 973.2998, 978.3196, 989.3021, 990.3233, 1051.4844, 1059.3593, 1075.3245, 1077.3251, 1092.3180, 1106.3196, 1113.3235, 1124.3960, 1133.3765, 1151.4335, 1165.4408, 1179.3809, 1187.4171, 1219.3715, 1220.3700, 1235.3402, 1261.4243, 1265.4111, 1275.3618, 1282.4142, 1304.4307, 1305.3752, 1323.3627, 1323.3627, 1331.4021, 1334.5239, 1342.3771, 1343.3965, 1348.4684, 1365.3990, 1397.3610, 1455.4926, 1465.4800, 1480.4446, 1493.4347, 1496.4390, 1541.5176, 1545.4602, 1557.4686, 1564.5000, 1564.5000, 1616.4342, 1616.4342, 1643.4521, 1680.5129, 1707.4725, 1717.5173, 1783.4988, 1786.5065, 1791.4049, 1845.5654, 1845.5654, 1863.5095, 1891.4999, 1911.5594, 1975.5592, 1991.5623, 2002.6912, 2082.5930, 2103.6006, 2140.6035, 2142.5549, 2220.6631, 2238.6951, 2383.4990 | | --- | | **S12** [**Cs1g06050.1**](http://zhangyang-pc/mascot/cgi/protein_view.pl?file=../data/20140118/F012194.dat&hit=1) **Mass: 27745 Score: 265 Expect: 1.4e-022 Matches: 11** | |
| | Observed | Mr(expt) | Mr(calc) | ppm | Start |  | End | Miss | Ions | Peptide | | --- | --- | --- | --- | --- | --- | --- | --- | --- | --- | | 811.2372 | 810.2300 | 810.3912 | -198.95 | 124 | - | 130 | 0 | --- | K.EAGFPYK.I | | 872.2830 | 871.2757 | 871.4287 | -175.60 | 198 | - | 205 | 0 | --- | K.DDGEPLVK.V | | 1133.4492 | 1132.4419 | 1132.6274 | -163.77 | 150 | - | 160 | 0 | --- | R.LSLSAVIMGSR.G | | 1149.4199 | 1148.4126 | 1148.6223 | -182.57 | 150 | - | 160 | 0 | --- | R.LSLSAVIMGSR.G + Oxidation (M) | | 1149.4199 | 1148.4126 | 1148.6223 | -182.57 | 150 | - | 160 | 0 | 34 | R.LSLSAVIMGSR.G + Oxidation (M) | | 1490.4360 | 1489.4287 | 1489.6936 | -177.84 | 193 | - | 205 | 1 | --- | R.YPDDKDDGEPLVK.V | | 1490.4360 | 1489.4287 | 1489.6936 | -177.84 | 193 | - | 205 | 1 | 69 | R.YPDDKDDGEPLVK.V | | 1530.4122 | 1529.4049 | 1529.6886 | -185.44 | 102 | - | 114 | 0 | --- | K.QLEDDFDTFTATK.A | | 1530.4122 | 1529.4049 | 1529.6886 | -185.44 | 102 | - | 114 | 0 | 56 | K.QLEDDFDTFTATK.A | | 1648.5586 | 1647.5513 | 1647.8468 | -179.31 | 37 | - | 52 | 0 | --- | K.IGVAVDLSDESAFAVR.W | | 1648.5586 | 1647.5513 | 1647.8468 | -179.31 | 37 | - | 52 | 0 | 87 | K.IGVAVDLSDESAFAVR.W | |
| | No match to: 700.2443, 705.2203, 709.2308, 713.2739, 716.2258, 720.2227, 724.3467, 730.2414, 734.3422, 736.3556, 738.2551, 744.2531, 750.3616, 752.2778, 757.2576, 768.3752, 834.3360, 841.3094, 842.3412, 848.3346, 850.3573, 851.3467, 859.2981, 860.9098, 864.3462, 868.3707, 876.8757, 882.3889, 895.2492, 920.2371, 932.3210, 948.2918, 956.2891, 959.2822, 973.3591, 982.3739, 996.3554, 1017.4696, 1031.3461, 1045.3362, 1051.5026, 1059.3730, 1059.3730, 1085.3799, 1107.3679, 1126.3367, 1128.3386, 1138.4194, 1151.4540, 1165.4637, 1179.3871, 1247.3403, 1278.3533, 1315.4058, 1331.3907, 1334.6072, 1349.3934, 1349.3934, 1365.3978, 1383.4376, 1412.5106, 1424.5181, 1428.3994, 1434.5573, 1444.4662, 1448.6235, 1512.4653, 1513.3887, 1513.3887, 1521.5867, 1535.3866, 1541.5496, 1552.4015, 1570.5043, 1574.4252, 1601.5781, 1617.7178, 1707.4636, 1725.4397, 1825.4998, 1865.5688, 2002.6602, 2173.6680, 2177.6216, 2197.6843, 2215.6968, 2216.6973, 2217.7100, 2218.7263, 2218.7263, 2224.7156, 2238.7236, 2241.6389, 2241.6389, 2245.7056, 2257.6294, 2257.6294, 2279.6187, 2383.5410 | | --- | | **S13** [**Cs3g21500.1**](http://zhangyang-pc/mascot/cgi/protein_view.pl?file=../data/20140118/F012120.dat&hit=1) **Mass: 20038 Score: 411 Expect: 3.5e-037 Matches: 14** | |
| | Observed | Mr(expt) | Mr(calc) | ppm | Start |  | End | Miss | Ions | Peptide | | --- | --- | --- | --- | --- | --- | --- | --- | --- | --- | | 861.2325 | 860.2252 | 860.3889 | -190.26 | 128 | - | 134 | 0 | --- | K.DPEHAHR.H | | 1042.2531 | 1041.2458 | 1041.4363 | -182.89 | 165 | - | 173 | 0 | --- | K.EEDQEAHGK.K | | 1087.3113 | 1086.3040 | 1086.4869 | -168.34 | 88 | - | 95 | 1 | --- | K.EEEFDYKK.E | | 1353.3705 | 1352.3632 | 1352.5957 | -171.84 | 75 | - | 86 | 0 | --- | R.RPSETESGDYGR.K | | 1353.3705 | 1352.3632 | 1352.5957 | -171.84 | 75 | - | 86 | 0 | 83 | R.RPSETESGDYGR.K | | 1423.4512 | 1422.4439 | 1422.6919 | -174.28 | 6 | - | 16 | 0 | --- | K.HHHHGLFHHHK.E | | 1423.4512 | 1422.4439 | 1422.6919 | -174.28 | 6 | - | 16 | 0 | 67 | K.HHHHGLFHHHK.E | | 1509.4419 | 1508.4346 | 1508.6968 | -173.75 | 74 | - | 86 | 1 | --- | R.RRPSETESGDYGR.K | | 1509.4419 | 1508.4346 | 1508.6968 | -173.75 | 74 | - | 86 | 1 | 48 | R.RRPSETESGDYGR.K | | 2133.7510 | 2132.7437 | 2133.1218 | -177.25 | 102 | - | 122 | 0 | --- | K.HLEHLGELGTAGAGAFALLEK.H | | 2506.7515 | 2505.7442 | 2506.1877 | -176.94 | 137 | - | 160 | 0 | --- | K.IEEEIAAAAAVGSGGFAFHEHHEK.K | | 2506.7515 | 2505.7442 | 2506.1877 | -176.94 | 137 | - | 160 | 0 | 72 | K.IEEEIAAAAAVGSGGFAFHEHHEK.K | | 2771.8523 | 2770.8450 | 2771.3415 | -179.16 | 135 | - | 160 | 1 | --- | R.HKIEEEIAAAAAVGSGGFAFHEHHEK.K | | 2771.8523 | 2770.8450 | 2771.3415 | -179.16 | 135 | - | 160 | 1 | 92 | R.HKIEEEIAAAAAVGSGGFAFHEHHEK.K | |
| | No match to: 700.2503, 705.2032, 709.2642, 713.2759, 717.2289, 734.3495, 744.1643, 755.2005, 761.1892, 768.3778, 823.2340, 824.2322, 842.3522, 848.3134, 868.3841, 943.3455, 1036.3662, 1059.3805, 1081.3679, 1093.3270, 1109.3151, 1158.3353, 1197.2947, 1215.3651, 1326.4038, 1335.3386, 1336.3384, 1337.3480, 1343.4861, 1347.2968, 1349.4032, 1361.4819, 1361.4819, 1367.3959, 1375.3655, 1386.3495, 1405.4259, 1421.4083, 1422.4243, 1437.4233, 1443.4832, 1444.4950, 1445.4530, 1454.3870, 1461.4166, 1463.4221, 1465.4779, 1474.5266, 1480.4672, 1483.4436, 1486.5137, 1488.5055, 1491.4874, 1492.4552, 1502.5059, 1524.4209, 1534.4790, 1561.5151, 1561.5151, 1579.5148, 1579.5148, 1593.5171, 1601.5076, 1617.4980, 1619.5607, 1665.6001, 1668.4489, 1675.5601, 1677.5822, 1693.5808, 1739.5511, 1739.5511, 1755.5422, 1756.5636, 1809.6366, 1833.6942, 1858.6292, 1907.6221, 1937.7068, 1938.6864, 1949.4647, 2072.6094, 2080.7849, 2146.7463, 2238.7590, 2528.7129, 2660.5923, 2660.5923, 2717.7888, 2769.8987, 2785.8484, 2793.8391, 2809.8093, 2828.8284, 3328.7732, 3381.7354 | | --- | | **S17** [**Cs4g11090.1**](http://zhangyang-pc/mascot/cgi/protein_view.pl?file=../data/20140118/F012189.dat&hit=1) **Mass: 37571 Score: 304 Expect: 1.8e-026 Matches: 15** | |
| | Observed | Mr(expt) | Mr(calc) | ppm | Start |  | End | Miss | Ions | Peptide | | --- | --- | --- | --- | --- | --- | --- | --- | --- | --- | | 910.3531 | 909.3458 | 909.5760 | -253.02 | 180 | - | 187 | 0 | --- | K.LLARPNVK.L | | 1290.4854 | 1289.4781 | 1289.6359 | -122.36 | 254 | - | 265 | 0 | --- | K.SIGMIEEVPGMK.A | | 1290.4854 | 1289.4781 | 1289.6979 | -170.44 | 65 | - | 75 | 1 | 46 | K.FDPIKESIVSR.E | | 1322.4154 | 1321.4081 | 1321.6258 | -164.68 | 254 | - | 265 | 0 | --- | K.SIGMIEEVPGMK.A + 2 Oxidation (M) | | 1402.5397 | 1401.5324 | 1401.7867 | -181.42 | 188 | - | 200 | 0 | --- | K.LFNAVAAEDLIVK.G | | 1402.5397 | 1401.5324 | 1401.7867 | -181.42 | 188 | - | 200 | 0 | 91 | K.LFNAVAAEDLIVK.G | | 1420.4296 | 1419.4223 | 1419.6664 | -171.90 | 266 | - | 278 | 0 | --- | K.ALDMNSAEDAIVR.L + Oxidation (M) | | 1487.4435 | 1486.4362 | 1486.6619 | -151.77 | 303 | - | 316 | 0 | --- | R.MGPTFGAMMISGQK.A + 2 Oxidation (M) | | 1503.3796 | 1502.3723 | 1502.6568 | -189.29 | 303 | - | 316 | 0 | --- | R.MGPTFGAMMISGQK.A + 3 Oxidation (M) | | 1503.3796 | 1502.3723 | 1502.6568 | -189.29 | 303 | - | 316 | 0 | 42 | R.MGPTFGAMMISGQK.A + 3 Oxidation (M) | | 1531.5128 | 1530.5055 | 1530.8149 | -202.13 | 252 | - | 265 | 1 | --- | R.LKSIGMIEEVPGMK.A | | 2201.6624 | 2200.6551 | 2201.0708 | -188.86 | 282 | - | 302 | 0 | --- | R.EVVPGMIVTGMEVAEIDGAPR.M + 2 Oxidation (M) | | 2201.6624 | 2200.6551 | 2201.0708 | -188.86 | 282 | - | 302 | 0 | 95 | R.EVVPGMIVTGMEVAEIDGAPR.M + 2 Oxidation (M) | | 2691.8328 | 2690.8255 | 2691.2917 | -173.23 | 254 | - | 278 | 1 | --- | K.SIGMIEEVPGMKALDMNSAEDAIVR.L + Oxidation (M) | | 2833.8765 | 2832.8692 | 2833.4174 | -193.47 | 144 | - | 167 | 0 | --- | R.KPAHIFLDELGIDYDEQDNYVVIK.H | |
| | No match to: 700.2686, 705.2428, 713.2744, 716.2278, 724.3425, 725.2244, 730.2465, 734.3532, 736.3567, 744.2535, 750.3661, 768.3801, 782.3060, 834.3594, 842.3480, 847.2695, 850.3714, 864.3561, 868.3801, 882.3931, 933.3290, 934.3179, 952.3059, 965.2977, 974.3585, 987.3167, 1017.4750, 1051.5004, 1059.3854, 1059.3854, 1151.4907, 1165.5140, 1179.3969, 1208.4299, 1225.4141, 1232.4075, 1252.4338, 1260.4773, 1293.4642, 1296.4609, 1315.4155, 1323.4485, 1331.3845, 1334.4703, 1334.6333, 1338.4514, 1339.4160, 1339.4160, 1356.4674, 1361.3998, 1365.4573, 1368.3833, 1410.4033, 1416.4844, 1419.4833, 1423.4431, 1426.3981, 1432.4216, 1434.4869, 1441.4395, 1448.6395, 1454.4496, 1519.3888, 1533.5110, 1535.4623, 1553.4756, 1678.4771, 1682.5519, 1682.5519, 1696.5294, 1773.5555, 1789.5026, 2002.6879, 2019.6295, 2022.6394, 2022.6394, 2073.5886, 2137.7241, 2137.7241, 2183.7175, 2184.6609, 2199.6689, 2217.6516, 2220.6860, 2224.6746, 2238.7246, 2238.7246, 2245.7859, 2336.7375, 2383.5217, 2612.8037, 2613.7783, 2666.7515, 2806.8625, 3346.0735 | | --- | | **S39** [**Cs6g03210.1**](http://zhangyang-pc/mascot/cgi/protein_view.pl?file=../data/20140118/F012104.dat&hit=1) **Mass: 17278 Score: 485 Expect: 1.4e-044 Matches: 17** | |
| | Observed | Mr(expt) | Mr(calc) | ppm | Start |  | End | Miss | Ions | Peptide | | --- | --- | --- | --- | --- | --- | --- | --- | --- | --- | | 878.3160 | 877.3087 | 877.4545 | -166.17 | 72 | - | 78 | 0 | --- | K.FIEIDNK.K | | 906.3330 | 905.3257 | 905.4719 | -161.40 | 148 | - | 154 | 0 | --- | K.NYLLNNR.D | | 906.3330 | 905.3257 | 905.4719 | -161.40 | 148 | - | 154 | 0 | 50 | K.NYLLNNR.D | | 990.4252 | 989.4179 | 989.5797 | -163.52 | 101 | - | 108 | 0 | --- | R.VIFEIIEK.G | | 990.4252 | 989.4179 | 989.5797 | -163.52 | 101 | - | 108 | 0 | 46 | R.VIFEIIEK.G | | 1006.3820 | 1005.3747 | 1005.5495 | -173.78 | 72 | - | 79 | 1 | --- | K.FIEIDNKK.R | | 1010.3506 | 1009.3433 | 1009.5080 | -163.12 | 117 | - | 124 | 0 | --- | K.STIEYELR.E | | 1010.3506 | 1009.3433 | 1009.5080 | -163.12 | 117 | - | 124 | 0 | 55 | K.STIEYELR.E | | 1021.3426 | 1020.3353 | 1020.4988 | -160.22 | 148 | - | 155 | 1 | --- | K.NYLLNNRD.- | | 1021.3426 | 1020.3353 | 1020.4988 | -160.22 | 148 | - | 155 | 1 | 21 | K.NYLLNNRD.- | | 1269.4570 | 1268.4497 | 1268.6554 | -162.09 | 58 | - | 69 | 0 | --- | K.FKPGTPGFAGYK.E | | 1269.4570 | 1268.4497 | 1268.6554 | -162.09 | 58 | - | 69 | 0 | 90 | K.FKPGTPGFAGYK.E | | 1722.5752 | 1721.5679 | 1721.8472 | -162.18 | 125 | - | 141 | 0 | --- | R.EEAAANASFVSIDTVAK.I | | 1722.5752 | 1721.5679 | 1721.8472 | -162.18 | 125 | - | 141 | 0 | 79 | R.EEAAANASFVSIDTVAK.I | | 1986.7169 | 1985.7096 | 1986.0099 | -151.19 | 83 | - | 100 | 0 | --- | R.VTDVVEGGYLDVGFTLFR.V | | 1986.7169 | 1985.7096 | 1986.0099 | -151.19 | 83 | - | 100 | 0 | 82 | R.VTDVVEGGYLDVGFTLFR.V | | 2600.8733 | 2599.8660 | 2600.2858 | -161.42 | 34 | - | 57 | 0 | --- | K.EYDTVEEIEVVEGDGGVGTILHIK.F | |
| | No match to: 700.2756, 706.2685, 709.2547, 713.2914, 715.2933, 716.2525, 722.3014, 724.3623, 727.2661, 734.3559, 739.2632, 744.2751, 768.3929, 788.2808, 839.3383, 842.3684, 846.3353, 850.3492, 860.9349, 864.3406, 868.3962, 882.4139, 888.2865, 951.3731, 965.3198, 982.4093, 993.3704, 996.3924, 1003.3138, 1017.4976, 1020.3417, 1024.3605, 1026.3591, 1031.3722, 1032.3317, 1033.3445, 1043.3439, 1045.3785, 1049.3816, 1051.5369, 1059.4053, 1059.4053, 1065.3661, 1074.3549, 1115.4636, 1126.3856, 1148.3384, 1151.4689, 1161.4072, 1164.4220, 1165.5157, 1173.4113, 1174.4457, 1174.4457, 1179.4153, 1202.4509, 1219.4796, 1230.5007, 1230.5007, 1235.3486, 1247.4375, 1251.4299, 1265.4681, 1268.4287, 1285.4033, 1291.4484, 1307.4233, 1315.4467, 1323.4537, 1331.4226, 1365.4260, 1383.4718, 1423.4562, 1475.5409, 1479.5253, 1491.5040, 1704.5948, 1721.5581, 1744.5596, 1791.4458, 1850.5903, 2002.7308, 2220.7488, 2238.7739, 2245.8367, 2359.7124, 2383.5830, 2457.8569, 2469.8552, 2642.9033, 2654.9197, 3311.7654, 3346.1013 | | --- | | **S14** [**Cs1g06360.1**](http://zhangyang-pc/mascot/cgi/protein_view.pl?file=../data/20140118/F012147.dat&hit=1) **Mass: 28915 Score: 71 Expect: 0.0034 Matches: 6** | |
| | Observed | Mr(expt) | Mr(calc) | ppm | Start |  | End | Miss | Ions | Peptide | | --- | --- | --- | --- | --- | --- | --- | --- | --- | --- | | 716.2478 | 715.2405 | 715.3977 | -219.65 | 216 | - | 221 | 1 | --- | K.EIKNGR.L | | 894.3495 | 893.3422 | 893.5083 | -185.87 | 97 | - | 104 | 0 | --- | R.ALEVIHGR.W | | 894.3495 | 893.3422 | 893.5083 | -185.87 | 97 | - | 104 | 0 | 56 | R.ALEVIHGR.W | | 1844.5363 | 1843.5290 | 1843.8563 | -177.50 | 43 | - | 57 | 1 | --- | K.YTMGNELWYGPDRVK.Y + Oxidation (M) | | 2025.6124 | 2024.6051 | 2025.0104 | -200.14 | 222 | - | 239 | 0 | --- | R.LAMFSMFGFFVQAIVTGK.G + 2 Oxidation (M) | | 3748.1230 | 3747.1157 | 3747.8308 | -190.81 | 178 | - | 213 | 0 | --- | R.INGLPGVGEGNDLYPGGQYFDPLGLADDPVTFAELK.V | |
| | No match to: 700.2457, 705.2469, 734.3644, 768.3505, 800.2568, 803.1861, 806.1686, 809.2382, 819.2499, 821.2634, 837.2357, 842.3377, 850.3768, 877.2618, 985.2961, 998.3514, 1176.3511, 1178.3638, 1204.3301, 1205.3384, 1207.3414, 1210.3412, 1210.3412, 1218.3136, 1220.3339, 1221.3179, 1222.3256, 1222.3256, 1234.3396, 1236.3346, 1238.3330, 1238.3330, 1243.2971, 1244.3140, 1250.3455, 1252.3374, 1254.3329, 1260.3042, 1264.3571, 1266.3376, 1267.3395, 1276.3187, 1278.3617, 1279.3553, 1279.3553, 1281.3679, 1292.3403, 1293.3735, 1295.3732, 1298.4529, 1305.3956, 1309.3785, 1310.4475, 1310.4475, 1318.3895, 1320.3303, 1322.3593, 1326.4193, 1332.4199, 1334.3824, 1338.3988, 1346.3873, 1348.2576, 1350.3641, 1363.4705, 1366.4167, 1368.4003, 1420.4281, 1474.4821, 1501.5885, 1515.5984, 1515.5984, 1721.6418, 1744.5792, 1769.6624, 1842.5186, 2111.6282, 2131.6658, 2131.6658, 2153.6216, 2171.6763, 2238.7544, 2681.8618, 2681.8618, 2691.8740, 2693.8770, 2709.8467, 2807.8171, 2810.7568, 2821.7456, 2821.7456, 2837.7791, 3347.0830, 3375.9104, 3380.8967, 3390.8899, 3392.8862, 3400.7659, 3403.9016, 3407.9104, 3408.8867, 3409.8865, 3422.8723, 3488.8262 | | --- | | **S20** [**Cs3g06180.1**](http://zhangyang-pc/mascot/cgi/protein_view.pl?file=../data/20140118/F012151.dat&hit=1) **Mass: 29500 Score: 167 Expect: 8.8e-013 Matches: 11** | |
| | Observed | Mr(expt) | Mr(calc) | ppm | Start |  | End | Miss | Ions | Peptide | | --- | --- | --- | --- | --- | --- | --- | --- | --- | --- | | 1031.3346 | 1030.3273 | 1030.5124 | -179.57 | 185 | - | 192 | 0 | --- | R.QYFLGFEK.Y | | 1105.3516 | 1104.3443 | 1104.5523 | -188.32 | 26 | - | 36 | 1 | --- | R.SAQSPTGSSRK.G | | 1105.3516 | 1104.3443 | 1104.5523 | -188.32 | 26 | - | 36 | 1 | --- | R.SAQSPTGSSRK.G | | 1277.4459 | 1276.4386 | 1276.6564 | -170.59 | 97 | - | 107 | 0 | --- | K.WLAYGEVINGR.Y | | 1277.4459 | 1276.4386 | 1276.6564 | -170.59 | 97 | - | 107 | 0 | 88 | K.WLAYGEVINGR.Y | | 1381.4027 | 1380.3954 | 1380.5881 | -139.55 | 173 | - | 184 | 0 | --- | R.FQDWANPGSMGR.Q + Oxidation (M) | | 1689.6149 | 1688.6076 | 1688.9171 | -183.24 | 108 | - | 124 | 0 | --- | R.YAMLGAVGAIAPEILGK.A + Oxidation (M) | | 2246.7302 | 2245.7229 | 2246.1372 | -184.43 | 193 | - | 215 | 0 | --- | K.YLGGSGDPAYPGGPLFNPLGLGK.D | | 2246.7302 | 2245.7229 | 2246.1372 | -184.43 | 193 | - | 215 | 0 | 50 | K.YLGGSGDPAYPGGPLFNPLGLGK.D | | 2618.8335 | 2617.8262 | 2618.3017 | -181.58 | 193 | - | 218 | 1 | --- | K.YLGGSGDPAYPGGPLFNPLGLGKDEK.S | | 3529.0093 | 3528.0020 | 3527.6620 | 96.4 | 63 | - | 96 | 0 | --- | K.QSLSYLDGSLPGDYGFDPLGLSDPEGTGGFIEPK.W | |
| | No match to: 741.2674, 842.3492, 1014.3109, 1072.3409, 1076.3344, 1087.3401, 1088.3293, 1088.3293, 1091.3557, 1093.3590, 1104.3269, 1121.3497, 1131.3810, 1145.3793, 1146.3940, 1147.3990, 1258.4099, 1260.4252, 1262.4130, 1263.4147, 1264.4235, 1272.4044, 1275.4297, 1276.4218, 1281.4403, 1285.4342, 1287.4315, 1289.4470, 1290.4397, 1291.4287, 1292.4196, 1293.4359, 1293.4359, 1299.4250, 1309.4362, 1309.4362, 1321.4000, 1327.4243, 1328.4211, 1331.4160, 1332.4447, 1333.3938, 1349.4086, 1358.4614, 1361.4631, 1362.4509, 1362.4509, 1372.4288, 1374.4509, 1379.4562, 1379.4562, 1385.3606, 1388.4054, 1389.4413, 1390.4484, 1391.4655, 1395.3900, 1397.3531, 1397.3531, 1401.3785, 1405.4539, 1407.4615, 1413.3544, 1413.3544, 1419.4281, 1429.4133, 1430.4896, 1431.5016, 1486.5854, 1503.4928, 1505.4714, 1509.4578, 1514.4702, 1527.4510, 1535.4825, 1543.4712, 1545.5031, 1547.4939, 1564.4943, 1567.4249, 1574.4701, 1584.5131, 1589.5314, 1593.5336, 1597.5270, 1603.5090, 1607.5055, 1711.6149, 1832.6543, 1896.6057, 1975.7404, 2268.7407, 2284.7085, 2342.6833, 2640.8330, 3511.0012, 3512.0024, 3526.0283, 3549.9753 | | --- | | **S16** [**Cs1g23450.1**](http://zhangyang-pc/mascot/cgi/protein_view.pl?file=../data/20140118/F012117.dat&hit=1) **Mass: 35355 Score: 371 Expect: 3.5e-033 Matches:14** | |
| | Observed | Mr(expt) | Mr(calc) | ppm | Start |  | End | Miss | Ions | Peptide | | --- | --- | --- | --- | --- | --- | --- | --- | --- | --- | | 964.3889 | 963.3816 | 963.5793 | -205.25 | 210 | - | 217 | 0 | --- | R.VPFLFTIK.Q | | 1063.4330 | 1062.4257 | 1062.5458 | -113.01 | 239 | - | 248 | 1 | --- | R.GSSFLDPKGR.G | | 1131.3689 | 1130.3616 | 1130.5356 | -153.91 | 154 | - | 163 | 0 | --- | K.NAPPDFQNTK.L | | 1252.4546 | 1251.4473 | 1251.6459 | -158.64 | 92 | - | 101 | 1 | --- | K.RLTYDEIQSK.T | | 1252.4546 | 1251.4473 | 1251.6459 | -158.64 | 92 | - | 101 | 1 | 33 | K.RLTYDEIQSK.T | | 1576.5237 | 1575.5164 | 1575.7641 | -157.20 | 249 | - | 265 | 0 | --- | R.GGSTGYDNAIALPAGGR.G | | 1576.5237 | 1575.5164 | 1575.7641 | -157.20 | 249 | - | 265 | 0 | 110 | R.GGSTGYDNAIALPAGGR.G | | 1760.6007 | 1759.5934 | 1759.8741 | -159.48 | 193 | - | 209 | 0 | --- | K.DGIDYAAVTVQLPGGER.V | | 1760.6007 | 1759.5934 | 1759.8741 | -159.48 | 193 | - | 209 | 0 | 101 | K.DGIDYAAVTVQLPGGER.V | | 2294.7971 | 2293.7898 | 2294.1695 | -165.51 | 218 | - | 238 | 0 | --- | K.QLVASGKPENFGGEFLVPSYR.G | | 2294.7971 | 2293.7898 | 2294.1695 | -165.51 | 218 | - | 238 | 0 | 92 | K.QLVASGKPENFGGEFLVPSYR.G | | 2323.7532 | 2322.7459 | 2323.1471 | -172.70 | 168 | - | 188 | 0 | --- | R.LTYTLDEIEGPFEVSPDGTIK.F | | 2433.7468 | 2432.7395 | 2433.1408 | -164.92 | 249 | - | 273 | 1 | --- | R.GGSTGYDNAIALPAGGRGDEEDLAK.E | | 2637.7993 | 2636.7920 | 2637.2480 | -172.89 | 293 | - | 317 | 0 | --- | K.SKPETGEVIGVFESLQPSDTDMGAK.V + Oxidation (M) | |
| | No match to: 705.2618, 713.2710, 768.3590, 809.2652, 842.3581, 944.3942, 945.3707, 986.3723, 987.3265, 987.3265, 996.3467, 998.3413, 999.3217, 1009.3125, 1013.3197, 1014.3677, 1015.3320, 1021.3049, 1025.2905, 1037.3113, 1055.3574, 1062.4124, 1067.3712, 1071.3402, 1072.3763, 1094.4154, 1130.3932, 1163.3624, 1170.4918, 1214.4855, 1241.3787, 1267.5260, 1275.4559, 1279.4725, 1292.4707, 1320.4600, 1337.4725, 1371.4828, 1384.6130, 1398.4084, 1398.4084, 1406.3770, 1408.4063, 1410.4028, 1410.4028, 1424.4005, 1426.3999, 1426.3999, 1448.4056, 1458.5007, 1472.6121, 1477.5179, 1520.3867, 1541.4839, 1557.4971, 1611.5283, 1625.5387, 1635.6449, 1645.4808, 1707.6390, 1715.5778, 1725.6289, 1734.5923, 1734.5923, 1796.6190, 1805.6373, 1831.6852, 1832.6818, 1844.6750, 1860.6243, 1881.6921, 1927.6243, 1939.6208, 1951.6235, 1955.6487, 2015.6798, 2075.6318, 2097.6328, 2113.5928, 2158.6799, 2237.7639, 2276.8164, 2277.7783, 2277.7783, 2291.7686, 2300.7473, 2309.7061, 2316.7522, 2320.7227, 2345.7122, 2357.7488, 2440.7031, 2603.8359, 2738.9648, 3123.9607, 3377.8970 | | --- | | **S19** [**Cs1g23450.1**](http://zhangyang-pc/mascot/cgi/protein_view.pl?file=../data/20140118/F012111.dat&hit=1)**Mass: 35355    Score: 523    Expect: 2.2e-048  Matches: 19** | |
| | Observed | | | | Mr(expt) | | | | | | | | | | Mr(calc) | | | | | | | | | | | ppm | | | | | | | | | | | Start | | | | | | | | | | |  | | | | | | | | | | | End | | | | | | | | | | | Miss | | | | | | | | | | | Ions | | | | | | | | | | | Peptide | | | | | | | | | | | | |  | | | --- | --- | --- | --- | --- | --- | --- | --- | --- | --- | --- | --- | --- | --- | --- | --- | --- | --- | --- | --- | --- | --- | --- | --- | --- | --- | --- | --- | --- | --- | --- | --- | --- | --- | --- | --- | --- | --- | --- | --- | --- | --- | --- | --- | --- | --- | --- | --- | --- | --- | --- | --- | --- | --- | --- | --- | --- | --- | --- | --- | --- | --- | --- | --- | --- | --- | --- | --- | --- | --- | --- | --- | --- | --- | --- | --- | --- | --- | --- | --- | --- | --- | --- | --- | --- | --- | --- | --- | --- | --- | --- | --- | --- | --- | --- | --- | --- | --- | --- | --- | --- | --- | --- | --- | --- | --- | | 964.4048 | | | | 963.3975 | | | | | | | | | | 963.5793 | | | | | | | | | | | -188.70 | | | | | | | | | | | 210 | | | | | | | | | | | - | | | | | | | | | | | 217 | | | | | | | | | | | 0 | | | | | | | | | | | --- | | | | | | | | | | | R.VPFLFTIK.Q | | | | | | | | | | | | |  | | | 1096.3655 | | | | 1095.3582 | | | | | | | | | | 1095.5448 | | | | | | | | | | | -170.29 | | | | | | | | | | | 93 | | | | | | | | | | | - | | | | | | | | | | | 101 | | | | | | | | | | | 0 | | | | | | | | | | | --- | | | | | | | | | | | R.LTYDEIQSK.T | | | | | | | | | | | | |  | | | 1131.3374 | | | | 1130.3301 | | | | | | | | | | 1130.5356 | | | | | | | | | | | -181.78 | | | | | | | | | | | 154 | | | | | | | | | | | - | | | | | | | | | | | 163 | | | | | | | | | | | 0 | | | | | | | | | | | --- | | | | | | | | | | | K.NAPPDFQNTK.L | | | | | | | | | | | | |  | | | 1252.4329 | | | | 1251.4256 | | | | | | | | | | 1251.6459 | | | | | | | | | | | -175.98 | | | | | | | | | | | 92 | | | | | | | | | | | - | | | | | | | | | | | 101 | | | | | | | | | | | 1 | | | | | | | | | | | --- | | | | | | | | | | | K.RLTYDEIQSK.T | | | | | | | | | | | | |  | | | 1252.4329 | | | | 1251.4256 | | | | | | | | | | 1251.6459 | | | | | | | | | | | -175.98 | | | | | | | | | | | 92 | | | | | | | | | | | - | | | | | | | | | | | 101 | | | | | | | | | | | 1 | | | | | | | | | | | 68 | | | | | | | | | | | K.RLTYDEIQSK.T | | | | | | | | | | | | |  | | | 1271.4331 | | | | 1270.4258 | | | | | | | | | | 1270.6268 | | | | | | | | | | | -158.15 | | | | | | | | | | | 137 | | | | | | | | | | | - | | | | | | | | | | | 147 | | | | | | | | | | | 0 | | | | | | | | | | | --- | | | | | | | | | | | .FCLEPTSFTVK.A | | | | | | | | | | | | |  | | | 1576.4977 | | | | 1575.4904 | | | | | | | | | | 1575.7641 | | | | | | | | | | | -173.70 | | | | | | | | | | | 249 | | | | | | | | | | | - | | | | | | | | | | | 265 | | | | | | | | | | | 0 | | | | | | | | | | | --- | | | | | | | | | | | R.GGSTGYDNAIALPAGGR.G | | | | | | | | | | | | |  | | | 1576.4977 | | | | 1575.4904 | | | | | | | | | | 1575.7641 | | | | | | | | | | | -173.70 | | | | | | | | | | | 249 | | | | | | | | | | | - | | | | | | | | | | | 265 | | | | | | | | | | | 0 | | | | | | | | | | | 135 | | | | | | | | | | | R.GGSTGYDNAIALPAGGR.G | | | | | | | | | | | | |  | | | 1760.5676 | | | | 1759.5603 | | | | | | | | | | 1759.8741 | | | | | | | | | | | -178.29 | | | | | | | | | | | 193 | | | | | | | | | | | - | | | | | | | | | | | 209 | | | | | | | | | | | 0 | | | | | | | | | | | --- | | | | | | | | | | | K.DGIDYAAVTVQLPGGER.V | | | | | | | | | | | | |  | | | 1760.5676 | | | | 1759.5603 | | | | | | | | | | 1759.8741 | | | | | | | | | | | -178.29 | | | | | | | | | | | 193 | | | | | | | | | | | - | | | | | | | | | | | 209 | | | | | | | | | | | 0 | | | | | | | | | | | 119 | | | | | | | | | | | K.DGIDYAAVTVQLPGGER.V | | | | | | | | | | | | |  | | | 2263.8020 | | | | 2262.7947 | | | | | | | | | | 2263.1485 | | | | | | | | | | | -156.31 | | | | | | | | | | | 189 | | | | | | | | | | | - | | | | | | | | | | | 209 | | | | | | | | | | | 1 | | | | | | | | | | | --- | | | | | | | | | | | K.FVEKDGIDYAAVTVQLPGGER.V | | | | | | | | | | | | |  | | | 2264.7791 | | | | 2263.7718 | | | | | | | | | | 2263.1485 | | | | | | | | | | | 275 | | | | | | | | | | | 189 | | | | | | | | | | | - | | | | | | | | | | | 209 | | | | | | | | | | | 1 | | | | | | | | | | | --- | | | | | | | | | | | K.FVEKDGIDYAAVTVQLPGGER.V | | | | | | | | | | | | |  | | | 2294.7639 | | | | 2293.7566 | | | | | | | | | | 2294.1695 | | | | | | | | | | | -179.98 | | | | | | | | | | | 218 | | | | | | | | | | | - | | | | | | | | | | | 238 | | | | | | | | | | | 0 | | | | | | | | | | | --- | | | | | | | | | | | K.QLVASGKPENFGGEFLVPSYR.G | | | | | | | | | | | | |  | | | 2294.7639 | | | | 2293.7566 | | | | | | | | | | 2294.1695 | | | | | | | | | | | -179.98 | | | | | | | | | | | 218 | | | | | | | | | | | - | | | | | | | | | | | 238 | | | | | | | | | | | 0 | | | | | | | | | | | 99 | | | | | | | | | | | K.QLVASGKPENFGGEFLVPSYR.G | | | | | | | | | | | | |  | | | 2323.7346 | | | | 2322.7273 | | | | | | | | | | 2323.1471 | | | | | | | | | | | -180.70 | | | | | | | | | | | 168 | | | | | | | | | | | - | | | | | | | | | | | 188 | | | | | | | | | | | 0 | | | | | | | | | | | --- | | | | | | | | | | | R.LTYTLDEIEGPFEVSPDGTIK.F | | | | | | | | | | | | |  | | | 2323.7346 | | | | 2322.7273 | | | | | | | | | | 2323.1471 | | | | | | | | | | | -180.70 | | | | | | | | | | | 168 | | | | | | | | | | | - | | | | | | | | | | | 188 | | | | | | | | | | | 0 | | | | | | | | | | | 30 | | | | | | | | | | | R.LTYTLDEIEGPFEVSPDGTIK.F | | | | | | | | | | | | |  | | | 2433.7263 | | | | 2432.7190 | | | | | | | | | | 2433.1408 | | | | | | | | | | | -173.34 | | | | | | | | | | | 249 | | | | | | | | | | | - | | | | | | | | | | | 273 | | | | | | | | | | | 1 | | | | | | | | | | | --- | | | | | | | | | | | R.GGSTGYDNAIALPAGGRGDEEDLAK.E | | | | | | | | | | | | |  | | | 2433.7263 | | | | 2432.7190 | | | | | | | | | | 2433.1408 | | | | | | | | | | | -173.34 | | | | | | | | | | | 249 | | | | | | | | | | | - | | | | | | | | | | | 273 | | | | | | | | | | | 1 | | | | | | | | | | | 24 | | | | | | | | | | | R.GGSTGYDNAIALPAGGRGDEEDLAK.E | | | | | | | | | | | | |  | | | 2637.7871 | | | | 2636.7798 | | | | | | | | | | 2637.2480 | | | | | | | | | | | -177.52 | | | | | | | | | | | 293 | | | | | | | | | | | - | | | | | | | | | | | 317 | | | | | | | | | | | 0 | | | | | | | | | | | --- | | | | | | | | | | | K.SKPETGEVIGVFESLQPSDTDMGAK.V + Oxidation (M) | | | | | | | | | | | | |  | | | No match to: 842.3530, 892.8465, 933.3404, 952.3201, 1002.3567, 1059.3817, 1165.3335, 1237.4188, 1263.4279, 1296.4751, 1365.4973, 1374.4310, 1385.4568, 1390.4132, 1422.4933, 1461.4318, 1496.4766, 1528.4978, 1529.4946, 1598.4994, 1611.5118, 1611.5118, 1615.5087, 1623.5225, 1625.5242, 1637.5228, 1645.5923, 1689.5511, 1695.5392, 1715.5798, 1716.5872, 1734.5585, 1734.5585, 1739.5846, 1742.5826, 1748.5762, 1769.6091, 1773.5416, 1796.6033, 1818.5594, 1833.5718, 1833.5718, 1849.5775, 1927.5947, 1946.6183, 1981.5872, 2074.7363, 2166.7759, 2237.7637, 2238.7673, 2246.8101, 2259.7905, 2262.7742, 2275.7759, 2276.8032, 2277.7461, 2277.7461, 2287.8086, 2291.7754, 2293.7764, 2299.7842, 2302.7581, 2306.7632, 2309.8049, 2315.7070, 2317.7368, 2321.7324, 2322.7400, 2332.7341, 2337.7358, 2345.7227, 2360.7144, 2361.6953, 2365.7029, 2399.6875, 2403.6765, 2415.7136, 2422.7412, 2439.7600, 2440.6943, 2448.7754, 2462.6819, 2478.6489, 2482.7866, 2573.8042, 2603.8420, 2625.8435, 2641.7844, 2653.7749, 2677.7825, 2917.9185 | | | | | | | | | | | | | | | | | | | | | | | | | | | | | | | | | | | | | | | | | | | | | | | | | | | | | | | | | | | | | | | | | | | | | | | | | | | | | | | | | | | | | | | | | | | | | | | | | | | |  | | | | | | | **S18** [**Cs7g31800.4**](http://zhangyang-pc/mascot/cgi/protein_view.pl?file=../data/20140118/F012122.dat&hit=2) **Mass: 50870 Score: 534 Expect: 1.8e-049 Matches: 23** | | | | | | | | | | | | | | | | | | | | | | | | | | | | | | | | | | | | | | | | | | | | | | | | | | | | | | | | | | | | | | | | | | | | | | | | | | | | | | | | | | | | | | | | | | | | | | | | | | | |  | | | | | | | Observed | Mr(expt) | | | | | | | | | | Mr(calc) | | | | | | | | | | | ppm | | | | | | | | | | | Start | | | | | | | | | | |  | | | | | End | | | | | | | | | | | | | | | | | Miss | | | | | | | | | | | Ions | | | | | | | | | | | Peptide | | | | | | | | | | | | | | | |  | | | 895.2548 | 894.2475 | | | | | | | | | | 894.4083 | | | | | | | | | | | -179.75 | | | | | | | | | | | 343 | | | | | | | | | | | - | | | | | 349 | | | | | | | | | | | | | | | | | 0 | | | | | | | | | | | --- | | | | | | | | | | | R.VYDDEVR.K | | | | | | | | | | | | | | | |  | | | 895.2548 | 894.2475 | | | | | | | | | | 894.4083 | | | | | | | | | | | -179.75 | | | | | | | | | | | 343 | | | | | | | | | | | - | | | | | 349 | | | | | | | | | | | | | | | | | 0 | | | | | | | | | | | 61 | | | | | | | | | | | R.VYDDEVR.K | | | | | | | | | | | | | | | |  | | | 1023.3326 | 1022.3253 | | | | | | | | | | 1022.5033 | | | | | | | | | | | -174.02 | | | | | | | | | | | 343 | | | | | | | | | | | - | | | | | 350 | | | | | | | | | | | | | | | | | 1 | | | | | | | | | | | --- | | | | | | | | | | | R.VYDDEVRK.W | | | | | | | | | | | | | | | |  | | | 1079.3596 | 1078.3523 | | | | | | | | | | 1078.5481 | | | | | | | | | | | -181.51 | | | | | | | | | | | 139 | | | | | | | | | | | - | | | | | 147 | | | | | | | | | | | | | | | | | 0 | | | | | | | | | | | --- | | | | | | | | | | | K.NFMSLPNIK.V + Oxidation (M) | | | | | | | | | | | | | | | |  | | | 1145.3655 | 1144.3582 | | | | | | | | | | 1144.5513 | | | | | | | | | | | -168.67 | | | | | | | | | | | 369 | | | | | | | | | | | - | | | | | 378 | | | | | | | | | | | | | | | | | 0 | | | | | | | | | | | --- | | | | | | | | | | | K.EAAPTFEQPR.M | | | | | | | | | | | | | | | |  | | | 1145.3655 | 1144.3582 | | | | | | | | | | 1144.5513 | | | | | | | | | | | -168.67 | | | | | | | | | | | 369 | | | | | | | | | | | - | | | | | 378 | | | | | | | | | | | | | | | | | 0 | | | | | | | | | | | 72 | | | | | | | | | | | K.EAAPTFEQPR.M | | | | | | | | | | | | | | | |  | | | 1159.3601 | 1158.3528 | | | | | | | | | | 1158.6397 | | | | | | | | | | | -247.62 | | | | | | | | | | | 351 | | | | | | | | | | | - | | | | | 362 | | | | | | | | | | | | | | | | | 0 | | | | | | | | | | | --- | | | | | | | | | | | K.WISGVGVGSIGK.S | | | | | | | | | | | | | | | |  | | | 1725.4690 | 1724.4617 | | | | | | | | | | 1724.7489 | | | | | | | | | | | -166.52 | | | | | | | | | | | 64 | | | | | | | | | | | - | | | | | 78 | | | | | | | | | | | | | | | | | 0 | | | | | | | | | | | --- | | | | | | | | | | | K.GLAYDESDDQQDITR.G | | | | | | | | | | | | | | | |  | | | 1725.4690 | 1724.4617 | | | | | | | | | | 1724.7489 | | | | | | | | | | | -166.52 | | | | | | | | | | | 64 | | | | | | | | | | | - | | | | | 78 | | | | | | | | | | | | | | | | | 0 | | | | | | | | | | | 91 | | | | | | | | | | | K.GLAYDESDDQQDITR.G | | | | | | | | | | | | | | | |  | | | 1882.6561 | 1881.6488 | | | | | | | | | | 1881.9625 | | | | | | | | | | | -166.69 | | | | | | | | | | | 324 | | | | | | | | | | | - | | | | | 340 | | | | | | | | | | | | | | | | | 0 | | | | | | | | | | | --- | | | | | | | | | | | K.LVDTFPGQSIDFFGALR.A | | | | | | | | | | | | | | | |  | | | 1882.6561 | 1881.6488 | | | | | | | | | | 1881.9625 | | | | | | | | | | | -166.69 | | | | | | | | | | | 324 | | | | | | | | | | | - | | | | | 340 | | | | | | | | | | | | | | | | | 0 | | | | | | | | | | | 82 | | | | | | | | | | | K.LVDTFPGQSIDFFGALR.A | | | | | | | | | | | | | | | |  | | | 1922.5824 | 1921.5751 | | | | | | | | | | 1921.9455 | | | | | | | | | | | -192.71 | | | | | | | | | | | 384 | | | | | | | | | | | - | | | | | 399 | | | | | | | | | | | | | | | | | 0 | | | | | | | | | | | --- | | | | | | | | | | | K.LLEYGNMIVQEQENVK.R + Oxidation (M) | | | | | | | | | | | | | | | |  | | | 1922.5824 | 1921.5751 | | | | | | | | | | 1921.9455 | | | | | | | | | | | -192.71 | | | | | | | | | | | 384 | | | | | | | | | | | - | | | | | 399 | | | | | | | | | | | | | | | | | 0 | | | | | | | | | | | 36 | | | | | | | | | | | K.LLEYGNMIVQEQENVK.R + Oxidation (M) | | | | | | | | | | | | | | | |  | | | 2063.6689 | 2062.6616 | | | | | | | | | | 2062.0517 | | | | | | | | | | | 296 | | | | | | | | | | | 384 | | | | | | | | | | | - | | | | | 400 | | | | | | | | | | | | | | | | | 1 | | | | | | | | | | | --- | | | | | | | | | | | K.LLEYGNMIVQEQENVKR.V | | | | | | | | | | | | | | | |  | | | 2078.6794 | 2077.6721 | | | | | | | | | | 2078.0466 | | | | | | | | | | | -180.21 | | | | | | | | | | | 384 | | | | | | | | | | | - | | | | | 400 | | | | | | | | | | | | | | | | | 1 | | | | | | | | | | | --- | | | | | | | | | | | K.LLEYGNMIVQEQENVKR.V + Oxidation (M) | | | | | | | | | | | | | | | |  | | | 2088.8159 | 2087.8086 | | | | | | | | | | 2088.1619 | | | | | | | | | | | -169.19 | | | | | | | | | | | 269 | | | | | | | | | | | - | | | | | 287 | | | | | | | | | | | | | | | | | 0 | | | | | | | | | | | --- | | | | | | | | | | | R.VPIIVTGNDFSTLYAPLIR.D | | | | | | | | | | | | | | | |  | | | 2088.8159 | 2087.8086 | | | | | | | | | | 2088.1619 | | | | | | | | | | | -169.19 | | | | | | | | | | | 269 | | | | | | | | | | | - | | | | | 287 | | | | | | | | | | | | | | | | | 0 | | | | | | | | | | | 91 | | | | | | | | | | | R.VPIIVTGNDFSTLYAPLIR.D | | | | | | | | | | | | | | | |  | | | 2324.5942 | 2323.5869 | | | | | | | | | | 2324.0341 | | | | | | | | | | | -192.40 | | | | | | | | | | | 112 | | | | | | | | | | | - | | | | | 131 | | | | | | | | | | | | | | | | | 0 | | | | | | | | | | | --- | | | | | | | | | | | R.TYNLDNTMDGLYIAPAFMDK.L + 2 Oxidation (M) | | | | | | | | | | | | | | | |  | | | 2351.5920 | 2350.5847 | | | | | | | | | | 2351.0443 | | | | | | | | | | | -195.48 | | | | | | | | | | | 173 | | | | | | | | | | | - | | | | | 195 | | | | | | | | | | | | | | | | | 0 | | | | | | | | | | | --- | | | | | | | | | | | K.MGINPIMMSAGELESGNAGEPAK.L + 3 Oxidation (M) | | | | | | | | | | | | | | | |  | | | 2446.6716 | 2445.6643 | | | | | | | | | | 2446.1288 | | | | | | | | | | | -189.88 | | | | | | | | | | | 407 | | | | | | | | | | | - | | | | | 429 | | | | | | | | | | | | | | | | | 0 | | | | | | | | | | | --- | | | | | | | | | | | K.YLSEAALGEANEDAIQSGNFYGK.A | | | | | | | | | | | | | | | |  | | | 2446.6716 | 2445.6643 | | | | | | | | | | 2446.1288 | | | | | | | | | | | -189.88 | | | | | | | | | | | 407 | | | | | | | | | | | - | | | | | 429 | | | | | | | | | | | | | | | | | 0 | | | | | | | | | | | 63 | | | | | | | | | | | K.YLSEAALGEANEDAIQSGNFYGK.A | | | | | | | | | | | | | | | |  | | | 2774.7153 | 2773.7080 | | | | | | | | | | 2774.2276 | | | | | | | | | | | -187.29 | | | | | | | | | | | 430 | | | | | | | | | | | - | | | | | 455 | | | | | | | | | | | | | | | | | 0 | | | | | | | | | | | --- | | | | | | | | | | | K.AAQQMNVPVPEGCTDPTAENFDPTAR.S + Oxidation (M) | | | | | | | | | | | | | | | |  | | | 3353.9224 | 3352.9151 | | | | | | | | | | 3353.5333 | | | | | | | | | | | -184.33 | | | | | | | | | | | 81 | | | | | | | | | | | - | | | | | 111 | | | | | | | | | | | | | | | | | 0 | | | | | | | | | | | --- | | | | | | | | | | | K.GAVDSLFQAPMGTGTHYAVMSSYDYISQGLR.T + 2 Oxidation (M) | | | | | | | | | | | | | | | |  | | | No match to: 724.2095, 768.3928, 810.2896, 842.3413, 860.9056, 941.2942, 942.2806, 943.2766, 944.2970, 944.2970, 953.2961, 954.2931, 955.2825, 956.2929, 956.2929, 966.2666, 968.3138, 970.2726, 972.2906, 972.2906, 978.2782, 982.3036, 995.2929, 996.3051, 997.2949, 1026.3203, 1040.3136, 1059.3834, 1082.3110, 1089.3025, 1101.3403, 1110.3398, 1112.3064, 1120.3250, 1125.3297, 1127.3571, 1129.3474, 1137.3892, 1144.3309, 1153.4113, 1156.4771, 1164.3895, 1167.3417, 1168.5063, 1169.4630, 1174.3644, 1175.4094, 1190.4071, 1218.3805, 1228.3912, 1244.3472, 1251.3870, 1288.3914, 1315.4497, 1331.4229, 1332.5704, 1338.4948, 1358.4023, 1370.3894, 1399.4652, 1410.4493, 1605.4763, 1707.4894, 1724.4718, 1739.4530, 1747.4243, 1769.5273, 1838.6858, 1858.5709, 1880.6576, 1896.5889, 1898.6130, 1904.6216, 1938.6018, 1942.5729, 1944.5789, 2014.6985, 2070.7905, 2094.6912, 2221.6814, 2468.7036, 3290.0623, 3314.8333, 3369.9707, 3795.9717, 3859.8809, 3874.9028 | | | | | | | | | | | | | | | | | | | | | | | | | | | | | | | | | | | | | | | | | | | | | | | | | | | | | | | | | | | | | | | | | | | | | | | | | | | | | | | | | | | | | | | | | | | | | | | | | | | |  | | | | | | | **S21** [**Cs7g31800.3**](http://zhangyang-pc/mascot/cgi/protein_view.pl?file=../data/20140118/F012192.dat&hit=1) **Mass: 46932 Score: 511 Expect: 3.5e-047 Matches: 20** | | | | | | | | | | | | | | | | | | | | | | | | | | | | | | | | | | | | | | | | | | | | | | | | | | | | | | | | | | | | | | | | | | | | | | | | | | | | | | | | | | | | | | | | | | | | | | | | | | | |  | | | | | | | Observed | Mr(expt) | | | | | | | | | | Mr(calc) | | | | | | | | | | | ppm | | | | | | | | | | | Start | | | | | | | | | | |  | | | | | End | | | | | | | | | | | | | | | | | Miss | | | | | | | | | | | Ions | | | | | | | | | | | Peptide | | | | | | | | | | | | | | | |  | | | 895.2437 | 894.2364 | | | | | | | | | | 894.4083 | | | | | | | | | | | -192.17 | | | | | | | | | | | 343 | | | | | | | | | | | - | | | | | 349 | | | | | | | | | | | | | | | | | 0 | | | | | | | | | | | --- | | | | | | | | | | | R.VYDDEVR.K | | | | | | | | | | | | | | | |  | | | 895.2437 | 894.2364 | | | | | | | | | | 894.4083 | | | | | | | | | | | -192.17 | | | | | | | | | | | 343 | | | | | | | | | | | - | | | | | 349 | | | | | | | | | | | | | | | | | 0 | | | | | | | | | | | 50 | | | | | | | | | | | R.VYDDEVR.K | | | | | | | | | | | | | | | |  | | | 1079.3550 | 1078.3477 | | | | | | | | | | 1078.5481 | | | | | | | | | | | -185.78 | | | | | | | | | | | 139 | | | | | | | | | | | - | | | | | 147 | | | | | | | | | | | | | | | | | 0 | | | | | | | | | | | --- | | | | | | | | | | | K.NFMSLPNIK.V + Oxidation (M) | | | | | | | | | | | | | | | |  | | | 1122.3632 | 1121.3559 | | | | | | | | | | 1121.5465 | | | | | | | | | | | -169.94 | | | | | | | | | | | 341 | | | | | | | | | | | - | | | | | 349 | | | | | | | | | | | | | | | | | 1 | | | | | | | | | | | --- | | | | | | | | | | | R.ARVYDDEVR.K | | | | | | | | | | | | | | | |  | | | 1145.3485 | 1144.3412 | | | | | | | | | | 1144.5513 | | | | | | | | | | | -183.52 | | | | | | | | | | | 369 | | | | | | | | | | | - | | | | | 378 | | | | | | | | | | | | | | | | | 0 | | | | | | | | | | | --- | | | | | | | | | | | K.EAAPTFEQPR.M | | | | | | | | | | | | | | | |  | | | 1145.3485 | 1144.3412 | | | | | | | | | | 1144.5513 | | | | | | | | | | | -183.52 | | | | | | | | | | | 369 | | | | | | | | | | | - | | | | | 378 | | | | | | | | | | | | | | | | | 0 | | | | | | | | | | | 66 | | | | | | | | | | | K.EAAPTFEQPR.M | | | | | | | | | | | | | | | |  | | | 1159.3704 | 1158.3631 | | | | | | | | | | 1158.6397 | | | | | | | | | | | -238.73 | | | | | | | | | | | 351 | | | | | | | | | | | - | | | | | 362 | | | | | | | | | | | | | | | | | 0 | | | | | | | | | | | --- | | | | | | | | | | | K.WISGVGVGSIGK.S | | | | | | | | | | | | | | | |  | | | 1725.4475 | 1724.4402 | | | | | | | | | | 1724.7489 | | | | | | | | | | | -178.99 | | | | | | | | | | | 64 | | | | | | | | | | | - | | | | | 78 | | | | | | | | | | | | | | | | | 0 | | | | | | | | | | | --- | | | | | | | | | | | K.GLAYDESDDQQDITR.G | | | | | | | | | | | | | | | |  | | | 1725.4475 | 1724.4402 | | | | | | | | | | 1724.7489 | | | | | | | | | | | -178.99 | | | | | | | | | | | 64 | | | | | | | | | | | - | | | | | 78 | | | | | | | | | | | | | | | | | 0 | | | | | | | | | | | 109 | | | | | | | | | | | K.GLAYDESDDQQDITR.G | | | | | | | | | | | | | | | |  | | | 1767.5029 | 1766.4956 | | | | | | | | | | 1766.7936 | | | | | | | | | | | -168.64 | | | | | | | | | | | 211 | | | | | | | | | | | - | | | | | 227 | | | | | | | | | | | | | | | | | 1 | | | | | | | | | | | --- | | | | | | | | | | | K.GKMCCLMINDLDAGAGR.M | | | | | | | | | | | | | | | |  | | | 1882.6317 | 1881.6244 | | | | | | | | | | 1881.9625 | | | | | | | | | | | -179.65 | | | | | | | | | | | 324 | | | | | | | | | | | - | | | | | 340 | | | | | | | | | | | | | | | | | 0 | | | | | | | | | | | --- | | | | | | | | | | | K.LVDTFPGQSIDFFGALR.A | | | | | | | | | | | | | | | |  | | | 1882.6317 | 1881.6244 | | | | | | | | | | 1881.9625 | | | | | | | | | | | -179.65 | | | | | | | | | | | 324 | | | | | | | | | | | - | | | | | 340 | | | | | | | | | | | | | | | | | 0 | | | | | | | | | | | 88 | | | | | | | | | | | K.LVDTFPGQSIDFFGALR.A | | | | | | | | | | | | | | | |  | | | 1922.5883 | 1921.5810 | | | | | | | | | | 1921.9455 | | | | | | | | | | | -189.64 | | | | | | | | | | | 384 | | | | | | | | | | | - | | | | | 399 | | | | | | | | | | | | | | | | | 0 | | | | | | | | | | | --- | | | | | | | | | | | K.LLEYGNMIVQEQENVK.R + Oxidation (M) | | | | | | | | | | | | | | | |  | | | 1922.5883 | 1921.5810 | | | | | | | | | | 1921.9455 | | | | | | | | | | | -189.64 | | | | | | | | | | | 384 | | | | | | | | | | | - | | | | | 399 | | | | | | | | | | | | | | | | | 0 | | | | | | | | | | | 68 | | | | | | | | | | | K.LLEYGNMIVQEQENVK.R + Oxidation (M) | | | | | | | | | | | | | | | |  | | | 2078.6738 | 2077.6665 | | | | | | | | | | 2078.0466 | | | | | | | | | | | -182.90 | | | | | | | | | | | 384 | | | | | | | | | | | - | | | | | 400 | | | | | | | | | | | | | | | | | 1 | | | | | | | | | | | --- | | | | | | | | | | | K.LLEYGNMIVQEQENVKR.V + Oxidation (M) | | | | | | | | | | | | | | | |  | | | 2088.7983 | 2087.7910 | | | | | | | | | | 2088.1619 | | | | | | | | | | | -177.62 | | | | | | | | | | | 269 | | | | | | | | | | | - | | | | | 287 | | | | | | | | | | | | | | | | | 0 | | | | | | | | | | | --- | | | | | | | | | | | R.VPIIVTGNDFSTLYAPLIR.D | | | | | | | | | | | | | | | |  | | | 2088.7983 | 2087.7910 | | | | | | | | | | 2088.1619 | | | | | | | | | | | -177.62 | | | | | | | | | | | 269 | | | | | | | | | | | - | | | | | 287 | | | | | | | | | | | | | | | | | 0 | | | | | | | | | | | 101 | | | | | | | | | | | R.VPIIVTGNDFSTLYAPLIR.D | | | | | | | | | | | | | | | |  | | | 2351.5889 | 2350.5816 | | | | | | | | | | 2351.0443 | | | | | | | | | | | -196.79 | | | | | | | | | | | 173 | | | | | | | | | | | - | | | | | 195 | | | | | | | | | | | | | | | | | 0 | | | | | | | | | | | --- | | | | | | | | | | | K.MGINPIMMSAGELESGNAGEPAK.L + 3 Oxidation (M) | | | | | | | | | | | | | | | |  | | | 2352.6926 | 2351.6853 | | | | | | | | | | 2351.0443 | | | | | | | | | | | 273 | | | | | | | | | | | 173 | | | | | | | | | | | - | | | | | 195 | | | | | | | | | | | | | | | | | 0 | | | | | | | | | | | --- | | | | | | | | | | | K.MGINPIMMSAGELESGNAGEPAK.L + 3 Oxidation (M) | | | | | | | | | | | | | | | |  | | | 3354.8992 | 3353.8919 | | | | | | | | | | 3353.5333 | | | | | | | | | | | 107 | | | | | | | | | | | 81 | | | | | | | | | | | - | | | | | 111 | | | | | | | | | | | | | | | | | 0 | | | | | | | | | | | --- | | | | | | | | | | | K.GAVDSLFQAPMGTGTHYAVMSSYDYISQGLR.T + 2 Oxidation (M) | | | | | | | | | | | | | | | |  | | | No match to: 713.2720, 716.2274, 724.2481, 734.3523, 768.3625, 787.2740, 809.2766, 842.3466, 860.9086, 868.3715, 882.3726, 944.2845, 944.2845, 956.2769, 956.2769, 970.2636, 972.2759, 972.2759, 1014.3777, 1024.3063, 1051.4550, 1053.3014, 1059.3715, 1080.3021, 1096.3281, 1110.3168, 1127.3427, 1136.3677, 1138.3813, 1142.3967, 1151.4285, 1154.3693, 1156.4576, 1168.4752, 1179.4056, 1218.4017, 1228.3729, 1244.3571, 1252.3949, 1255.4159, 1286.4408, 1288.3921, 1291.4493, 1303.4675, 1315.4139, 1332.4822, 1361.3942, 1418.4640, 1422.4664, 1435.4280, 1435.4280, 1454.4426, 1467.4832, 1478.5458, 1487.4413, 1559.4954, 1605.4669, 1639.5164, 1642.5680, 1703.5236, 1707.4856, 1723.5178, 1739.4742, 1746.6118, 1747.4364, 1778.6056, 1788.5625, 1793.6285, 1810.5824, 1858.6228, 1881.6268, 1904.6035, 1926.5941, 1930.6514, 1938.5894, 1960.5604, 2002.6788, 2014.3959, 2014.7017, 2094.6782, 2172.6887, 2220.7153, 2224.6853, 2238.7339, 2245.7854, 2260.6833, 2282.6040, 2290.6528, 2403.6399, 3290.0051 | | | | | | | | | | | | | | | | | | | | | | | | | | | | | | | | | | | | | | | | | | | | | | | | | | | | | | | | | | | | | | | | | | | | | | | | | | | | | | | | | | | | | | | | | | | | | | | | | | | |  | | | | | | | **S22** [**Cs9g03300.1**](http://zhangyang-pc/mascot/cgi/protein_view.pl?file=../data/20140118/F012129.dat&hit=3) **Mass: 64744 Score: 798 Expect: 7e-076 Matches: 39** | | | | | | | | | | | | | | | | | | | | | | | | | | | | | | | | | | | | | | | | | | | | | | | | | | | | | | | | | | | | | | | | | | | | | | | | | | | | | | | | | | | | | | | | | | | | | | | | | | | |  | | | | | | | Observed | | | | Mr(expt) | | | | | | | | | | Mr(calc) | | | | | | | | | | | ppm | | | | | | | | | | | Start | | | | | | | | | | |  | | | | | | | | | | | End | | | | | | | | | | | Miss | | | | | | | | | | | Ions | | | | | | | | | | | Peptide | | | | | | | | | | | | |  | | | 700.2453 | | | | 699.2380 | | | | | | | | | | 699.2898 | | | | | | | | | | | -73.99 | | | | | | | | | | | 71 | | | | | | | | | | | - | | | | | | | | | | | 76 | | | | | | | | | | | 0 | | | | | | | | | | | --- | | | | | | | | | | | K.DGYAMK.K + Oxidation (M) | | | | | | | | | | | | |  | | | 733.2252 | | | | 732.2179 | | | | | | | | | | 732.3555 | | | | | | | | | | | -187.89 | | | | | | | | | | | 339 | | | | | | | | | | | - | | | | | | | | | | | 345 | | | | | | | | | | | 0 | | | | | | | | | | | --- | | | | | | | | | | | K.APGFGER.K | | | | | | | | | | | | |  | | | 759.3013 | | | | 758.2941 | | | | | | | | | | 758.4538 | | | | | | | | | | | -210.60 | | | | | | | | | | | 188 | | | | | | | | | | | - | | | | | | | | | | | 194 | | | | | | | | | | | 0 | | | | | | | | | | | --- | | | | | | | | | | | K.ALVSELK.Q | | | | | | | | | | | | |  | | | 762.2480 | | | | 761.2407 | | | | | | | | | | 761.4283 | | | | | | | | | | | -246.36 | | | | | | | | | | | 181 | | | | | | | | | | | - | | | | | | | | | | | 187 | | | | | | | | | | | 1 | | | | | | | | | | | --- | | | | | | | | | | | R.GIEKTSK.A | | | | | | | | | | | | |  | | | 773.2648 | | | | 772.2576 | | | | | | | | | | 772.3868 | | | | | | | | | | | -167.31 | | | | | | | | | | | 65 | | | | | | | | | | | - | | | | | | | | | | | 70 | | | | | | | | | | | 0 | | | | | | | | | | | --- | | | | | | | | | | | K.DLHFNK.D | | | | | | | | | | | | |  | | | 788.3002 | | | | 787.2930 | | | | | | | | | | 787.4188 | | | | | | | | | | | -159.79 | | | | | | | | | | | 424 | | | | | | | | | | | - | | | | | | | | | | | 429 | | | | | | | | | | | 1 | | | | | | | | | | | --- | | | | | | | | | | | R.EKLNER.I | | | | | | | | | | | | |  | | | 899.3227 | | | | 898.3154 | | | | | | | | | | 898.4508 | | | | | | | | | | | -150.73 | | | | | | | | | | | 532 | | | | | | | | | | | - | | | | | | | | | | | 539 | | | | | | | | | | | 0 | | | | | | | | | | | --- | | | | | | | | | | | K.VLSSDNHK.Y | | | | | | | | | | | | |  | | | 931.3215 | | | | 930.3143 | | | | | | | | | | 930.5022 | | | | | | | | | | | -202.00 | | | | | | | | | | | 234 | | | | | | | | | | | - | | | | | | | | | | | 242 | | | | | | | | | | | 0 | | | | | | | | | | | --- | | | | | | | | | | | K.GVVTLEEGK.S | | | | | | | | | | | | |  | | | 959.3317 | | | | 958.3244 | | | | | | | | | | 958.4971 | | | | | | | | | | | -180.20 | | | | | | | | | | | 364 | | | | | | | | | | | - | | | | | | | | | | | 372 | | | | | | | | | | | 0 | | | | | | | | | | | --- | | | | | | | | | | | R.DEVGLALDK.V | | | | | | | | | | | | |  | | | 1059.3911 | | | | 1058.3838 | | | | | | | | | | 1058.5972 | | | | | | | | | | | -201.53 | | | | | | | | | | | 233 | | | | | | | | | | | - | | | | | | | | | | | 242 | | | | | | | | | | | 1 | | | | | | | | | | | --- | | | | | | | | | | | R.KGVVTLEEGK.S | | | | | | | | | | | | |  | | | 1182.5112 | | | | 1181.5039 | | | | | | | | | | 1181.7020 | | | | | | | | | | | -167.60 | | | | | | | | | | | 85 | | | | | | | | | | | - | | | | | | | | | | | 96 | | | | | | | | | | | 0 | | | | | | | | | | | --- | | | | | | | | | | | K.LADLVGVTLGPK.G | | | | | | | | | | | | |  | | | 1182.5112 | | | | 1181.5039 | | | | | | | | | | 1181.7020 | | | | | | | | | | | -167.60 | | | | | | | | | | | 85 | | | | | | | | | | | - | | | | | | | | | | | 96 | | | | | | | | | | | 0 | | | | | | | | | | | 86 | | | | | | | | | | | K.LADLVGVTLGPK.G | | | | | | | | | | | | |  | | | 1229.4681 | | | | 1228.4608 | | | | | | | | | | 1228.6775 | | | | | | | | | | | -176.37 | | | | | | | | | | | 455 | | | | | | | | | | | - | | | | | | | | | | | 465 | | | | | | | | | | | 1 | | | | | | | | | | | --- | | | | | | | | | | | K.LRVEDALNATK.A | | | | | | | | | | | | |  | | | 1270.4856 | | | | 1269.4783 | | | | | | | | | | 1269.6928 | | | | | | | | | | | -168.95 | | | | | | | | | | | 293 | | | | | | | | | | | - | | | | | | | | | | | 303 | | | | | | | | | | | 0 | | | | | | | | | | | --- | | | | | | | | | | | R.DLINVLEDAIR.G | | | | | | | | | | | | |  | | | 1270.4856 | | | | 1269.4783 | | | | | | | | | | 1269.6928 | | | | | | | | | | | -168.95 | | | | | | | | | | | 293 | | | | | | | | | | | - | | | | | | | | | | | 303 | | | | | | | | | | | 0 | | | | | | | | | | | 96 | | | | | | | | | | | R.DLINVLEDAIR.G | | | | | | | | | | | | |  | | | 1280.5579 | | | | 1279.5506 | | | | | | | | | | 1279.7612 | | | | | | | | | | | -164.55 | | | | | | | | | | | 168 | | | | | | | | | | | - | | | | | | | | | | | 180 | | | | | | | | | | | 0 | | | | | | | | | | | --- | | | | | | | | | | | K.VVAAGANPVLITR.G | | | | | | | | | | | | |  | | | 1280.5579 | | | | 1279.5506 | | | | | | | | | | 1279.7612 | | | | | | | | | | | -164.55 | | | | | | | | | | | 168 | | | | | | | | | | | - | | | | | | | | | | | 180 | | | | | | | | | | | 0 | | | | | | | | | | | 83 | | | | | | | | | | | K.VVAAGANPVLITR.G | | | | | | | | | | | | |  | | | 1395.5354 | | | | 1394.5281 | | | | | | | | | | 1394.8246 | | | | | | | | | | | -212.52 | | | | | | | | | | | 85 | | | | | | | | | | | - | | | | | | | | | | | 98 | | | | | | | | | | | 1 | | | | | | | | | | | --- | | | | | | | | | | | K.LADLVGVTLGPKGR.N | | | | | | | | | | | | |  | | | 1480.4482 | | | | 1479.4409 | | | | | | | | | | 1479.6841 | | | | | | | | | | | -164.35 | | | | | | | | | | | 412 | | | | | | | | | | | - | | | | | | | | | | | 423 | | | | | | | | | | | 0 | | | | | | | | | | | --- | | | | | | | | | | | R.TLIENAEQDYER.E | | | | | | | | | | | | |  | | | 1480.4482 | | | | 1479.4409 | | | | | | | | | | 1479.6841 | | | | | | | | | | | -164.35 | | | | | | | | | | | 412 | | | | | | | | | | | - | | | | | | | | | | | 423 | | | | | | | | | | | 0 | | | | | | | | | | | 106 | | | | | | | | | | | R.TLIENAEQDYER.E | | | | | | | | | | | | |  | | | 1505.4575 | | | | 1504.4502 | | | | | | | | | | 1504.7086 | | | | | | | | | | | -171.69 | | | | | | | | | | | 259 | | | | | | | | | | | - | | | | | | | | | | | 271 | | | | | | | | | | | 0 | | | | | | | | | | | --- | | | | | | | | | | | R.GYISPYFVTDSEK.M | | | | | | | | | | | | |  | | | 1505.4575 | | | | 1504.4502 | | | | | | | | | | 1504.7086 | | | | | | | | | | | -171.69 | | | | | | | | | | | 259 | | | | | | | | | | | - | | | | | | | | | | | 271 | | | | | | | | | | | 0 | | | | | | | | | | | 59 | | | | | | | | | | | R.GYISPYFVTDSEK.M | | | | | | | | | | | | |  | | | 1541.5173 | | | | 1540.5100 | | | | | | | | | | 1540.7620 | | | | | | | | | | | -163.57 | | | | | | | | | | | 121 | | | | | | | | | | | - | | | | | | | | | | | 134 | | | | | | | | | | | 0 | | | | | | | | | | | --- | | | | | | | | | | | K.EVELEDPVENIGAK.L | | | | | | | | | | | | |  | | | 1737.5475 | | | | 1736.5402 | | | | | | | | | | 1736.8217 | | | | | | | | | | | -162.04 | | | | | | | | | | | 412 | | | | | | | | | | | - | | | | | | | | | | | 425 | | | | | | | | | | | 1 | | | | | | | | | | | --- | | | | | | | | | | | R.TLIENAEQDYEREK.L | | | | | | | | | | | | |  | | | 1774.5828 | | | | 1773.5755 | | | | | | | | | | 1773.8632 | | | | | | | | | | | -162.19 | | | | | | | | | | | 492 | | | | | | | | | | | - | | | | | | | | | | | 507 | | | | | | | | | | | 1 | | | | | | | | | | | --- | | | | | | | | | | | K.ETLDNDEEKVGADIVK.R | | | | | | | | | | | | |  | | | 1834.6842 | | | | 1833.6769 | | | | | | | | | | 1833.9836 | | | | | | | | | | | -167.23 | | | | | | | | | | | 347 | | | | | | | | | | | - | | | | | | | | | | | 363 | | | | | | | | | | | 0 | | | | | | | | | | | --- | | | | | | | | | | | K.SQYLDDIAILTGGTVIR.D | | | | | | | | | | | | |  | | | 1834.6842 | | | | 1833.6769 | | | | | | | | | | 1833.9836 | | | | | | | | | | | -167.23 | | | | | | | | | | | 347 | | | | | | | | | | | - | | | | | | | | | | | 363 | | | | | | | | | | | 0 | | | | | | | | | | | 107 | | | | | | | | | | | K.SQYLDDIAILTGGTVIR.D | | | | | | | | | | | | |  | | | 1899.7083 | | | | 1898.7010 | | | | | | | | | | 1899.0313 | | | | | | | | | | | -173.92 | | | | | | | | | | | 433 | | | | | | | | | | | - | | | | | | | | | | | 451 | | | | | | | | | | | 0 | | | | | | | | | | | --- | | | | | | | | | | | K.LSGGVAVIQVGAQTETELK.E | | | | | | | | | | | | |  | | | 1904.5375 | | | | 1903.5302 | | | | | | | | | | 1903.8444 | | | | | | | | | | | -165.03 | | | | | | | | | | | 243 | | | | | | | | | | | - | | | | | | | | | | | 258 | | | | | | | | | | | 0 | | | | | | | | | | | --- | | | | | | | | | | | K.SAENMLYVVEGMQFDR.G + Oxidation (M) | | | | | | | | | | | | |  | | | 1920.5151 | | | | 1919.5078 | | | | | | | | | | 1919.8393 | | | | | | | | | | | -172.68 | | | | | | | | | | | 243 | | | | | | | | | | | - | | | | | | | | | | | 258 | | | | | | | | | | | 0 | | | | | | | | | | | --- | | | | | | | | | | | K.SAENMLYVVEGMQFDR.G + 2 Oxidation (M) | | | | | | | | | | | | |  | | | 1920.5151 | | | | 1919.5078 | | | | | | | | | | 1919.8393 | | | | | | | | | | | -172.68 | | | | | | | | | | | 243 | | | | | | | | | | | - | | | | | | | | | | | 258 | | | | | | | | | | | 0 | | | | | | | | | | | 64 | | | | | | | | | | | K.SAENMLYVVEGMQFDR.G + 2 Oxidation (M) | | | | | | | | | | | | |  | | | 1936.5139 | | | | 1935.5066 | | | | | | | | | | 1935.1153 | | | | | | | | | | | 202 | | | | | | | | | | | 78 | | | | | | | | | | | - | | | | | | | | | | | 96 | | | | | | | | | | | 1 | | | | | | | | | | | --- | | | | | | | | | | | K.LQNGVNKLADLVGVTLGPK.G | | | | | | | | | | | | |  | | | 1962.7512 | | | | 1961.7439 | | | | | | | | | | 1962.0786 | | | | | | | | | | | -170.56 | | | | | | | | | | | 346 | | | | | | | | | | | - | | | | | | | | | | | 363 | | | | | | | | | | | 1 | | | | | | | | | | | --- | | | | | | | | | | | R.KSQYLDDIAILTGGTVIR.D | | | | | | | | | | | | |  | | | 2047.6725 | | | | 2046.6652 | | | | | | | | | | 2047.0334 | | | | | | | | | | | -179.85 | | | | | | | | | | | 407 | | | | | | | | | | | - | | | | | | | | | | | 423 | | | | | | | | | | | 1 | | | | | | | | | | | --- | | | | | | | | | | | R.VAQIRTLIENAEQDYER.E | | | | | | | | | | | | |  | | | 2156.7830 | | | | 2155.7757 | | | | | | | | | | 2156.1689 | | | | | | | | | | | -182.33 | | | | | | | | | | | 433 | | | | | | | | | | | - | | | | | | | | | | | 453 | | | | | | | | | | | 1 | | | | | | | | | | | --- | | | | | | | | | | | K.LSGGVAVIQVGAQTETELKEK.K | | | | | | | | | | | | |  | | | 2429.8330 | | | | 2428.8257 | | | | | | | | | | 2429.2650 | | | | | | | | | | | -180.81 | | | | | | | | | | | 143 | | | | | | | | | | | - | | | | | | | | | | | 167 | | | | | | | | | | | 0 | | | | | | | | | | | --- | | | | | | | | | | | K.TNDLAGDGTTTSVVLAQGLIAEGVK.V | | | | | | | | | | | | |  | | | 2429.8330 | | | | 2428.8257 | | | | | | | | | | 2429.2650 | | | | | | | | | | | -180.81 | | | | | | | | | | | 143 | | | | | | | | | | | - | | | | | | | | | | | 167 | | | | | | | | | | | 0 | | | | | | | | | | | 59 | | | | | | | | | | | K.TNDLAGDGTTTSVVLAQGLIAEGVK.V | | | | | | | | | | | | |  | | | 2451.8225 | | | | 2450.8152 | | | | | | | | | | 2451.1080 | | | | | | | | | | | -119.44 | | | | | | | | | | | 2 | | | | | | | | | | | - | | | | | | | | | | | 24 | | | | | | | | | | | 1 | | | | | | | | | | | --- | | | | | | | | | | | M.ASTFTAMSSISSMIAPNGRMTDK.K + 3 Oxidation (M) | | | | | | | | | | | | |  | | | 2463.6775 | | | | 2462.6702 | | | | | | | | | | 2463.1264 | | | | | | | | | | | -185.19 | | | | | | | | | | | 540 | | | | | | | | | | | - | | | | | | | | | | | 562 | | | | | | | | | | | 0 | | | | | | | | | | | --- | | | | | | | | | | | K.YGYNAATGNYEDLMAAGIIDPTK.V + Oxidation (M) | | | | | | | | | | | | |  | | | No match to: 705.2482, 710.2504, 713.2806, 716.2344, 720.2944, 722.2529, 730.2313, 737.2668, 739.2731, 744.2289, 747.2366, 755.2318, 758.2225, 768.3703, 816.3086, 818.2868, 842.3562, 850.3733, 868.3818, 882.3918, 915.2711, 1051.5021, 1159.2756, 1165.5138, 1181.4194, 1225.4768, 1260.4200, 1275.4528, 1284.5302, 1286.4734, 1292.4728, 1315.4507, 1329.4225, 1428.4126, 1434.4733, 1447.4467, 1462.4594, 1465.5106, 1479.5248, 1494.4667, 1502.4242, 1519.4546, 1523.4836, 1527.4478, 1540.5313, 1606.3250, 1607.3439, 1607.8127, 1608.4564, 1609.6290, 1609.6290, 1658.5895, 1698.5834, 1700.6221, 1716.5646, 17.5452, 1833.6379, 1856.5507, 1856.5507, 1872.5566, 1903.5717, 1934.5176, 1952.5078, 1954.5082, 2064.5176, 2080.5505, 2220.7478, 2238.7444, 2245.7815, 2305.6841, 2399.7405 | | | | | | | | | | | | | | | | | | | | | | | | | | | | | | | | | | | | | | | | | | | | | | | | | | | | | | | | | | | | | | | | | | | | | | | | | | | | | | | | | | | | | | | | | | | | | | | | | | | |  | | | | | | | **S35** [**Cs1g25510.1**](http://zhangyang-pc/mascot/cgi/protein_view.pl?file=../data/20140118/F012143.dat&hit=3) **Mass: 40452 Score: 258 Expect: 7e-022 Matches: 22** | | | | | | | | | | | | | | | | | | | | | | | | | | | | | | | | | | | | | | | | | | | | | | | | | | | | | | | | | | | | | | | | | | | | | | | | | | | | | | | | | | | | | | | | | | | | | | | | | | | |  | | | | | | | Observed | | | | | | | | | | Mr(expt) | | | | | | | | | | Mr(calc) | | | | | | | | | | | ppm | | | | | | | | | | | Start | | | | | | | | | | | |  | | | | | | | | | | End | | | | | | | | | | | Miss | | | | | | | | | | | Ions | | | | | | | | | | | Peptide | | | | | | |  | | | 706.2405 | | | | | | | | | | 705.2332 | | | | | | | | | | 705.3810 | | | | | | | | | | | -209.48 | | | | | | | | | | | 83 | | | | | | | | | | | | - | | | | | | | | | | 88 | | | | | | | | | | | 0 | | | | | | | | | | | --- | | | | | | | | | | | K.TPYIGR.C | | | | | | |  | | | 717.1848 | | | | | | | | | | 716.1775 | | | | | | | | | | 716.3203 | | | | | | | | | | | -199.45 | | | | | | | | | | | 231 | | | | | | | | | | | | - | | | | | | | | | | 235 | | | | | | | | | | | 0 | | | | | | | | | | | --- | | | | | | | | | | | K.MFFEK.H + Oxidation (M) | | | | | | |  | | | 733.2141 | | | | | | | | | | 732.2068 | | | | | | | | | | 732.3555 | | | | | | | | | | | -203.04 | | | | | | | | | | | 270 | | | | | | | | | | | | - | | | | | | | | | | 275 | | | | | | | | | | | 0 | | | | | | | | | | | --- | | | | | | | | | | | K.APENFR.L | | | | | | |  | | | 807.2721 | | | | | | | | | | 806.2648 | | | | | | | | | | 806.4286 | | | | | | | | | | | -203.16 | | | | | | | | | | | 276 | | | | | | | | | | | | - | | | | | | | | | | 282 | | | | | | | | | | | 0 | | | | | | | | | | | --- | | | | | | | | | | | R.LDFAVSR.E | | | | | | |  | | | 827.2456 | | | | | | | | | | 826.2383 | | | | | | | | | | 826.4007 | | | | | | | | | | | -196.49 | | | | | | | | | | | 292 | | | | | | | | | | | | - | | | | | | | | | | 297 | | | | | | | | | | | 0 | | | | | | | | | | | --- | | | | | | | | | | | K.MYIQTR.M + Oxidation (M) | | | | | | |  | | | 990.3227 | | | | | | | | | | 989.3154 | | | | | | | | | | 989.4930 | | | | | | | | | | | -179.48 | | | | | | | | | | | 268 | | | | | | | | | | | | - | | | | | | | | | | 275 | | | | | | | | | | | 1 | | | | | | | | | | | --- | | | | | | | | | | | K.EKAPENFR.L | | | | | | |  | | | 1332.3661 | | | | | | | | | | 1331.3588 | | | | | | | | | | 1331.6736 | | | | | | | | | | | -236.41 | | | | | | | | | | | 226 | | | | | | | | | | | | - | | | | | | | | | | 235 | | | | | | | | | | | 1 | | | | | | | | | | | --- | | | | | | | | | | | R.GFLWKMFFEK.H | | | | | | |  | | | 1334.3590 | | | | | | | | | | 1333.3517 | | | | | | | | | | 1333.5795 | | | | | | | | | | | -170.79 | | | | | | | | | | | 312 | | | | | | | | | | | | - | | | | | | | | | | 322 | | | | | | | | | | | 0 | | | | | | | | | | | --- | | | | | | | | | | | K.DNTYVYMCGLR.G | | | | | | |  | | | 1350.3473 | | | | | | | | | | 1349.3400 | | | | | | | | | | 1349.5744 | | | | | | | | | | | -173.67 | | | | | | | | | | | 312 | | | | | | | | | | | | - | | | | | | | | | | 322 | | | | | | | | | | | 0 | | | | | | | | | | | --- | | | | | | | | | | | K.DNTYVYMCGLR.G + Oxidation (M) | | | | | | |  | | | 1378.4725 | | | | | | | | | | 1377.4652 | | | | | | | | | | 1377.7140 | | | | | | | | | | | -180.55 | | | | | | | | | | | 165 | | | | | | | | | | | | - | | | | | | | | | | 176 | | | | | | | | | | | 0 | | | | | | | | | | | --- | | | | | | | | | | | R.LVYTNENGEIVK.G | | | | | | |  | | | 1387.4668 | | | | | | | | | | 1386.4595 | | | | | | | | | | 1386.6991 | | | | | | | | | | | -172.75 | | | | | | | | | | | 119 | | | | | | | | | | | | - | | | | | | | | | | 132 | | | | | | | | | | | 0 | | | | | | | | | | | --- | | | | | | | | | | | K.EGQSIGVIADGVDK.N | | | | | | |  | | | 1444.4330 | | | | | | | | | | 1443.4257 | | | | | | | | | | 1443.7578 | | | | | | | | | | | -230.00 | | | | | | | | | | | 194 | | | | | | | | | | | | - | | | | | | | | | | 206 | | | | | | | | | | | 1 | | | | | | | | | | | --- | | | | | | | | | | | K.ITGPVGKEMLMPR.D + Oxidation (M) | | | | | | |  | | | 1460.4182 | | | | | | | | | | 1459.4109 | | | | | | | | | | 1459.7527 | | | | | | | | | | | -234.13 | | | | | | | | | | | 194 | | | | | | | | | | | | - | | | | | | | | | | 206 | | | | | | | | | | | 1 | | | | | | | | | | | --- | | | | | | | | | | | K.ITGPVGKEMLMPR.D + 2 Oxidation (M) | | | | | | |  | | | 1521.5273 | | | | | | | | | | 1520.5200 | | | | | | | | | | 1520.7736 | | | | | | | | | | | -166.72 | | | | | | | | | | | 270 | | | | | | | | | | | | - | | | | | | | | | | 282 | | | | | | | | | | | 1 | | | | | | | | | | | --- | | | | | | | | | | | K.APENFRLDFAVSR.E | | | | | | |  | | | 1534.5558 | | | | | | | | | | 1533.5485 | | | | | | | | | | 1533.8151 | | | | | | | | | | | -173.79 | | | | | | | | | | | 164 | | | | | | | | | | | | - | | | | | | | | | | 176 | | | | | | | | | | | 1 | | | | | | | | | | | --- | | | | | | | | | | | K.RLVYTNENGEIVK.G | | | | | | |  | | | 1534.5558 | | | | | | | | | | 1533.5485 | | | | | | | | | | 1533.8151 | | | | | | | | | | | -173.79 | | | | | | | | | | | 164 | | | | | | | | | | | | - | | | | | | | | | | 176 | | | | | | | | | | | 1 | | | | | | | | | | | 48 | | | | | | | | | | | K.RLVYTNENGEIVK.G | | | | | | |  | | | 1625.4497 | | | | | | | | | | 1624.4424 | | | | | | | | | | 1624.7806 | | | | | | | | | | | -208.16 | | | | | | | | | | | 298 | | | | | | | | | | | | - | | | | | | | | | | 310 | | | | | | | | | | | 0 | | | | | | | | | | | --- | | | | | | | | | | | R.MAEYANELWELLK.K + Oxidation (M) | | | | | | |  | | | 1630.4965 | | | | | | | | | | 1629.4892 | | | | | | | | | | 1629.7886 | | | | | | | | | | | -183.69 | | | | | | | | | | | 141 | | | | | | | | | | | | - | | | | | | | | | | 156 | | | | | | | | | | | 0 | | | | | | | | | | | --- | | | | | | | | | | | R.LYSIASSALGDFGDSK.T | | | | | | |  | | | 1630.4965 | | | | | | | | | | 1629.4892 | | | | | | | | | | 1629.7886 | | | | | | | | | | | -183.69 | | | | | | | | | | | 141 | | | | | | | | | | | | - | | | | | | | | | | 156 | | | | | | | | | | | 0 | | | | | | | | | | | 79 | | | | | | | | | | | R.LYSIASSALGDFGDSK.T | | | | | | |  | | | 1944.6683 | | | | | | | | | | 1943.6610 | | | | | | | | | | 1944.0139 | | | | | | | | | | | -181.51 | | | | | | | | | | | 207 | | | | | | | | | | | | - | | | | | | | | | | 225 | | | | | | | | | | | 0 | | | | | | | | | | | --- | | | | | | | | | | | R.DPNATVIMLATGTGIAPFR.G | | | | | | |  | | | 1960.6490 | | | | | | | | | | 1959.6417 | | | | | | | | | | 1960.0088 | | | | | | | | | | | -187.28 | | | | | | | | | | | 207 | | | | | | | | | | | | - | | | | | | | | | | 225 | | | | | | | | | | | 0 | | | | | | | | | | | --- | | | | | | | | | | | R.DPNATVIMLATGTGIAPFR.G + Oxidation (M) | | | | | | |  | | | 1960.6490 | | | | | | | | | | 1959.6417 | | | | | | | | | | 1960.0088 | | | | | | | | | | | -187.28 | | | | | | | | | | | 207 | | | | | | | | | | | | - | | | | | | | | | | 225 | | | | | | | | | | | 0 | | | | | | | | | | | 80 | | | | | | | | | | | R.DPNATVIMLATGTGIAPFR.G + Oxidation (M) | | | | | | |  | | | No match to: 728.2285, 744.2230, 747.2225, 755.1862, 763.2348, 768.3451, 790.2172, 822.2330, 824.2155, 825.2461, 830.2616, 842.3428, 844.2333, 868.3377, 1016.3953, 1059.3734, 1096.4272, 1179.2747, 1191.2908, 1207.2670, 1252.3771, 1285.3762, 1285.3762, 1297.3741, 1300.3842, 1307.3539, 1313.3655, 1316.3645, 1316.3645, 1319.3630, 1336.3639, 1359.4205, 1365.3352, 1366.3402, 1376.4050, 1390.3744, 1407.3517, 1407.3517, 1423.3024, 1426.3403, 1429.3894, 1429.3894, 1439.2928, 1442.3406, 1494.3929, 1498.5299, 1535.5460, 1551.3906, 1629.4695, 1634.5586, 1641.4719, 1641.4719, 1657.4802, 1663.4701, 1689.4360, 1695.4114, 1707.4270, 1711.5585, 1805.5280, 1818.4780, 1845.6422, 1878.5934, 1895.6687, 1896.6874, 1896.6874, 1912.6772, 1916.6390, 1942.6422, 1945.6536, 1958.6615, 1974.6561, 1976.6633, 1976.6633, 1982.6260, 1988.6765, 1998.5851, 2220.7407, 2238.7332, 2448.8313, 2483.7339, 2621.7073, 2633.7312, 2679.6970, 2683.7214, 2685.6755, 2697.6948, 2712.7090, 2713.6833 | | | | | | | | | | | | | | | | | | | | | | | | | | | | | | | | | | | | | | | | | | | | | | | | | | | | | | | | | | | | | | | | | | | | | | | | | | | | | | | | | | | | | | | | | | | | | | | | | | | |  | | | | | | | **S37** [**Cs1g25510.1**](http://zhangyang-pc/mascot/cgi/protein_view.pl?file=../data/20140118/F012176.dat&hit=2) **Mass: 40452 Score: 240 Expect: 4.4e-020 Matches: 21** | | | | | | | | | | | | | | | | | | | | | | | | | | | | | | | | | | | | | | | | | | | | | | | | | | | | | | | | | | | | | | | | | | | | | | | | | | | | | | | | | | | | | | | | | | | | | | | | | | | |  | | | | | | | Observed | | | | | | | | | | Mr(expt) | | | | | | | | | | Mr(calc) | | | | | | | | | | | ppm | | | | | | | | | | | Start | | | | | | | | | | | |  | | | | | | | | | | End | | | | | | | | | | | Miss | | | | | | | | | | | Ions | | | | | | | | | | | Peptide | | | | | | |  | | | 706.2394 | | | | | | | | | | 705.2321 | | | | | | | | | | 705.3810 | | | | | | | | | | | -211.04 | | | | | | | | | | | 83 | | | | | | | | | | | | - | | | | | | | | | | 88 | | | | | | | | | | | 0 | | | | | | | | | | | --- | | | | | | | | | | | K.TPYIGR.C | | | | | | |  | | | 717.1836 | | | | | | | | | | 716.1764 | | | | | | | | | | 716.3203 | | | | | | | | | | | -200.98 | | | | | | | | | | | 231 | | | | | | | | | | | | - | | | | | | | | | | 235 | | | | | | | | | | | 0 | | | | | | | | | | | --- | | | | | | | | | | | K.MFFEK.H + Oxidation (M) | | | | | | |  | | | 733.2150 | | | | | | | | | | 732.2078 | | | | | | | | | | 732.3555 | | | | | | | | | | | -201.70 | | | | | | | | | | | 270 | | | | | | | | | | | | - | | | | | | | | | | 275 | | | | | | | | | | | 0 | | | | | | | | | | | --- | | | | | | | | | | | K.APENFR.L | | | | | | |  | | | 792.2358 | | | | | | | | | | 791.2285 | | | | | | | | | | 791.3670 | | | | | | | | | | | -174.96 | | | | | | | | | | | 201 | | | | | | | | | | | | - | | | | | | | | | | 206 | | | | | | | | | | | 0 | | | | | | | | | | | --- | | | | | | | | | | | K.EMLMPR.D + Oxidation (M) | | | | | | |  | | | 807.2744 | | | | | | | | | | 806.2671 | | | | | | | | | | 806.4286 | | | | | | | | | | | -200.36 | | | | | | | | | | | 276 | | | | | | | | | | | | - | | | | | | | | | | 282 | | | | | | | | | | | 0 | | | | | | | | | | | --- | | | | | | | | | | | R.LDFAVSR.E | | | | | | |  | | | 808.2452 | | | | | | | | | | 807.2379 | | | | | | | | | | 807.3619 | | | | | | | | | | | -153.55 | | | | | | | | | | | 201 | | | | | | | | | | | | - | | | | | | | | | | 206 | | | | | | | | | | | 0 | | | | | | | | | | | --- | | | | | | | | | | | K.EMLMPR.D + 2 Oxidation (M) | | | | | | |  | | | 827.2407 | | | | | | | | | | 826.2334 | | | | | | | | | | 826.4007 | | | | | | | | | | | -202.40 | | | | | | | | | | | 292 | | | | | | | | | | | | - | | | | | | | | | | 297 | | | | | | | | | | | 0 | | | | | | | | | | | --- | | | | | | | | | | | K.MYIQTR.M + Oxidation (M) | | | | | | |  | | | 990.3164 | | | | | | | | | | 989.3091 | | | | | | | | | | 989.4930 | | | | | | | | | | | -185.89 | | | | | | | | | | | 268 | | | | | | | | | | | | - | | | | | | | | | | 275 | | | | | | | | | | | 1 | | | | | | | | | | | --- | | | | | | | | | | | K.EKAPENFR.L | | | | | | |  | | | 1332.3606 | | | | | | | | | | 1331.3533 | | | | | | | | | | 1331.6736 | | | | | | | | | | | -240.54 | | | | | | | | | | | 226 | | | | | | | | | | | | - | | | | | | | | | | 235 | | | | | | | | | | | 1 | | | | | | | | | | | --- | | | | | | | | | | | R.GFLWKMFFEK.H | | | | | | |  | | | 1350.3412 | | | | | | | | | | 1349.3339 | | | | | | | | | | 1349.5744 | | | | | | | | | | | -178.19 | | | | | | | | | | | 312 | | | | | | | | | | | | - | | | | | | | | | | 322 | | | | | | | | | | | 0 | | | | | | | | | | | --- | | | | | | | | | | | K.DNTYVYMCGLR.G + Oxidation (M) | | | | | | |  | | | 1378.4530 | | | | | | | | | | 1377.4457 | | | | | | | | | | 1377.7140 | | | | | | | | | | | -194.71 | | | | | | | | | | | 165 | | | | | | | | | | | | - | | | | | | | | | | 176 | | | | | | | | | | | 0 | | | | | | | | | | | --- | | | | | | | | | | | R.LVYTNENGEIVK.G | | | | | | |  | | | 1387.4194 | | | | | | | | | | 1386.4121 | | | | | | | | | | 1386.6991 | | | | | | | | | | | -206.94 | | | | | | | | | | | 119 | | | | | | | | | | | | - | | | | | | | | | | 132 | | | | | | | | | | | 0 | | | | | | | | | | | --- | | | | | | | | | | | K.EGQSIGVIADGVDK.N | | | | | | |  | | | 1444.4304 | | | | | | | | | | 1443.4231 | | | | | | | | | | 1443.7578 | | | | | | | | | | | -231.80 | | | | | | | | | | | 194 | | | | | | | | | | | | - | | | | | | | | | | 206 | | | | | | | | | | | 1 | | | | | | | | | | | --- | | | | | | | | | | | K.ITGPVGKEMLMPR.D + Oxidation (M) | | | | | | |  | | | 1460.4276 | | | | | | | | | | 1459.4203 | | | | | | | | | | 1459.7527 | | | | | | | | | | | -227.69 | | | | | | | | | | | 194 | | | | | | | | | | | | - | | | | | | | | | | 206 | | | | | | | | | | | 1 | | | | | | | | | | | --- | | | | | | | | | | | K.ITGPVGKEMLMPR.D + 2 Oxidation (M) | | | | | | |  | | | 1534.5503 | | | | | | | | | | 1533.5430 | | | | | | | | | | 1533.8151 | | | | | | | | | | | -177.37 | | | | | | | | | | | 164 | | | | | | | | | | | | - | | | | | | | | | | 176 | | | | | | | | | | | 1 | | | | | | | | | | | --- | | | | | | | | | | | K.RLVYTNENGEIVK.G | | | | | | |  | | | 1534.5503 | | | | | | | | | | 1533.5430 | | | | | | | | | | 1533.8151 | | | | | | | | | | | -177.37 | | | | | | | | | | | 164 | | | | | | | | | | | | - | | | | | | | | | | 176 | | | | | | | | | | | 1 | | | | | | | | | | | 38 | | | | | | | | | | | K.RLVYTNENGEIVK.G | | | | | | |  | | | 1630.4778 | | | | | | | | | | 1629.4705 | | | | | | | | | | 1629.7886 | | | | | | | | | | | -195.17 | | | | | | | | | | | 141 | | | | | | | | | | | | - | | | | | | | | | | 156 | | | | | | | | | | | 0 | | | | | | | | | | | --- | | | | | | | | | | | R.LYSIASSALGDFGDSK.T | | | | | | |  | | | 1630.4778 | | | | | | | | | | 1629.4705 | | | | | | | | | | 1629.7886 | | | | | | | | | | | -195.17 | | | | | | | | | | | 141 | | | | | | | | | | | | - | | | | | | | | | | 156 | | | | | | | | | | | 0 | | | | | | | | | | | 72 | | | | | | | | | | | R.LYSIASSALGDFGDSK.T | | | | | | |  | | | 1944.6746 | | | | | | | | | | 1943.6673 | | | | | | | | | | 1944.0139 | | | | | | | | | | | -178.27 | | | | | | | | | | | 207 | | | | | | | | | | | | - | | | | | | | | | | 225 | | | | | | | | | | | 0 | | | | | | | | | | | --- | | | | | | | | | | | R.DPNATVIMLATGTGIAPFR.G | | | | | | |  | | | 1960.6412 | | | | | | | | | | 1959.6339 | | | | | | | | | | 1960.0088 | | | | | | | | | | | -191.26 | | | | | | | | | | | 207 | | | | | | | | | | | | - | | | | | | | | | | 225 | | | | | | | | | | | 0 | | | | | | | | | | | --- | | | | | | | | | | | R.DPNATVIMLATGTGIAPFR.G + Oxidation (M) | | | | | | |  | | | 1960.6412 | | | | | | | | | | 1959.6339 | | | | | | | | | | 1960.0088 | | | | | | | | | | | -191.26 | | | | | | | | | | | 207 | | | | | | | | | | | | - | | | | | | | | | | 225 | | | | | | | | | | | 0 | | | | | | | | | | | 82 | | | | | | | | | | | R.DPNATVIMLATGTGIAPFR.G + Oxidation (M) | | | | | | |  | | | No match to: 700.2249, 705.2255, 712.1760, 715.1967, 725.1655, 728.2312, 736.2231, 744.2300, 747.2279, 755.2113, 758.2551, 763.2479, 766.2068, 768.2989, 790.2022, 810.2398, 817.2485, 822.2349, 824.2086, 830.2531, 842.3422, 843.2883, 868.3086, 884.2841, 960.3494, 964.2703, 1016.4229, 1050.3239, 1059.3639, 1096.4055, 1122.3785, 1128.3405, 1252.3668, 1269.3649, 1269.3649, 1280.3744, 1281.3549, 1291.3574, 1297.3551, 1300.3934, 1303.3574, 1304.4452, 1313.3293, 1314.3890, 1316.3550, 1316.3550, 1319.3457, 1366.3353, 1376.4415, 1390.4241, 1407.3429, 1407.3429, 1412.3755, 1423.3009, 1423.3009, 1429.3779, 1439.3213, 1442.3561, 1465.4893, 1532.5275, 1551.3835, 1577.4780, 1581.5248, 1581.5248, 1629.4615, 1634.5625, 1641.4612, 1652.4749, 1657.4584, 1663.4401, 1695.4070, 1711.5137, 1802.5403, 1805.5037, 1845.6300, 1896.6716, 1896.6716, 1912.6398, 1942.6560, 1945.6578, 1958.6112, 1976.6525, 1976.6525, 1982.6162, 2238.6902, 2621.7395, 2633.6836, 2685.6631, 2697.6821 | | | | | | | | | | | | | | | | | | | | | | | | | | | | | | | | | | | | | | | | | | | | | | | | | | | | | | | | | | | | | | | | | | | | | | | | | | | | | | | | | | | | | | | | | | | | | | | | | | | |  | | | | | | | **S27** [**orange1.1t03280.1**](http://zhangyang-pc/mascot/cgi/protein_view.pl?file=../data/20140118/F012089.dat&hit=1) **Mass: 49415 Score: 567 Expect: 8.8e-053 Matches: 25** | | | | | | | | | | | | | | | | | | | | | | | | | | | | | | | | | | | | | | | | | | | | | | | | | | | | | | | | | | | | | | | | | | | | | | | | | | | | | | | | | | | | | | | | | | | | | | | | | | | |  | | | | | | | Observed | | | | Mr(expt) | | | | | | | | | | Mr(calc) | | | | | | | | | | | ppm | | | | | | | | | | | Start | | | | | | | | | | |  | | | | | | | | | | | End | | | | | | | | | | | Miss | | | | | | | | | | | Ions | | | | | | | | | | | Peptide | | | | | | | | | | | | |  | | | 705.2756 | | | | 704.2684 | | | | | | | | | | 704.3891 | | | | | | | | | | | -171.40 | | | | | | | | | | | 66 | | | | | | | | | | | - | | | | | | | | | | | 72 | | | | | | | | | | | 0 | | | | | | | | | | | --- | | | | | | | | | | | R.AVVSMAK.K | | | | | | | | | | | | |  | | | 880.3454 | | | | 879.3381 | | | | | | | | | | 879.4926 | | | | | | | | | | | -175.68 | | | | | | | | | | | 119 | | | | | | | | | | | - | | | | | | | | | | | 126 | | | | | | | | | | | 0 | | | | | | | | | | | --- | | | | | | | | | | | K.HLIQNGAK.V | | | | | | | | | | | | |  | | | 999.4255 | | | | 998.4183 | | | | | | | | | | 998.5913 | | | | | | | | | | | -173.27 | | | | | | | | | | | 143 | | | | | | | | | | | - | | | | | | | | | | | 151 | | | | | | | | | | | 0 | | | | | | | | | | | --- | | | | | | | | | | | K.FSLAPLVPR.L | | | | | | | | | | | | |  | | | 999.4255 | | | | 998.4183 | | | | | | | | | | 998.5913 | | | | | | | | | | | -173.27 | | | | | | | | | | | 143 | | | | | | | | | | | - | | | | | | | | | | | 151 | | | | | | | | | | | 0 | | | | | | | | | | | 69 | | | | | | | | | | | K.FSLAPLVPR.L | | | | | | | | | | | | |  | | | 1006.3936 | | | | 1005.3863 | | | | | | | | | | 1005.5495 | | | | | | | | | | | -162.26 | | | | | | | | | | | 400 | | | | | | | | | | | - | | | | | | | | | | | 409 | | | | | | | | | | | 0 | | | | | | | | | | | --- | | | | | | | | | | | K.FAVGTEAIAK.K | | | | | | | | | | | | |  | | | 1102.4523 | | | | 1101.4450 | | | | | | | | | | 1101.6295 | | | | | | | | | | | -167.43 | | | | | | | | | | | 260 | | | | | | | | | | | - | | | | | | | | | | | 270 | | | | | | | | | | | 0 | | | | | | | | | | | --- | | | | | | | | | | | K.RPFAAIVGGSK.V | | | | | | | | | | | | |  | | | 1102.4523 | | | | 1101.4450 | | | | | | | | | | 1101.6295 | | | | | | | | | | | -167.43 | | | | | | | | | | | 260 | | | | | | | | | | | - | | | | | | | | | | | 270 | | | | | | | | | | | 0 | | | | | | | | | | | 88 | | | | | | | | | | | K.RPFAAIVGGSK.V | | | | | | | | | | | | |  | | | 1139.3737 | | | | 1138.3664 | | | | | | | | | | 1138.5506 | | | | | | | | | | | -161.77 | | | | | | | | | | | 375 | | | | | | | | | | | - | | | | | | | | | | | 384 | | | | | | | | | | | 0 | | | | | | | | | | | --- | | | | | | | | | | | K.TFNEALDTTK.T | | | | | | | | | | | | |  | | | 1198.5238 | | | | 1197.5165 | | | | | | | | | | 1197.7333 | | | | | | | | | | | -180.96 | | | | | | | | | | | 152 | | | | | | | | | | | - | | | | | | | | | | | 162 | | | | | | | | | | | 0 | | | | | | | | | | | --- | | | | | | | | | | | R.LSELLGIQVVK.A | | | | | | | | | | | | |  | | | 1377.5222 | | | | 1376.5149 | | | | | | | | | | 1376.7187 | | | | | | | | | | | -148.04 | | | | | | | | | | | 247 | | | | | | | | | | | - | | | | | | | | | | | 259 | | | | | | | | | | | 0 | | | | | | | | | | | --- | | | | | | | | | | | K.ELDYLVGAVSSPK.R | | | | | | | | | | | | |  | | | 1377.5222 | | | | 1376.5149 | | | | | | | | | | 1376.7187 | | | | | | | | | | | -148.04 | | | | | | | | | | | 247 | | | | | | | | | | | - | | | | | | | | | | | 259 | | | | | | | | | | | 0 | | | | | | | | | | | 63 | | | | | | | | | | | K.ELDYLVGAVSSPK.R | | | | | | | | | | | | |  | | | 1463.5715 | | | | 1462.5642 | | | | | | | | | | 1462.8548 | | | | | | | | | | | -198.61 | | | | | | | | | | | 234 | | | | | | | | | | | - | | | | | | | | | | | 246 | | | | | | | | | | | 0 | | | | | | | | | | | --- | | | | | | | | | | | K.YLKPSVAGFLLQK.E | | | | | | | | | | | | |  | | | 1552.6096 | | | | 1551.6023 | | | | | | | | | | 1551.8872 | | | | | | | | | | | -183.57 | | | | | | | | | | | 330 | | | | | | | | | | | - | | | | | | | | | | | 344 | | | | | | | | | | | 0 | | | | | | | | | | | --- | | | | | | | | | | | K.GVNLLLPSDVVIADK.F | | | | | | | | | | | | |  | | | 1573.5763 | | | | 1572.5690 | | | | | | | | | | 1572.8359 | | | | | | | | | | | -169.69 | | | | | | | | | | | 418 | | | | | | | | | | | - | | | | | | | | | | | 434 | | | | | | | | | | | 0 | | | | | | | | | | | --- | | | | | | | | | | | K.GVTTIIGGGDSVAAVEK.V | | | | | | | | | | | | |  | | | 1778.7515 | | | | 1777.7442 | | | | | | | | | | 1778.0302 | | | | | | | | | | | -160.82 | | | | | | | | | | | 174 | | | | | | | | | | | - | | | | | | | | | | | 190 | | | | | | | | | | | 0 | | | | | | | | | | | --- | | | | | | | | | | | K.LVASLPEGGVLLLENVR.F | | | | | | | | | | | | |  | | | 1778.7515 | | | | 1777.7442 | | | | | | | | | | 1778.0302 | | | | | | | | | | | -160.82 | | | | | | | | | | | 174 | | | | | | | | | | | - | | | | | | | | | | | 190 | | | | | | | | | | | 0 | | | | | | | | | | | 95 | | | | | | | | | | | K.LVASLPEGGVLLLENVR.F | | | | | | | | | | | | |  | | | 1933.6660 | | | | 1932.6587 | | | | | | | | | | 1932.9694 | | | | | | | | | | | -160.70 | | | | | | | | | | | 206 | | | | | | | | | | | - | | | | | | | | | | | 223 | | | | | | | | | | | 0 | | | | | | | | | | | --- | | | | | | | | | | | K.LASLADLYVNDAFGTAHR.A | | | | | | | | | | | | |  | | | 1933.6660 | | | | 1932.6587 | | | | | | | | | | 1932.9694 | | | | | | | | | | | -160.70 | | | | | | | | | | | 206 | | | | | | | | | | | - | | | | | | | | | | | 223 | | | | | | | | | | | 0 | | | | | | | | | | | 87 | | | | | | | | | | | K.LASLADLYVNDAFGTAHR.A | | | | | | | | | | | | |  | | | 2028.6115 | | | | 2027.6042 | | | | | | | | | | 2027.9396 | | | | | | | | | | | -165.37 | | | | | | | | | | | 92 | | | | | | | | | | | - | | | | | | | | | | | 109 | | | | | | | | | | | 0 | | | | | | | | | | | --- | | | | | | | | | | | R.ADLNVPLDDNQNITDDTR.I | | | | | | | | | | | | |  | | | 2028.6115 | | | | 2027.6042 | | | | | | | | | | 2027.9396 | | | | | | | | | | | -165.37 | | | | | | | | | | | 92 | | | | | | | | | | | - | | | | | | | | | | | 109 | | | | | | | | | | | 0 | | | | | | | | | | | 91 | | | | | | | | | | | R.ADLNVPLDDNQNITDDTR.I | | | | | | | | | | | | |  | | | 2061.7097 | | | | 2060.7024 | | | | | | | | | | 2061.0643 | | | | | | | | | | | -175.59 | | | | | | | | | | | 205 | | | | | | | | | | | - | | | | | | | | | | | 223 | | | | | | | | | | | 1 | | | | | | | | | | | --- | | | | | | | | | | | K.KLASLADLYVNDAFGTAHR.A | | | | | | | | | | | | |  | | | 2238.7820 | | | | 2237.7747 | | | | | | | | | | 2237.1402 | | | | | | | | | | | 284 | | | | | | | | | | | 353 | | | | | | | | | | | - | | | | | | | | | | | 374 | | | | | | | | | | | 0 | | | | | | | | | | | --- | | | | | | | | | | | K.VVPATAIPDGWMGLDIGPDSVK.T | | | | | | | | | | | | |  | | | 2269.7327 | | | | 2268.7254 | | | | | | | | | | 2268.1784 | | | | | | | | | | | 241 | | | | | | | | | | | 435 | | | | | | | | | | | - | | | | | | | | | | | 458 | | | | | | | | | | | 0 | | | | | | | | | | | --- | | | | | | | | | | | K.VGVAGVMSHISTGGGASLELLEGK.E | | | | | | | | | | | | |  | | | 2284.7732 | | | | 2283.7659 | | | | | | | | | | 2284.1733 | | | | | | | | | | | -178.35 | | | | | | | | | | | 435 | | | | | | | | | | | - | | | | | | | | | | | 458 | | | | | | | | | | | 0 | | | | | | | | | | | --- | | | | | | | | | | | K.VGVAGVMSHISTGGGASLELLEGK.E + Oxidation (M) | | | | | | | | | | | | |  | | | 2557.9448 | | | | 2556.9375 | | | | | | | | | | 2557.3850 | | | | | | | | | | | -174.99 | | | | | | | | | | | 301 | | | | | | | | | | | - | | | | | | | | | | | 325 | | | | | | | | | | | 1 | | | | | | | | | | | --- | | | | | | | | | | | K.AQGISVGSSLVEEDKLDLATTLLAK.A | | | | | | | | | | | | |  | | | No match to: 700.2723, 713.2905, 716.2158, 730.2371, 742.2377, 768.3666, 771.2604, 773.2599, 799.2558, 801.3159, 842.3572, 857.2282, 858.3104, 868.3844, 870.2989, 882.3672, 981.4207, 1021.3755, 1051.5144, 1059.4015, 1079.4551, 1081.3666, 1099.3456, 1110.3698, 1147.3813, 1148.3657, 1151.4447, 1155.3759, 1155.3759, 1162.4056, 1177.3779, 1304.4899, 1307.3948, 1309.5067, 1322.4607, 1396.5345, 1396.5345, 1415.4812, 1435.4510, 1435.4510, 1437.4796, 1449.4994, 1465.5421, 1484.4790, 1496.5093, 1591.6449, 1639.5537, 1656.5411, 1664.5327, 1673.6028, 1673.6028, 1693.6245, 1695.5662, 1703.5881, 1711.5649, 1759.5525, 1771.5282, 1773.5377, 1777.6545, 1783.5248, 1784.5211, 1785.5288, 1800.7300, 1810.5968, 1823.6464, 1825.6426, 1851.6456, 1855.6687, 1916.6355, 1961.6729, 1972.6794, 2002.6864, 2011.6344, 2027.6503, 2051.5969, 2054.6667, 2088.7168, 2152.7104, 2169.7422, 2293.7678, 2490.7688, 2494.7297, 2507.7700, 2519.7764, 2535.7556 | | | | | | | | | | | | | | | | | | | | | | | | | | | | | | | | | | | | | | | | | | | | | | | | | | | | | | | | | | | | | | | | | | | | | | | | | | | | | | | | | | | | | | | | | | | | | | | | | | | |  | | | | | | | **S29** [**orange1.1t03280.1**](http://zhangyang-pc/mascot/cgi/protein_view.pl?file=../data/20140118/F012103.dat&hit=2) **Mass: 49415 Score: 346 Expect: 1.1e-030 Matches: 13** | | | | | | | | | | | | | | | | | | | | | | | | | | | | | | | | | | | | | | | | | | | | | | | | | | | | | | | | | | | | | | | | | | | | | | | | | | | | | | | | | | | | | | | | | | | | | | | | | | | |  | | | | | | | Observed | | | | | | | | | | Mr(expt) | | | | | | | | | | Mr(calc) | | | | | | | | | | | ppm | | | | | | | | | | | Start | | | | | | | | | | | |  | | | | | | | | | | End | | | | | | | | | | | Miss | | | | | | | | | | | Ions | | | | | | | | | | | Peptide | | | | | | |  | | | 999.4426 | | | | | | | | | | 998.4354 | | | | | | | | | | 998.5913 | | | | | | | | | | | -156.16 | | | | | | | | | | | 143 | | | | | | | | | | | | - | | | | | | | | | | 151 | | | | | | | | | | | 0 | | | | | | | | | | | --- | | | | | | | | | | | K.FSLAPLVPR.L | | | | | | |  | | | 999.4426 | | | | | | | | | | 998.4354 | | | | | | | | | | 998.5913 | | | | | | | | | | | -156.16 | | | | | | | | | | | 143 | | | | | | | | | | | | - | | | | | | | | | | 151 | | | | | | | | | | | 0 | | | | | | | | | | | 68 | | | | | | | | | | | K.FSLAPLVPR.L | | | | | | |  | | | 1102.4570 | | | | | | | | | | 1101.4497 | | | | | | | | | | 1101.6295 | | | | | | | | | | | -163.16 | | | | | | | | | | | 260 | | | | | | | | | | | | - | | | | | | | | | | 270 | | | | | | | | | | | 0 | | | | | | | | | | | --- | | | | | | | | | | | K.RPFAAIVGGSK.V | | | | | | |  | | | 1377.5087 | | | | | | | | | | 1376.5014 | | | | | | | | | | 1376.7187 | | | | | | | | | | | -157.85 | | | | | | | | | | | 247 | | | | | | | | | | | | - | | | | | | | | | | 259 | | | | | | | | | | | 0 | | | | | | | | | | | --- | | | | | | | | | | | K.ELDYLVGAVSSPK.R | | | | | | |  | | | 1463.5559 | | | | | | | | | | 1462.5486 | | | | | | | | | | 1462.8548 | | | | | | | | | | | -209.28 | | | | | | | | | | | 234 | | | | | | | | | | | | - | | | | | | | | | | 246 | | | | | | | | | | | 0 | | | | | | | | | | | --- | | | | | | | | | | | K.YLKPSVAGFLLQK.E | | | | | | |  | | | 1552.5856 | | | | | | | | | | 1551.5783 | | | | | | | | | | 1551.8872 | | | | | | | | | | | -199.04 | | | | | | | | | | | 330 | | | | | | | | | | | | - | | | | | | | | | | 344 | | | | | | | | | | | 0 | | | | | | | | | | | --- | | | | | | | | | | | K.GVNLLLPSDVVIADK.F | | | | | | |  | | | 1778.7708 | | | | | | | | | | 1777.7635 | | | | | | | | | | 1778.0302 | | | | | | | | | | | -149.97 | | | | | | | | | | | 174 | | | | | | | | | | | | - | | | | | | | | | | 190 | | | | | | | | | | | 0 | | | | | | | | | | | --- | | | | | | | | | | | K.LVASLPEGGVLLLENVR.F | | | | | | |  | | | 1778.7708 | | | | | | | | | | 1777.7635 | | | | | | | | | | 1778.0302 | | | | | | | | | | | -149.97 | | | | | | | | | | | 174 | | | | | | | | | | | | - | | | | | | | | | | 190 | | | | | | | | | | | 0 | | | | | | | | | | | 92 | | | | | | | | | | | K.LVASLPEGGVLLLENVR.F | | | | | | |  | | | 1933.6887 | | | | | | | | | | 1932.6814 | | | | | | | | | | 1932.9694 | | | | | | | | | | | -148.96 | | | | | | | | | | | 206 | | | | | | | | | | | | - | | | | | | | | | | 223 | | | | | | | | | | | 0 | | | | | | | | | | | --- | | | | | | | | | | | K.LASLADLYVNDAFGTAHR.A | | | | | | |  | | | 1933.6887 | | | | | | | | | | 1932.6814 | | | | | | | | | | 1932.9694 | | | | | | | | | | | -148.96 | | | | | | | | | | | 206 | | | | | | | | | | | | - | | | | | | | | | | 223 | | | | | | | | | | | 0 | | | | | | | | | | | 78 | | | | | | | | | | | K.LASLADLYVNDAFGTAHR.A | | | | | | |  | | | 2028.6360 | | | | | | | | | | 2027.6287 | | | | | | | | | | 2027.9396 | | | | | | | | | | | -153.29 | | | | | | | | | | | 92 | | | | | | | | | | | | - | | | | | | | | | | 109 | | | | | | | | | | | 0 | | | | | | | | | | | --- | | | | | | | | | | | R.ADLNVPLDDNQNITDDTR.I | | | | | | |  | | | 2028.6360 | | | | | | | | | | 2027.6287 | | | | | | | | | | 2027.9396 | | | | | | | | | | | -153.29 | | | | | | | | | | | 92 | | | | | | | | | | | | - | | | | | | | | | | 109 | | | | | | | | | | | 0 | | | | | | | | | | | 91 | | | | | | | | | | | R.ADLNVPLDDNQNITDDTR.I | | | | | | |  | | | 2238.7964 | | | | | | | | | | 2237.7891 | | | | | | | | | | 2237.1402 | | | | | | | | | | | 290 | | | | | | | | | | | 353 | | | | | | | | | | | | - | | | | | | | | | | 374 | | | | | | | | | | | 0 | | | | | | | | | | | --- | | | | | | | | | | | K.VVPATAIPDGWMGLDIGPDSVK.T | | | | | | |  | | | No match to: 713.2899, 714.3124, 716.2619, 731.2869, 750.3190, 753.2657, 758.2262, 768.3987, 819.3168, 842.3740, 850.3679, 868.3854, 870.4042, 878.2454, 883.3035, 884.2574, 895.2230, 896.2431, 896.2431, 909.3221, 912.2499, 914.2615, 943.4138, 962.3082, 981.3210, 981.3210, 1010.3921, 1021.3592, 1059.4052, 1067.4146, 1143.4248, 1157.4202, 1159.4558, 1164.3198, 1174.4875, 1174.4875, 1187.4562, 1191.5146, 1241.4198, 1249.4242, 1250.4496, 1250.4496, 1261.5222, 1296.4874, 1311.4458, 1370.5013, 1422.5684, 1425.5920, 1438.5165, 1460.5123, 1465.5381, 1475.5468, 1479.5376, 1479.5376, 1483.5370, 1515.4181, 1530.4922, 1531.5857, 1541.5809, 1547.5059, 1575.6608, 1592.6028, 1601.5951, 1618.5508, 1630.5574, 1632.6069, 1668.5306, 1690.4972, 1697.6638, 1725.6666, 1741.6285, 1759.5934, 1761.6454, 1825.6255, 1843.5851, 1843.5851, 1859.6091, 1870.7323, 1938.6283, 1943.7234, 1990.6647, 2007.6824, 2023.6776, 2220.8110, 2245.8079, 2248.7109, 2251.8455, 2270.7161, 2271.7766, 2274.7700, 2562.7961, 2638.8650, 2652.8245, 2783.8135, 2795.8052, 2910.0046, 3346.1436 | | | | | | | | | | | | | | | | | | | | | | | | | | | | | | | | | | | | | | | | | | | | | | | | | | | | | | | | | | | | | | | | | | | | | | | | | | | | | | | | | | | | | | | | | | | | | | | | | | | | |  | | | | | | **S23** [**Cs3g01420.1**](http://zhangyang-pc/mascot/cgi/protein_view.pl?file=../data/20140118/F012154.dat&hit=3) **Mass: 32572 Score: 415 Expect: 1.4e-037 Matches: 14** | | | | | | | | | | | | | | | | | | | | | | | | | | | | | | | | | | | | | | | | | | | | | | | | | | | | | | | | | | | | | | | | | | | | | | | | | | | | | | | | | | | | | | | | | | | | | | | | | | | | |  | | | | | | Observed | | | | | | | | | | Mr(expt) | | | | | | | | | | Mr(calc) | | | | | | | | | | | ppm | | | | | | | | | | | Start | | | | | | | | | | | |  | | | | | | | | | | End | | | | | | | | | | | Miss | | | | | | | | | | | Ions | | | | | | | | | | | Peptide | | | | | | |  | | | 817.2971 | | | | | | | | | | 816.2898 | | | | | | | | | | 816.4527 | | | | | | | | | | | -199.52 | | | | | | | | | | | 182 | | | | | | | | | | | | - | | | | | | | | | | 188 | | | | | | | | | | | 0 | | | | | | | | | | | --- | | | | | | | | | | | K.VLMDALR.V | | | | | | |  | | | 833.2814 | | | | | | | | | | 832.2742 | | | | | | | | | | 832.4477 | | | | | | | | | | | -208.43 | | | | | | | | | | | 182 | | | | | | | | | | | | - | | | | | | | | | | 188 | | | | | | | | | | | 0 | | | | | | | | | | | --- | | | | | | | | | | | K.VLMDALR.V + Oxidation (M) | | | | | | |  | | | 834.2717 | | | | | | | | | | 833.2644 | | | | | | | | | | 833.4395 | | | | | | | | | | | -210.15 | | | | | | | | | | | 237 | | | | | | | | | | | | - | | | | | | | | | | 243 | | | | | | | | | | | 0 | | | | | | | | | | | --- | | | | | | | | | | | K.GFELVNR.E | | | | | | |  | | | 949.3653 | | | | | | | | | | 948.3580 | | | | | | | | | | 948.4764 | | | | | | | | | | | -124.77 | | | | | | | | | | | 286 | | | | | | | | | | | | - | | | | | | | | | | 294 | | | | | | | | | | | 0 | | | | | | | | | | | --- | | | | | | | | | | | R.ILNLSSESS.- | | | | | | |  | | | 962.3483 | | | | | | | | | | 961.3410 | | | | | | | | | | 961.5345 | | | | | | | | | | | -201.25 | | | | | | | | | | | 236 | | | | | | | | | | | | - | | | | | | | | | | 243 | | | | | | | | | | | 1 | | | | | | | | | | | --- | | | | | | | | | | | K.KGFELVNR.E | | | | | | |  | | | 1043.4008 | | | | | | | | | | 1042.3935 | | | | | | | | | | 1042.6386 | | | | | | | | | | | -235.09 | | | | | | | | | | | 67 | | | | | | | | | | | | - | | | | | | | | | | 77 | | | | | | | | | | | 0 | | | | | | | | | | | --- | | | | | | | | | | | K.SVSVILLAGGK.G | | | | | | |  | | | 1137.3348 | | | | | | | | | | 1136.3275 | | | | | | | | | | 1136.5462 | | | | | | | | | | | -192.39 | | | | | | | | | | | 204 | | | | | | | | | | | | - | | | | | | | | | | 213 | | | | | | | | | | | 0 | | | | | | | | | | | --- | | | | | | | | | | | K.EANSESFVVR.T | | | | | | |  | | | 1137.3348 | | | | | | | | | | 1136.3275 | | | | | | | | | | 1136.5462 | | | | | | | | | | | -192.39 | | | | | | | | | | | 204 | | | | | | | | | | | | - | | | | | | | | | | 213 | | | | | | | | | | | 0 | | | | | | | | | | | 98 | | | | | | | | | | | K.EANSESFVVR.T | | | | | | |  | | | 1342.4604 | | | | | | | | | | 1341.4531 | | | | | | | | | | 1341.7140 | | | | | | | | | | | -194.43 | | | | | | | | | | | 274 | | | | | | | | | | | | - | | | | | | | | | | 285 | | | | | | | | | | | 0 | | | | | | | | | | | --- | | | | | | | | | | | K.VTTPDDLLIAER.I | | | | | | |  | | | 1342.4604 | | | | | | | | | | 1341.4531 | | | | | | | | | | 1341.7140 | | | | | | | | | | | -194.43 | | | | | | | | | | | 274 | | | | | | | | | | | | - | | | | | | | | | | 285 | | | | | | | | | | | 0 | | | | | | | | | | | 121 | | | | | | | | | | | K.VTTPDDLLIAER.I | | | | | | |  | | | 1621.4980 | | | | | | | | | | 1620.4907 | | | | | | | | | | 1620.8148 | | | | | | | | | | | -199.92 | | | | | | | | | | | 260 | | | | | | | | | | | | - | | | | | | | | | | 273 | | | | | | | | | | | 0 | | | | | | | | | | | --- | | | | | | | | | | | K.HPVYITEGSYTNIK.V | | | | | | |  | | | 1621.4980 | | | | | | | | | | 1620.4907 | | | | | | | | | | 1620.8148 | | | | | | | | | | | -199.92 | | | | | | | | | | | 260 | | | | | | | | | | | | - | | | | | | | | | | 273 | | | | | | | | | | | 0 | | | | | | | | | | | 85 | | | | | | | | | | | K.HPVYITEGSYTNIK.V | | | | | | |  | | | 1782.5568 | | | | | | | | | | 1781.5495 | | | | | | | | | | 1781.9047 | | | | | | | | | | | -199.33 | | | | | | | | | | | 244 | | | | | | | | | | | | - | | | | | | | | | | 259 | | | | | | | | | | | 0 | | | | | | | | | | | --- | | | | | | | | | | | R.EGLEVTDDVSIVEHLK.H | | | | | | |  | | | 1782.5568 | | | | | | | | | | 1781.5495 | | | | | | | | | | 1781.9047 | | | | | | | | | | | -199.33 | | | | | | | | | | | 244 | | | | | | | | | | | | - | | | | | | | | | | 259 | | | | | | | | | | | 0 | | | | | | | | | | | 83 | | | | | | | | | | | R.EGLEVTDDVSIVEHLK.H | | | | | | |  | | | No match to: 703.8342, 713.2787, 716.2302, 729.2648, 734.3243, 750.3431, 757.3049, 768.3531, 773.2748, 801.3202, 842.3377, 845.3445, 848.3097, 861.3138, 861.3138, 868.3452, 882.3616, 889.3316, 892.8389, 905.3336, 908.8161, 993.3796, 1037.3612, 1051.4611, 1059.3613, 1071.4490, 1071.4490, 1087.3003, 1090.3683, 1107.3462, 1119.3292, 1128.3308, 1151.4764, 1165.3849, 1172.4415, 1175.3083, 1179.3630, 1263.4227, 1315.3746, 1326.3958, 1331.3789, 1361.4368, 1364.4125, 1378.4103, 1380.4335, 1428.3798, 1434.5599, 1479.4642, 1481.5189, 1490.5391, 1517.3992, 1603.5272, 1603.5272, 1619.5143, 1633.4813, 1643.4874, 1659.4600, 1661.4908, 1676.5619, 1703.5402, 1716.5266, 1739.5227, 1761.4791, 1796.5941, 1820.5321, 1843.5526, 1873.5927, 1890.6177, 1907.6107, 1921.6663, 1993.6294, 2002.7035, 2007.6748, 2038.6123, 2059.7007, 2063.7542, 2071.6924, 2071.6924, 2075.6604, 2087.6943, 2109.6638, 2220.7000, 2238.7117, 2238.7117, 2245.7771, 2276.6565, 2359.7922, 2759.7595, 2759.7595, 2776.7485, 2848.7188, 2850.7439, 2866.7454, 2883.7126, 2913.9348, 3346.0356 | | | | | | | | | | | | | | | | | | | | | | | | | | | | | | | | | | | | | | | | | | | | | | | | | | | | | | | | | | | | | | | | | | | | | | | | | | | | | | | | | | | | | | | | | | | | | | | | | | | | | |  | | | | | **S24** [**Cs5g29390.1**](http://zhangyang-pc/mascot/cgi/protein_view.pl?file=../data/20140118/F012085.dat&hit=1) **Mass: 45232 Score: 286 Expect: 1.1e-024 Matches: 27** | | | | | | | | | | | | | | | | | | | | | | | | | | | | | | | | | | | | | | | | | | | | | | | | | | | | | | | | | | | | | | | | | | | | | | | | | | | | | | | | | | | | | | | | | | | | | | | | | | | | | |  | | | | | Observed | | | Mr(expt) | | | | | | | | | | Mr(calc) | | | | | | | | | | | ppm | | | | | | | | | | | Start | | | | | | | | | | |  | | | | | | | | | | | End | | | | | | | | | | | Miss | | | | | | | | | | | Ions | | | | | | | | | | | Peptide | | | | | | | | | | | | | |  | | | 748.2971 | | | 747.2898 | | | | | | | | | | 747.4279 | | | | | | | | | | | -184.79 | | | | | | | | | | | 263 | | | | | | | | | | | - | | | | | | | | | | | 268 | | | | | | | | | | | 0 | | | | | | | | | | | --- | | | | | | | | | | | K.EIFALR.D | | | | | | | | | | | | | |  | | | 790.2886 | | | 789.2814 | | | | | | | | | | 789.4385 | | | | | | | | | | | -199.02 | | | | | | | | | | | 42 | | | | | | | | | | | - | | | | | | | | | | | 48 | | | | | | | | | | | 0 | | | | | | | | | | | --- | | | | | | | | | | | K.YGINVPK.G | | | | | | | | | | | | | |  | | | 801.3146 | | | 800.3074 | | | | | | | | | | 800.4504 | | | | | | | | | | | -178.74 | | | | | | | | | | | 76 | | | | | | | | | | | - | | | | | | | | | | | 83 | | | | | | | | | | | 0 | | | | | | | | | | | --- | | | | | | | | | | | K.SQILAGGR.G | | | | | | | | | | | | | |  | | | 880.2959 | | | 879.2886 | | | | | | | | | | 879.4524 | | | | | | | | | | | -186.23 | | | | | | | | | | | 175 | | | | | | | | | | | - | | | | | | | | | | | 181 | | | | | | | | | | | 0 | | | | | | | | | | | --- | | | | | | | | | | | K.YPNMIVK.V + Oxidation (M) | | | | | | | | | | | | | |  | | | 916.3159 | | | 915.3086 | | | | | | | | | | 915.4661 | | | | | | | | | | | -172.04 | | | | | | | | | | | 210 | | | | | | | | | | | - | | | | | | | | | | | 217 | | | | | | | | | | | 0 | | | | | | | | | | | --- | | | | | | | | | | | R.NDAIEQVK.K | | | | | | | | | | | | | |  | | | 957.2755 | | | 956.2682 | | | | | | | | | | 956.4199 | | | | | | | | | | | -158.62 | | | | | | | | | | | 269 | | | | | | | | | | | - | | | | | | | | | | | 276 | | | | | | | | | | | 0 | | | | | | | | | | | --- | | | | | | | | | | | R.DPTQEDPR.E | | | | | | | | | | | | | |  | | | 1018.4412 | | | 1017.4339 | | | | | | | | | | 1017.5892 | | | | | | | | | | | -152.64 | | | | | | | | | | | 111 | | | | | | | | | | | - | | | | | | | | | | | 119 | | | | | | | | | | | 0 | | | | | | | | | | | --- | | | | | | | | | | | K.MLGQILVTK.Q + Oxidation (M) | | | | | | | | | | | | | |  | | | 1059.3945 | | | 1058.3872 | | | | | | | | | | 1058.5794 | | | | | | | | | | | -181.55 | | | | | | | | | | | 153 | | | | | | | | | | | - | | | | | | | | | | | 163 | | | | | | | | | | | 0 | | | | | | | | | | | --- | | | | | | | | | | | K.TAGPIIIGCSK.G | | | | | | | | | | | | | |  | | | 1059.3945 | | | 1058.3872 | | | | | | | | | | 1058.5794 | | | | | | | | | | | -181.55 | | | | | | | | | | | 153 | | | | | | | | | | | - | | | | | | | | | | | 163 | | | | | | | | | | | 0 | | | | | | | | | | | --- | | | | | | | | | | | K.TAGPIIIGCSK.G | | | | | | | | | | | | | |  | | | 1117.4095 | | | 1116.4022 | | | | | | | | | | 1116.5662 | | | | | | | | | | | -146.89 | | | | | | | | | | | 101 | | | | | | | | | | | - | | | | | | | | | | | 110 | | | | | | | | | | | 1 | | | | | | | | | | | --- | | | | | | | | | | | K.KEEVEDLAGK.M | | | | | | | | | | | | | |  | | | 1119.3896 | | | 1118.3823 | | | | | | | | | | 1118.5455 | | | | | | | | | | | -145.90 | | | | | | | | | | | 164 | | | | | | | | | | | - | | | | | | | | | | | 174 | | | | | | | | | | | 0 | | | | | | | | | | | --- | | | | | | | | | | | K.GGTSIEDLAEK.Y | | | | | | | | | | | | | |  | | | 1182.3640 | | | 1181.3567 | | | | | | | | | | 1181.5465 | | | | | | | | | | | -160.64 | | | | | | | | | | | 251 | | | | | | | | | | | - | | | | | | | | | | | 260 | | | | | | | | | | | 0 | | | | | | | | | | | --- | | | | | | | | | | | K.LNFDDNAAFR.Q | | | | | | | | | | | | | |  | | | 1182.3640 | | | 1181.3567 | | | | | | | | | | 1181.5465 | | | | | | | | | | | -160.64 | | | | | | | | | | | 251 | | | | | | | | | | | - | | | | | | | | | | | 260 | | | | | | | | | | | 0 | | | | | | | | | | | 78 | | | | | | | | | | | K.LNFDDNAAFR.Q | | | | | | | | | | | | | |  | | | 1215.4634 | | | 1214.4561 | | | | | | | | | | 1214.6870 | | | | | | | | | | | -190.10 | | | | | | | | | | | 49 | | | | | | | | | | | - | | | | | | | | | | | 60 | | | | | | | | | | | 1 | | | | | | | | | | | --- | | | | | | | | | | | K.GLAVASVDEVKK.A | | | | | | | | | | | | | |  | | | 1216.4633 | | | 1215.4560 | | | | | | | | | | 1215.6208 | | | | | | | | | | | -135.51 | | | | | | | | | | | 384 | | | | | | | | | | | - | | | | | | | | | | | 394 | | | | | | | | | | | 1 | | | | | | | | | | | --- | | | | | | | | | | | R.LEGTNVDQGKR.I | | | | | | | | | | | | | |  | | | 1291.5233 | | | 1290.5160 | | | | | | | | | | 1290.7370 | | | | | | | | | | | -171.18 | | | | | | | | | | | 348 | | | | | | | | | | | - | | | | | | | | | | | 359 | | | | | | | | | | | 0 | | | | | | | | | | | --- | | | | | | | | | | | K.AILVNIFGGIMK.C + Oxidation (M) | | | | | | | | | | | | | |  | | | 1357.4930 | | | 1356.4857 | | | | | | | | | | 1356.6997 | | | | | | | | | | | -157.73 | | | | | | | | | | | 206 | | | | | | | | | | | - | | | | | | | | | | | 217 | | | | | | | | | | | 1 | | | | | | | | | | | --- | | | | | | | | | | | K.VADRNDAIEQVK.K | | | | | | | | | | | | | |  | | | 1632.5405 | | | 1631.5332 | | | | | | | | | | 1631.7977 | | | | | | | | | | | -162.08 | | | | | | | | | | | 28 | | | | | | | | | | | - | | | | | | | | | | | 41 | | | | | | | | | | | 0 | | | | | | | | | | | --- | | | | | | | | | | | R.LNIHEYQGAELMAK.Y + Oxidation (M) | | | | | | | | | | | | | |  | | | 1686.5709 | | | 1685.5636 | | | | | | | | | | 1685.8373 | | | | | | | | | | | -162.33 | | | | | | | | | | | 263 | | | | | | | | | | | - | | | | | | | | | | | 276 | | | | | | | | | | | 1 | | | | | | | | | | | --- | | | | | | | | | | | K.EIFALRDPTQEDPR.E | | | | | | | | | | | | | |  | | | 1703.5763 | | | 1702.5690 | | | | | | | | | | 1702.8414 | | | | | | | | | | | -159.95 | | | | | | | | | | | 182 | | | | | | | | | | | - | | | | | | | | | | | 197 | | | | | | | | | | | 0 | | | | | | | | | | | --- | | | | | | | | | | | K.VPIDVFNGITDEDAAK.V | | | | | | | | | | | | | |  | | | 1830.6157 | | | 1829.6084 | | | | | | | | | | 1829.9055 | | | | | | | | | | | -162.37 | | | | | | | | | | | 137 | | | | | | | | | | | - | | | | | | | | | | | 151 | | | | | | | | | | | 0 | | | | | | | | | | | --- | | | | | | | | | | | K.LSLVNEMYFAIMLDR.K + Oxidation (M) | | | | | | | | | | | | | |  | | | 1846.6019 | | | 1845.5946 | | | | | | | | | | 1845.9005 | | | | | | | | | | | -165.69 | | | | | | | | | | | 137 | | | | | | | | | | | - | | | | | | | | | | | 151 | | | | | | | | | | | 0 | | | | | | | | | | | --- | | | | | | | | | | | K.LSLVNEMYFAIMLDR.K + 2 Oxidation (M) | | | | | | | | | | | | | |  | | | 1846.6019 | | | 1845.5946 | | | | | | | | | | 1845.9005 | | | | | | | | | | | -165.69 | | | | | | | | | | | 137 | | | | | | | | | | | - | | | | | | | | | | | 151 | | | | | | | | | | | 0 | | | | | | | | | | | 47 | | | | | | | | | | | K.LSLVNEMYFAIMLDR.K + 2 Oxidation (M) | | | | | | | | | | | | | |  | | | 1974.6754 | | | 1973.6681 | | | | | | | | | | 1973.9954 | | | | | | | | | | | -165.80 | | | | | | | | | | | 137 | | | | | | | | | | | - | | | | | | | | | | | 152 | | | | | | | | | | | 1 | | | | | | | | | | | --- | | | | | | | | | | | K.LSLVNEMYFAIMLDRK.T + 2 Oxidation (M) | | | | | | | | | | | | | |  | | | 1974.6754 | | | 1973.6681 | | | | | | | | | | 1973.9954 | | | | | | | | | | | -165.80 | | | | | | | | | | | 137 | | | | | | | | | | | - | | | | | | | | | | | 152 | | | | | | | | | | | 1 | | | | | | | | | | | --- | | | | | | | | | | | K.LSLVNEMYFAIMLDRK.T + 2 Oxidation (M) | | | | | | | | | | | | | |  | | | 2613.8391 | | | 2612.8318 | | | | | | | | | | 2613.2823 | | | | | | | | | | | -172.40 | | | | | | | | | | | 313 | | | | | | | | | | | - | | | | | | | | | | | 338 | | | | | | | | | | | 0 | | | | | | | | | | | --- | | | | | | | | | | | K.LHGGTPANFLDVGGNASEGQVVEAFK.I | | | | | | | | | | | | | |  | | | 2613.8391 | | | 2612.8318 | | | | | | | | | | 2613.2823 | | | | | | | | | | | -172.40 | | | | | | | | | | | 313 | | | | | | | | | | | - | | | | | | | | | | | 338 | | | | | | | | | | | 0 | | | | | | | | | | | 69 | | | | | | | | | | | K.LHGGTPANFLDVGGNASEGQVVEAFK.I | | | | | | | | | | | | | |  | | | No match to: 700.1382, 709.2230, 712.1193, 714.1253, 734.3479, 737.2517, 752.2277, 768.3833, 770.2588, 804.1340, 818.1315, 830.1511, 832.1487, 834.1597, 842.3599, 848.1503, 850.3832, 854.1450, 854.1450, 860.9243, 860.9243, 862.1394, 866.1298, 868.3953, 870.1229, 876.1254, 876.8950, 876.8950, 882.4020, 886.1116, 905.3300, 944.3034, 956.2967, 968.3855, 982.4017, 996.4163, 1003.1720, 1025.3945, 1042.3499, 1051.5276, 1061.3923, 1139.3887, 1141.3724, 1145.3800, 1151.5309, 1165.5286, 1167.4272, 1179.3943, 1204.3450, 1251.4934, 1292.4940, 1313.4950, 1315.4303, 1329.4547, 1331.4175, 1335.5283, 1345.4188, 1391.5122, 1434.6003, 1465.5009, 1566.6077, 1693.5537, 1781.6237, 1782.0181, 1782.6206, 1862.5969, 1882.6702, 1882.6702, 1910.6946, 1993.6650, 2088.8342, 2088.8342, 2220.7522, 2231.9131, 2238.7739, 2248.9287, 2507.9063, 2567.8040, 2634.8120, 2635.8140, 2694.8196, 2810.8579, 3346.0967 | | | | | | | | | | | | | | | | | | | | | | | | | | | | | | | | | | | | | | | | | | | | | | | | | | | | | | | | | | | | | | | | | | | | | | | | | | | | | | | | | | | | | | | | | | | | | | | | | | | | | |  | | | | | **S28** [**Cs1g17930.1**](http://zhangyang-pc/mascot/cgi/protein_view.pl?file=../data/20140118/F012173.dat&hit=4) **Mass: 59332 Score: 390 Expect: 4.4e-035 Matches: 23** | | | | | | | | | | | | | | | | | | | | | | | | | | | | | | | | | | | | | | | | | | | | | | | | | | | | | | | | | | | | | | | | | | | | | | | | | | | | | | | | | | | | | | | | | | | | | | | | | | | | | |  | | | | | Observed | | | Mr(expt) | | | | | | | | | | Mr(calc) | | | | | | | | | | | ppm | | | | | | | | | | | Start | | | | | | | | | | |  | | | | | | | | | | | End | | | | | | | | | | | Miss | | | | | | | | | | | Ions | | | | | | | | | | | Peptide | | | | | | | | | | | | | |  | | | 860.3392 | | | 859.3320 | | | | | | | | | | 859.4916 | | | | | | | | | | | -185.72 | | | | | | | | | | | 255 | | | | | | | | | | | - | | | | | | | | | | | 262 | | | | | | | | | | | 0 | | | | | | | | | | | --- | | | | | | | | | | | R.LFASPVAR.N | | | | | | | | | | | | | |  | | | 860.3392 | | | 859.3320 | | | | | | | | | | 859.4916 | | | | | | | | | | | -185.72 | | | | | | | | | | | 255 | | | | | | | | | | | - | | | | | | | | | | | 262 | | | | | | | | | | | 0 | | | | | | | | | | | 37 | | | | | | | | | | | R.LFASPVAR.N | | | | | | | | | | | | | |  | | | 945.3231 | | | 944.3158 | | | | | | | | | | 944.4967 | | | | | | | | | | | -191.57 | | | | | | | | | | | 220 | | | | | | | | | | | - | | | | | | | | | | | 228 | | | | | | | | | | | 0 | | | | | | | | | | | --- | | | | | | | | | | | K.EPSPPPPPK.Q | | | | | | | | | | | | | |  | | | 1074.3925 | | | 1073.3852 | | | | | | | | | | 1073.5717 | | | | | | | | | | | -173.67 | | | | | | | | | | | 432 | | | | | | | | | | | - | | | | | | | | | | | 441 | | | | | | | | | | | 0 | | | | | | | | | | | --- | | | | | | | | | | | K.GLSTIAEEVR.Q | | | | | | | | | | | | | |  | | | 1074.3925 | | | 1073.3852 | | | | | | | | | | 1073.5717 | | | | | | | | | | | -173.67 | | | | | | | | | | | 432 | | | | | | | | | | | - | | | | | | | | | | | 441 | | | | | | | | | | | 0 | | | | | | | | | | | 57 | | | | | | | | | | | K.GLSTIAEEVR.Q | | | | | | | | | | | | | |  | | | 1152.3527 | | | 1151.3454 | | | | | | | | | | 1151.5458 | | | | | | | | | | | -174.04 | | | | | | | | | | | 287 | | | | | | | | | | | - | | | | | | | | | | | 296 | | | | | | | | | | | 0 | | | | | | | | | | | --- | | | | | | | | | | | K.ADIEDYLASR.G | | | | | | | | | | | | | |  | | | 1152.3527 | | | 1151.3454 | | | | | | | | | | 1151.5458 | | | | | | | | | | | -174.04 | | | | | | | | | | | 287 | | | | | | | | | | | - | | | | | | | | | | | 296 | | | | | | | | | | | 0 | | | | | | | | | | | 58 | | | | | | | | | | | K.ADIEDYLASR.G | | | | | | | | | | | | | |  | | | 1172.4244 | | | 1171.4171 | | | | | | | | | | 1171.6237 | | | | | | | | | | | -176.35 | | | | | | | | | | | 497 | | | | | | | | | | | - | | | | | | | | | | | 507 | | | | | | | | | | | 0 | | | | | | | | | | | --- | | | | | | | | | | | R.VVPGLGPDQYK.F | | | | | | | | | | | | | |  | | | 1215.4045 | | | 1214.3972 | | | | | | | | | | 1214.5891 | | | | | | | | | | | -157.97 | | | | | | | | | | | 243 | | | | | | | | | | | - | | | | | | | | | | | 254 | | | | | | | | | | | 0 | | | | | | | | | | | --- | | | | | | | | | | | K.ASKPSAASPEDR.L | | | | | | | | | | | | | |  | | | 1328.4133 | | | 1327.4060 | | | | | | | | | | 1327.7248 | | | | | | | | | | | -240.12 | | | | | | | | | | | 496 | | | | | | | | | | | - | | | | | | | | | | | 507 | | | | | | | | | | | 1 | | | | | | | | | | | --- | | | | | | | | | | | K.RVVPGLGPDQYK.F | | | | | | | | | | | | | |  | | | 1359.4515 | | | 1358.4442 | | | | | | | | | | 1358.6790 | | | | | | | | | | | -172.78 | | | | | | | | | | | 359 | | | | | | | | | | | - | | | | | | | | | | | 371 | | | | | | | | | | | 0 | | | | | | | | | | | --- | | | | | | | | | | | R.NQLNSIQEASAGK.R | | | | | | | | | | | | | |  | | | 1435.4856 | | | 1434.4783 | | | | | | | | | | 1434.6627 | | | | | | | | | | | -128.50 | | | | | | | | | | | 205 | | | | | | | | | | | - | | | | | | | | | | | 219 | | | | | | | | | | | 0 | | | | | | | | | | | --- | | | | | | | | | | | K.DYSPSVSDAGAAPAK.E | | | | | | | | | | | | | |  | | | 1435.4856 | | | 1434.4783 | | | | | | | | | | 1434.6627 | | | | | | | | | | | -128.50 | | | | | | | | | | | 205 | | | | | | | | | | | - | | | | | | | | | | | 219 | | | | | | | | | | | 0 | | | | | | | | | | | --- | | | | | | | | | | | K.DYSPSVSDAGAAPAK.E | | | | | | | | | | | | | |  | | | 1540.5126 | | | 1539.5053 | | | | | | | | | | 1539.7892 | | | | | | | | | | | -184.39 | | | | | | | | | | | 263 | | | | | | | | | | | - | | | | | | | | | | | 276 | | | | | | | | | | | 0 | | | | | | | | | | | --- | | | | | | | | | | | R.NLAEEHNVSLSSIK.G | | | | | | | | | | | | | |  | | | 1869.6155 | | | 1868.6082 | | | | | | | | | | 1868.9619 | | | | | | | | | | | -189.22 | | | | | | | | | | | 186 | | | | | | | | | | | - | | | | | | | | | | | 202 | | | | | | | | | | | 0 | | | | | | | | | | | --- | | | | | | | | | | | K.VGEVIAITVEEEEDIPK.F | | | | | | | | | | | | | |  | | | 1979.6733 | | | 1978.6660 | | | | | | | | | | 1979.0112 | | | | | | | | | | | -174.43 | | | | | | | | | | | 309 | | | | | | | | | | | - | | | | | | | | | | | 326 | | | | | | | | | | | 0 | | | | | | | | | | | --- | | | | | | | | | | | K.DVAAPALDYVDIPHSQIR.K | | | | | | | | | | | | | |  | | | 1979.6733 | | | 1978.6660 | | | | | | | | | | 1979.0112 | | | | | | | | | | | -174.43 | | | | | | | | | | | 309 | | | | | | | | | | | - | | | | | | | | | | | 326 | | | | | | | | | | | 0 | | | | | | | | | | | 94 | | | | | | | | | | | K.DVAAPALDYVDIPHSQIR.K | | | | | | | | | | | | | |  | | | 2164.7468 | | | 2163.7395 | | | | | | | | | | 2164.1277 | | | | | | | | | | | -179.35 | | | | | | | | | | | 307 | | | | | | | | | | | - | | | | | | | | | | | 326 | | | | | | | | | | | 1 | | | | | | | | | | | --- | | | | | | | | | | | K.GKDVAAPALDYVDIPHSQIR.K | | | | | | | | | | | | | |  | | | 2211.8384 | | | 2210.8311 | | | | | | | | | | 2211.2012 | | | | | | | | | | | -167.34 | | | | | | | | | | | 407 | | | | | | | | | | | - | | | | | | | | | | | 426 | | | | | | | | | | | 0 | | | | | | | | | | | --- | | | | | | | | | | | K.NVNINVAVQTENGLYVPVIR.D | | | | | | | | | | | | | |  | | | 2361.7407 | | | 2360.7334 | | | | | | | | | | 2361.1488 | | | | | | | | | | | -175.94 | | | | | | | | | | | 205 | | | | | | | | | | | - | | | | | | | | | | | 228 | | | | | | | | | | | 1 | | | | | | | | | | | --- | | | | | | | | | | | K.DYSPSVSDAGAAPAKEPSPPPPPK.Q | | | | | | | | | | | | | |  | | | 2526.8291 | | | 2525.8218 | | | | | | | | | | 2526.2853 | | | | | | | | | | | -183.47 | | | | | | | | | | | 220 | | | | | | | | | | | - | | | | | | | | | | | 242 | | | | | | | | | | | 1 | | | | | | | | | | | --- | | | | | | | | | | | K.EPSPPPPPKQEEVEKPISTSEPK.A | | | | | | | | | | | | | |  | | | 2754.8496 | | | 2753.8423 | | | | | | | | | | 2754.3501 | | | | | | | | | | | -184.35 | | | | | | | | | | | 449 | | | | | | | | | | | - | | | | | | | | | | | 474 | | | | | | | | | | | 0 | | | | | | | | | | | --- | | | | | | | | | | | K.DNSLKPQDYEGGTFTVTNLGGPFGIK.Q | | | | | | | | | | | | | |  | | | 2754.8496 | | | 2753.8423 | | | | | | | | | | 2754.3501 | | | | | | | | | | | -184.35 | | | | | | | | | | | 449 | | | | | | | | | | | - | | | | | | | | | | | 474 | | | | | | | | | | | 0 | | | | | | | | | | | 87 | | | | | | | | | | | K.DNSLKPQDYEGGTFTVTNLGGPFGIK.Q | | | | | | | | | | | | | |  | | | No match to: 700.2446, 707.2474, 713.2718, 730.2510, 734.2718, 763.2454, 768.3631, 770.2525, 792.2447, 841.2651, 842.3431, 850.3436, 859.2967, 868.3711, 873.3875, 876.8827, 882.3566, 901.2775, 929.2770, 970.2925, 972.2571, 984.2342, 989.2873, 990.3638, 1019.3970, 1021.3527, 1029.3796, 1051.4994, 1059.3910, 1151.4380, 1157.4663, 1170.3801, 1179.3615, 1186.4115, 1187.4590, 1217.4015, 1261.4989, 1275.4509, 1287.4666, 1303.4336, 1309.4021, 1312.4733, 1313.4629, 1314.4042, 1315.3649, 1318.3953, 1326.3417, 1326.3417, 1341.3805, 1342.5033, 1357.4504, 1381.4592, 1397.3662, 1403.3180, 1414.3802, 1415.3171, 1415.3171, 1431.3124, 1432.3157, 1451.4921, 1465.5144, 1513.4928, 1537.4758, 1539.4611, 1566.5560, 1583.5067, 1591.4808, 1598.4633, 1625.5724, 1705.3922, 1800.5060, 1819.5636, 1930.5726, 2001.6179, 2007.6974, 2007.6974, 2018.5861, 2041.5863, 2192.7715, 2192.7715, 2218.8027, 2238.7271, 2278.7979, 2395.7852, 2903.8704, 2967.8323, 2983.8381 | | | | | | | | | | | | | | | | | | | | | | | | | | | | | | | | | | | | | | | | | | | | | | | | | | | | | | | | | | | | | | | | | | | | | | | | | | | | | | | | | | | | | | | | | | | | | | | | | | | | | |  | | | | | **S32** [**Cs2g21190.1**](http://zhangyang-pc/mascot/cgi/protein_view.pl?file=../data/20140118/F012181.dat&hit=1) **Mass: 51029 Score: 181 Expect: 3.5e-014 Matches: 11** | | | | | | | | | | | | | | | | | | | | | | | | | | | | | | | | | | | | | | | | | | | | | | | | | | | | | | | | | | | | | | | | | | | | | | | | | | | | | | | | | | | | | | | | | | | | | | | | | | | | | |  | | | | | Observed | | | | | | Mr(expt) | | | | | | | | | | Mr(calc) | | | | | | | | | | | ppm | | | | | | | | | | | Start | | | | | | | | | | | |  | | | | | | | | | | End | | | | | | | | | | | Miss | | | | | | | | | | | Ions | | | | | | | | | | | Peptide | | | | | | | | | | |  | | | 743.2371 | | | | | | 742.2298 | | | | | | | | | | 742.4490 | | | | | | | | | | | -295.25 | | | | | | | | | | | 2 | | | | | | | | | | | | - | | | | | | | | | | 7 | | | | | | | | | | | 0 | | | | | | | | | | | --- | | | | | | | | | | | M.IWGIVR.R | | | | | | | | | | |  | | | 775.2405 | | | | | | 774.2333 | | | | | | | | | | 774.4170 | | | | | | | | | | | -237.31 | | | | | | | | | | | 242 | | | | | | | | | | | | - | | | | | | | | | | 247 | | | | | | | | | | | 1 | | | | | | | | | | | --- | | | | | | | | | | | R.RVPMTR.L + Oxidation (M) | | | | | | | | | | |  | | | 852.3755 | | | | | | 851.3683 | | | | | | | | | | 851.5593 | | | | | | | | | | | -224.30 | | | | | | | | | | | 342 | | | | | | | | | | | | - | | | | | | | | | | 349 | | | | | | | | | | | 0 | | | | | | | | | | | --- | | | | | | | | | | | K.GLVVPVIR.N | | | | | | | | | | |  | | | 881.3101 | | | | | | 880.3028 | | | | | | | | | | 880.4807 | | | | | | | | | | | -202.05 | | | | | | | | | | | 447 | | | | | | | | | | | | - | | | | | | | | | | 453 | | | | | | | | | | | 0 | | | | | | | | | | | --- | | | | | | | | | | | R.EAVFFLR.R | | | | | | | | | | |  | | | 881.3101 | | | | | | 880.3028 | | | | | | | | | | 880.4807 | | | | | | | | | | | -202.05 | | | | | | | | | | | 447 | | | | | | | | | | | | - | | | | | | | | | | 453 | | | | | | | | | | | 0 | | | | | | | | | | | 29 | | | | | | | | | | | R.EAVFFLR.R | | | | | | | | | | |  | | | 1328.4222 | | | | | | 1327.4149 | | | | | | | | | | 1327.6660 | | | | | | | | | | | -189.10 | | | | | | | | | | | 329 | | | | | | | | | | | | - | | | | | | | | | | 340 | | | | | | | | | | | 0 | | | | | | | | | | | --- | | | | | | | | | | | R.DYIDISFAVGTK.K | | | | | | | | | | |  | | | 1783.5172 | | | | | | 1782.5099 | | | | | | | | | | 1782.8359 | | | | | | | | | | | -182.85 | | | | | | | | | | | 48 | | | | | | | | | | | | - | | | | | | | | | | 63 | | | | | | | | | | | 0 | | | | | | | | | | | --- | | | | | | | | | | | R.SSYHILSGNYVCSTPR.S | | | | | | | | | | |  | | | 1825.5007 | | | | | | 1824.4934 | | | | | | | | | | 1824.9945 | | | | | | | | | | | -274.56 | | | | | | | | | | | 161 | | | | | | | | | | | | - | | | | | | | | | | 179 | | | | | | | | | | | 1 | | | | | | | | | | | --- | | | | | | | | | | | K.IAVISKSGEGVAQAAPAEK.A | | | | | | | | | | |  | | | 1951.5697 | | | | | | 1950.5624 | | | | | | | | | | 1950.1514 | | | | | | | | | | | 211 | | | | | | | | | | | 132 | | | | | | | | | | | | - | | | | | | | | | | 150 | | | | | | | | | | | 1 | | | | | | | | | | | --- | | | | | | | | | | | K.VTIDVASPQAGVIQKLIAK.E | | | | | | | | | | |  | | | 2563.8574 | | | | | | 2562.8501 | | | | | | | | | | 2563.3394 | | | | | | | | | | | -190.89 | | | | | | | | | | | 305 | | | | | | | | | | | | - | | | | | | | | | | 328 | | | | | | | | | | | 0 | | | | | | | | | | | --- | | | | | | | | | | | K.AAVSALQHQPVVNAVIDGDDIIYR.D | | | | | | | | | | |  | | | 2563.8574 | | | | | | 2562.8501 | | | | | | | | | | 2563.3394 | | | | | | | | | | | -190.89 | | | | | | | | | | | 305 | | | | | | | | | | | | - | | | | | | | | | | 328 | | | | | | | | | | | 0 | | | | | | | | | | | 135 | | | | | | | | | | | K.AAVSALQHQPVVNAVIDGDDIIYR.D | | | | | | | | | | |  | | | No match to: 700.2492, 710.2061, 712.1056, 713.2723, 716.2440, 718.2320, 730.2311, 734.3436, 737.2286, 744.2346, 750.3682, 756.2013, 765.2045, 768.3460, 834.3383, 836.3323, 842.3388, 846.2056, 848.3494, 849.2907, 850.3439, 860.8995, 864.3096, 868.3638, 873.2306, 876.8669, 880.2675, 924.3155, 982.3580, 988.3398, 1017.4688, 1051.4740, 1059.3621, 1059.3621, 1061.3705, 1117.4410, 1125.4446, 1151.4774, 1176.4229, 1275.3679, 1297.4614, 1301.4089, 1303.4211, 1313.3986, 1315.3993, 1331.3850, 1340.4833, 1384.4275, 1448.4775, 1537.4513, 1554.4222, 1554.4222, 1566.4174, 1566.4174, 1582.4036, 1584.4265, 1596.4178, 1596.4178, 1636.4950, 1639.5953, 1646.4731, 1647.5166, 1647.5166, 1659.5349, 1675.5275, 1675.5275, 1701.5339, 1709.6008, 1794.5104, 1801.4231, 1810.5338, 1895.6069, 1922.5865, 1934.5543, 1954.5964, 1993.6157, 2002.5863, 2008.6283, 2016.6447, 2020.6113, 2032.5930, 2144.6335, 2155.7554, 2161.6758, 2220.6960, 2226.7112, 2238.7090, 2238.7090, 2260.6855, 2290.7192, 2290.7192, 2306.6980, 2400.6538, 2417.6597, 2423.6548, 2568.7539, 2694.7695, 2914.9246, 3346.0056 | | | | | | | | | | | | | | | | | | | | | | | | | | | | | | | | | | | | | | | | | | | | | | | | | | | | | | | | | | | | | | | | | | | | | | | | | | | | | | | | | | | | | | | | | | | | | | | | | | | | | |  | | | | | **S3** [**Cs8g06410.1**](http://zhangyang-pc/mascot/cgi/protein_view.pl?file=../data/20140118/F012153.dat&hit=1) **Mass: 26089 Score: 238 Expect: 7e-020 Matches: 20** | | | | | | | | | | | | | | | | | | | | | | | | | | | | | | | | | | | | | | | | | | | | | | | | | | | | | | | | | | | | | | | | | | | | | | | | | | | | | | | | | | | | | | | | | | | | | | | | | | |  | | | | | | | | Observed | | | | | | | | | Mr(expt) | | | | | | | | | | Mr(calc) | | | | | | | | | | | ppm | | | | | | | | | | | Start | | | | | | | | | | | |  | | | | | | | | | | End | | | | | | | | | | | Miss | | | | | | | | | | | Ions | | | | | | | | | | | Peptide | | | | | | | | | | | 701.2761 | | | | | | | | | 700.2688 | | | | | | | | | | 700.4483 | | | | | | | | | | | -256.23 | | | | | | | | | | | 83 | | | | | | | | | | | | - | | | | | | | | | | 89 | | | | | | | | | | | 0 | | | | | | | | | | | --- | | | | | | | | | | | R.GLGLTLK.Y | | | | | | | | | | | 719.1993 | | | | | | | | | 718.1921 | | | | | | | | | | 718.3326 | | | | | | | | | | | -195.68 | | | | | | | | | | | 90 | | | | | | | | | | | | - | | | | | | | | | | 94 | | | | | | | | | | | 0 | | | | | | | | | | | --- | | | | | | | | | | | K.YFFDK.K | | | | | | | | | | | 754.2307 | | | | | | | | | 753.2234 | | | | | | | | | | 753.3657 | | | | | | | | | | | -188.83 | | | | | | | | | | | 221 | | | | | | | | | | | | - | | | | | | | | | | 226 | | | | | | | | | | | 0 | | | | | | | | | | | --- | | | | | | | | | | | R.SESLYR.- | | | | | | | | | | | 816.2618 | | | | | | | | | 815.2546 | | | | | | | | | | 815.4137 | | | | | | | | | | | -195.17 | | | | | | | | | | | 204 | | | | | | | | | | | | - | | | | | | | | | | 210 | | | | | | | | | | | 0 | | | | | | | | | | | --- | | | | | | | | | | | K.LLENGDR.W | | | | | | | | | | | 847.2742 | | | | | | | | | 846.2670 | | | | | | | | | | 846.4276 | | | | | | | | | | | -189.77 | | | | | | | | | | | 90 | | | | | | | | | | | | - | | | | | | | | | | 95 | | | | | | | | | | | 1 | | | | | | | | | | | --- | | | | | | | | | | | K.YFFDKK.V | | | | | | | | | | | 851.2313 | | | | | | | | | 850.2240 | | | | | | | | | | 850.3821 | | | | | | | | | | | -185.91 | | | | | | | | | | | 120 | | | | | | | | | | | | - | | | | | | | | | | 126 | | | | | | | | | | | 0 | | | | | | | | | | | --- | | | | | | | | | | | R.YPTGEER.C | | | | | | | | | | | 851.2313 | | | | | | | | | 850.2240 | | | | | | | | | | 850.3821 | | | | | | | | | | | -185.91 | | | | | | | | | | | 120 | | | | | | | | | | | | - | | | | | | | | | | 126 | | | | | | | | | | | 0 | | | | | | | | | | | 17 | | | | | | | | | | | R.YPTGEER.C | | | | | | | | | | | 885.2821 | | | | | | | | | 884.2748 | | | | | | | | | | 884.3950 | | | | | | | | | | | -135.84 | | | | | | | | | | | 159 | | | | | | | | | | | | - | | | | | | | | | | 165 | | | | | | | | | | | 0 | | | | | | | | | | | --- | | | | | | | | | | | R.YDIDMTK.C | | | | | | | | | | | 985.3545 | | | | | | | | | 984.3472 | | | | | | | | | | 984.5253 | | | | | | | | | | | -180.92 | | | | | | | | | | | 111 | | | | | | | | | | | | - | | | | | | | | | | 118 | | | | | | | | | | | 1 | | | | | | | | | | | --- | | | | | | | | | | | R.FRGEHALR.R | | | | | | | | | | | 985.3545 | | | | | | | | | 984.3472 | | | | | | | | | | 984.5253 | | | | | | | | | | | -180.92 | | | | | | | | | | | 111 | | | | | | | | | | | | - | | | | | | | | | | 118 | | | | | | | | | | | 1 | | | | | | | | | | | 36 | | | | | | | | | | | R.FRGEHALR.R | | | | | | | | | | | 1007.3057 | | | | | | | | | 1006.2984 | | | | | | | | | | 1006.4832 | | | | | | | | | | | -183.57 | | | | | | | | | | | 119 | | | | | | | | | | | | - | | | | | | | | | | 126 | | | | | | | | | | | 1 | | | | | | | | | | | --- | | | | | | | | | | | R.RYPTGEER.C | | | | | | | | | | | 1007.3057 | | | | | | | | | 1006.2984 | | | | | | | | | | 1006.4832 | | | | | | | | | | | -183.57 | | | | | | | | | | | 119 | | | | | | | | | | | | - | | | | | | | | | | 126 | | | | | | | | | | | 1 | | | | | | | | | | | 45 | | | | | | | | | | | R.RYPTGEER.C | | | | | | | | | | | 1110.3815 | | | | | | | | | 1109.3742 | | | | | | | | | | 1109.5757 | | | | | | | | | | | -181.58 | | | | | | | | | | | 96 | | | | | | | | | | | | - | | | | | | | | | | 104 | | | | | | | | | | | 0 | | | | | | | | | | | --- | | | | | | | | | | | K.VTINYPFEK.G | | | | | | | | | | | 1110.3815 | | | | | | | | | 1109.3742 | | | | | | | | | | 1109.5757 | | | | | | | | | | | -181.58 | | | | | | | | | | | 96 | | | | | | | | | | | | - | | | | | | | | | | 104 | | | | | | | | | | | 0 | | | | | | | | | | | 38 | | | | | | | | | | | K.VTINYPFEK.G | | | | | | | | | | | 1238.4402 | | | | | | | | | 1237.4329 | | | | | | | | | | 1237.6707 | | | | | | | | | | | -192.08 | | | | | | | | | | | 95 | | | | | | | | | | | | - | | | | | | | | | | 104 | | | | | | | | | | | 1 | | | | | | | | | | | --- | | | | | | | | | | | K.KVTINYPFEK.G | | | | | | | | | | | 1246.3986 | | | | | | | | | 1245.3913 | | | | | | | | | | 1245.6088 | | | | | | | | | | | -174.62 | | | | | | | | | | | 49 | | | | | | | | | | | | - | | | | | | | | | | 58 | | | | | | | | | | | 1 | | | | | | | | | | | --- | | | | | | | | | | | K.DDEEKEQLLK.E | | | | | | | | | | | 1260.4398 | | | | | | | | | 1259.4325 | | | | | | | | | | 1259.6146 | | | | | | | | | | | -144.53 | | | | | | | | | | | 211 | | | | | | | | | | | | - | | | | | | | | | | 220 | | | | | | | | | | | 0 | | | | | | | | | | | --- | | | | | | | | | | | R.WETEIAENLR.S | | | | | | | | | | | 1469.4777 | | | | | | | | | 1468.4704 | | | | | | | | | | 1468.7418 | | | | | | | | | | | -184.76 | | | | | | | | | | | 71 | | | | | | | | | | | | - | | | | | | | | | | 82 | | | | | | | | | | | 0 | | | | | | | | | | | --- | | | | | | | | | | | R.SINMLFLTEMVR.G + Oxidation (M) | | | | | | | | | | | 1485.4705 | | | | | | | | | 1484.4632 | | | | | | | | | | 1484.7367 | | | | | | | | | | | -184.20 | | | | | | | | | | | 71 | | | | | | | | | | | | - | | | | | | | | | | 82 | | | | | | | | | | | 0 | | | | | | | | | | | --- | | | | | | | | | | | R.SINMLFLTEMVR.G + 2 Oxidation (M) | | | | | | | | | | | 1485.4705 | | | | | | | | | 1484.4632 | | | | | | | | | | 1484.7367 | | | | | | | | | | | -184.20 | | | | | | | | | | | 71 | | | | | | | | | | | | - | | | | | | | | | | 82 | | | | | | | | | | | 0 | | | | | | | | | | | 48 | | | | | | | | | | | R.SINMLFLTEMVR.G + 2 Oxidation (M) | | | | | | | | | | | No match to: 700.2504, 705.2455, 709.2177, 713.2757, 716.2366, 721.2264, 730.2536, 734.3275, 741.2230, 744.2591, 752.2723, 765.2761, 768.3689, 782.2617, 797.2646, 809.2107, 815.2304, 823.2499, 834.2209, 842.3483, 860.9075, 868.3691, 873.2597, 882.3818, 963.3310, 984.3164, 990.2974, 996.3354, 1016.3017, 1018.3196, 1018.3196, 1021.3303, 1023.2797, 1029.2780, 1041.2742, 1041.2742, 1045.3438, 1051.5123, 1052.3383, 1053.3483, 1055.2985, 1057.2733, 1059.3622, 1063.2853, 1081.2914, 1100.3357, 1109.3162, 1116.3123, 1116.3123, 1119.3351, 1125.3201, 1132.3311, 1145.3464, 1151.4108, 1155.3636, 1172.4537, 1197.3280, 1263.4220, 1264.4111, 1276.3979, 1292.3896, 1315.4016, 1322.4022, 1328.4102, 1374.4425, 1413.4003, 1418.4874, 1421.4922, 1437.4741, 1459.4257, 1501.4680, 1501.4680, 1507.4548, 1517.4583, 1519.4446, 1607.6603, 1881.5371, 1984.5942, 2000.6387, 2044.6475, 2055.6418, 2056.6318, 2061.6448, 2072.6497, 2073.6460, 2073.6460, 2089.6499, 2091.6160, 2238.7283, 2245.7683 | | | | | | | | | | | | | | | | | | | | | | | | | | | | | | | | | | | | | | | | | | | | | | | | | | | | | | | | | | | | | | | | | | | | | | | | | | | | | | | | | | | | | | | | | | | | | | | | | | | | | |  | | | | | **S26** [**Cs2g03080.1**](http://zhangyang-pc/mascot/cgi/protein_view.pl?file=../data/20140118/F012171.dat&hit=2) **Mass: 40593 Score: 208 Expect: 7e-017 Matches: 10** | | | | | | | | | | | | | | | | | | | | | | | | | | | | | | | | | | | | | | | | | | | | | | | | | | | | | | | | | | | | | | | | | | | | | | | | | | | | | | | | | | | | | | | | | | | | | | | | | | | | | |  | | | | | Observed | | | | | | | | | | Mr(expt) | | | | | | | | | | Mr(calc) | | | | | | | | | | | ppm | | | | | | | | | | | Start | | | | | | | | | | | |  | | | | | | | | | | End | | | | | | | | | | | Miss | | | | | | | | | | | Ions | | | | | | | | | | | Peptide | | | | | | |  | | | 840.2688 | | | | | | | | | | 839.2615 | | | | | | | | | | 839.4290 | | | | | | | | | | | -199.48 | | | | | | | | | | | 168 | | | | | | | | | | | | - | | | | | | | | | | 174 | | | | | | | | | | | 0 | | | | | | | | | | | --- | | | | | | | | | | | K.GNAYFLR.R | | | | | | |  | | | 846.3135 | | | | | | | | | | 845.3063 | | | | | | | | | | 845.4607 | | | | | | | | | | | -182.62 | | | | | | | | | | | 53 | | | | | | | | | | | | - | | | | | | | | | | 59 | | | | | | | | | | | 1 | | | | | | | | | | | --- | | | | | | | | | | | R.ERIDSVK.N | | | | | | |  | | | 978.3517 | | | | | | | | | | 977.3444 | | | | | | | | | | 977.4423 | | | | | | | | | | | -100.12 | | | | | | | | | | | 2 | | | | | | | | | | | | - | | | | | | | | | | 10 | | | | | | | | | | | 0 | | | | | | | | | | | --- | | | | | | | | | | | M.SCSNLPVCR.D | | | | | | |  | | | 1015.3783 | | | | | | | | | | 1014.3710 | | | | | | | | | | 1014.5611 | | | | | | | | | | | -187.30 | | | | | | | | | | | 175 | | | | | | | | | | | | - | | | | | | | | | | 182 | | | | | | | | | | | 0 | | | | | | | | | | | --- | | | | | | | | | | | R.RPYIPVDR.F | | | | | | |  | | | 1028.4233 | | | | | | | | | | 1027.4160 | | | | | | | | | | 1027.6026 | | | | | | | | | | | -181.58 | | | | | | | | | | | 122 | | | | | | | | | | | | - | | | | | | | | | | 131 | | | | | | | | | | | 0 | | | | | | | | | | | --- | | | | | | | | | | | K.VALVVVTGDR.G | | | | | | |  | | | 1261.4636 | | | | | | | | | | 1260.4563 | | | | | | | | | | 1260.7111 | | | | | | | | | | | -202.11 | | | | | | | | | | | 64 | | | | | | | | | | | | - | | | | | | | | | | 75 | | | | | | | | | | | 1 | | | | | | | | | | | --- | | | | | | | | | | | K.ITEAMKLVAAAK.V + Oxidation (M) | | | | | | |  | | | 1374.4780 | | | | | | | | | | 1373.4707 | | | | | | | | | | 1373.7150 | | | | | | | | | | | -177.83 | | | | | | | | | | | 311 | | | | | | | | | | | | - | | | | | | | | | | 323 | | | | | | | | | | | 0 | | | | | | | | | | | --- | | | | | | | | | | | R.ALQESLASELASR.M | | | | | | |  | | | 1374.4780 | | | | | | | | | | 1373.4707 | | | | | | | | | | 1373.7150 | | | | | | | | | | | -177.83 | | | | | | | | | | | 311 | | | | | | | | | | | | - | | | | | | | | | | 323 | | | | | | | | | | | 0 | | | | | | | | | | | 84 | | | | | | | | | | | R.ALQESLASELASR.M | | | | | | |  | | | 1544.6101 | | | | | | | | | | 1543.6028 | | | | | | | | | | 1543.8722 | | | | | | | | | | | -174.50 | | | | | | | | | | | 227 | | | | | | | | | | | | - | | | | | | | | | | 240 | | | | | | | | | | | 0 | | | | | | | | | | | --- | | | | | | | | | | | K.SDPVIHTLLPLSPR.G | | | | | | |  | | | 1544.6101 | | | | | | | | | | 1543.6028 | | | | | | | | | | 1543.8722 | | | | | | | | | | | -174.50 | | | | | | | | | | | 227 | | | | | | | | | | | | - | | | | | | | | | | 240 | | | | | | | | | | | 0 | | | | | | | | | | | 103 | | | | | | | | | | | K.SDPVIHTLLPLSPR.G | | | | | | |  | | | No match to: 705.2244, 710.1734, 712.2504, 728.1970, 729.2390, 734.2925, 745.2325, 768.2955, 800.2640, 842.3036, 860.8995, 876.8777, 943.3184, 997.3082, 1006.3324, 1023.2857, 1030.2937, 1036.3309, 1047.3101, 1050.3699, 1050.3699, 1096.3794, 1108.3297, 1108.3297, 1121.3719, 1128.3381, 1138.3392, 1145.3998, 1157.4530, 1158.5170, 1158.5170, 1187.4188, 1215.4437, 1227.4669, 1263.4370, 1285.4468, 1294.4829, 1315.4011, 1342.5507, 1385.5050, 1445.4521, 1449.4883, 1461.4728, 1465.4817, 1466.4633, 1477.4703, 1480.5280, 1493.4711, 1499.4204, 1516.4479, 1558.4938, 1564.6006, 1574.5734, 1721.4491, 1722.4510, 1736.5031, 1738.4636, 1738.4636, 1752.4963, 1775.7649, 1777.5557, 1783.6190, 1785.6178, 1785.6178, 1835.5869, 1843.4406, 1866.5463, 1867.5217, 1876.6028, 1892.5259, 1986.7119, 1986.7119, 2158.7446, 2168.7239, 2170.7410, 2170.7410, 2179.3923, 2179.9333, 2180.7300, 2183.6956, 2186.7312, 2201.7363, 2208.7285, 2227.6523, 2230.6780, 2238.7224, 2271.6997, 2287.6218, 2301.5762, 2307.9209, 2308.7957, 2357.7888, 2397.8108, 2400.8467, 2415.7283, 2804.8318, 2805.8149, 2822.8008, 2822.8008, 3003.0142 | | | | | | | | | | | | | | | | | | | | | | | | | | | | | | | | | | | | | | | | | | | | | | | | | | | | | | | | | | | | | | | | | | | | | | | | | | | | | | | | | | | | | | | | | | | | | | | | | | | | | |  | | | | | **S30** [**Cs1g04030.1**](http://zhangyang-pc/mascot/cgi/protein_view.pl?file=../data/20140118/F012126.dat&hit=1) **Mass: 27505 Score: 368 Expect: 7e-033 Matches: 22** | | | | | | | | | | | | | | | | | | | | | | | | | | | | | | | | | | | | | | | | | | | | | | | | | | | | | | | | | | | | | | | | | | | | | | | | | | | | | | | | | | | | | | | | | | | | | | | | | | | | | |  | | | | | Observed | | | | | | | | | | Mr(expt) | | | | | | | | | | Mr(calc) | | | | | | | | | | | ppm | | | | | | | | | | | Start | | | | | | | | | | | |  | | | | | | | | | | End | | | | | | | | | | | Miss | | | | | | | | | | | Ions | | | | | | | | | | | Peptide | | | | | | |  | | | 729.2766 | | | | | | | | | | 728.2693 | | | | | | | | | | 728.4068 | | | | | | | | | | | -188.85 | | | | | | | | | | | 184 | | | | | | | | | | | | - | | | | | | | | | | 189 | | | | | | | | | | | 1 | | | | | | | | | | | --- | | | | | | | | | | | R.KEDLPK.Y | | | | | | |  | | | 800.2822 | | | | | | | | | | 799.2750 | | | | | | | | | | 799.4188 | | | | | | | | | | | -179.95 | | | | | | | | | | | 111 | | | | | | | | | | | | - | | | | | | | | | | 118 | | | | | | | | | | | 0 | | | | | | | | | | | --- | | | | | | | | | | | R.TAGIPDAR.T | | | | | | |  | | | 830.2683 | | | | | | | | | | 829.2610 | | | | | | | | | | 829.4545 | | | | | | | | | | | -233.24 | | | | | | | | | | | 201 | | | | | | | | | | | | - | | | | | | | | | | 207 | | | | | | | | | | | 0 | | | | | | | | | | | --- | | | | | | | | | | | K.AQLEELK.K | | | | | | |  | | | 848.3302 | | | | | | | | | | 847.3229 | | | | | | | | | | 847.4803 | | | | | | | | | | | -185.75 | | | | | | | | | | | 49 | | | | | | | | | | | | - | | | | | | | | | | 55 | | | | | | | | | | | 0 | | | | | | | | | | | --- | | | | | | | | | | | K.NIFLDVK.K | | | | | | |  | | | 958.3527 | | | | | | | | | | 957.3454 | | | | | | | | | | 957.5495 | | | | | | | | | | | -213.12 | | | | | | | | | | | 201 | | | | | | | | | | | | - | | | | | | | | | | 208 | | | | | | | | | | | 1 | | | | | | | | | | | --- | | | | | | | | | | | K.AQLEELKK.D | | | | | | |  | | | 966.3590 | | | | | | | | | | 965.3517 | | | | | | | | | | 965.5182 | | | | | | | | | | | -172.45 | | | | | | | | | | | 103 | | | | | | | | | | | | - | | | | | | | | | | 110 | | | | | | | | | | | 0 | | | | | | | | | | | --- | | | | | | | | | | | R.IAYTIETR.T | | | | | | |  | | | 966.3590 | | | | | | | | | | 965.3517 | | | | | | | | | | 965.5182 | | | | | | | | | | | -172.45 | | | | | | | | | | | 103 | | | | | | | | | | | | - | | | | | | | | | | 110 | | | | | | | | | | | 0 | | | | | | | | | | | 36 | | | | | | | | | | | R.IAYTIETR.T | | | | | | |  | | | 976.3612 | | | | | | | | | | 975.3539 | | | | | | | | | | 975.5753 | | | | | | | | | | | -226.91 | | | | | | | | | | | 49 | | | | | | | | | | | | - | | | | | | | | | | 56 | | | | | | | | | | | 1 | | | | | | | | | | | --- | | | | | | | | | | | K.NIFLDVKK.K | | | | | | |  | | | 1005.4025 | | | | | | | | | | 1004.3952 | | | | | | | | | | 1004.5655 | | | | | | | | | | | -169.48 | | | | | | | | | | | 58 | | | | | | | | | | | | - | | | | | | | | | | 66 | | | | | | | | | | | 0 | | | | | | | | | | | --- | | | | | | | | | | | K.FETALGVLR.K | | | | | | |  | | | 1005.4025 | | | | | | | | | | 1004.3952 | | | | | | | | | | 1004.5655 | | | | | | | | | | | -169.48 | | | | | | | | | | | 58 | | | | | | | | | | | | - | | | | | | | | | | 66 | | | | | | | | | | | 0 | | | | | | | | | | | 48 | | | | | | | | | | | K.FETALGVLR.K | | | | | | |  | | | 1051.3857 | | | | | | | | | | 1050.3784 | | | | | | | | | | 1050.5233 | | | | | | | | | | | -137.91 | | | | | | | | | | | 190 | | | | | | | | | | | | - | | | | | | | | | | 197 | | | | | | | | | | | 0 | | | | | | | | | | | --- | | | | | | | | | | | K.YEEQLELK.I | | | | | | |  | | | 1052.3301 | | | | | | | | | | 1051.3228 | | | | | | | | | | 1051.4934 | | | | | | | | | | | -162.24 | | | | | | | | | | | 94 | | | | | | | | | | | | - | | | | | | | | | | 102 | | | | | | | | | | | 0 | | | | | | | | | | | --- | | | | | | | | | | | K.ADLFSESQR.I | | | | | | |  | | | 1052.3301 | | | | | | | | | | 1051.3228 | | | | | | | | | | 1051.4934 | | | | | | | | | | | -162.24 | | | | | | | | | | | 94 | | | | | | | | | | | | - | | | | | | | | | | 102 | | | | | | | | | | | 0 | | | | | | | | | | | 46 | | | | | | | | | | | K.ADLFSESQR.I | | | | | | |  | | | 1133.4731 | | | | | | | | | | 1132.4658 | | | | | | | | | | 1132.6604 | | | | | | | | | | | -171.81 | | | | | | | | | | | 58 | | | | | | | | | | | | - | | | | | | | | | | 67 | | | | | | | | | | | 1 | | | | | | | | | | | --- | | | | | | | | | | | K.FETALGVLRK.E | | | | | | |  | | | 1133.4731 | | | | | | | | | | 1132.4658 | | | | | | | | | | 1132.6604 | | | | | | | | | | | -171.81 | | | | | | | | | | | 57 | | | | | | | | | | | | - | | | | | | | | | | 66 | | | | | | | | | | | 1 | | | | | | | | | | | 77 | | | | | | | | | | | K.KFETALGVLR.K | | | | | | |  | | | 1211.4049 | | | | | | | | | | 1210.3976 | | | | | | | | | | 1210.5904 | | | | | | | | | | | -159.21 | | | | | | | | | | | 165 | | | | | | | | | | | | - | | | | | | | | | | 175 | | | | | | | | | | | 0 | | | | | | | | | | | --- | | | | | | | | | | | K.GMALLTAEFDK.I + Oxidation (M) | | | | | | |  | | | 1279.4963 | | | | | | | | | | 1278.4890 | | | | | | | | | | 1278.6125 | | | | | | | | | | | -96.60 | | | | | | | | | | | 209 | | | | | | | | | | | | - | | | | | | | | | | 219 | | | | | | | | | | | 1 | | | | | | | | | | | --- | | | | | | | | | | | K.DALEAMETQKK.R + Oxidation (M) | | | | | | |  | | | 1309.4272 | | | | | | | | | | 1308.4199 | | | | | | | | | | 1308.6310 | | | | | | | | | | | -161.27 | | | | | | | | | | | 92 | | | | | | | | | | | | - | | | | | | | | | | 102 | | | | | | | | | | | 1 | | | | | | | | | | | --- | | | | | | | | | | | R.EKADLFSESQR.I | | | | | | |  | | | 1309.4272 | | | | | | | | | | 1308.4199 | | | | | | | | | | 1308.6310 | | | | | | | | | | | -161.27 | | | | | | | | | | | 92 | | | | | | | | | | | | - | | | | | | | | | | 102 | | | | | | | | | | | 1 | | | | | | | | | | | 96 | | | | | | | | | | | R.EKADLFSESQR.I | | | | | | |  | | | 1339.4620 | | | | | | | | | | 1338.4547 | | | | | | | | | | 1338.6853 | | | | | | | | | | | -172.25 | | | | | | | | | | | 164 | | | | | | | | | | | | - | | | | | | | | | | 175 | | | | | | | | | | | 1 | | | | | | | | | | | --- | | | | | | | | | | | K.KGMALLTAEFDK.I + Oxidation (M) | | | | | | |  | | | 1527.4906 | | | | | | | | | | 1526.4833 | | | | | | | | | | 1526.6810 | | | | | | | | | | | -129.49 | | | | | | | | | | | 221 | | | | | | | | | | | | - | | | | | | | | | | 232 | | | | | | | | | | | 1 | | | | | | | | | | | --- | | | | | | | | | | | R.EEFKDEEMVEVK.S + Oxidation (M) | | | | | | |  | | | 2033.6353 | | | | | | | | | | 2032.6280 | | | | | | | | | | 2033.0139 | | | | | | | | | | | -189.81 | | | | | | | | | | | 70 | | | | | | | | | | | | - | | | | | | | | | | 88 | | | | | | | | | | | 0 | | | | | | | | | | | --- | | | | | | | | | | | K.ITIAPEDPAAVSQYANVMK.T + Oxidation (M) | | | | | | |  | | | No match to: 700.2654, 706.2560, 709.2471, 713.2880, 716.2310, 718.2532, 722.2776, 724.3056, 727.2518, 730.2374, 734.3403, 744.2432, 750.3486, 756.2597, 762.1844, 768.3915, 812.2790, 823.2396, 834.3303, 836.3213, 842.3602, 850.3580, 860.9160, 864.3342, 868.3889, 870.3271, 882.3996, 908.2800, 914.2873, 930.2911, 951.3587, 956.3710, 970.3136, 982.3881, 995.3658, 996.3867, 1019.4189, 1022.3422, 1036.3719, 1045.3843, 1057.3779, 1059.3921, 1060.3995, 1072.3633, 1073.3125, 1104.4066, 1119.4316, 1156.4211, 1160.3152, 1184.3848, 1188.3934, 1205.4781, 1205.4781, 1237.4258, 1251.3619, 1315.4297, 1371.5205, 1421.4652, 1458.5001, 1547.4706, 1559.5876, 1571.5098, 1619.5870, 1619.5870, 1635.5233, 1656.5293, 1660.6105, 1683.5363, 1685.5283, 1718.6150, 1721.5902, 1721.5902, 1731.6429, 1738.5775, 1796.6537, 1811.5706, 1890.6744, 1921.7200, 2002.7070, 2008.4574, 2072.6741, 2072.6741, 2220.7700, 2238.7495, 2238.7495, 2245.8071, 2433.6936, 3346.0374 | | | | | | | | | | | | | | | | | | | | | | | | | | | | | | | | | | | | | | | | | | | | | | | | | | | | | | | | | | | | | | | | | | | | | | | | | | | | | | | | | | | | | | | | | | | | | | | | | | | | | |  | | | | | **S36** [**Cs1g04030.1**](http://zhangyang-pc/mascot/cgi/protein_view.pl?file=../data/20140118/F012142.dat&hit=1) **Mass: 27505 Score: 543 Expect: 2.2e-050 Matches: 27** | | | | | | | | | | | | | | | | | | | | | | | | | | | | | | | | | | | | | | | | | | | | | | | | | | | | | | | | | | | | | | | | | | | | | | | | | | | | | | | | | | | | | | | | | | | | | | | | | | | | | |  | | | | | Observed | | | | | | | | | | Mr(expt) | | | | | | | | | | Mr(calc) | | | | | | | | | | | ppm | | | | | | | | | | | Start | | | | | | | | | | | |  | | | | | | | | | | End | | | | | | | | | | | Miss | | | | | | | | | | | Ions | | | | | | | | | | | Peptide | | | | | | |  | | | 729.2379 | | | | | | | | | | 728.2306 | | | | | | | | | | 728.4068 | | | | | | | | | | | -241.88 | | | | | | | | | | | 184 | | | | | | | | | | | | - | | | | | | | | | | 189 | | | | | | | | | | | 1 | | | | | | | | | | | --- | | | | | | | | | | | R.KEDLPK.Y | | | | | | |  | | | 800.2704 | | | | | | | | | | 799.2631 | | | | | | | | | | 799.4188 | | | | | | | | | | | -194.76 | | | | | | | | | | | 111 | | | | | | | | | | | | - | | | | | | | | | | 118 | | | | | | | | | | | 0 | | | | | | | | | | | --- | | | | | | | | | | | R.TAGIPDAR.T | | | | | | |  | | | 848.3126 | | | | | | | | | | 847.3053 | | | | | | | | | | 847.4803 | | | | | | | | | | | -206.57 | | | | | | | | | | | 49 | | | | | | | | | | | | - | | | | | | | | | | 55 | | | | | | | | | | | 0 | | | | | | | | | | | --- | | | | | | | | | | | K.NIFLDVK.K | | | | | | |  | | | 851.3380 | | | | | | | | | | 850.3307 | | | | | | | | | | 850.5164 | | | | | | | | | | | -218.28 | | | | | | | | | | | 119 | | | | | | | | | | | | - | | | | | | | | | | 125 | | | | | | | | | | | 0 | | | | | | | | | | | --- | | | | | | | | | | | R.TYLLTLK.E | | | | | | |  | | | 958.3372 | | | | | | | | | | 957.3299 | | | | | | | | | | 957.5495 | | | | | | | | | | | -229.30 | | | | | | | | | | | 201 | | | | | | | | | | | | - | | | | | | | | | | 208 | | | | | | | | | | | 1 | | | | | | | | | | | --- | | | | | | | | | | | K.AQLEELKK.D | | | | | | |  | | | 966.3422 | | | | | | | | | | 965.3349 | | | | | | | | | | 965.5182 | | | | | | | | | | | -189.77 | | | | | | | | | | | 103 | | | | | | | | | | | | - | | | | | | | | | | 110 | | | | | | | | | | | 0 | | | | | | | | | | | --- | | | | | | | | | | | R.IAYTIETR.T | | | | | | |  | | | 966.3422 | | | | | | | | | | 965.3349 | | | | | | | | | | 965.5182 | | | | | | | | | | | -189.77 | | | | | | | | | | | 103 | | | | | | | | | | | | - | | | | | | | | | | 110 | | | | | | | | | | | 0 | | | | | | | | | | | 63 | | | | | | | | | | | R.IAYTIETR.T | | | | | | |  | | | 1005.3839 | | | | | | | | | | 1004.3766 | | | | | | | | | | 1004.5655 | | | | | | | | | | | -187.99 | | | | | | | | | | | 58 | | | | | | | | | | | | - | | | | | | | | | | 66 | | | | | | | | | | | 0 | | | | | | | | | | | --- | | | | | | | | | | | K.FETALGVLR.K | | | | | | |  | | | 1005.3839 | | | | | | | | | | 1004.3766 | | | | | | | | | | 1004.5655 | | | | | | | | | | | -187.99 | | | | | | | | | | | 58 | | | | | | | | | | | | - | | | | | | | | | | 66 | | | | | | | | | | | 0 | | | | | | | | | | | 62 | | | | | | | | | | | K.FETALGVLR.K | | | | | | |  | | | 1017.3771 | | | | | | | | | | 1016.3698 | | | | | | | | | | 1016.5437 | | | | | | | | | | | -171.02 | | | | | | | | | | | 156 | | | | | | | | | | | | - | | | | | | | | | | 163 | | | | | | | | | | | 1 | | | | | | | | | | | --- | | | | | | | | | | | K.KPLMRNDK.K + Oxidation (M) | | | | | | |  | | | 1051.3389 | | | | | | | | | | 1050.3316 | | | | | | | | | | 1050.5233 | | | | | | | | | | | -182.46 | | | | | | | | | | | 190 | | | | | | | | | | | | - | | | | | | | | | | 197 | | | | | | | | | | | 0 | | | | | | | | | | | --- | | | | | | | | | | | K.YEEQLELK.I | | | | | | |  | | | 1052.3071 | | | | | | | | | | 1051.2998 | | | | | | | | | | 1051.4934 | | | | | | | | | | | -184.12 | | | | | | | | | | | 94 | | | | | | | | | | | | - | | | | | | | | | | 102 | | | | | | | | | | | 0 | | | | | | | | | | | --- | | | | | | | | | | | K.ADLFSESQR.I | | | | | | |  | | | 1052.3071 | | | | | | | | | | 1051.2998 | | | | | | | | | | 1051.4934 | | | | | | | | | | | -184.12 | | | | | | | | | | | 94 | | | | | | | | | | | | - | | | | | | | | | | 102 | | | | | | | | | | | 0 | | | | | | | | | | | 62 | | | | | | | | | | | K.ADLFSESQR.I | | | | | | |  | | | 1133.4603 | | | | | | | | | | 1132.4530 | | | | | | | | | | 1132.6604 | | | | | | | | | | | -183.11 | | | | | | | | | | | 58 | | | | | | | | | | | | - | | | | | | | | | | 67 | | | | | | | | | | | 1 | | | | | | | | | | | --- | | | | | | | | | | | K.FETALGVLRK.E | | | | | | |  | | | 1133.4603 | | | | | | | | | | 1132.4530 | | | | | | | | | | 1132.6604 | | | | | | | | | | | -183.11 | | | | | | | | | | | 57 | | | | | | | | | | | | - | | | | | | | | | | 66 | | | | | | | | | | | 1 | | | | | | | | | | | 96 | | | | | | | | | | | K.KFETALGVLR.K | | | | | | |  | | | 1151.3328 | | | | | | | | | | 1150.3255 | | | | | | | | | | 1150.5176 | | | | | | | | | | | -166.93 | | | | | | | | | | | 209 | | | | | | | | | | | | - | | | | | | | | | | 218 | | | | | | | | | | | 0 | | | | | | | | | | | --- | | | | | | | | | | | K.DALEAMETQK.K + Oxidation (M) | | | | | | |  | | | 1211.3856 | | | | | | | | | | 1210.3783 | | | | | | | | | | 1210.5904 | | | | | | | | | | | -175.15 | | | | | | | | | | | 165 | | | | | | | | | | | | - | | | | | | | | | | 175 | | | | | | | | | | | 0 | | | | | | | | | | | --- | | | | | | | | | | | K.GMALLTAEFDK.I + Oxidation (M) | | | | | | |  | | | 1274.4663 | | | | | | | | | | 1273.4590 | | | | | | | | | | 1273.6812 | | | | | | | | | | | -174.47 | | | | | | | | | | | 13 | | | | | | | | | | | | - | | | | | | | | | | 23 | | | | | | | | | | | 0 | | | | | | | | | | | --- | | | | | | | | | | | K.QLCSSQVILQR.Q | | | | | | |  | | | 1309.4021 | | | | | | | | | | 1308.3948 | | | | | | | | | | 1308.6310 | | | | | | | | | | | -180.45 | | | | | | | | | | | 92 | | | | | | | | | | | | - | | | | | | | | | | 102 | | | | | | | | | | | 1 | | | | | | | | | | | --- | | | | | | | | | | | R.EKADLFSESQR.I | | | | | | |  | | | 1309.4021 | | | | | | | | | | 1308.3948 | | | | | | | | | | 1308.6310 | | | | | | | | | | | -180.45 | | | | | | | | | | | 92 | | | | | | | | | | | | - | | | | | | | | | | 102 | | | | | | | | | | | 1 | | | | | | | | | | | 102 | | | | | | | | | | | R.EKADLFSESQR.I | | | | | | |  | | | 1339.4309 | | | | | | | | | | 1338.4236 | | | | | | | | | | 1338.6853 | | | | | | | | | | | -195.49 | | | | | | | | | | | 164 | | | | | | | | | | | | - | | | | | | | | | | 175 | | | | | | | | | | | 1 | | | | | | | | | | | --- | | | | | | | | | | | K.KGMALLTAEFDK.I + Oxidation (M) | | | | | | |  | | | 1527.4061 | | | | | | | | | | 1526.3988 | | | | | | | | | | 1526.6810 | | | | | | | | | | | -184.84 | | | | | | | | | | | 221 | | | | | | | | | | | | - | | | | | | | | | | 232 | | | | | | | | | | | 1 | | | | | | | | | | | --- | | | | | | | | | | | R.EEFKDEEMVEVK.S + Oxidation (M) | | | | | | |  | | | 1564.4456 | | | | | | | | | | 1563.4383 | | | | | | | | | | 1563.7450 | | | | | | | | | | | -196.13 | | | | | | | | | | | 225 | | | | | | | | | | | | - | | | | | | | | | | 237 | | | | | | | | | | | 1 | | | | | | | | | | | --- | | | | | | | | | | | K.DEEMVEVKSLDVR.N + Oxidation (M) | | | | | | |  | | | 1747.5808 | | | | | | | | | | 1746.5735 | | | | | | | | | | 1746.9264 | | | | | | | | | | | -202.01 | | | | | | | | | | | 103 | | | | | | | | | | | | - | | | | | | | | | | 118 | | | | | | | | | | | 1 | | | | | | | | | | | --- | | | | | | | | | | | R.IAYTIETRTAGIPDAR.T | | | | | | |  | | | 2033.6212 | | | | | | | | | | 2032.6139 | | | | | | | | | | 2033.0139 | | | | | | | | | | | -196.75 | | | | | | | | | | | 70 | | | | | | | | | | | | - | | | | | | | | | | 88 | | | | | | | | | | | 0 | | | | | | | | | | | --- | | | | | | | | | | | K.ITIAPEDPAAVSQYANVMK.T + Oxidation (M) | | | | | | |  | | | 2033.6212 | | | | | | | | | | 2032.6139 | | | | | | | | | | 2033.0139 | | | | | | | | | | | -196.75 | | | | | | | | | | | 70 | | | | | | | | | | | | - | | | | | | | | | | 88 | | | | | | | | | | | 0 | | | | | | | | | | | 56 | | | | | | | | | | | K.ITIAPEDPAAVSQYANVMK.T + Oxidation (M) | | | | | | |  | | | 2350.5923 | | | | | | | | | | 2349.5850 | | | | | | | | | | 2350.0491 | | | | | | | | | | | -197.46 | | | | | | | | | | | 132 | | | | | | | | | | | | - | | | | | | | | | | 152 | | | | | | | | | | | 1 | | | | | | | | | | | --- | | | | | | | | | | | R.GLIDEHGAEAMMMDALDKVEK.E + 3 Oxidation (M) | | | | | | |  | | | No match to: 700.2562, 705.2466, 707.2043, 712.1694, 713.2599, 716.2371, 717.2396, 728.2374, 734.2974, 750.2690, 768.3586, 781.2300, 788.2472, 808.2286, 824.2355, 833.2919, 836.3090, 842.3460, 860.9056, 868.3568, 869.2958, 870.3003, 870.3003, 882.3648, 886.2716, 892.2811, 914.2520, 937.2547, 938.2479, 948.3046, 951.3242, 987.3333, 990.3456, 1004.3281, 1012.3533, 1019.4016, 1027.3529, 1034.3156, 1059.3550, 1074.3016, 1103.3889, 1114.3960, 1118.4509, 1155.4507, 1164.3866, 1165.4058, 1177.3977, 1187.4026, 1227.3801, 1273.4775, 1291.4163, 1308.3942, 1315.4142, 1331.3986, 1361.4694, 1361.4694, 1389.3774, 1461.4984, 1542.4072, 1606.5626, 1642.4635, 1683.4807, 1701.5225, 1793.5883, 1796.5675, 1809.6200, 1811.5686, 1811.5686, 1827.5444, 1858.6486, 1858.6486, 1937.6895, 1944.5363, 2001.6401, 2008.4766, 2024.4943, 2049.6025, 2055.6213, 2071.5906, 2077.5864, 2164.5688, 2180.5808, 2238.7251 | | | | | | | | | | | | | | | | | | | | | | | | | | | | | | | | | | | | | | | | | | | | | | | | | | | | | | | | | | | | | | | | | | | | | | | | | | | | | | | | | | | | | | | | | | | | | | | | | | | | | | |  | | | | **S47** [**Cs3g11320.1**](http://zhangyang-pc/mascot/cgi/protein_view.pl?file=../data/20140118/F012169.dat&hit=1) **Mass: 33497 Score: 393 Expect: 2.2e-035 Matches: 19** | | | | | | | | | | | | | | | | | | | | | | | | | | | | | | | | | | | | | | | | | | | | | | | | | | | | | | | | | | | | | | | | | | | | | | | | | | | | | | | | | | | | | | | | | | | | | | | | | | | | | | |  | | | | Observed | | | | | | | | | | Mr(expt) | | | | | | | | | | | Mr(calc) | | | | | | | | | | | ppm | | | | | | | | | | | Start | | | | | | | | | | | |  | | | | | | | | | | End | | | | | | | | | | | Miss | | | | | | | | | | | Ions | | | | | | | | | | | Peptide | | | | | | | | | 747.1698 | | | | | | | | | | 746.1625 | | | | | | | | | | | 746.3810 | | | | | | | | | | | -292.73 | | | | | | | | | | | 303 | | | | | | | | | | | | - | | | | | | | | | | 309 | | | | | | | | | | | 1 | | | | | | | | | | | --- | | | | | | | | | | | K.AVEKETA.- | | | | | | | | | 910.3302 | | | | | | | | | | 909.3229 | | | | | | | | | | | 909.5032 | | | | | | | | | | | -198.22 | | | | | | | | | | | 28 | | | | | | | | | | | | - | | | | | | | | | | 35 | | | | | | | | | | | 0 | | | | | | | | | | | --- | | | | | | | | | | | K.DALVSHLR.S | | | | | | | | | 910.3302 | | | | | | | | | | 909.3229 | | | | | | | | | | | 909.5032 | | | | | | | | | | | -198.22 | | | | | | | | | | | 28 | | | | | | | | | | | | - | | | | | | | | | | 35 | | | | | | | | | | | 0 | | | | | | | | | | | 45 | | | | | | | | | | | K.DALVSHLR.S | | | | | | | | | 959.3382 | | | | | | | | | | 958.3309 | | | | | | | | | | | 958.5083 | | | | | | | | | | | -185.10 | | | | | | | | | | | 211 | | | | | | | | | | | | - | | | | | | | | | | 219 | | | | | | | | | | | 0 | | | | | | | | | | | --- | | | | | | | | | | | R.ESPTSAIVR.F | | | | | | | | | 1138.3356 | | | | | | | | | | 1137.3283 | | | | | | | | | | | 1137.5410 | | | | | | | | | | | -186.93 | | | | | | | | | | | 162 | | | | | | | | | | | | - | | | | | | | | | | 171 | | | | | | | | | | | 0 | | | | | | | | | | | --- | | | | | | | | | | | K.SMTEMPLIGK.N + 2 Oxidation (M) | | | | | | | | | 1242.3234 | | | | | | | | | | 1241.3161 | | | | | | | | | | | 1241.5372 | | | | | | | | | | | -178.04 | | | | | | | | | | | 60 | | | | | | | | | | | | - | | | | | | | | | | 71 | | | | | | | | | | | 0 | | | | | | | | | | | --- | | | | | | | | | | | R.VSSSDSSDTTTR.G | | | | | | | | | 1291.4508 | | | | | | | | | | 1290.4435 | | | | | | | | | | | 1290.6819 | | | | | | | | | | | -184.73 | | | | | | | | | | | 15 | | | | | | | | | | | | - | | | | | | | | | | 27 | | | | | | | | | | | 0 | | | | | | | | | | | --- | | | | | | | | | | | K.IIAGADSFGAELK.D | | | | | | | | | 1291.4508 | | | | | | | | | | 1290.4435 | | | | | | | | | | | 1290.6819 | | | | | | | | | | | -184.73 | | | | | | | | | | | 15 | | | | | | | | | | | | - | | | | | | | | | | 27 | | | | | | | | | | | 0 | | | | | | | | | | | 42 | | | | | | | | | | | K.IIAGADSFGAELK.D | | | | | | | | | 1368.3799 | | | | | | | | | | 1367.3726 | | | | | | | | | | | 1367.6285 | | | | | | | | | | | -187.10 | | | | | | | | | | | 275 | | | | | | | | | | | | - | | | | | | | | | | 284 | | | | | | | | | | | 0 | | | | | | | | | | | --- | | | | | | | | | | | K.YYEETEFFIK.W | | | | | | | | | 1368.3799 | | | | | | | | | | 1367.3726 | | | | | | | | | | | 1367.6285 | | | | | | | | | | | -187.10 | | | | | | | | | | | 275 | | | | | | | | | | | | - | | | | | | | | | | 284 | | | | | | | | | | | 0 | | | | | | | | | | | 72 | | | | | | | | | | | K.YYEETEFFIK.W | | | | | | | | | 1398.3616 | | | | | | | | | | 1397.3543 | | | | | | | | | | | 1397.6383 | | | | | | | | | | | -203.16 | | | | | | | | | | | 59 | | | | | | | | | | | | - | | | | | | | | | | 71 | | | | | | | | | | | 1 | | | | | | | | | | | --- | | | | | | | | | | | R.RVSSSDSSDTTTR.G | | | | | | | | | 1407.4274 | | | | | | | | | | 1406.4201 | | | | | | | | | | | 1406.6976 | | | | | | | | | | | -197.24 | | | | | | | | | | | 1 | | | | | | | | | | | | - | | | | | | | | | | 14 | | | | | | | | | | | 0 | | | | | | | | | | | --- | | | | | | | | | | | -.MAENAAAAAPHPLK.I + Oxidation (M) | | | | | | | | | 1533.4584 | | | | | | | | | | 1532.4511 | | | | | | | | | | | 1532.7215 | | | | | | | | | | | -176.37 | | | | | | | | | | | 194 | | | | | | | | | | | | - | | | | | | | | | | 207 | | | | | | | | | | | 0 | | | | | | | | | | | --- | | | | | | | | | | | R.ELNPVEMIPGGSMK.I + 2 Oxidation (M) | | | | | | | | | 2022.6187 | | | | | | | | | | 2021.6114 | | | | | | | | | | | 2021.9847 | | | | | | | | | | | -184.62 | | | | | | | | | | | 255 | | | | | | | | | | | | - | | | | | | | | | | 272 | | | | | | | | | | | 0 | | | | | | | | | | | --- | | | | | | | | | | | R.FDLTVGDYLFTPAGDVHR.V | | | | | | | | | 2022.6187 | | | | | | | | | | 2021.6114 | | | | | | | | | | | 2021.9847 | | | | | | | | | | | -184.62 | | | | | | | | | | | 255 | | | | | | | | | | | | - | | | | | | | | | | 272 | | | | | | | | | | | 0 | | | | | | | | | | | 85 | | | | | | | | | | | R.FDLTVGDYLFTPAGDVHR.V | | | | | | | | | 2335.7395 | | | | | | | | | | 2334.7322 | | | | | | | | | | | 2335.1822 | | | | | | | | | | | -192.69 | | | | | | | | | | | 222 | | | | | | | | | | | | - | | | | | | | | | | 243 | | | | | | | | | | | 0 | | | | | | | | | | | --- | | | | | | | | | | | K.AGSVEPAHHHTFGHDLVVLQGK.K | | | | | | | | | 2335.7395 | | | | | | | | | | 2334.7322 | | | | | | | | | | | 2335.1822 | | | | | | | | | | | -192.69 | | | | | | | | | | | 222 | | | | | | | | | | | | - | | | | | | | | | | 243 | | | | | | | | | | | 0 | | | | | | | | | | | 83 | | | | | | | | | | | K.AGSVEPAHHHTFGHDLVVLQGK.K | | | | | | | | | 2373.6763 | | | | | | | | | | 2372.6690 | | | | | | | | | | | 2372.0606 | | | | | | | | | | | 257 | | | | | | | | | | | 141 | | | | | | | | | | | | - | | | | | | | | | | 161 | | | | | | | | | | | 0 | | | | | | | | | | | --- | | | | | | | | | | | K.APCPASGFKPWEENISCFFDK.S | | | | | | | | | 2472.7271 | | | | | | | | | | 2471.7198 | | | | | | | | | | | 2472.1656 | | | | | | | | | | | -180.33 | | | | | | | | | | | 36 | | | | | | | | | | | | - | | | | | | | | | | 58 | | | | | | | | | | | 0 | | | | | | | | | | | --- | | | | | | | | | | | R.SLNIDVEDLGTSDYYSIGAEVGR.R | | | | | | | | | No match to: 700.2103, 703.1447, 713.2703, 716.2177, 737.2570, 768.3740, 771.2465, 842.3341, 850.3539, 851.3022, 868.3727, 879.2763, 882.3936, 897.2361, 905.3000, 958.3447, 972.3062, 974.3556, 1027.3364, 1051.4885, 1059.3717, 1133.3958, 1151.4824, 1232.4055, 1244.3861, 1255.3221, 1286.4038, 1287.4667, 1299.4541, 1299.4541, 1300.4926, 1302.4954, 1313.4464, 1315.4539, 1321.4344, 1324.4186, 1334.4486, 1390.3622, 1406.3480, 1416.4010, 1419.4576, 1432.4042, 1434.4283, 1451.4341, 1465.4609, 1469.4460, 1487.5079, 1496.4338, 1542.5563, 1551.5541, 1551.5541, 1555.4814, 1567.5393, 1572.4205, 1583.5317, 1608.6078, 1619.5790, 1626.6200, 1626.6200, 1636.3827, 1648.3694, 1701.5500, 1719.5852, 1719.5852, 1770.5690, 1812.6296, 1865.4861, 1901.5848, 1923.5927, 1926.4232, 1927.6406, 1927.6406, 1976.5934, 2002.6006, 2005.6191, 2009.5546, 2011.6559, 2016.6027, 2019.6349, 2020.6608, 2034.5360, 2044.5964, 2096.6521, 2105.6289, 2112.7295, 2224.7327, 2238.6938, 2245.7783, 2334.7297, 2357.7239, 2392.7456 | | | | | | | | | | | | | | | | | | | | | | | | | | | | | | | | | | | | | | | | | | | | | | | | | | | | | | | | | | | | | | | | | | | | | | | | | | | | | | | | | | | | | | | | | | | | | | | | | | | | | | |  | | | | **S40** [**Cs1g06710.1**](http://zhangyang-pc/mascot/cgi/protein_view.pl?file=../data/20140118/F012161.dat&hit=1) **Mass: 50360 Score: 579 Expect: 5.6e-054 Matches: 21** | | | | | | | | | | | | | | | | | | | | | | | | | | | | | | | | | | | | | | | | | | | | | | | | | | | | | | | | | | | | | | | | | | | | | | | | | | | | | | | | | | | | | | | | | | | | | | | | | | | | | | |  | | | | Observed | | | | | | | | Mr(expt) | | | | | | | | | | Mr(calc) | | | | | | | | | | | ppm | | | | | | | | | | | Start | | | | | | | | | | | |  | | | | | | | | | | End | | | | | | | | | | | Miss | | | | | | | | | | | Ions | | | | | | | | | | | Peptide | | | | | | | | | | | | 759.2836 | | | | | | | | 758.2764 | | | | | | | | | | 758.4286 | | | | | | | | | | | -200.78 | | | | | | | | | | | 127 | | | | | | | | | | | | - | | | | | | | | | | 133 | | | | | | | | | | | 0 | | | | | | | | | | | --- | | | | | | | | | | | R.TGELALR.R | | | | | | | | | | | | 826.2988 | | | | | | | | 825.2916 | | | | | | | | | | 825.4960 | | | | | | | | | | | -247.65 | | | | | | | | | | | 431 | | | | | | | | | | | | - | | | | | | | | | | 437 | | | | | | | | | | | 0 | | | | | | | | | | | --- | | | | | | | | | | | R.DILPQIK.T | | | | | | | | | | | | 830.2731 | | | | | | | | 829.2658 | | | | | | | | | | 829.4294 | | | | | | | | | | | -197.21 | | | | | | | | | | | 447 | | | | | | | | | | | | - | | | | | | | | | | 453 | | | | | | | | | | | 0 | | | | | | | | | | | --- | | | | | | | | | | | K.LIEGQDR.L | | | | | | | | | | | | 1001.3858 | | | | | | | | 1000.3785 | | | | | | | | | | 1000.5804 | | | | | | | | | | | -201.80 | | | | | | | | | | | 266 | | | | | | | | | | | | - | | | | | | | | | | 274 | | | | | | | | | | | 0 | | | | | | | | | | | --- | | | | | | | | | | | R.GIVELTIEK.G | | | | | | | | | | | | 1179.3801 | | | | | | | | 1178.3728 | | | | | | | | | | 1178.5932 | | | | | | | | | | | -186.94 | | | | | | | | | | | 313 | | | | | | | | | | | | - | | | | | | | | | | 323 | | | | | | | | | | | 0 | | | | | | | | | | | --- | | | | | | | | | | | K.LVIDGAYDGTR.L | | | | | | | | | | | | 1179.3801 | | | | | | | | 1178.3728 | | | | | | | | | | 1178.5932 | | | | | | | | | | | -186.94 | | | | | | | | | | | 313 | | | | | | | | | | | | - | | | | | | | | | | 323 | | | | | | | | | | | 0 | | | | | | | | | | | 101 | | | | | | | | | | | K.LVIDGAYDGTR.L | | | | | | | | | | | | 1224.4078 | | | | | | | | 1223.4005 | | | | | | | | | | 1223.6398 | | | | | | | | | | | -195.51 | | | | | | | | | | | 197 | | | | | | | | | | | | - | | | | | | | | | | 208 | | | | | | | | | | | 0 | | | | | | | | | | | --- | | | | | | | | | | | K.GSTLYASLIDGK.G | | | | | | | | | | | | 1242.4297 | | | | | | | | 1241.4224 | | | | | | | | | | 1241.6867 | | | | | | | | | | | -212.83 | | | | | | | | | | | 183 | | | | | | | | | | | | - | | | | | | | | | | 194 | | | | | | | | | | | 0 | | | | | | | | | | | --- | | | | | | | | | | | K.DSILASIPADLK.E | | | | | | | | | | | | 1310.4332 | | | | | | | | 1309.4259 | | | | | | | | | | 1309.7394 | | | | | | | | | | | -239.35 | | | | | | | | | | | 2 | | | | | | | | | | | | - | | | | | | | | | | 13 | | | | | | | | | | | 0 | | | | | | | | | | | --- | | | | | | | | | | | M.AFTLSSPILPHK.L | | | | | | | | | | | | 1449.3535 | | | | | | | | 1448.3462 | | | | | | | | | | 1448.6168 | | | | | | | | | | | -186.79 | | | | | | | | | | | 275 | | | | | | | | | | | | - | | | | | | | | | | 289 | | | | | | | | | | | 0 | | | | | | | | | | | --- | | | | | | | | | | | K.GDGSSFSPEAGGEPR.K | | | | | | | | | | | | 1449.3535 | | | | | | | | 1448.3462 | | | | | | | | | | 1448.6168 | | | | | | | | | | | -186.79 | | | | | | | | | | | 275 | | | | | | | | | | | | - | | | | | | | | | | 289 | | | | | | | | | | | 0 | | | | | | | | | | | 139 | | | | | | | | | | | K.GDGSSFSPEAGGEPR.K | | | | | | | | | | | | 1575.5806 | | | | | | | | 1574.5733 | | | | | | | | | | 1574.8668 | | | | | | | | | | | -186.33 | | | | | | | | | | | 144 | | | | | | | | | | | | - | | | | | | | | | | 157 | | | | | | | | | | | 0 | | | | | | | | | | | --- | | | | | | | | | | | K.AIQASLEDISFLLR.I | | | | | | | | | | | | 1575.5806 | | | | | | | | 1574.5733 | | | | | | | | | | 1574.8668 | | | | | | | | | | | -186.33 | | | | | | | | | | | 144 | | | | | | | | | | | | - | | | | | | | | | | 157 | | | | | | | | | | | 0 | | | | | | | | | | | 118 | | | | | | | | | | | K.AIQASLEDISFLLR.I | | | | | | | | | | | | 1945.6257 | | | | | | | | 1944.6184 | | | | | | | | | | 1945.0078 | | | | | | | | | | | -200.17 | | | | | | | | | | | 177 | | | | | | | | | | | | - | | | | | | | | | | 194 | | | | | | | | | | | 1 | | | | | | | | | | | --- | | | | | | | | | | | K.IAMDEKDSILASIPADLK.E + Oxidation (M) | | | | | | | | | | | | 1945.6257 | | | | | | | | 1944.6184 | | | | | | | | | | 1945.0078 | | | | | | | | | | | -200.17 | | | | | | | | | | | 177 | | | | | | | | | | | | - | | | | | | | | | | 194 | | | | | | | | | | | 1 | | | | | | | | | | | 43 | | | | | | | | | | | K.IAMDEKDSILASIPADLK.E + Oxidation (M) | | | | | | | | | | | | 2188.7495 | | | | | | | | 2187.7422 | | | | | | | | | | 2188.1739 | | | | | | | | | | | -197.30 | | | | | | | | | | | 291 | | | | | | | | | | | | - | | | | | | | | | | 312 | | | | | | | | | | | 0 | | | | | | | | | | | --- | | | | | | | | | | | K.TATIQVVLDGYSAPLTAGNVAK.L | | | | | | | | | | | | 2426.7217 | | | | | | | | 2425.7144 | | | | | | | | | | 2425.1624 | | | | | | | | | | | 228 | | | | | | | | | | | 340 | | | | | | | | | | | | - | | | | | | | | | | 360 | | | | | | | | | | | 0 | | | | | | | | | | | --- | | | | | | | | | | | K.GSDYNLPLEIMPSGQFEPLYR.T | | | | | | | | | | | | 2441.6956 | | | | | | | | 2440.6883 | | | | | | | | | | 2441.1573 | | | | | | | | | | | -192.11 | | | | | | | | | | | 340 | | | | | | | | | | | | - | | | | | | | | | | 360 | | | | | | | | | | | 0 | | | | | | | | | | | --- | | | | | | | | | | | K.GSDYNLPLEIMPSGQFEPLYR.T + Oxidation (M) | | | | | | | | | | | | 2441.6956 | | | | | | | | 2440.6883 | | | | | | | | | | 2441.1573 | | | | | | | | | | | -192.11 | | | | | | | | | | | 340 | | | | | | | | | | | | - | | | | | | | | | | 360 | | | | | | | | | | | 0 | | | | | | | | | | | 67 | | | | | | | | | | | K.GSDYNLPLEIMPSGQFEPLYR.T + Oxidation (M) | | | | | | | | | | | | 2478.7322 | | | | | | | | 2477.7249 | | | | | | | | | | 2478.1816 | | | | | | | | | | | -184.28 | | | | | | | | | | | 407 | | | | | | | | | | | | - | | | | | | | | | | 430 | | | | | | | | | | | 0 | | | | | | | | | | | --- | | | | | | | | | | | R.NAGLGGLSFDEGQFSVFGYTTVGR.D | | | | | | | | | | | | 2478.7322 | | | | | | | | 2477.7249 | | | | | | | | | | 2478.1816 | | | | | | | | | | | -184.28 | | | | | | | | | | | 407 | | | | | | | | | | | | - | | | | | | | | | | 430 | | | | | | | | | | | 0 | | | | | | | | | | | 63 | | | | | | | | | | | R.NAGLGGLSFDEGQFSVFGYTTVGR.D | | | | | | | | | | | | No match to: 700.2463, 713.2738, 716.2258, 724.3449, 730.2484, 733.2501, 734.3203, 750.3704, 765.1982, 768.3671, 773.2867, 842.3422, 848.2875, 850.3450, 864.2478, 868.3634, 882.3785, 908.2284, 956.2742, 982.3401, 991.4000, 997.2917, 1016.3271, 1051.4980, 1059.3695, 1072.3755, 1109.3560, 1140.3578, 1151.4822, 1155.3850, 1165.4330, 1193.3834, 1239.3755, 1244.4093, 1277.4259, 1299.4468, 1320.3759, 1331.3906, 1334.5189, 1365.3981, 1380.4003, 1382.4861, 1396.4061, 1413.4025, 1418.4729, 1428.4026, 1431.3704, 1431.3704, 1434.4481, 1463.3757, 1471.3622, 1475.4835, 1479.4595, 1487.3871, 1519.4862, 1589.5557, 1597.5687, 1613.5396, 1632.4664, 1649.4585, 1724.5997, 1753.4969, 1791.4269, 1795.5582, 1820.5264, 1865.4821, 1881.5564, 1896.5156, 1913.6130, 1961.6193, 1993.6224, 2002.6497, 2018.6354, 2220.7129, 2224.7075, 2238.7061, 2238.7061, 2245.7751, 2377.7415, 2377.7415, 2383.5164, 2423.6970, 2457.7083, 2457.7083, 2467.7783, 2484.6917, 2666.7246, 2694.7937, 2810.7764 | | | | | | | | | | | | | | | | | | | | | | | | | | | | | | | | | | | | | | | | | | | | | | | | | | | | | | | | | | | | | | | | | | | | | | | | | | | | | | | | | | | | | | | | | | | | | | | | | | | | | | |  | | | | **S43** [**Cs3g26890.2**](http://zhangyang-pc/mascot/cgi/protein_view.pl?file=../data/20140118/F012131.dat&hit=2) **Mass: 57159 Score: 226 Expect: 1.1e-018 Matches: 22** | | | | | | | | | | | | | | | | | | | | | | | | | | | | | | | | | | | | | | | | | | | | | | | | | | | | | | | | | | | | | | | | | | | | | | | | | | | | | | | | | | | | | | | | | | | | | | | | | | | | | | |  | | | | Observed | | | | | Mr(expt) | | | | | | | | | | Mr(calc) | | | | | | | | | | | ppm | | | | | | | | | | | Start | | | | | | | | | | |  | | | | | | | | | | | End | | | | | | | | | | | Miss | | | | | | | | | | | Ions | | | | | | | | | | | Peptide | | | | | | | | | | | | |  | | 730.2411 | | | | | 729.2338 | | | | | | | | | | 729.4133 | | | | | | | | | | | -246.07 | | | | | | | | | | | 226 | | | | | | | | | | | - | | | | | | | | | | | 231 | | | | | | | | | | | 1 | | | | | | | | | | | --- | | | | | | | | | | | K.RIENAK.I | | | | | | | | | | | | |  | | 831.2537 | | | | | 830.2464 | | | | | | | | | | 830.4134 | | | | | | | | | | | -201.02 | | | | | | | | | | | 415 | | | | | | | | | | | - | | | | | | | | | | | 421 | | | | | | | | | | | 0 | | | | | | | | | | | --- | | | | | | | | | | | K.EVDELAR.K | | | | | | | | | | | | |  | | 834.3171 | | | | | 833.3098 | | | | | | | | | | 833.3403 | | | | | | | | | | | -36.54 | | | | | | | | | | | 9 | | | | | | | | | | | - | | | | | | | | | | | 15 | | | | | | | | | | | 0 | | | | | | | | | | | --- | | | | | | | | | | | K.DEANEEK.G | | | | | | | | | | | | |  | | 868.3649 | | | | | 867.3577 | | | | | | | | | | 867.4524 | | | | | | | | | | | -109.23 | | | | | | | | | | | 150 | | | | | | | | | | | - | | | | | | | | | | | 156 | | | | | | | | | | | 1 | | | | | | | | | | | --- | | | | | | | | | | | K.FKSDLMK.I | | | | | | | | | | | | |  | | 967.2684 | | | | | 966.2612 | | | | | | | | | | 966.5056 | | | | | | | | | | | -252.89 | | | | | | | | | | | 157 | | | | | | | | | | | - | | | | | | | | | | | 165 | | | | | | | | | | | 0 | | | | | | | | | | | --- | | | | | | | | | | | K.IAMTTLSSK.I + Oxidation (M) | | | | | | | | | | | | |  | | 1287.4485 | | | | | 1286.4412 | | | | | | | | | | 1286.6805 | | | | | | | | | | | -185.98 | | | | | | | | | | | 116 | | | | | | | | | | | - | | | | | | | | | | | 126 | | | | | | | | | | | 0 | | | | | | | | | | | --- | | | | | | | | | | | K.IHPMTIISGFR.M + Oxidation (M) | | | | | | | | | | | | |  | | 1287.4485 | | | | | 1286.4412 | | | | | | | | | | 1286.6805 | | | | | | | | | | | -185.98 | | | | | | | | | | | 116 | | | | | | | | | | | - | | | | | | | | | | | 126 | | | | | | | | | | | 0 | | | | | | | | | | | 20 | | | | | | | | | | | K.IHPMTIISGFR.M + Oxidation (M) | | | | | | | | | | | | |  | | 1302.4518 | | | | | 1301.4445 | | | | | | | | | | 1301.7191 | | | | | | | | | | | -210.90 | | | | | | | | | | | 187 | | | | | | | | | | | - | | | | | | | | | | | 198 | | | | | | | | | | | 0 | | | | | | | | | | | --- | | | | | | | | | | | K.GSTNLESIQIIK.K | | | | | | | | | | | | |  | | 1305.4199 | | | | | 1304.4126 | | | | | | | | | | 1304.6533 | | | | | | | | | | | -184.50 | | | | | | | | | | | 343 | | | | | | | | | | | - | | | | | | | | | | | 353 | | | | | | | | | | | 0 | | | | | | | | | | | --- | | | | | | | | | | | K.LIEEIMIGEDK.L + Oxidation (M) | | | | | | | | | | | | |  | | 1320.3977 | | | | | 1319.3904 | | | | | | | | | | 1319.6218 | | | | | | | | | | | -175.34 | | | | | | | | | | | 372 | | | | | | | | | | | - | | | | | | | | | | | 383 | | | | | | | | | | | 0 | | | | | | | | | | | --- | | | | | | | | | | | R.GASHHVLDEAER.S | | | | | | | | | | | | |  | | 1398.4246 | | | | | 1397.4173 | | | | | | | | | | 1397.6715 | | | | | | | | | | | -181.83 | | | | | | | | | | | 206 | | | | | | | | | | | - | | | | | | | | | | | 217 | | | | | | | | | | | 0 | | | | | | | | | | | --- | | | | | | | | | | | K.DSFLDEGFILDK.K | | | | | | | | | | | | |  | | 1488.4529 | | | | | 1487.4456 | | | | | | | | | | 1487.7191 | | | | | | | | | | | -183.82 | | | | | | | | | | | 172 | | | | | | | | | | | - | | | | | | | | | | | 184 | | | | | | | | | | | 0 | | | | | | | | | | | --- | | | | | | | | | | | K.EHFGQLAVDAVMR.L + Oxidation (M) | | | | | | | | | | | | |  | | 1526.4852 | | | | | 1525.4779 | | | | | | | | | | 1525.7664 | | | | | | | | | | | -189.08 | | | | | | | | | | | 206 | | | | | | | | | | | - | | | | | | | | | | | 218 | | | | | | | | | | | 1 | | | | | | | | | | | --- | | | | | | | | | | | K.DSFLDEGFILDKK.I | | | | | | | | | | | | |  | | 1555.4537 | | | | | 1554.4464 | | | | | | | | | | 1554.7786 | | | | | | | | | | | -213.64 | | | | | | | | | | | 21 | | | | | | | | | | | - | | | | | | | | | | | 35 | | | | | | | | | | | 0 | | | | | | | | | | | --- | | | | | | | | | | | R.MASFVGAMAIADLVK.T + 2 Oxidation (M) | | | | | | | | | | | | |  | | 1731.6066 | | | | | 1730.5993 | | | | | | | | | | 1730.9236 | | | | | | | | | | | -187.36 | | | | | | | | | | | 496 | | | | | | | | | | | - | | | | | | | | | | | 511 | | | | | | | | | | | 0 | | | | | | | | | | | --- | | | | | | | | | | | K.QAVLLSATEAAEMILR.V + Oxidation (M) | | | | | | | | | | | | |  | | 2047.6661 | | | | | 2046.6588 | | | | | | | | | | 2047.0473 | | | | | | | | | | | -189.80 | | | | | | | | | | | 318 | | | | | | | | | | | - | | | | | | | | | | | 337 | | | | | | | | | | | 0 | | | | | | | | | | | --- | | | | | | | | | | | R.LALVTGGEIASTFDNPESVK.L | | | | | | | | | | | | |  | | 2047.6661 | | | | | 2046.6588 | | | | | | | | | | 2047.0473 | | | | | | | | | | | -189.80 | | | | | | | | | | | 318 | | | | | | | | | | | - | | | | | | | | | | | 337 | | | | | | | | | | | 0 | | | | | | | | | | | 65 | | | | | | | | | | | R.LALVTGGEIASTFDNPESVK.L | | | | | | | | | | | | |  | | 2172.7017 | | | | | 2171.6944 | | | | | | | | | | 2172.0997 | | | | | | | | | | | -186.60 | | | | | | | | | | | 166 | | | | | | | | | | | - | | | | | | | | | | | 184 | | | | | | | | | | | 1 | | | | | | | | | | | --- | | | | | | | | | | | K.ILSQDKEHFGQLAVDAVMR.L + Oxidation (M) | | | | | | | | | | | | |  | | 2172.7017 | | | | | 2171.6944 | | | | | | | | | | 2172.0997 | | | | | | | | | | | -186.60 | | | | | | | | | | | 166 | | | | | | | | | | | - | | | | | | | | | | | 184 | | | | | | | | | | | 1 | | | | | | | | | | | 20 | | | | | | | | | | | K.ILSQDKEHFGQLAVDAVMR.L + Oxidation (M) | | | | | | | | | | | | |  | | 2271.7585 | | | | | 2270.7512 | | | | | | | | | | 2271.1595 | | | | | | | | | | | -179.75 | | | | | | | | | | | 85 | | | | | | | | | | | - | | | | | | | | | | | 106 | | | | | | | | | | | 0 | | | | | | | | | | | --- | | | | | | | | | | | K.VQDDEVGDGTTSVVVLAGELLR.E | | | | | | | | | | | | |  | | 2271.7585 | | | | | 2270.7512 | | | | | | | | | | 2271.1595 | | | | | | | | | | | -179.75 | | | | | | | | | | | 85 | | | | | | | | | | | - | | | | | | | | | | | 106 | | | | | | | | | | | 0 | | | | | | | | | | | 73 | | | | | | | | | | | K.VQDDEVGDGTTSVVVLAGELLR.E | | | | | | | | | | | | |  | | 2535.9553 | | | | | 2534.9480 | | | | | | | | | | 2535.3908 | | | | | | | | | | | -174.63 | | | | | | | | | | | 437 | | | | | | | | | | | - | | | | | | | | | | | 461 | | | | | | | | | | | 0 | | | | | | | | | | | --- | | | | | | | | | | | R.ALVAIPTTIADNAGLDSAELIAQLR.A | | | | | | | | | | | | |  | | No match to: 700.2438, 705.2061, 713.2642, 716.2258, 718.2011, 734.3325, 744.2372, 750.3581, 768.3587, 769.3398, 793.2327, 824.2618, 842.3362, 850.3564, 860.9028, 876.8820, 882.3733, 888.2828, 902.2641, 914.2403, 929.2951, 957.2543, 968.3221, 986.2891, 1004.3353, 1021.3326, 1051.4706, 1059.3601, 1082.3827, 1119.3492, 1151.4714, 1157.4016, 1157.4016, 1165.4810, 1182.4066, 1187.4235, 1188.4054, 1196.4005, 1238.4254, 1246.3268, 1250.3727, 1261.4901, 1261.4901, 1303.4528, 1319.4314, 1322.4578, 1329.3790, 1347.4585, 1368.4464, 1381.4824, 1394.4553, 1413.4701, 1421.4352, 1424.4199, 1434.5498, 1448.4911, 1456.4254, 1465.4956, 1465.4956, 1473.4293, 1487.4449, 1569.4513, 1576.5586, 1585.4131, 1592.5151, 1592.5151, 1618.5286, 1622.5017, 1675.4835, 1679.5369, 1680.5355, 1691.5178, 1714.5695, 1714.5695, 1760.4370, 1784.5181, 1804.4324, 1823.5920, 1835.5460, 1870.6917, 1984.5964, 2002.6749, 2085.6379, 2185.6138, 2189.7268, 2238.7229, 2238.7229, 2245.7712 | | | | | | | | | | | | | | | | | | | | | | | | | | | | | | | | | | | | | | | | | | | | | | | | | | | | | | | | | | | | | | | | | | | | | | | | | | | | | | | | | | | | | | | | | | | | | | | | | | | | | | |  | | | | **S41** [**Cs7g07630.1**](http://zhangyang-pc/mascot/cgi/protein_view.pl?file=../data/20140118/F012197.dat&hit=1) **Mass: 25558 Score: 254 Expect: 1.8e-021 Matches: 12** | | | | | | | | | | | | | | | | | | | | | | | | | | | | | | | | | | | | | | | | | | | | | | | | | | | | | | | | | | | | | | | | | | | | | | | | | | | | | | | | | | | | | | | | | | | | | | | | | | | | | | |  | | | | Observed | | | | | | | Mr(expt) | | | | | | | | | | Mr(calc) | | | | | | | | | | | ppm | | | | | | | | | | | Start | | | | | | | | | | | |  | | | | | | | | | | End | | | | | | | | | | | Miss | | | | | | | | | | | Ions | | | | | | | | | | | Peptide | | | | | | | | | | |  | | 742.3141 | | | | | | | 741.3068 | | | | | | | | | | 741.4497 | | | | | | | | | | | -192.72 | | | | | | | | | | | 93 | | | | | | | | | | | | - | | | | | | | | | | 99 | | | | | | | | | | | 0 | | | | | | | | | | | --- | | | | | | | | | | | K.VAANLVR.L | | | | | | | | | | |  | | 800.3396 | | | | | | | 799.3323 | | | | | | | | | | 799.4916 | | | | | | | | | | | -199.17 | | | | | | | | | | | 174 | | | | | | | | | | | | - | | | | | | | | | | 181 | | | | | | | | | | | 0 | | | | | | | | | | | --- | | | | | | | | | | | K.AVSLAIAR.D | | | | | | | | | | |  | | 817.2602 | | | | | | | 816.2529 | | | | | | | | | | 816.4090 | | | | | | | | | | | -191.18 | | | | | | | | | | | 182 | | | | | | | | | | | | - | | | | | | | | | | 190 | | | | | | | | | | | 0 | | | | | | | | | | | --- | | | | | | | | | | | R.DGASGGVVR.T | | | | | | | | | | |  | | 851.3058 | | | | | | | 850.2985 | | | | | | | | | | 850.4548 | | | | | | | | | | | -183.81 | | | | | | | | | | | 100 | | | | | | | | | | | | - | | | | | | | | | | 106 | | | | | | | | | | | 0 | | | | | | | | | | | --- | | | | | | | | | | | R.LLSYNNK.N | | | | | | | | | | |  | | 1067.3590 | | | | | | | 1066.3517 | | | | | | | | | | 1066.5407 | | | | | | | | | | | -177.21 | | | | | | | | | | | 35 | | | | | | | | | | | | - | | | | | | | | | | 44 | | | | | | | | | | | 0 | | | | | | | | | | | --- | | | | | | | | | | | R.TSTGVYVANR.A | | | | | | | | | | |  | | 1067.3590 | | | | | | | 1066.3517 | | | | | | | | | | 1066.5407 | | | | | | | | | | | -177.21 | | | | | | | | | | | 35 | | | | | | | | | | | | - | | | | | | | | | | 44 | | | | | | | | | | | 0 | | | | | | | | | | | 70 | | | | | | | | | | | R.TSTGVYVANR.A | | | | | | | | | | |  | | 1162.4031 | | | | | | | 1161.3958 | | | | | | | | | | 1161.5990 | | | | | | | | | | | -174.88 | | | | | | | | | | | 191 | | | | | | | | | | | | - | | | | | | | | | | 201 | | | | | | | | | | | 0 | | | | | | | | | | | --- | | | | | | | | | | | R.TVTINSEGVSR.K | | | | | | | | | | |  | | 1542.4349 | | | | | | | 1541.4276 | | | | | | | | | | 1541.6958 | | | | | | | | | | | -173.94 | | | | | | | | | | | 61 | | | | | | | | | | | | - | | | | | | | | | | 75 | | | | | | | | | | | 0 | | | | | | | | | | | --- | | | | | | | | | | | R.SGSAADSQTVSDYVR.Y | | | | | | | | | | |  | | 1542.4349 | | | | | | | 1541.4276 | | | | | | | | | | 1541.6958 | | | | | | | | | | | -173.94 | | | | | | | | | | | 61 | | | | | | | | | | | | - | | | | | | | | | | 75 | | | | | | | | | | | 0 | | | | | | | | | | | 86 | | | | | | | | | | | R.SGSAADSQTVSDYVR.Y | | | | | | | | | | |  | | 1980.7020 | | | | | | | 1979.6947 | | | | | | | | | | 1980.0581 | | | | | | | | | | | -183.54 | | | | | | | | | | | 76 | | | | | | | | | | | | - | | | | | | | | | | 92 | | | | | | | | | | | 0 | | | | | | | | | | | --- | | | | | | | | | | | R.YFLHQHTIQLGQPATVK.V | | | | | | | | | | |  | | 1980.7020 | | | | | | | 1979.6947 | | | | | | | | | | 1980.0581 | | | | | | | | | | | -183.54 | | | | | | | | | | | 76 | | | | | | | | | | | | - | | | | | | | | | | 92 | | | | | | | | | | | 0 | | | | | | | | | | | 59 | | | | | | | | | | | R.YFLHQHTIQLGQPATVK.V | | | | | | | | | | |  | | 2704.0215 | | | | | | | 2703.0142 | | | | | | | | | | 2703.4973 | | | | | | | | | | | -178.68 | | | | | | | | | | | 76 | | | | | | | | | | | | - | | | | | | | | | | 99 | | | | | | | | | | | 1 | | | | | | | | | | | --- | | | | | | | | | | | R.YFLHQHTIQLGQPATVKVAANLVR.L | | | | | | | | | | |  | | No match to: 700.2396, 713.2726, 716.2253, 734.3068, 750.3638, 768.3766, 812.2554, 823.2394, 834.3246, 837.2713, 842.3478, 848.3256, 850.3718, 859.2811, 860.9058, 868.3616, 882.3906, 899.2913, 916.3166, 931.3594, 931.3594, 955.2873, 996.3696, 1014.3046, 1017.4257, 1031.3259, 1051.4670, 1059.3762, 1076.3235, 1087.4183, 1088.3295, 1093.3524, 1105.3414, 1136.3390, 1148.3530, 1151.5070, 1165.4536, 1183.4613, 1183.4613, 1195.4591, 1277.4352, 1281.4351, 1293.4325, 1309.4307, 1315.4117, 1331.4125, 1333.3822, 1362.4420, 1374.4381, 1379.4404, 1385.3499, 1386.4015, 1390.4495, 1391.4572, 1397.3414, 1400.3988, 1401.3796, 1402.4469, 1404.5051, 1404.5051, 1405.0385, 1413.3447, 1433.4740, 1453.4644, 1478.4556, 1489.5233, 1495.4974, 1511.4419, 1521.5302, 1527.4895, 1533.4919, 1534.5432, 1534.5432, 1585.4644, 1597.4799, 1613.4745, 1625.5587, 1639.5166, 1651.5159, 1689.5624, 1761.5031, 1892.6697, 1892.6697, 1921.7113, 2002.6743, 2018.6486, 2072.6455, 2143.6008, 2214.6062, 2214.6062, 2220.7014, 2228.6274, 2234.6091, 2238.7002, 2245.7996, 2268.7100, 2319.6814, 2319.6814 | | | | | | | | | | | | | | | | | | | | | | | | | | | | | | | | | | | | | | | | | | | | | | | | | | | | | | | | | | | | | | | | | | | | | | | | | | | | | | | | | | | | | | | | | | | | | | | | | | | | | | |  | | | | **S44** [**Cs4g04180.1**](http://zhangyang-pc/mascot/cgi/protein_view.pl?file=../data/20140118/F012140.dat&hit=1)**Mass: 47020    Score: 367    Expect: 8.8e-033  Matches: 24** | | | | | | | | | | | | | | | | | | | | | | | | | | | | | | | | | | | | | | | | | | | | | | | | | | | | | | | | | | | | | | | | | | | | | | | | | | | | | | | | | | | | | | | | | | | | | | | | | | | | | | |  | | | | Observed | | Mr(expt) | | | | | | | | | | Mr(calc) | | | | | | | | | | | ppm | | | | | | | | | | | Start | | | | | | | | | | |  | | | | | | | | | | | End | | | | | | | | | | | Miss | | | | | | | | | | | Ions | | | | | | | | | | | Peptide | | | | | | | | | | | | | | | |  | | 770.2930 | | 769.2857 | | | | | | | | | | 769.3866 | | | | | | | | | | | -131.19 | | | | | | | | | | | 252 | | | | | | | | | | | - | | | | | | | | | | | 257 | | | | | | | | | | | 0 | | | | | | | | | | | --- | | | | | | | | | | | K.YMLLCK.I | | | | | | | | | | | | | | | |  | | 772.2836 | | 771.2763 | | | | | | | | | | 771.4391 | | | | | | | | | | | -211.10 | | | | | | | | | | | 176 | | | | | | | | | | | - | | | | | | | | | | | 181 | | | | | | | | | | | 0 | | | | | | | | | | | --- | | | | | | | | | | | K.LHFSLR.N | | | | | | | | | | | | | | | |  | | 849.3220 | | 848.3147 | | | | | | | | | | 848.4756 | | | | | | | | | | | -189.57 | | | | | | | | | | | 300 | | | | | | | | | | | - | | | | | | | | | | | 306 | | | | | | | | | | | 0 | | | | | | | | | | | --- | | | | | | | | | | | K.LFETALR.D | | | | | | | | | | | | | | | |  | | 877.3125 | | 876.3052 | | | | | | | | | | 876.4705 | | | | | | | | | | | -188.56 | | | | | | | | | | | 337 | | | | | | | | | | | - | | | | | | | | | | | 343 | | | | | | | | | | | 0 | | | | | | | | | | | --- | | | | | | | | | | | R.LIEPYSR.V | | | | | | | | | | | | | | | |  | | 877.3125 | | 876.3052 | | | | | | | | | | 876.4705 | | | | | | | | | | | -188.56 | | | | | | | | | | | 337 | | | | | | | | | | | - | | | | | | | | | | | 343 | | | | | | | | | | | 0 | | | | | | | | | | | 19 | | | | | | | | | | | R.LIEPYSR.V | | | | | | | | | | | | | | | |  | | 934.3560 | | 933.3487 | | | | | | | | | | 933.5284 | | | | | | | | | | | -192.49 | | | | | | | | | | | 409 | | | | | | | | | | | - | | | | | | | | | | | 416 | | | | | | | | | | | 0 | | | | | | | | | | | --- | | | | | | | | | | | K.VVDSLFVR.S | | | | | | | | | | | | | | | |  | | 934.3560 | | 933.3487 | | | | | | | | | | 933.5284 | | | | | | | | | | | -192.49 | | | | | | | | | | | 409 | | | | | | | | | | | - | | | | | | | | | | | 416 | | | | | | | | | | | 0 | | | | | | | | | | | 53 | | | | | | | | | | | K.VVDSLFVR.S | | | | | | | | | | | | | | | |  | | 1019.3694 | | 1018.3621 | | | | | | | | | | 1018.5481 | | | | | | | | | | | -182.57 | | | | | | | | | | | 133 | | | | | | | | | | | - | | | | | | | | | | | 141 | | | | | | | | | | | 0 | | | | | | | | | | | --- | | | | | | | | | | | R.LAALLMESR.E + Oxidation (M) | | | | | | | | | | | | | | | |  | | 1177.4049 | | 1176.3976 | | | | | | | | | | 1176.6866 | | | | | | | | | | | -245.61 | | | | | | | | | | | 297 | | | | | | | | | | | - | | | | | | | | | | | 306 | | | | | | | | | | | 1 | | | | | | | | | | | --- | | | | | | | | | | | R.SLKLFETALR.D | | | | | | | | | | | | | | | |  | | 1239.3949 | | 1238.3876 | | | | | | | | | | 1238.6659 | | | | | | | | | | | -224.67 | | | | | | | | | | | 300 | | | | | | | | | | | - | | | | | | | | | | | 309 | | | | | | | | | | | 1 | | | | | | | | | | | --- | | | | | | | | | | | K.LFETALRDFK.A | | | | | | | | | | | | | | | |  | | 1306.4525 | | 1305.4452 | | | | | | | | | | 1305.6677 | | | | | | | | | | | -170.39 | | | | | | | | | | | 310 | | | | | | | | | | | - | | | | | | | | | | | 320 | | | | | | | | | | | 0 | | | | | | | | | | | --- | | | | | | | | | | | K.AQLEEDPIVHR.H | | | | | | | | | | | | | | | |  | | 1306.4525 | | 1305.4452 | | | | | | | | | | 1305.6677 | | | | | | | | | | | -170.39 | | | | | | | | | | | 310 | | | | | | | | | | | - | | | | | | | | | | | 320 | | | | | | | | | | | 0 | | | | | | | | | | | 72 | | | | | | | | | | | K.AQLEEDPIVHR.H | | | | | | | | | | | | | | | |  | | 1372.5311 | | 1371.5238 | | | | | | | | | | 1371.7432 | | | | | | | | | | | -159.90 | | | | | | | | | | | 99 | | | | | | | | | | | - | | | | | | | | | | | 111 | | | | | | | | | | | 0 | | | | | | | | | | | --- | | | | | | | | | | | K.IPGTSELQIALCK.E | | | | | | | | | | | | | | | |  | | 1372.5311 | | 1371.5238 | | | | | | | | | | 1371.7609 | | | | | | | | | | | -172.83 | | | | | | | | | | | 47 | | | | | | | | | | | - | | | | | | | | | | | 58 | | | | | | | | | | | 0 | | | | | | | | | | | 97 | | | | | | | | | | | K.ELAITELSDLLR.Q | | | | | | | | | | | | | | | |  | | 1375.4534 | | 1374.4461 | | | | | | | | | | 1374.6627 | | | | | | | | | | | -157.53 | | | | | | | | | | | 32 | | | | | | | | | | | - | | | | | | | | | | | 44 | | | | | | | | | | | 0 | | | | | | | | | | | --- | | | | | | | | | | | R.VLDDPSSSSEALR.V | | | | | | | | | | | | | | | |  | | 1507.4678 | | 1506.4605 | | | | | | | | | | 1506.7388 | | | | | | | | | | | -184.70 | | | | | | | | | | | 274 | | | | | | | | | | | - | | | | | | | | | | | 287 | | | | | | | | | | | 0 | | | | | | | | | | | --- | | | | | | | | | | | K.AGLQYVGPELDAMK.A + Oxidation (M) | | | | | | | | | | | | | | | |  | | 1553.4755 | | 1552.4682 | | | | | | | | | | 1552.7191 | | | | | | | | | | | -161.58 | | | | | | | | | | | 18 | | | | | | | | | | | - | | | | | | | | | | | 31 | | | | | | | | | | | 0 | | | | | | | | | | | --- | | | | | | | | | | | K.EASNPSDAISMLYR.V | | | | | | | | | | | | | | | |  | | 1569.4447 | | 1568.4374 | | | | | | | | | | 1568.7140 | | | | | | | | | | | -176.33 | | | | | | | | | | | 18 | | | | | | | | | | | - | | | | | | | | | | | 31 | | | | | | | | | | | 0 | | | | | | | | | | | --- | | | | | | | | | | | K.EASNPSDAISMLYR.V + Oxidation (M) | | | | | | | | | | | | | | | |  | | 1569.4447 | | 1568.4374 | | | | | | | | | | 1568.7140 | | | | | | | | | | | -176.33 | | | | | | | | | | | 18 | | | | | | | | | | | - | | | | | | | | | | | 31 | | | | | | | | | | | 0 | | | | | | | | | | | 49 | | | | | | | | | | | K.EASNPSDAISMLYR.V + Oxidation (M) | | | | | | | | | | | | | | | |  | | 1580.5586 | | 1579.5513 | | | | | | | | | | 1579.8709 | | | | | | | | | | | -202.26 | | | | | | | | | | | 142 | | | | | | | | | | | - | | | | | | | | | | | 155 | | | | | | | | | | | 0 | | | | | | | | | | | --- | | | | | | | | | | | R.EYTEALTLLTSLVK.E | | | | | | | | | | | | | | | |  | | 1649.5403 | | 1648.5330 | | | | | | | | | | 1648.8342 | | | | | | | | | | | -182.65 | | | | | | | | | | | 258 | | | | | | | | | | | - | | | | | | | | | | | 273 | | | | | | | | | | | 0 | | | | | | | | | | | --- | | | | | | | | | | | K.IMVSQADDVAGIISSK.A + Oxidation (M) | | | | | | | | | | | | | | | |  | | 1810.5502 | | 1809.5429 | | | | | | | | | | 1809.8818 | | | | | | | | | | | -187.26 | | | | | | | | | | | 392 | | | | | | | | | | | - | | | | | | | | | | | 408 | | | | | | | | | | | 0 | | | | | | | | | | | --- | | | | | | | | | | | K.ADAIYPATLETISNMGK.V + Oxidation (M) | | | | | | | | | | | | | | | |  | | 2167.8057 | | 2166.7984 | | | | | | | | | | 2167.1888 | | | | | | | | | | | -180.14 | | | | | | | | | | | 344 | | | | | | | | | | | - | | | | | | | | | | | 362 | | | | | | | | | | | 0 | | | | | | | | | | | --- | | | | | | | | | | | R.VEIAHIAELIELPIDHVEK.K | | | | | | | | | | | | | | | |  | | 2934.9932 | | 2933.9859 | | | | | | | | | | 2934.5087 | | | | | | | | | | | -178.14 | | | | | | | | | | | 195 | | | | | | | | | | | - | | | | | | | | | | | 222 | | | | | | | | | | | 0 | | | | | | | | | | | --- | | | | | | | | | | | R.TAANAIYVPPAQQGTIDLQSGILHAEEK.D | | | | | | | | | | | | | | | |  |  | No match to: 713.2748, 716.2394, 724.3471, 730.2477, 734.3373, 756.2355, 768.3678, 771.2694, 782.2689, 806.2557, 822.2650, 824.3030, 834.3315, 842.3498, 847.2815, 848.3101, 868.3701, 882.3799, 898.3582, 906.2909, 918.2842, 948.3191, 948.3191, 961.2850, 962.3041, 962.3041, 969.2799, 979.3016, 981.2820, 994.3414, 995.3447, 997.3102, 999.3166, 1034.3241, 1045.3833, 1049.3502, 1059.3816, 1074.3475, 1080.4270, 1102.3750, 1127.3781, 1138.3651, 1141.4176, 1150.3777, 1151.4419, 1158.3939, 1161.3690, 1165.4354, 1173.3896, 1194.3547, 1223.4063, 1252.4615, 1258.4233, 1265.4294, 1267.3840, 1273.3602, 1280.4683, 1289.4076, 1324.4744, 1354.5172, 1368.4049, 1405.4501, 1410.4055, 1416.5397, 1424.5182, 1425.5641, 1428.4742, 1453.5087, 1453.5087, 1501.5483, 1523.4667, 1535.5204, 1585.4526, 1618.5793, 1681.5133, 1693.5728, 1693.5728, 1704.4955, 1870.6615, 1871.6658, 2206.6819, 2206.6819, 2238.7354, 2245.8123, 2301.7439, 2808.9839 | | | | | | | | | | | | | | | | | | | | | | | | | | | | | | | | | | | | | | | | | | | | | | | | | | | | | | | | | | | | | | | | | | | | | | | | | | | | |  | | | | | | | | | --- | --- | --- | --- | --- | --- | --- | --- | --- | --- | --- | --- | --- | --- | --- | --- | --- | --- | --- | --- | --- | --- | --- | --- | --- | --- | --- | --- | --- | --- | --- | --- | --- | --- | --- | --- | --- | --- | --- | --- | --- | --- | --- | --- | --- | --- | --- | --- | --- | --- | --- | --- | --- | --- | --- | --- | --- | --- | --- | --- | --- | --- | --- | --- | --- | --- | --- | --- | --- | --- | --- | --- | --- | --- | --- | --- | --- | --- | --- | --- | --- | --- | --- | --- | --- | | **S50** [**orange1.1t04488.1**](http://zhangyang-pc/mascot/cgi/protein_view.pl?file=../data/20140118/F012128.dat&hit=1) **Mass: 30675 Score: 333 Expect: 2.2e-029 Matches: 15** | | | | | | | | | | | | | | | | | | | | | | | | | | | | | | | | | | | | | | | | | | | | | | | | | | | | | | | | | | | | | | | | | | | | | | | | | | | | |  | | | | | | | | | Observed | | | | | | Mr(expt) | | | | | | | | Mr(calc) | | | | | | | | ppm | | | | | | | | Start | | | | | | | | |  | | | | | | | End | | | | | | | | Miss | | | | | | | | Ions | | | | | | | | Peptide | | | | | | | | | | | | | | | | 780.2541 | | | | | | 779.2469 | | | | | | | | 779.3966 | | | | | | | | -192.13 | | | | | | | | 250 | | | | | | | | | - | | | | | | | 255 | | | | | | | | 0 | | | | | | | | --- | | | | | | | | K.FHVSYK.G | | | | | | | | | | | | | | | | 1037.3213 | | | | | | 1036.3140 | | | | | | | | 1036.4825 | | | | | | | | -162.57 | | | | | | | | 277 | | | | | | | | | - | | | | | | | 285 | | | | | | | | 0 | | | | | | | | --- | | | | | | | | R.RPTTEEASF.- | | | | | | | | | | | | | | | | 1037.3213 | | | | | | 1036.3140 | | | | | | | | 1036.4825 | | | | | | | | -162.57 | | | | | | | | 277 | | | | | | | | | - | | | | | | | 285 | | | | | | | | 0 | | | | | | | | 45 | | | | | | | | R.RPTTEEASF.- | | | | | | | | | | | | | | | | 1205.4532 | | | | | | 1204.4459 | | | | | | | | 1204.5845 | | | | | | | | -115.04 | | | | | | | | 35 | | | | | | | | | - | | | | | | | 45 | | | | | | | | 0 | | | | | | | | --- | | | | | | | | R.FGNKPLCCPAR.R | | | | | | | | | | | | | | | | 1205.4532 | | | | | | 1204.4459 | | | | | | | | 1204.5845 | | | | | | | | -115.04 | | | | | | | | 35 | | | | | | | | | - | | | | | | | 45 | | | | | | | | 0 | | | | | | | | --- | | | | | | | | R.FGNKPLCCPAR.R | | | | | | | | | | | | | | | | 1275.4736 | | | | | | 1274.4663 | | | | | | | | 1274.6904 | | | | | | | | -175.79 | | | | | | | | 102 | | | | | | | | | - | | | | | | | 113 | | | | | | | | 0 | | | | | | | | --- | | | | | | | | R.LGALLDTMNALK.N + Oxidation (M) | | | | | | | | | | | | | | | | 1383.5233 | | | | | | 1382.5160 | | | | | | | | 1382.7405 | | | | | | | | -162.36 | | | | | | | | 145 | | | | | | | | | - | | | | | | | 156 | | | | | | | | 1 | | | | | | | | --- | | | | | | | | R.KVDDPEVLEAIR.L | | | | | | | | | | | | | | | | 1383.5233 | | | | | | 1382.5160 | | | | | | | | 1382.7405 | | | | | | | | -162.36 | | | | | | | | 145 | | | | | | | | | - | | | | | | | 156 | | | | | | | | 1 | | | | | | | | 75 | | | | | | | | R.KVDDPEVLEAIR.L | | | | | | | | | | | | | | | | 1663.7533 | | | | | | 1662.7460 | | | | | | | | 1663.0144 | | | | | | | | -161.40 | | | | | | | | 258 | | | | | | | | | - | | | | | | | 272 | | | | | | | | 0 | | | | | | | | --- | | | | | | | | K.AIIKPLQQVLANSLR.Y | | | | | | | | | | | | | | | | 1663.7533 | | | | | | 1662.7460 | | | | | | | | 1663.0144 | | | | | | | | -161.40 | | | | | | | | 258 | | | | | | | | | - | | | | | | | 272 | | | | | | | | 0 | | | | | | | | 105 | | | | | | | | K.AIIKPLQQVLANSLR.Y | | | | | | | | | | | | | | | | 2050.8757 | | | | | | 2049.8684 | | | | | | | | 2050.2038 | | | | | | | | -163.57 | | | | | | | | 207 | | | | | | | | | - | | | | | | | 225 | | | | | | | | 0 | | | | | | | | --- | | | | | | | | R.SLLLVETADRPGLLVDLVK.I | | | | | | | | | | | | | | | | 2050.8757 | | | | | | 2049.8684 | | | | | | | | 2050.2038 | | | | | | | | -163.57 | | | | | | | | 207 | | | | | | | | | - | | | | | | | 225 | | | | | | | | 0 | | | | | | | | 71 | | | | | | | | R.SLLLVETADRPGLLVDLVK.I | | | | | | | | | | | | | | | | 2133.6221 | | | | | | 2132.6148 | | | | | | | | 2132.9710 | | | | | | | | -166.97 | | | | | | | | 59 | | | | | | | | | - | | | | | | | 80 | | | | | | | | 0 | | | | | | | | --- | | | | | | | | R.ASSATAVEDGSNGDTDTIPTPK.V | | | | | | | | | | | | | | | | 2226.6946 | | | | | | 2225.6873 | | | | | | | | 2226.0553 | | | | | | | | -165.32 | | | | | | | | 187 | | | | | | | | | - | | | | | | | 206 | | | | | | | | 0 | | | | | | | | --- | | | | | | | | K.QQVDVDIATHISVFDDGPDR.S | | | | | | | | | | | | | | | | 2292.7507 | | | | | | 2291.7434 | | | | | | | | 2291.0861 | | | | | | | | 287 | | | | | | | | 2 | | | | | | | | | - | | | | | | | 23 | | | | | | | | 0 | | | | | | | | --- | | | | | | | | M.AVAMGSASVHLAVQICCNNEFK.A | | | | | | | | | | | | | | | | No match to: 700.2306, 705.2383, 708.2240, 711.2346, 713.2837, 716.2358, 730.2214, 732.2452, 733.2076, 734.2120, 737.2893, 746.2136, 747.2514, 748.2085, 752.2419, 768.3791, 770.2643, 792.2416, 816.2711, 838.2676, 842.3568, 850.3476, 853.4280, 860.9295, 868.3695, 872.2979, 882.3926, 911.4689, 913.3741, 917.3673, 922.2680, 939.3320, 948.2693, 956.2855, 969.4976, 985.4634, 988.3084, 1027.5333, 1043.5095, 1045.4092, 1059.3826, 1081.3850, 1085.5764, 1108.3481, 1116.3866, 1126.3601, 1127.3662, 1141.4750, 1143.5936, 1150.4159, 1159.4846, 1190.4478, 1202.4333, 1221.4512, 1221.4512, 1227.4266, 1236.4395, 1239.4200, 1254.4429, 1254.4429, 1301.4644, 1315.4329, 1366.3304, 1404.3403, 1503.6090, 1517.6364, 1750.5570, 1750.5570, 1787.6447, 1787.6447, 1801.6399, 1809.6212, 2107.6953, 2111.6262, 2121.7705, 2124.7014, 2124.7014, 2129.6443, 2138.7166, 2142.8220, 2146.6917, 2150.6038, 2162.6138, 2183.6309, 2184.6060, 2198.5732, 2201.6008, 2206.6045, 2209.6658, 2214.5742, 2220.7324, 2237.6448, 2238.7129, 2245.8101, 2330.7676 | | | | | | | | | | | | | | | | | | | | | | | | | | | | | | | | | | | | | | | | | | | | | | | | | | | | | | | | | | | | | | | | | | | | | | | | | | | | |  | | | | | | | | | **S51** [**Cs8g05140.1**](http://zhangyang-pc/mascot/cgi/protein_view.pl?file=../data/20140118/F012167.dat&hit=1) **Mass: 30596 Score: 270 Expect: 4.4e-023 Matches: 13** | | | | | | | | | | | | | | | | | | | | | | | | | | | | | | | | | | | | | | | | | | | | | | | | | | | | | | | | | | | | | | | | | | | | | | | | | | | | |  | | | | | | | | | Observed | | | Mr(expt) | | | | | | | Mr(calc) | | | | | | | | ppm | | | | | | | | Start | | | | | | | |  | | | | | | | | End | | | | | | | | Miss | | | | | | | | Ions | | | | | | | | Peptide | | | | | | | | | | | | | | | | | | | | 719.2143 | | | 718.2070 | | | | | | | 718.3610 | | | | | | | | -214.30 | | | | | | | | 175 | | | | | | | | - | | | | | | | | 180 | | | | | | | | 0 | | | | | | | | --- | | | | | | | | R.TLNTDR.Q | | | | | | | | | | | | | | | | | | | | 737.2110 | | | 736.2037 | | | | | | | 736.3504 | | | | | | | | -199.20 | | | | | | | | 55 | | | | | | | | - | | | | | | | | 61 | | | | | | | | 0 | | | | | | | | --- | | | | | | | | R.EVYGGGR.I | | | | | | | | | | | | | | | | | | | | 893.2788 | | | 892.2715 | | | | | | | 892.4515 | | | | | | | | -201.66 | | | | | | | | 54 | | | | | | | | - | | | | | | | | 61 | | | | | | | | 1 | | | | | | | | --- | | | | | | | | R.REVYGGGR.I | | | | | | | | | | | | | | | | | | | | 997.4052 | | | 996.3979 | | | | | | | 996.5855 | | | | | | | | -188.29 | | | | | | | | 236 | | | | | | | | - | | | | | | | | 244 | | | | | | | | 0 | | | | | | | | --- | | | | | | | | R.EIIPVEGLK.L | | | | | | | | | | | | | | | | | | | | 1018.3051 | | | 1017.2978 | | | | | | | 1017.4913 | | | | | | | | -190.18 | | | | | | | | 262 | | | | | | | | - | | | | | | | | 271 | | | | | | | | 0 | | | | | | | | --- | | | | | | | | R.MVGAEGSPVR.C + Oxidation (M) | | | | | | | | | | | | | | | | | | | | 1018.3051 | | | 1017.2978 | | | | | | | 1017.4913 | | | | | | | | -190.18 | | | | | | | | 262 | | | | | | | | - | | | | | | | | 271 | | | | | | | | 0 | | | | | | | | 25 | | | | | | | | R.MVGAEGSPVR.C + Oxidation (M) | | | | | | | | | | | | | | | | | | | | 1524.3832 | | | 1523.3759 | | | | | | | 1523.6603 | | | | | | | | -186.62 | | | | | | | | 187 | | | | | | | | - | | | | | | | | 200 | | | | | | | | 0 | | | | | | | | --- | | | | | | | | K.FDTSYVGFMADGAK.W + Oxidation (M) | | | | | | | | | | | | | | | | | | | | 1524.3832 | | | 1523.3759 | | | | | | | 1523.6603 | | | | | | | | -186.62 | | | | | | | | 187 | | | | | | | | - | | | | | | | | 200 | | | | | | | | 0 | | | | | | | | 68 | | | | | | | | K.FDTSYVGFMADGAK.W + Oxidation (M) | | | | | | | | | | | | | | | | | | | | 1652.4493 | | | 1651.4420 | | | | | | | 1651.7552 | | | | | | | | -189.61 | | | | | | | | 186 | | | | | | | | - | | | | | | | | 200 | | | | | | | | 1 | | | | | | | | --- | | | | | | | | K.KFDTSYVGFMADGAK.W + Oxidation (M) | | | | | | | | | | | | | | | | | | | | 1652.4493 | | | 1651.4420 | | | | | | | 1651.7552 | | | | | | | | -189.61 | | | | | | | | 186 | | | | | | | | - | | | | | | | | 200 | | | | | | | | 1 | | | | | | | | 67 | | | | | | | | K.KFDTSYVGFMADGAK.W + Oxidation (M) | | | | | | | | | | | | | | | | | | | | 2652.9048 | | | 2651.8975 | | | | | | | 2652.3911 | | | | | | | | -186.10 | | | | | | | | 210 | | | | | | | | - | | | | | | | | 233 | | | | | | | | 0 | | | | | | | | --- | | | | | | | | K.LVGVDYLSVAAFDDIISAHHELLR.N | | | | | | | | | | | | | | | | | | | | 2652.9048 | | | 2651.8975 | | | | | | | 2652.3911 | | | | | | | | -186.10 | | | | | | | | 210 | | | | | | | | - | | | | | | | | 233 | | | | | | | | 0 | | | | | | | | 75 | | | | | | | | K.LVGVDYLSVAAFDDIISAHHELLR.N | | | | | | | | | | | | | | | | | | | | 3033.9746 | | | 3032.9673 | | | | | | | 3033.5448 | | | | | | | | -190.36 | | | | | | | | 62 | | | | | | | | - | | | | | | | | 88 | | | | | | | | 0 | | | | | | | | --- | | | | | | | | R.IFDITHQVTVDLPSYDTEGGLLGQFLR.L | | | | | | | | | | | | | | | | | | | | No match to: 700.2368, 703.2083, 706.2429, 712.1754, 713.2667, 716.2232, 717.2156, 721.2203, 726.2175, 730.2159, 733.2739, 734.3048, 736.2651, 743.2907, 768.3708, 771.2175, 812.2298, 842.3444, 848.3414, 850.3447, 856.2925, 864.3232, 868.3723, 871.3457, 876.8767, 882.3870, 905.3218, 905.3218, 952.2935, 954.3113, 982.3643, 996.3505, 1003.2715, 1034.3104, 1051.5026, 1055.3698, 1059.3644, 1083.3219, 1094.3496, 1107.2673, 1115.4022, 1121.3785, 1126.3478, 1127.3929, 1133.3759, 1149.3595, 1151.4396, 1165.4597, 1206.4584, 1268.3989, 1280.3789, 1292.4336, 1296.3925, 1300.5188, 1321.4489, 1321.4489, 1343.4242, 1349.3746, 1357.4492, 1404.4290, 1411.5381, 1422.4779, 1427.4448, 1465.4703, 1465.4703, 1507.4675, 1529.4224, 1540.3956, 1543.5310, 1563.3950, 1586.5140, 1641.4594, 1668.4572, 1674.4440, 1690.4314, 1693.5592, 1714.4816, 1735.5350, 1825.5669, 1868.7074, 1868.7074, 1871.6934, 1883.6234, 1890.6638, 1901.6600, 1918.6387, 1919.6538, 1976.6299, 1976.6299, 1992.6598, 2220.7222, 2238.7148, 2245.7805, 2362.7183, 2786.8525, 2786.8525, 2875.8975 | | | | | | | | | | | | | | | | | | | | | | | | | | | | | | | | | | | | | | | | | | | | | | | | | | | | | | | | | | | | | | | | | | | | | | | | | | | | |  | | | | | | | | | **S45** [**Cs9g03120.1**](http://zhangyang-pc/mascot/cgi/protein_view.pl?file=../data/20140118/F012087.dat&hit=1) **Mass: 49720 Score: 487 Expect: 8.8e-045 Matches: 20** | | | | | | | | | | | | | | | | | | | | | | | | | | | | | | | | | | | | | | | | | | | | | | | | | | | | | | | | | | | | | | | | | | | | | | | | | | | | |  | | | | | | | | | Observed | | | | Mr(expt) | | | | | | | Mr(calc) | | | | | | | | ppm | | | | | | | | Start | | | | | | | |  | | | | | | | | End | | | | | | | | Miss | | | | | | | | Ions | | | | | | | | Peptide | | | | | | | | |  | | | | | | | | | | 781.2609 | | | | 780.2537 | | | | | | | 780.4018 | | | | | | | | -189.79 | | | | | | | | 157 | | | | | | | | - | | | | | | | | 163 | | | | | | | | 0 | | | | | | | | --- | | | | | | | | R.LSVDYGK.K | | | | | | | | |  | | | | | | | | | | 903.2682 | | | | 902.2610 | | | | | | | 902.4208 | | | | | | | | -177.08 | | | | | | | | 395 | | | | | | | | - | | | | | | | | 401 | | | | | | | | 0 | | | | | | | | --- | | | | | | | | K.FDLMYAK.R + Oxidation (M) | | | | | | | | |  | | | | | | | | | | 1001.3829 | | | | 1000.3756 | | | | | | | 1000.5553 | | | | | | | | -179.57 | | | | | | | | 327 | | | | | | | | - | | | | | | | | 336 | | | | | | | | 0 | | | | | | | | --- | | | | | | | | K.DVNAAVATIK.T | | | | | | | | |  | | | | | | | | | | 1007.2834 | | | | 1006.2761 | | | | | | | 1006.4468 | | | | | | | | -169.58 | | | | | | | | 97 | | | | | | | | - | | | | | | | | 105 | | | | | | | | 0 | | | | | | | | --- | | | | | | | | K.EDAANNFAR.G | | | | | | | | |  | | | | | | | | | | 1007.2834 | | | | 1006.2761 | | | | | | | 1006.4468 | | | | | | | | -169.58 | | | | | | | | 97 | | | | | | | | - | | | | | | | | 105 | | | | | | | | 0 | | | | | | | | 62 | | | | | | | | K.EDAANNFAR.G | | | | | | | | |  | | | | | | | | | | 1059.3777 | | | | 1058.3704 | | | | | | | 1058.5219 | | | | | | | | -143.08 | | | | | | | | 395 | | | | | | | | - | | | | | | | | 402 | | | | | | | | 1 | | | | | | | | --- | | | | | | | | K.FDLMYAKR.A + Oxidation (M) | | | | | | | | |  | | | | | | | | | | 1396.5104 | | | | 1395.5031 | | | | | | | 1395.6857 | | | | | | | | -130.78 | | | | | | | | 391 | | | | | | | | - | | | | | | | | 401 | | | | | | | | 1 | | | | | | | | --- | | | | | | | | R.IDHKFDLMYAK.R + Oxidation (M) | | | | | | | | |  | | | | | | | | | | 1396.5104 | | | | 1395.5031 | | | | | | | 1395.7510 | | | | | | | | -177.62 | | | | | | | | 85 | | | | | | | | - | | | | | | | | 96 | | | | | | | | 0 | | | | | | | | 81 | | | | | | | | R.QLFHPEQLISGK.E | | | | | | | | |  | | | | | | | | | | 1531.6523 | | | | 1530.6450 | | | | | | | 1530.8981 | | | | | | | | -165.30 | | | | | | | | 230 | | | | | | | | - | | | | | | | | 243 | | | | | | | | 0 | | | | | | | | --- | | | | | | | | R.LISQIISSLTTSLR.F | | | | | | | | |  | | | | | | | | | | 1531.6523 | | | | 1530.6450 | | | | | | | 1530.8981 | | | | | | | | -165.30 | | | | | | | | 230 | | | | | | | | - | | | | | | | | 243 | | | | | | | | 0 | | | | | | | | 78 | | | | | | | | R.LISQIISSLTTSLR.F | | | | | | | | |  | | | | | | | | | | 1691.5826 | | | | 1690.5753 | | | | | | | 1690.8638 | | | | | | | | -170.62 | | | | | | | | 216 | | | | | | | | - | | | | | | | | 229 | | | | | | | | 0 | | | | | | | | --- | | | | | | | | R.SLDIERPTYTNLNR.L | | | | | | | | |  | | | | | | | | | | 1691.5826 | | | | 1690.5753 | | | | | | | 1690.8638 | | | | | | | | -170.62 | | | | | | | | 216 | | | | | | | | - | | | | | | | | 229 | | | | | | | | 0 | | | | | | | | 46 | | | | | | | | R.SLDIERPTYTNLNR.L | | | | | | | | |  | | | | | | | | | | 1701.6229 | | | | 1700.6156 | | | | | | | 1700.8985 | | | | | | | | -166.32 | | | | | | | | 65 | | | | | | | | - | | | | | | | | 79 | | | | | | | | 0 | | | | | | | | --- | | | | | | | | R.AVFVDLEPTVIDEVR.T | | | | | | | | |  | | | | | | | | | | 1701.6229 | | | | 1700.6156 | | | | | | | 1700.8985 | | | | | | | | -166.32 | | | | | | | | 65 | | | | | | | | - | | | | | | | | 79 | | | | | | | | 0 | | | | | | | | 85 | | | | | | | | R.AVFVDLEPTVIDEVR.T | | | | | | | | |  | | | | | | | | | | 1792.6166 | | | | 1791.6093 | | | | | | | 1791.9229 | | | | | | | | -175.00 | | | | | | | | 265 | | | | | | | | - | | | | | | | | 280 | | | | | | | | 0 | | | | | | | | --- | | | | | | | | R.IHFMLSSYAPVISAEK.A | | | | | | | | |  | | | | | | | | | | 1808.5936 | | | | 1807.5863 | | | | | | | 1807.9178 | | | | | | | | -183.36 | | | | | | | | 265 | | | | | | | | - | | | | | | | | 280 | | | | | | | | 0 | | | | | | | | --- | | | | | | | | R.IHFMLSSYAPVISAEK.A + Oxidation (M) | | | | | | | | |  | | | | | | | | | | 1808.5936 | | | | 1807.5863 | | | | | | | 1807.9178 | | | | | | | | -183.36 | | | | | | | | 265 | | | | | | | | - | | | | | | | | 280 | | | | | | | | 0 | | | | | | | | 37 | | | | | | | | R.IHFMLSSYAPVISAEK.A + Oxidation (M) | | | | | | | | |  | | | | | | | | | | 2408.8079 | | | | 2407.8006 | | | | | | | 2408.2012 | | | | | | | | -166.35 | | | | | | | | 244 | | | | | | | | - | | | | | | | | 264 | | | | | | | | 0 | | | | | | | | --- | | | | | | | | R.FDGAINVDITEFQTNLVPYPR.I | | | | | | | | |  | | | | | | | | | | 2408.8079 | | | | 2407.8006 | | | | | | | 2408.2012 | | | | | | | | -166.35 | | | | | | | | 244 | | | | | | | | - | | | | | | | | 264 | | | | | | | | 0 | | | | | | | | 57 | | | | | | | | R.FDGAINVDITEFQTNLVPYPR.I | | | | | | | | |  | | | | | | | | | | 2710.7788 | | | | 2709.7715 | | | | | | | 2710.2618 | | | | | | | | -180.91 | | | | | | | | 281 | | | | | | | | - | | | | | | | | 304 | | | | | | | | 0 | | | | | | | | --- | | | | | | | | K.AYHEQLSVPEITNAVFEPSSMMAK.C + 2 Oxidation (M) | | | | | | | | |  | | | | | | | | | | No match to: 705.2492, 713.2869, 728.2409, 730.2292, 743.2681, 744.2997, 758.1960, 768.3754, 839.2729, 842.3592, 846.2873, 850.3844, 854.2599, 868.3829, 876.8942, 882.4005, 978.3242, 989.2844, 1006.2612, 1041.3833, 1045.4084, 1051.5311, 1053.3224, 1064.3838, 1102.3335, 1117.2979, 1132.4006, 1148.3651, 1150.4211, 1216.3699, 1218.4211, 1224.3091, 1231.3656, 1328.4492, 1378.4984, 1379.4961, 1379.4961, 1395.4884, 1401.4965, 1416.5055, 1417.4861, 1418.5072, 1431.4589, 1433.5150, 1434.5106, 1486.5367, 1542.4214, 1549.4441, 1549.4441, 1604.4395, 1616.4482, 1632.4694, 1647.5454, 1657.6123, 1657.6123, 1663.5297, 1673.5688, 1674.5935, 1687.6139, 1705.4536, 1706.5863, 1713.5634, 1715.5992, 1721.4364, 1723.5461, 1727.5203, 1729.5500, 1794.5868, 1807.5586, 1820.6156, 1824.5958, 1830.5765, 1842.5598, 1882.6328, 1884.5789, 1900.5326, 1906.6024, 1912.5862, 2002.7242, 2127.6707, 2156.4136, 2238.7312, 2245.7732, 2297.6614, 2349.6106, 2356.6890, 2361.5999, 2377.6082, 2393.5911, 2646.7444 | | | | | | | | | | | | | | | | | | | | | | | | | | | | | | | | | | | | | | | | | | | | | | | | | | | | | | | | | | | | | | | | | | | | | | | | | | | | |  | | | | | | | | | **S46** [**orange1.1t05216.1**](http://zhangyang-pc/mascot/cgi/protein_view.pl?file=../data/20140118/F012179.dat&hit=1) **Mass: 29957 Score: 194 Expect: 1.8e-015 Matches: 11** | | | | | | | | | | | | | | | | | | | | | | | | | | | | | | | | | | | | | | | | | | | | | | | | | | | | | | | | | | | | | | | | | | | | | | | | | | | | |  | | | | | | | | | Observed | | | | | | Mr(expt) | | | | | | | | Mr(calc) | | | | | | | | ppm | | | | | | | | Start | | | | | | | | |  | | | | | | | End | | | | | | | | Miss | | | | | | | | Ions | | | | | | | | Peptide | | | | | | | | | | | | | | | | 1356.4442 | | | | | | 1355.4369 | | | | | | | | 1355.7700 | | | | | | | | -245.70 | | | | | | | | 105 | | | | | | | | | - | | | | | | | 116 | | | | | | | | 0 | | | | | | | | --- | | | | | | | | K.AILDPTIEPFLK.V | | | | | | | | | | | | | | | | 1370.3956 | | | | | | 1369.3883 | | | | | | | | 1369.6302 | | | | | | | | -176.64 | | | | | | | | 117 | | | | | | | | | - | | | | | | | 128 | | | | | | | | 0 | | | | | | | | --- | | | | | | | | K.VHGEPAYSYYGK.I | | | | | | | | | | | | | | | | 1408.4756 | | | | | | 1407.4683 | | | | | | | | 1407.7147 | | | | | | | | -174.99 | | | | | | | | 46 | | | | | | | | | - | | | | | | | 57 | | | | | | | | 0 | | | | | | | | --- | | | | | | | | R.LLTSYGVFSEHR.E | | | | | | | | | | | | | | | | 1408.4756 | | | | | | 1407.4683 | | | | | | | | 1407.7147 | | | | | | | | -174.99 | | | | | | | | 46 | | | | | | | | | - | | | | | | | 57 | | | | | | | | 0 | | | | | | | | 66 | | | | | | | | R.LLTSYGVFSEHR.E | | | | | | | | | | | | | | | | 1426.4718 | | | | | | 1425.4645 | | | | | | | | 1425.7212 | | | | | | | | -180.03 | | | | | | | | 29 | | | | | | | | | - | | | | | | | 42 | | | | | | | | 0 | | | | | | | | --- | | | | | | | | R.ILPSGGGDAENLQR.I | | | | | | | | | | | | | | | | 1426.4718 | | | | | | 1425.4645 | | | | | | | | 1425.7212 | | | | | | | | -180.03 | | | | | | | | 29 | | | | | | | | | - | | | | | | | 42 | | | | | | | | 0 | | | | | | | | 62 | | | | | | | | R.ILPSGGGDAENLQR.I | | | | | | | | | | | | | | | | 1640.5566 | | | | | | 1639.5493 | | | | | | | | 1639.8643 | | | | | | | | -192.10 | | | | | | | | 257 | | | | | | | | | - | | | | | | | 270 | | | | | | | | 0 | | | | | | | | --- | | | | | | | | R.ALLEGDIFVMTIYR.A | | | | | | | | | | | | | | | | 1656.5581 | | | | | | 1655.5508 | | | | | | | | 1655.8593 | | | | | | | | -186.27 | | | | | | | | 257 | | | | | | | | | - | | | | | | | 270 | | | | | | | | 0 | | | | | | | | --- | | | | | | | | R.ALLEGDIFVMTIYR.A + Oxidation (M) | | | | | | | | | | | | | | | | 1656.5581 | | | | | | 1655.5508 | | | | | | | | 1655.8593 | | | | | | | | -186.27 | | | | | | | | 257 | | | | | | | | | - | | | | | | | 270 | | | | | | | | 0 | | | | | | | | 48 | | | | | | | | R.ALLEGDIFVMTIYR.A + Oxidation (M) | | | | | | | | | | | | | | | | 2223.6279 | | | | | | 2222.6206 | | | | | | | | 2223.0228 | | | | | | | | -180.93 | | | | | | | | 139 | | | | | | | | | - | | | | | | | 159 | | | | | | | | 0 | | | | | | | | --- | | | | | | | | K.AMSGVSVPFMTSVLDGYDGFK.G + Oxidation (M) | | | | | | | | | | | | | | | | 2336.6677 | | | | | | 2335.6604 | | | | | | | | 2335.1229 | | | | | | | | 230 | | | | | | | | 138 | | | | | | | | | - | | | | | | | 159 | | | | | | | | 1 | | | | | | | | --- | | | | | | | | R.KAMSGVSVPFMTSVLDGYDGFK.G | | | | | | | | | | | | | | | | No match to: 713.2859, 842.3424, 884.3101, 1019.3659, 1074.3184, 1106.2637, 1107.2863, 1206.4243, 1212.4260, 1227.4233, 1254.3793, 1261.3706, 1267.3666, 1277.4275, 1278.4619, 1278.4619, 1295.4841, 1295.4841, 1309.4438, 1318.4025, 1327.4714, 1336.4495, 1339.4949, 1348.3822, 1354.4572, 1418.4658, 1430.4602, 1439.5415, 1446.4678, 1449.4464, 1457.5463, 1487.4089, 1516.4534, 1524.5090, 1554.4672, 1573.5200, 1575.5430, 1590.5795, 1591.6141, 1592.6007, 1593.5972, 1605.5409, 1608.5446, 1632.5593, 1638.5868, 1672.5629, 1707.5076, 1707.5076, 1723.4628, 1781.5952, 1848.5748, 1849.0929, 1849.6754, 1851.5881, 1864.5588, 1886.6030, 1886.6030, 1901.5552, 1908.7427, 1920.5981, 1920.5981, 1942.7155, 1942.7155, 1958.5656, 1962.5813, 1978.5879, 1985.6174, 2008.5652, 2027.6615, 2055.6541, 2086.5886, 2107.6877, 2147.7014, 2175.6711, 2215.7229, 2220.6648, 2227.6965, 2228.6831, 2238.6995, 2244.7366, 2255.6780, 2257.6592, 2276.7859, 2292.7522, 2308.7432, 2309.7273, 2449.7300, 2544.9065, 2547.8979, 2548.8831, 2559.8774, 2560.8811, 2560.8811, 2575.9019, 2576.8586, 2589.9033, 2638.8589, 2726.7979, 3486.0081 | | | | | | | | | | | | | | | | | | | | | | | | | | | | | | | | | | | | | | | | | | | | | | | | | | | | | | | | | | | | | | | | | | | | | | | | | | | | |  | | | | | | | | | **S48** [**Cs3g21990.1**](http://zhangyang-pc/mascot/cgi/protein_view.pl?file=../data/20140118/F012186.dat&hit=1) **Mass: 20471 Score: 143 Expect: 2.2e-010 Matches: 8** | | | | | | | | | | | | | | | | | | | | | | | | | | | | | | | | | | | | | | | | | | | | | | | | | | | | | | | | | | | | | | | | | | | | | | | | | | | | |  | | | | | | | | | Observed | | | | | | Mr(expt) | | | | | | | | Mr(calc) | | | | | | | | ppm | | | | | | | | Start | | | | | | | | |  | | | | | | | End | | | | | | | | Miss | | | | | | | | Ions | | | | | | | | Peptide | | | | | | | | | | | | | | | | 946.3872 | | | | | | 945.3799 | | | | | | | | 945.4226 | | | | | | | | -45.09 | | | | | | | | 2 | | | | | | | | | - | | | | | | | 9 | | | | | | | | 0 | | | | | | | | --- | | | | | | | | M.SMDSHIEK.V | | | | | | | | | | | | | | | | 1199.4742 | | | | | | 1198.4669 | | | | | | | | 1198.6921 | | | | | | | | -187.87 | | | | | | | | 139 | | | | | | | | | - | | | | | | | 149 | | | | | | | | 1 | | | | | | | | --- | | | | | | | | R.KIQVELVGEGK.F | | | | | | | | | | | | | | | | 1423.5360 | | | | | | 1422.5287 | | | | | | | | 1422.7983 | | | | | | | | -189.49 | | | | | | | | 58 | | | | | | | | | - | | | | | | | 69 | | | | | | | | 0 | | | | | | | | --- | | | | | | | | R.NIHLPISVDFIR.A | | | | | | | | | | | | | | | | 1423.5360 | | | | | | 1422.5287 | | | | | | | | 1422.7983 | | | | | | | | -189.49 | | | | | | | | 58 | | | | | | | | | - | | | | | | | 69 | | | | | | | | 0 | | | | | | | | 94 | | | | | | | | R.NIHLPISVDFIR.A | | | | | | | | | | | | | | | | 1603.5594 | | | | | | 1602.5521 | | | | | | | | 1602.8399 | | | | | | | | -179.56 | | | | | | | | 122 | | | | | | | | | - | | | | | | | 137 | | | | | | | | 0 | | | | | | | | --- | | | | | | | | K.GASSVSVCTLLDKPAR.R | | | | | | | | | | | | | | | | 1603.5594 | | | | | | 1602.5521 | | | | | | | | 1602.8399 | | | | | | | | -179.56 | | | | | | | | 122 | | | | | | | | | - | | | | | | | 137 | | | | | | | | 0 | | | | | | | | 26 | | | | | | | | K.GASSVSVCTLLDKPAR.R | | | | | | | | | | | | | | | | 1759.6732 | | | | | | 1758.6659 | | | | | | | | 1758.9410 | | | | | | | | -156.41 | | | | | | | | 122 | | | | | | | | | - | | | | | | | 138 | | | | | | | | 1 | | | | | | | | --- | | | | | | | | K.GASSVSVCTLLDKPARR.K | | | | | | | | | | | | | | | | 2025.6062 | | | | | | 2024.5989 | | | | | | | | 2024.9902 | | | | | | | | -193.23 | | | | | | | | 70 | | | | | | | | | - | | | | | | | 90 | | | | | | | | 0 | | | | | | | | --- | | | | | | | | R.AESYGSGTLSSGAPVLSLDSK.L | | | | | | | | | | | | | | | | No match to: 705.2475, 713.2679, 716.2295, 724.3281, 734.3407, 736.3576, 750.3738, 768.3706, 777.2686, 834.3368, 842.3428, 868.3651, 882.3834, 953.3377, 953.3377, 1015.3527, 1051.5016, 1057.2863, 1059.3656, 1086.3792, 1117.2970, 1122.4075, 1126.3521, 1151.4720, 1165.4713, 1179.3929, 1232.3976, 1243.4194, 1249.4293, 1249.4293, 1261.4202, 1261.4202, 1265.4340, 1271.4703, 1275.4117, 1277.4208, 1277.4208, 1290.4227, 1315.4193, 1327.3777, 1329.4207, 1334.5732, 1343.3386, 1358.4282, 1385.4358, 1405.4801, 1406.5205, 1408.5166, 1413.4458, 1415.4503, 1417.4604, 1421.4934, 1428.5131, 1429.4515, 1434.5236, 1445.5238, 1455.5179, 1473.3993, 1474.4285, 1479.4886, 1480.5298, 1488.5660, 1489.5295, 1491.4669, 1493.4910, 1505.4945, 1538.5227, 1569.5825, 1573.5051, 1588.5730, 1588.5730, 1610.5535, 1612.6228, 1618.6089, 1632.7032, 1634.5774, 1665.6378, 1695.5259, 1699.5286, 1710.4990, 1716.5437, 1744.5569, 1812.5708, 1821.7052, 1844.5752, 1921.6616, 1993.6295, 2108.6680, 2108.6680, 2130.6443, 2220.7314, 2238.7197, 2238.7197, 2245.7749, 2269.8110, 2458.6926, 2548.6211, 2566.6006, 2566.6006, 2704.6790, 2913.9192, 3346.0308 | | | | | | | | | | | | | | | | | | | | | | | | | | | | | | | | | | | | | | | | | | | | | | | | | | | | | | | | | | | | | | | | | | | | | | | | | | | | | |  | | | | | | | | **S2** [**Cs2g02520.1**](http://zhangyang-pc/mascot/cgi/protein_view.pl?file=../data/20140118/F012097.dat&hit=1) **Mass: 43154 Score: 503 Expect: 2.2e-046 Matches: 24** | | | | | | | | | | | | | | | | | | | | | | | | | | | | | | | | | | | | | | | | | | | | | | | | | | | | | | | | | | | | | | | | | | | | | | | | |  | | | | | | | | | | | | | Observed | | | | | | | Mr(expt) | | | | | | | | Mr(calc) | | | | | | | | ppm | | | | | | | | Start | | | | | | | | |  | | | | | | | End | | | | | | | | Miss | | | | | | | | Ions | | | | | | | Peptide | | | |  | | | | | | | | | | | | 727.3119 | | | | | | | 726.3046 | | | | | | | | 726.4388 | | | | | | | | -184.73 | | | | | | | | 237 | | | | | | | | | - | | | | | | | 243 | | | | | | | | 0 | | | | | | | | --- | | | | | | | R.GTLPLAR.V | | | |  | | | | | | | | | | | | 819.3206 | | | | | | | 818.3133 | | | | | | | | 818.4538 | | | | | | | | -171.68 | | | | | | | | 318 | | | | | | | | | - | | | | | | | 324 | | | | | | | | 0 | | | | | | | | --- | | | | | | | K.IDLSPFK.G | | | |  | | | | | | | | | | | | 948.3238 | | | | | | | 947.3165 | | | | | | | | 947.4672 | | | | | | | | -159.05 | | | | | | | | 182 | | | | | | | | | - | | | | | | | 190 | | | | | | | | 0 | | | | | | | | --- | | | | | | | R.GLNATSETR.A | | | |  | | | | | | | | | | | | 970.3784 | | | | | | | 969.3711 | | | | | | | | 969.5495 | | | | | | | | -184.00 | | | | | | | | 340 | | | | | | | | | - | | | | | | | 348 | | | | | | | | 0 | | | | | | | | --- | | | | | | | K.TISSQPPLK.F | | | |  | | | | | | | | | | | | 1153.4329 | | | | | | | 1152.4256 | | | | | | | | 1152.6179 | | | | | | | | -166.83 | | | | | | | | 374 | | | | | | | | | - | | | | | | | 384 | | | | | | | | 0 | | | | | | | | --- | | | | | | | R.ADAGSVFVFIK.E | | | |  | | | | | | | | | | | | 1153.4329 | | | | | | | 1152.4256 | | | | | | | | 1152.6179 | | | | | | | | -166.83 | | | | | | | | 374 | | | | | | | | | - | | | | | | | 384 | | | | | | | | 0 | | | | | | | | 76 | | | | | | | R.ADAGSVFVFIK.E | | | |  | | | | | | | | | | | | 1243.3965 | | | | | | | 1242.3892 | | | | | | | | 1242.5881 | | | | | | | | -160.03 | | | | | | | | 171 | | | | | | | | | - | | | | | | | 181 | | | | | | | | 0 | | | | | | | | --- | | | | | | | K.ALVDSFYGTDR.G | | | |  | | | | | | | | | | | | 1243.3965 | | | | | | | 1242.3892 | | | | | | | | 1242.5881 | | | | | | | | -160.03 | | | | | | | | 171 | | | | | | | | | - | | | | | | | 181 | | | | | | | | 0 | | | | | | | | 75 | | | | | | | K.ALVDSFYGTDR.G | | | |  | | | | | | | | | | | | 1371.4803 | | | | | | | 1370.4730 | | | | | | | | 1370.6830 | | | | | | | | -153.21 | | | | | | | | 170 | | | | | | | | | - | | | | | | | 181 | | | | | | | | 1 | | | | | | | | --- | | | | | | | K.KALVDSFYGTDR.G | | | |  | | | | | | | | | | | | 1456.5857 | | | | | | | 1455.5784 | | | | | | | | 1455.8184 | | | | | | | | -164.85 | | | | | | | | 191 | | | | | | | | | - | | | | | | | 203 | | | | | | | | 0 | | | | | | | | --- | | | | | | | R.AEIVELITQLEAK.N | | | |  | | | | | | | | | | | | 1456.5857 | | | | | | | 1455.5784 | | | | | | | | 1455.8184 | | | | | | | | -164.85 | | | | | | | | 191 | | | | | | | | | - | | | | | | | 203 | | | | | | | | 0 | | | | | | | | 92 | | | | | | | R.AEIVELITQLEAK.N | | | |  | | | | | | | | | | | | 1462.5552 | | | | | | | 1461.5479 | | | | | | | | 1461.7675 | | | | | | | | -150.20 | | | | | | | | 325 | | | | | | | | | - | | | | | | | 339 | | | | | | | | 0 | | | | | | | | --- | | | | | | | K.GILSSVQDTASSVAK.T | | | |  | | | | | | | | | | | | 1478.5587 | | | | | | | 1477.5514 | | | | | | | | 1477.7776 | | | | | | | | -153.08 | | | | | | | | 264 | | | | | | | | | - | | | | | | | 278 | | | | | | | | 0 | | | | | | | | --- | | | | | | | R.FAGPLATTSISTNAK.F | | | |  | | | | | | | | | | | | 1515.4890 | | | | | | | 1514.4817 | | | | | | | | 1514.7100 | | | | | | | | -150.70 | | | | | | | | 146 | | | | | | | | | - | | | | | | | 160 | | | | | | | | 0 | | | | | | | | --- | | | | | | | K.EEGGALAVAEEESPK.E | | | |  | | | | | | | | | | | | 1650.6346 | | | | | | | 1649.6273 | | | | | | | | 1649.8988 | | | | | | | | -164.54 | | | | | | | | 204 | | | | | | | | | - | | | | | | | 219 | | | | | | | | 0 | | | | | | | | --- | | | | | | | K.NPTPAPTEALTLLNAK.W | | | |  | | | | | | | | | | | | 1650.6346 | | | | | | | 1649.6273 | | | | | | | | 1649.8988 | | | | | | | | -164.54 | | | | | | | | 204 | | | | | | | | | - | | | | | | | 219 | | | | | | | | 0 | | | | | | | | 52 | | | | | | | K.NPTPAPTEALTLLNAK.W | | | |  | | | | | | | | | | | | 1772.5730 | | | | | | | 1771.5657 | | | | | | | | 1771.8475 | | | | | | | | -159.05 | | | | | | | | 144 | | | | | | | | | - | | | | | | | 160 | | | | | | | | 1 | | | | | | | | --- | | | | | | | K.EKEEGGALAVAEEESPK.E | | | |  | | | | | | | | | | | | 1993.6686 | | | | | | | 1992.6613 | | | | | | | | 1993.0231 | | | | | | | | -181.50 | | | | | | | | 374 | | | | | | | | | - | | | | | | | 392 | | | | | | | | 1 | | | | | | | | --- | | | | | | | R.ADAGSVFVFIKEGSPLLMP.- + Oxidation (M) | | | |  | | | | | | | | | | | | 2009.7635 | | | | | | | 2008.7562 | | | | | | | | 2009.0582 | | | | | | | | -150.30 | | | | | | | | 264 | | | | | | | | | - | | | | | | | 282 | | | | | | | | 1 | | | | | | | | --- | | | | | | | R.FAGPLATTSISTNAKFEVR.S | | | |  | | | | | | | | | | | | 2308.7515 | | | | | | | 2307.7442 | | | | | | | | 2308.1183 | | | | | | | | -162.06 | | | | | | | | 244 | | | | | | | | | - | | | | | | | 263 | | | | | | | | 0 | | | | | | | | --- | | | | | | | R.VEEISQTIDSENFTVQNSIR.F | | | |  | | | | | | | | | | | | 2308.7515 | | | | | | | 2307.7442 | | | | | | | | 2308.1183 | | | | | | | | -162.06 | | | | | | | | 244 | | | | | | | | | - | | | | | | | 263 | | | | | | | | 0 | | | | | | | | 87 | | | | | | | R.VEEISQTIDSENFTVQNSIR.F | | | |  | | | | | | | | | | | | 2385.9204 | | | | | | | 2384.9131 | | | | | | | | 2385.2751 | | | | | | | | -151.74 | | | | | | | | 182 | | | | | | | | | - | | | | | | | 203 | | | | | | | | 1 | | | | | | | | --- | | | | | | | R.GLNATSETRAEIVELITQLEAK.N | | | |  | | | | | | | | | | | | 2959.0369 | | | | | | | 2958.0296 | | | | | | | | 2958.5226 | | | | | | | | -166.64 | | | | | | | | 291 | | | | | | | | | - | | | | | | | 317 | | | | | | | | 0 | | | | | | | | --- | | | | | | | K.FEEGIIGTPQVTDSLVIPENVEFLGQK.I | | | |  | | | | | | | | | | | | 2959.0369 | | | | | | | 2958.0296 | | | | | | | | 2958.5226 | | | | | | | | -166.64 | | | | | | | | 291 | | | | | | | | | - | | | | | | | 317 | | | | | | | | 0 | | | | | | | | 36 | | | | | | | K.FEEGIIGTPQVTDSLVIPENVEFLGQK.I | | | |  | | | | | | | | | | | | No match to: 705.2631, 713.2986, 745.2631, 795.2848, 841.2963, 842.3644, 850.3677, 968.3930, 1037.3324, 1059.4182, 1138.4169, 1148.4336, 1150.4280, 1151.4486, 1160.4359, 1175.4113, 1197.3853, 1206.4492, 1225.4032, 1236.3320, 1238.4427, 1242.3756, 1248.3080, 1265.3649, 1270.3088, 1277.4264, 1324.5240, 1326.4694, 1351.5070, 1351.5070, 1369.3258, 1375.3984, 1383.5309, 1406.5128, 1434.5709, 1448.4901, 1470.6136, 1491.4867, 1505.4541, 1572.5006, 1628.6078, 1633.6193, 1645.6204, 1646.6211, 1663.7521, 1672.6342, 1694.5490, 1855.6563, 1855.6563, 2002.7837, 2028.6869, 2050.8926, 2050.8926, 2083.4727, 2142.8445, 2209.6958, 2220.7449, 2226.7288, 2238.7727, 2245.7832, 2264.7778, 2290.7786, 2293.7898, 2306.7151, 2307.7473, 2311.3025, 2322.7981, 2330.7324, 2336.7766, 2346.7417, 2350.7288, 2352.8057, 2568.7649, 2576.8484, 2576.8484, 2588.8496, 2604.8354, 2619.8594, 2628.8445, 2704.8772, 2725.9319, 2794.0662, 2845.7461, 2981.0229, 3001.8870, 3353.1482 | | | | | | | | | | | | | | | | | | | | | | | | | | | | | | | | | | | | | | | | | | | | | | | | | | | | | | | | | | | | | | | | | | | | | | | | | | | | | |  | | | | | | | | **S7** [**Cs2g06360.1**](http://zhangyang-pc/mascot/cgi/protein_view.pl?file=../data/20140118/F012109.dat&hit=1) **Mass: 34917 Score: 352 Expect: 2.8e-031 Matches: 21** | | | | | | | | | | | | | | | | | | | | | | | | | | | | | | | | | | | | | | | | | | | | | | | | | | | | | | | | | | | | | | | | | | | | | | | | | | | | | |  | | | | | | | | Observed | | | | | | Mr(expt) | | | | | | | | Mr(calc) | | | | | | | | ppm | | | | | | | | Start | | | | | | | | |  | | | | | | | End | | | | | | | | Miss | | | | | | | | Ions | | | | | | | | Peptide | | | | | | | | | | | | | | | | 765.2009 | | | | | | 764.1936 | | | | | | | | 764.3453 | | | | | | | | -198.49 | | | | | | | | 156 | | | | | | | | | - | | | | | | | 162 | | | | | | | | 0 | | | | | | | | --- | | | | | | | | R.AEADFGR.H | | | | | | | | | | | | | | | | 772.2816 | | | | | | 771.2743 | | | | | | | | 771.3664 | | | | | | | | -119.39 | | | | | | | | 150 | | | | | | | | | - | | | | | | | 155 | | | | | | | | 0 | | | | | | | | --- | | | | | | | | R.WQEPGR.A | | | | | | | | | | | | | | | | 805.2531 | | | | | | 804.2458 | | | | | | | | 804.4130 | | | | | | | | -207.79 | | | | | | | | 55 | | | | | | | | | - | | | | | | | 61 | | | | | | | | 0 | | | | | | | | --- | | | | | | | | R.AIAPDYR.G | | | | | | | | | | | | | | | | 947.3507 | | | | | | 946.3434 | | | | | | | | 946.5236 | | | | | | | | -190.35 | | | | | | | | 223 | | | | | | | | | - | | | | | | | 230 | | | | | | | | 0 | | | | | | | | --- | | | | | | | | R.TALQVPYR.S | | | | | | | | | | | | | | | | 1025.3898 | | | | | | 1024.3825 | | | | | | | | 1024.5705 | | | | | | | | -183.51 | | | | | | | | 171 | | | | | | | | | - | | | | | | | 178 | | | | | | | | 0 | | | | | | | | --- | | | | | | | | R.NIYILFSR.S | | | | | | | | | | | | | | | | 1033.3723 | | | | | | 1032.3650 | | | | | | | | 1032.5855 | | | | | | | | -213.56 | | | | | | | | 236 | | | | | | | | | - | | | | | | | 244 | | | | | | | | 0 | | | | | | | | --- | | | | | | | | K.FSLPELTVK.V | | | | | | | | | | | | | | | | 1052.4810 | | | | | | 1051.4737 | | | | | | | | 1051.6641 | | | | | | | | -181.03 | | | | | | | | 245 | | | | | | | | | - | | | | | | | 254 | | | | | | | | 0 | | | | | | | | --- | | | | | | | | K.VPALLILGEK.D | | | | | | | | | | | | | | | | 1109.3578 | | | | | | 1108.3505 | | | | | | | | 1108.5553 | | | | | | | | -184.72 | | | | | | | | 260 | | | | | | | | | - | | | | | | | 268 | | | | | | | | 0 | | | | | | | | --- | | | | | | | | K.FPGIEDYIR.S | | | | | | | | | | | | | | | | 1109.3578 | | | | | | 1108.3505 | | | | | | | | 1108.5553 | | | | | | | | -184.72 | | | | | | | | 260 | | | | | | | | | - | | | | | | | 268 | | | | | | | | 0 | | | | | | | | 73 | | | | | | | | K.FPGIEDYIR.S | | | | | | | | | | | | | | | | 1168.3861 | | | | | | 1167.3788 | | | | | | | | 1167.5924 | | | | | | | | -182.93 | | | | | | | | 140 | | | | | | | | | - | | | | | | | 149 | | | | | | | | 0 | | | | | | | | --- | | | | | | | | K.SLPEGFYISR.W | | | | | | | | | | | | | | | | 1168.3861 | | | | | | 1167.3788 | | | | | | | | 1167.5924 | | | | | | | | -182.93 | | | | | | | | 140 | | | | | | | | | - | | | | | | | 149 | | | | | | | | 0 | | | | | | | | 68 | | | | | | | | K.SLPEGFYISR.W | | | | | | | | | | | | | | | | 1215.4539 | | | | | | 1214.4466 | | | | | | | | 1214.6659 | | | | | | | | -180.53 | | | | | | | | 274 | | | | | | | | | - | | | | | | | 283 | | | | | | | | 0 | | | | | | | | --- | | | | | | | | K.DFVPNLEIIR.L | | | | | | | | | | | | | | | | 1215.4539 | | | | | | 1214.4466 | | | | | | | | 1214.6659 | | | | | | | | -180.53 | | | | | | | | 274 | | | | | | | | | - | | | | | | | 283 | | | | | | | | 0 | | | | | | | | 68 | | | | | | | | K.DFVPNLEIIR.L | | | | | | | | | | | | | | | | 1273.4100 | | | | | | 1272.4027 | | | | | | | | 1272.6397 | | | | | | | | -186.23 | | | | | | | | 43 | | | | | | | | | - | | | | | | | 54 | | | | | | | | 0 | | | | | | | | --- | | | | | | | | R.HQMVAVAAAGFR.A + Oxidation (M) | | | | | | | | | | | | | | | | 1359.3893 | | | | | | 1358.3820 | | | | | | | | 1358.6354 | | | | | | | | -186.49 | | | | | | | | 62 | | | | | | | | | - | | | | | | | 74 | | | | | | | | 0 | | | | | | | | --- | | | | | | | | R.GYGLSDPPAEPEK.A | | | | | | | | | | | | | | | | 1442.5801 | | | | | | 1441.5728 | | | | | | | | 1441.8293 | | | | | | | | -177.87 | | | | | | | | 272 | | | | | | | | | - | | | | | | | 283 | | | | | | | | 1 | | | | | | | | --- | | | | | | | | K.VKDFVPNLEIIR.L | | | | | | | | | | | | | | | | 1442.5801 | | | | | | 1441.5728 | | | | | | | | 1441.8293 | | | | | | | | -177.87 | | | | | | | | 272 | | | | | | | | | - | | | | | | | 283 | | | | | | | | 1 | | | | | | | | 60 | | | | | | | | K.VKDFVPNLEIIR.L | | | | | | | | | | | | | | | | 1480.5813 | | | | | | 1479.5740 | | | | | | | | 1479.8562 | | | | | | | | -190.66 | | | | | | | | 167 | | | | | | | | | - | | | | | | | 178 | | | | | | | | 1 | | | | | | | | --- | | | | | | | | K.TVVRNIYILFSR.S | | | | | | | | | | | | | | | | 1480.5813 | | | | | | 1479.5740 | | | | | | | | 1479.8562 | | | | | | | | -190.66 | | | | | | | | 167 | | | | | | | | | - | | | | | | | 178 | | | | | | | | 1 | | | | | | | | --- | | | | | | | | K.TVVRNIYILFSR.S | | | | | | | | | | | | | | | | 1865.6299 | | | | | | 1864.6226 | | | | | | | | 1864.9894 | | | | | | | | -196.68 | | | | | | | | 79 | | | | | | | | | - | | | | | | | 95 | | | | | | | | 0 | | | | | | | | --- | | | | | | | | K.DITNDLLATLDHLGINK.V | | | | | | | | | | | | | | | | 2165.7847 | | | | | | 2164.7774 | | | | | | | | 2165.1885 | | | | | | | | -189.86 | | | | | | | | 119 | | | | | | | | | - | | | | | | | 139 | | | | | | | | 0 | | | | | | | | --- | | | | | | | | R.VSGVITLGVPFIPPGTAEFHK.S | | | | | | | | | | | | | | | | No match to: 700.2496, 705.2460, 713.2723, 716.2323, 718.2423, 734.3412, 768.3577, 770.2911, 776.2120, 788.2150, 795.2799, 804.2191, 822.2637, 824.2902, 834.3317, 842.3432, 850.3602, 856.3321, 859.2739, 860.9050, 868.3739, 877.2830, 882.3845, 886.2788, 889.2757, 889.2757, 905.2753, 929.2775, 966.2997, 981.3027, 982.3483, 987.3339, 988.3514, 1030.3555, 1045.3300, 1051.4999, 1054.3754, 1056.3494, 1057.3684, 1059.3618, 1075.3159, 1082.3265, 1095.4197, 1121.3604, 1121.3604, 1123.3573, 1126.3495, 1128.3322, 1128.3322, 1137.3613, 1139.3643, 1147.3779, 1150.3243, 1151.3390, 1159.3763, 1165.4136, 1167.3115, 1172.3630, 1175.3710, 1184.3663, 1190.3556, 1199.3430, 1234.4039, 1249.4316, 1254.3859, 1265.4077, 1289.3936, 1315.3932, 1348.4578, 1370.4443, 1387.4701, 1387.4701, 1424.4786, 1426.5293, 1426.5293, 1434.5818, 1438.5133, 1452.5431, 1463.5382, 1502.5327, 1543.5392, 1669.5902, 1691.5834, 2002.6654, 2238.7114, 2245.7773, 2326.7295, 3002.9902, 3346.0542 | | | | | | | | | | | | | | | | | | | | | | | | | | | | | | | | | | | | | | | | | | | | | | | | | | | | | | | | | | | | | | | | | | | | | | | | | | | | | | |  | | | | | | | **S31** [**Cs8g20610.1**](http://zhangyang-pc/mascot/cgi/protein_view.pl?file=../data/20140118/F012136.dat&hit=1) **Mass: 35461 Score: 220 Expect: 4.4e-018 Matches: 14** | | | | | | | | | | | | | | | | | | | | | | | | | | | | | | | | | | | | | | | | | | | | | | | | | | | | | | | | | | | | | | | | | | | | | | | | | | | | | | |  | | | | | | | Observed | | | | | | Mr(expt) | | | | | | | | Mr(calc) | | | | | | | | ppm | | | | | | | | Start | | | | | | | | |  | | | | | | | End | | | | | | | | Miss | | | | | | | | Ions | | | | | | | | Peptide | | | | | | | | | | | | | | | | 733.2114 | | | | | | 732.2042 | | | | | | | | 732.3402 | | | | | | | | -185.78 | | | | | | | | 38 | | | | | | | | | - | | | | | | | 43 | | | | | | | | 0 | | | | | | | | --- | | | | | | | | K.NLSDER.E | | | | | | | | | | | | | | | | 832.2594 | | | | | | 831.2521 | | | | | | | | 831.4086 | | | | | | | | -188.24 | | | | | | | | 50 | | | | | | | | | - | | | | | | | 57 | | | | | | | | 0 | | | | | | | | --- | | | | | | | | K.ALEGADTR.L | | | | | | | | | | | | | | | | 899.3247 | | | | | | 898.3174 | | | | | | | | 898.4872 | | | | | | | | -189.04 | | | | | | | | 280 | | | | | | | | | - | | | | | | | 287 | | | | | | | | 0 | | | | | | | | --- | | | | | | | | K.DTQPGLLR.T | | | | | | | | | | | | | | | | 899.3247 | | | | | | 898.3174 | | | | | | | | 898.4872 | | | | | | | | -189.04 | | | | | | | | 280 | | | | | | | | | - | | | | | | | 287 | | | | | | | | 0 | | | | | | | | 49 | | | | | | | | K.DTQPGLLR.T | | | | | | | | | | | | | | | | 918.3199 | | | | | | 917.3127 | | | | | | | | 917.4971 | | | | | | | | -200.98 | | | | | | | | 30 | | | | | | | | | - | | | | | | | 37 | | | | | | | | 0 | | | | | | | | --- | | | | | | | | R.YTVHATVK.N | | | | | | | | | | | | | | | | 1074.4209 | | | | | | 1073.4136 | | | | | | | | 1073.5982 | | | | | | | | -171.90 | | | | | | | | 29 | | | | | | | | | - | | | | | | | 37 | | | | | | | | 1 | | | | | | | | --- | | | | | | | | R.RYTVHATVK.N | | | | | | | | | | | | | | | | 1128.3455 | | | | | | 1127.3382 | | | | | | | | 1127.6299 | | | | | | | | -258.63 | | | | | | | | 280 | | | | | | | | | - | | | | | | | 289 | | | | | | | | 1 | | | | | | | | --- | | | | | | | | K.DTQPGLLRTK.D | | | | | | | | | | | | | | | | 1464.4702 | | | | | | 1463.4629 | | | | | | | | 1463.7296 | | | | | | | | -182.20 | | | | | | | | 265 | | | | | | | | | - | | | | | | | 276 | | | | | | | | 0 | | | | | | | | --- | | | | | | | | K.VAELYPEYDIPR.L | | | | | | | | | | | | | | | | 1464.4702 | | | | | | 1463.4629 | | | | | | | | 1463.7296 | | | | | | | | -182.20 | | | | | | | | 265 | | | | | | | | | - | | | | | | | 276 | | | | | | | | 0 | | | | | | | | 77 | | | | | | | | K.VAELYPEYDIPR.L | | | | | | | | | | | | | | | | 1564.5430 | | | | | | 1563.5357 | | | | | | | | 1563.8257 | | | | | | | | -185.40 | | | | | | | | 93 | | | | | | | | | - | | | | | | | 106 | | | | | | | | 0 | | | | | | | | --- | | | | | | | | K.VEDPQNQLLNPAVK.G | | | | | | | | | | | | | | | | 1564.5430 | | | | | | 1563.5357 | | | | | | | | 1563.8257 | | | | | | | | -185.40 | | | | | | | | 93 | | | | | | | | | - | | | | | | | 106 | | | | | | | | 0 | | | | | | | | 57 | | | | | | | | K.VEDPQNQLLNPAVK.G | | | | | | | | | | | | | | | | 1906.6249 | | | | | | 1905.6176 | | | | | | | | 1905.9944 | | | | | | | | -197.67 | | | | | | | | 295 | | | | | | | | | - | | | | | | | 310 | | | | | | | | 0 | | | | | | | | --- | | | | | | | | K.LMDLGLQFIPMDQIIK.D + 2 Oxidation (M) | | | | | | | | | | | | | | | | 2002.6957 | | | | | | 2001.6884 | | | | | | | | 2002.0995 | | | | | | | | -205.32 | | | | | | | | 294 | | | | | | | | | - | | | | | | | 310 | | | | | | | | 1 | | | | | | | | --- | | | | | | | | K.KLMDLGLQFIPMDQIIK.D | | | | | | | | | | | | | | | | 2034.6997 | | | | | | 2033.6924 | | | | | | | | 2034.0893 | | | | | | | | -195.13 | | | | | | | | 294 | | | | | | | | | - | | | | | | | 310 | | | | | | | | 1 | | | | | | | | --- | | | | | | | | K.KLMDLGLQFIPMDQIIK.D + 2 Oxidation (M) | | | | | | | | | | | | | | | | No match to: 700.2574, 701.2662, 705.2416, 706.2422, 713.2729, 716.2345, 730.2519, 736.3417, 744.2495, 750.3256, 768.3630, 772.2553, 793.2715, 807.2701, 826.2534, 827.2489, 834.3071, 838.2407, 841.2245, 842.3377, 846.2980, 850.3409, 854.2501, 854.2501, 862.3005, 868.3447, 882.3689, 884.2819, 890.3141, 910.2546, 916.3101, 994.3644, 1019.3561, 1020.3688, 1050.3263, 1054.3521, 1059.3783, 1096.4364, 1126.3245, 1139.4006, 1147.4415, 1151.4755, 1153.3763, 1159.4359, 1159.4359, 1165.4254, 1179.3750, 1216.4789, 1221.4736, 1225.4795, 1264.3862, 1276.3917, 1281.3899, 1287.4465, 1287.4465, 1292.3661, 1293.3967, 1302.3588, 1304.4569, 1316.3693, 1328.4263, 1344.4915, 1378.4441, 1387.4441, 1391.4623, 1394.3789, 1418.4536, 1424.4595, 1444.4204, 1463.4727, 1475.5044, 1478.4833, 1478.4833, 1486.4570, 1498.5238, 1498.5238, 1502.4991, 1534.5187, 1574.5331, 1581.5280, 1583.5632, 1607.4812, 1629.5043, 1817.5221, 1833.4760, 1836.6130, 1893.6553, 1893.6553, 1922.6379, 1960.6604, 1986.6956, 1988.6859, 1988.6859, 2238.7046, 2245.7847, 2366.7319 | | | | | | | | | | | | | | | | | | | | | | | | | | | | | | | | | | | | | | | | | | | | | | | | | | | | | | | | | | | | | | | | | | | | | | | | | | | | | | |  | | | | | | | **S15** [**Cs6g09150.2**](http://zhangyang-pc/mascot/cgi/protein_view.pl?file=../data/20140118/F012101.dat&hit=1) **Mass: 28953 Score: 88 Expect: 6.5e-005 Matches: 8** | | | | | | | | | | | | | | | | | | | | | | | | | | | | | | | | | | | | | | | | | | | | | | | | | | | | | | | | | | | | | | | | | | | | | | | | | | | | | | |  | | | | | | | Observed | | | | | | | Mr(expt) | | | | | | | | Mr(calc) | | | | | | | | ppm | | | | | | | | Start | | | | | | | | |  | | | | | | | End | | | | | | | | Miss | | | | | | | | Ions | | | | | | | Peptide | | | | | | | | | | | | | | | | 765.2982 | | | | | | | 764.2909 | | | | | | | | 764.4181 | | | | | | | | -166.39 | | | | | | | | 234 | | | | | | | | | - | | | | | | | 239 | | | | | | | | 0 | | | | | | | | --- | | | | | | | K.YVSQLR.M | | | | | | | | | | | | | | | | 1116.3505 | | | | | | | 1115.3432 | | | | | | | | 1115.5070 | | | | | | | | -146.78 | | | | | | | | 148 | | | | | | | | | - | | | | | | | 155 | | | | | | | | 0 | | | | | | | | --- | | | | | | | K.FMEYQNLR.G + Oxidation (M) | | | | | | | | | | | | | | | | 1116.3505 | | | | | | | 1115.3432 | | | | | | | | 1115.5070 | | | | | | | | -146.78 | | | | | | | | 148 | | | | | | | | | - | | | | | | | 155 | | | | | | | | 0 | | | | | | | | 7 | | | | | | | K.FMEYQNLR.G + Oxidation (M) | | | | | | | | | | | | | | | | 1163.3953 | | | | | | | 1162.3880 | | | | | | | | 1162.6193 | | | | | | | | -198.95 | | | | | | | | 2 | | | | | | | | | - | | | | | | | 14 | | | | | | | | 0 | | | | | | | | --- | | | | | | | M.ALAASSSSSLAAK.Q | | | | | | | | | | | | | | | | 1607.6997 | | | | | | | 1606.6924 | | | | | | | | 1606.9294 | | | | | | | | -147.47 | | | | | | | | 74 | | | | | | | | | - | | | | | | | 88 | | | | | | | | 0 | | | | | | | | --- | | | | | | | K.EVLDVPVSPLLSLAR.Q | | | | | | | | | | | | | | | | 1607.6997 | | | | | | | 1606.6924 | | | | | | | | 1606.9294 | | | | | | | | -147.47 | | | | | | | | 74 | | | | | | | | | - | | | | | | | 88 | | | | | | | | 0 | | | | | | | | 64 | | | | | | | K.EVLDVPVSPLLSLAR.Q | | | | | | | | | | | | | | | | 1863.5760 | | | | | | | 1862.5687 | | | | | | | | 1863.0829 | | | | | | | | -276.00 | | | | | | | | 74 | | | | | | | | | - | | | | | | | 90 | | | | | | | | 1 | | | | | | | | --- | | | | | | | K.EVLDVPVSPLLSLARQK.Y | | | | | | | | | | | | | | | | 1881.6146 | | | | | | | 1880.6073 | | | | | | | | 1880.8727 | | | | | | | | -141.08 | | | | | | | | 161 | | | | | | | | | - | | | | | | | 176 | | | | | | | | 0 | | | | | | | | --- | | | | | | | K.LHSIMQPPSEFDHAEK.G + Oxidation (M) | | | | | | | | | | | | | | | | No match to: 834.2842, 842.3781, 944.4067, 964.3853, 970.3928, 987.3410, 987.3410, 996.3446, 998.3409, 999.3367, 999.3367, 1014.3793, 1015.3422, 1018.3510, 1037.3234, 1039.3320, 1055.3711, 1059.3679, 1067.3518, 1071.3558, 1072.3959, 1132.3494, 1155.3821, 1222.3861, 1246.4341, 1263.4054, 1320.4625, 1322.5021, 1337.4906, 1392.4423, 1394.4395, 1398.4277, 1398.4277, 1406.3960, 1408.4188, 1410.4221, 1410.4221, 1422.4543, 1424.4148, 1426.4176, 1426.4176, 1444.3937, 1448.4209, 1450.4346, 1465.4635, 1477.5081, 1520.3708, 1553.4348, 1617.5736, 1647.5824, 1650.4625, 1702.5184, 1702.5184, 1817.6219, 1824.6232, 1831.7467, 1832.6946, 1844.1125, 1844.7129, 1844.7129, 1848.7030, 1860.6899, 1861.6904, 1876.6567, 1892.6477, 1897.6315, 1921.7288, 1935.6830, 1939.6395, 1951.6411, 1955.6454, 1965.6735, 1967.6672, 2015.7039, 2025.6862, 2058.6768, 2063.6687, 2073.6411, 2075.6548, 2075.6548, 2091.6763, 2097.6360, 2106.6599, 2113.6311, 2140.7090, 2155.7207, 2158.7424, 2228.6965, 2238.7783, 2245.8040, 2288.7319, 2297.7134, 2309.7271, 2325.7114, 2357.8054, 2597.7908, 3112.0530, 3124.0112, 3139.9946, 3377.9604, 3393.9692, 3528.1611 | | | | | | | | | | | | | | | | | | | | | | | | | | | | | | | | | | | | | | | | | | | | | | | | | | | | | | | | | | | | | | | | | | | | | | | | | | | | | | | |  | | | | | | **S38** [**Cs1g20220.1**](http://zhangyang-pc/mascot/cgi/protein_view.pl?file=../data/20140118/F012095.dat&hit=1) **Mass: 27671 Score: 339 Expect: 5.6e-030 Matches: 20** | | | | | | | | | | | | | | | | | | | | | | | | | | | | | | | | | | | | | | | | | | | | | | | | | | | | | | | | | | | | | | | | | | | | | | | | | | | | | | | |  | | | | | | Observed | | | | | | | Mr(expt) | | | | | | | | Mr(calc) | | | | | | | | ppm | | | | | | | | Start | | | | | | | | |  | | | | | | | End | | | | | | | | Miss | | | | | | | | Ions | | | | | | | Peptide | | | | | | | | | | | | | | | | 758.3190 | | | | | | | 757.3117 | | | | | | | | 757.4446 | | | | | | | | -175.50 | | | | | | | | 56 | | | | | | | | | - | | | | | | | 62 | | | | | | | | 0 | | | | | | | | --- | | | | | | | K.NVIGSLR.A | | | | | | | | | | | | | | | | 770.2540 | | | | | | | 769.2468 | | | | | | | | 769.4010 | | | | | | | | -200.50 | | | | | | | | 136 | | | | | | | | | - | | | | | | | 141 | | | | | | | | 0 | | | | | | | | --- | | | | | | | R.YLAEFK.V | | | | | | | | | | | | | | | | 814.2329 | | | | | | | 813.2256 | | | | | | | | 813.3578 | | | | | | | | -162.54 | | | | | | | | 22 | | | | | | | | | - | | | | | | | 27 | | | | | | | | 0 | | | | | | | | --- | | | | | | | R.YEEMVK.F + Oxidation (M) | | | | | | | | | | | | | | | | 816.2803 | | | | | | | 815.2730 | | | | | | | | 815.4137 | | | | | | | | -172.55 | | | | | | | | 15 | | | | | | | | | - | | | | | | | 21 | | | | | | | | 0 | | | | | | | | --- | | | | | | | K.LAEQAER.Y | | | | | | | | | | | | | | | | 907.3745 | | | | | | | 906.3672 | | | | | | | | 906.5174 | | | | | | | | -165.76 | | | | | | | | 48 | | | | | | | | | - | | | | | | | 55 | | | | | | | | 0 | | | | | | | | --- | | | | | | | R.NLLSVAYK.N | | | | | | | | | | | | | | | | 917.3741 | | | | | | | 916.3668 | | | | | | | | 916.5229 | | | | | | | | -170.33 | | | | | | | | 67 | | | | | | | | | - | | | | | | | 74 | | | | | | | | 0 | | | | | | | | --- | | | | | | | R.IISSIEQK.E | | | | | | | | | | | | | | | | 944.3731 | | | | | | | 943.3658 | | | | | | | | 943.5201 | | | | | | | | -163.56 | | | | | | | | 124 | | | | | | | | | - | | | | | | | 130 | | | | | | | | 1 | | | | | | | | --- | | | | | | | K.VFYLKMK.G + Oxidation (M) | | | | | | | | | | | | | | | | 948.2704 | | | | | | | 947.2632 | | | | | | | | 947.4171 | | | | | | | | -162.45 | | | | | | | | 129 | | | | | | | | | - | | | | | | | 135 | | | | | | | | 1 | | | | | | | | --- | | | | | | | K.MKGDYYR.Y + Oxidation (M) | | | | | | | | | | | | | | | | 1013.3508 | | | | | | | 1012.3435 | | | | | | | | 1012.5229 | | | | | | | | -177.18 | | | | | | | | 7 | | | | | | | | | - | | | | | | | 14 | | | | | | | | 0 | | | | | | | | --- | | | | | | | R.EQYVYLAK.L | | | | | | | | | | | | | | | | 1205.4609 | | | | | | | 1204.4536 | | | | | | | | 1204.6485 | | | | | | | | -161.81 | | | | | | | | 221 | | | | | | | | | - | | | | | | | 230 | | | | | | | | 0 | | | | | | | | --- | | | | | | | K.DSTLIMQLLR.D + Oxidation (M) | | | | | | | | | | | | | | | | 1205.4609 | | | | | | | 1204.4536 | | | | | | | | 1204.6485 | | | | | | | | -161.81 | | | | | | | | 221 | | | | | | | | | - | | | | | | | 230 | | | | | | | | 0 | | | | | | | | 37 | | | | | | | K.DSTLIMQLLR.D + Oxidation (M) | | | | | | | | | | | | | | | | 1388.5262 | | | | | | | 1387.5189 | | | | | | | | 1387.7307 | | | | | | | | -152.58 | | | | | | | | 67 | | | | | | | | | - | | | | | | | 78 | | | | | | | | 1 | | | | | | | | --- | | | | | | | R.IISSIEQKEEGR.K | | | | | | | | | | | | | | | | 1388.5262 | | | | | | | 1387.5189 | | | | | | | | 1387.7307 | | | | | | | | -152.58 | | | | | | | | 67 | | | | | | | | | - | | | | | | | 78 | | | | | | | | 1 | | | | | | | | 43 | | | | | | | R.IISSIEQKEEGR.K | | | | | | | | | | | | | | | | 1624.5333 | | | | | | | 1623.5260 | | | | | | | | 1623.8468 | | | | | | | | -197.53 | | | | | | | | 108 | | | | | | | | | - | | | | | | | 123 | | | | | | | | 0 | | | | | | | | --- | | | | | | | K.LLDSHLVPSATAGESK.V | | | | | | | | | | | | | | | | 1646.6378 | | | | | | | 1645.6305 | | | | | | | | 1645.9515 | | | | | | | | -195.01 | | | | | | | | 48 | | | | | | | | | - | | | | | | | 62 | | | | | | | | 1 | | | | | | | | --- | | | | | | | R.NLLSVAYKNVIGSLR.A | | | | | | | | | | | | | | | | 1802.6925 | | | | | | | 1801.6852 | | | | | | | | 1801.9686 | | | | | | | | -157.27 | | | | | | | | 159 | | | | | | | | | - | | | | | | | 175 | | | | | | | | 0 | | | | | | | | --- | | | | | | | K.AAQDIALTDLAPTHPIR.L | | | | | | | | | | | | | | | | 1802.6925 | | | | | | | 1801.6852 | | | | | | | | 1801.9686 | | | | | | | | -157.27 | | | | | | | | 159 | | | | | | | | | - | | | | | | | 175 | | | | | | | | 0 | | | | | | | | 106 | | | | | | | K.AAQDIALTDLAPTHPIR.L | | | | | | | | | | | | | | | | 2142.6558 | | | | | | | 2141.6485 | | | | | | | | 2142.0004 | | | | | | | | -164.28 | | | | | | | | 202 | | | | | | | | | - | | | | | | | 220 | | | | | | | | 0 | | | | | | | | --- | | | | | | | K.QAFEEAIAELDTLGEESYK.D | | | | | | | | | | | | | | | | 2228.6938 | | | | | | | 2227.6865 | | | | | | | | 2228.0518 | | | | | | | | -163.97 | | | | | | | | 28 | | | | | | | | | - | | | | | | | 47 | | | | | | | | 0 | | | | | | | | --- | | | | | | | K.FMDSLVTSSTPATELTVEER.N + Oxidation (M) | | | | | | | | | | | | | | | | 2228.6938 | | | | | | | 2227.6865 | | | | | | | | 2228.0518 | | | | | | | | -163.97 | | | | | | | | 28 | | | | | | | | | - | | | | | | | 47 | | | | | | | | 0 | | | | | | | | 76 | | | | | | | K.FMDSLVTSSTPATELTVEER.N + Oxidation (M) | | | | | | | | | | | | | | | | No match to: 705.2545, 713.2607, 717.2396, 728.2952, 730.2062, 734.3315, 832.2978, 842.3697, 850.4000, 868.3581, 882.3722, 894.3691, 964.3387, 982.3724, 987.3332, 999.3294, 1014.3709, 1015.3361, 1041.3535, 1053.3606, 1055.3638, 1072.3765, 1126.3645, 1141.4756, 1142.4701, 1151.4276, 1160.4469, 1163.3827, 1179.3901, 1204.4045, 1210.3785, 1220.3846, 1221.4576, 1222.3749, 1222.3749, 1227.4291, 1236.3602, 1238.3699, 1279.3759, 1298.4445, 1320.4180, 1337.4553, 1368.4795, 1398.4080, 1406.4036, 1408.4131, 1410.4146, 1410.4146, 1413.4517, 1418.4711, 1421.5229, 1421.5229, 1425.4808, 1426.4115, 1432.4272, 1444.4408, 1555.5135, 1597.5210, 1605.5342, 1617.5562, 1617.5562, 1631.5565, 1633.5570, 1635.5538, 1647.5638, 1647.5638, 1663.5531, 1667.4944, 1728.4438, 1744.5269, 1801.6664, 1816.6385, 1824.6091, 1832.6053, 2075.6384, 2124.6492, 2125.6396, 2139.7607, 2158.7300, 2163.7979, 2164.7063, 2210.7019, 2238.7466, 2242.7310, 2244.7109, 2244.7109, 2308.7561, 3378.9321, 3392.9492, 3408.9221 | | | | | | | | | | | | | | | | | | | | | | | | | | | | | | | | | | | | | | | | | | | | | | | | | | | | | | | | | | | | | | | | | | | | | | | | | | | | | | | | |  | | | | | **S52** [**Cs5g09380.2**](http://zhangyang-pc/mascot/cgi/protein_view.pl?file=../data/20140118/F012118.dat&hit=1) **Mass: 27670 Score: 193 Expect: 2.2e-015 Matches: 14** | | | | | | | | | | | | | | | | | | | | | | | | | | | | | | | | | | | | | | | | | | | | | | | | | | | | | | | | | | | | | | | | | | | | | | | | | | | | | | | | |  | | | | | Observed | | | | | | | Mr(expt) | | | | | | | | Mr(calc) | | | | | | | | ppm | | | | | | | | Start | | | | | | | | |  | | | | | | | End | | | | | | | | Miss | | | | | | | | Ions | | | | | | | Peptide | | | | | | | | | | | | | | | | 862.3210 | | | | | | | 861.3138 | | | | | | | | 861.4708 | | | | | | | | -182.33 | | | | | | | | 129 | | | | | | | | | - | | | | | | | 135 | | | | | | | | 0 | | | | | | | | --- | | | | | | | K.FELGQLR.F | | | | | | | | | | | | | | | | 862.3210 | | | | | | | 861.3138 | | | | | | | | 861.4708 | | | | | | | | -182.33 | | | | | | | | 129 | | | | | | | | | - | | | | | | | 135 | | | | | | | | 0 | | | | | | | | 18 | | | | | | | K.FELGQLR.F | | | | | | | | | | | | | | | | 910.2106 | | | | | | | 909.2034 | | | | | | | | 909.3498 | | | | | | | | -161.05 | | | | | | | | 141 | | | | | | | | | - | | | | | | | 147 | | | | | | | | 0 | | | | | | | | --- | | | | | | | K.TQDMEDR.L + Oxidation (M) | | | | | | | | | | | | | | | | 931.3787 | | | | | | | 930.3714 | | | | | | | | 930.4770 | | | | | | | | -113.46 | | | | | | | | 59 | | | | | | | | | - | | | | | | | 66 | | | | | | | | 1 | | | | | | | | --- | | | | | | | R.LEKDAEAR.E | | | | | | | | | | | | | | | | 1015.3171 | | | | | | | 1014.3098 | | | | | | | | 1014.4883 | | | | | | | | -175.91 | | | | | | | | 67 | | | | | | | | | - | | | | | | | 74 | | | | | | | | 0 | | | | | | | | --- | | | | | | | R.EAFEQHVR.D | | | | | | | | | | | | | | | | 1015.3171 | | | | | | | 1014.3098 | | | | | | | | 1014.4883 | | | | | | | | -175.91 | | | | | | | | 67 | | | | | | | | | - | | | | | | | 74 | | | | | | | | 0 | | | | | | | | 47 | | | | | | | R.EAFEQHVR.D | | | | | | | | | | | | | | | | 1056.4470 | | | | | | | 1055.4397 | | | | | | | | 1055.6226 | | | | | | | | -173.26 | | | | | | | | 148 | | | | | | | | | - | | | | | | | 156 | | | | | | | | 0 | | | | | | | | --- | | | | | | | R.LIELEALQK.A | | | | | | | | | | | | | | | | 1257.4884 | | | | | | | 1256.4811 | | | | | | | | 1256.6976 | | | | | | | | -172.25 | | | | | | | | 190 | | | | | | | | | - | | | | | | | 200 | | | | | | | | 0 | | | | | | | | --- | | | | | | | K.ATLLELVEQNK.I | | | | | | | | | | | | | | | | 1262.4412 | | | | | | | 1261.4339 | | | | | | | | 1261.6455 | | | | | | | | -167.70 | | | | | | | | 119 | | | | | | | | | - | | | | | | | 128 | | | | | | | | 0 | | | | | | | | --- | | | | | | | R.LNQEFFSHLK.F | | | | | | | | | | | | | | | | 1262.4412 | | | | | | | 1261.4339 | | | | | | | | 1261.6455 | | | | | | | | -167.70 | | | | | | | | 119 | | | | | | | | | - | | | | | | | 128 | | | | | | | | 0 | | | | | | | | 38 | | | | | | | R.LNQEFFSHLK.F | | | | | | | | | | | | | | | | 1642.6201 | | | | | | | 1641.6128 | | | | | | | | 1641.8937 | | | | | | | | -171.07 | | | | | | | | 204 | | | | | | | | | - | | | | | | | 218 | | | | | | | | 0 | | | | | | | | --- | | | | | | | R.SLLTLLDENIANAQK.S | | | | | | | | | | | | | | | | 2210.6838 | | | | | | | 2209.6765 | | | | | | | | 2210.0525 | | | | | | | | -170.11 | | | | | | | | 157 | | | | | | | | | - | | | | | | | 176 | | | | | | | | 0 | | | | | | | | --- | | | | | | | K.ALLEGTEAYDNMQADLITAR.K + Oxidation (M) | | | | | | | | | | | | | | | | 2210.6838 | | | | | | | 2209.6765 | | | | | | | | 2210.0525 | | | | | | | | -170.11 | | | | | | | | 157 | | | | | | | | | - | | | | | | | 176 | | | | | | | | 0 | | | | | | | | 60 | | | | | | | K.ALLEGTEAYDNMQADLITAR.K + Oxidation (M) | | | | | | | | | | | | | | | | 2220.7642 | | | | | | | 2219.7569 | | | | | | | | 2219.2307 | | | | | | | | 237 | | | | | | | | 1 | | | | | | | | | - | | | | | | | 22 | | | | | | | | 1 | | | | | | | | --- | | | | | | | -.MASLSLGLGIASTAITTVRTTR.R | | | | | | | | | | | | | | | | No match to: 700.2690, 705.2584, 709.2194, 713.2760, 716.2386, 726.2260, 730.2244, 734.3377, 738.2269, 744.2255, 748.2481, 750.3389, 751.2170, 758.2815, 768.3752, 779.2264, 785.2349, 797.2078, 802.2963, 815.2574, 823.2442, 834.3362, 842.3516, 848.3036, 850.3754, 856.3243, 860.9188, 868.3801, 876.8886, 882.3974, 906.3129, 921.3116, 922.2790, 935.3407, 951.3398, 960.3710, 982.3562, 997.3242, 999.3246, 1029.3208, 1034.3413, 1038.3315, 1051.5105, 1059.3949, 1059.3949, 1078.4060, 1085.4387, 1087.3268, 1105.4294, 1126.3622, 1139.4385, 1151.4854, 1160.3234, 1172.4786, 1179.3993, 1265.4414, 1315.4331, 1323.4133, 1326.4403, 1334.6643, 1361.4557, 1424.5002, 1448.6071, 1481.5468, 1507.4895, 1592.5260, 1592.5260, 1611.6201, 1619.5837, 1635.5396, 1650.6688, 1658.5524, 1704.5503, 1739.5773, 1739.5773, 1791.4873, 1893.5187, 1909.5955, 1921.6949, 1926.5887, 1927.5984, 1927.5984, 1940.5935, 1958.5039, 1999.7094, 1999.7094, 2002.7096, 2015.6943, 2072.6257, 2092.7058, 2146.7253, 2238.7490, 2238.7490, 2245.8032, 2423.6899, 3346.1165 | | | | | | | | | | | | | | | | | | | | | | | | | | | | | | | | | | | | | | | | | | | | | | | | | | | | | | | | | | | | | | | | | | | | | | | | | | | | | | | | | | |  | | | **S42** [**Cs8g19010.1**](http://zhangyang-pc/mascot/cgi/protein_view.pl?file=../data/20140118/F012134.dat&hit=1) **Mass: 55648 Score: 248 Expect: 7e-021 Matches: 19** | | | | | | | | | | | | | | | | | | | | | | | | | | | | | | | | | | | | | | | | | | | | | | | | | | | | | | | | | | | | | | | | | | | | | | | | | | | | | | | | | | |  | | | Observed | | | | | | Mr(expt) | | | | | | | Mr(calc) | | | | | | | | ppm | | | | | | | | Start | | | | | | | | |  | | | | | | | End | | | | | | | | Miss | | | | | | | | Ions | | | | | | | | Peptide | | | | | |  | | | | | | | | | | | 786.3153 | | | | | | 785.3080 | | | | | | | 785.4759 | | | | | | | | -213.76 | | | | | | | | 452 | | | | | | | | | - | | | | | | | 458 | | | | | | | | 0 | | | | | | | | --- | | | | | | | | K.VQIATVR.G | | | | | |  | | | | | | | | | | | 814.3594 | | | | | | 813.3522 | | | | | | | 813.4596 | | | | | | | | -132.05 | | | | | | | | 488 | | | | | | | | | - | | | | | | | 494 | | | | | | | | 1 | | | | | | | | --- | | | | | | | | K.EELPKAK.A | | | | | |  | | | | | | | | | | | 1060.3553 | | | | | | 1059.3480 | | | | | | | 1059.5349 | | | | | | | | -176.39 | | | | | | | | 326 | | | | | | | | | - | | | | | | | 335 | | | | | | | | 0 | | | | | | | | --- | | | | | | | | K.VFTDPSAPAR.R | | | | | |  | | | | | | | | | | | 1158.4131 | | | | | | 1157.4058 | | | | | | | 1157.6404 | | | | | | | | -202.66 | | | | | | | | 459 | | | | | | | | | - | | | | | | | 470 | | | | | | | | 1 | | | | | | | | --- | | | | | | | | R.GQAKAGTLPSTK.A | | | | | |  | | | | | | | | | | | 1199.4055 | | | | | | 1198.3982 | | | | | | | 1198.6306 | | | | | | | | -193.84 | | | | | | | | 497 | | | | | | | | | - | | | | | | | 506 | | | | | | | | 0 | | | | | | | | --- | | | | | | | | K.QINESKPEVR.K | | | | | |  | | | | | | | | | | | 1381.5026 | | | | | | 1380.4953 | | | | | | | 1380.7361 | | | | | | | | -174.38 | | | | | | | | 129 | | | | | | | | | - | | | | | | | 142 | | | | | | | | 0 | | | | | | | | --- | | | | | | | | R.AGVPELGAAQELAR.L | | | | | |  | | | | | | | | | | | 1381.5026 | | | | | | 1380.4953 | | | | | | | 1380.7361 | | | | | | | | -174.38 | | | | | | | | 129 | | | | | | | | | - | | | | | | | 142 | | | | | | | | 0 | | | | | | | | 102 | | | | | | | | R.AGVPELGAAQELAR.L | | | | | |  | | | | | | | | | | | 1413.4702 | | | | | | 1412.4629 | | | | | | | 1412.7664 | | | | | | | | -214.78 | | | | | | | | 247 | | | | | | | | | - | | | | | | | 258 | | | | | | | | 0 | | | | | | | | --- | | | | | | | | R.NQPLTVPEFLQK.V | | | | | |  | | | | | | | | | | | 1421.4396 | | | | | | 1420.4323 | | | | | | | 1420.6834 | | | | | | | | -176.74 | | | | | | | | 337 | | | | | | | | | - | | | | | | | 349 | | | | | | | | 0 | | | | | | | | --- | | | | | | | | R.VDELFSAIAEDGR.R | | | | | |  | | | | | | | | | | | 1577.5081 | | | | | | 1576.5008 | | | | | | | 1576.7845 | | | | | | | | -179.92 | | | | | | | | 337 | | | | | | | | | - | | | | | | | 350 | | | | | | | | 1 | | | | | | | | --- | | | | | | | | R.VDELFSAIAEDGRR.A | | | | | |  | | | | | | | | | | | 1592.5260 | | | | | | 1591.5187 | | | | | | | 1591.7955 | | | | | | | | -173.86 | | | | | | | | 99 | | | | | | | | | - | | | | | | | 115 | | | | | | | | 0 | | | | | | | | --- | | | | | | | | K.DSGTVFVAGATGQAGVR.I | | | | | |  | | | | | | | | | | | 1592.5260 | | | | | | 1591.5187 | | | | | | | 1591.7955 | | | | | | | | -173.86 | | | | | | | | 99 | | | | | | | | | - | | | | | | | 115 | | | | | | | | 0 | | | | | | | | 88 | | | | | | | | K.DSGTVFVAGATGQAGVR.I | | | | | |  | | | | | | | | | | | 1694.5007 | | | | | | 1693.4934 | | | | | | | 1693.8159 | | | | | | | | -190.36 | | | | | | | | 158 | | | | | | | | | - | | | | | | | 173 | | | | | | | | 0 | | | | | | | | --- | | | | | | | | R.LNAVESNFDSAESIAK.A | | | | | |  | | | | | | | | | | | 2014.6732 | | | | | | 2013.6659 | | | | | | | 2014.0847 | | | | | | | | -207.92 | | | | | | | | 129 | | | | | | | | | - | | | | | | | 148 | | | | | | | | 1 | | | | | | | | --- | | | | | | | | R.AGVPELGAAQELARLAASYK.I | | | | | |  | | | | | | | | | | | 2189.7190 | | | | | | 2188.7117 | | | | | | | 2189.1328 | | | | | | | | -192.37 | | | | | | | | 75 | | | | | | | | | - | | | | | | | 96 | | | | | | | | 0 | | | | | | | | --- | | | | | | | | R.DVTSLIPVVSSPGTGLSFGNSR.R | | | | | |  | | | | | | | | | | | 2305.6697 | | | | | | 2304.6624 | | | | | | | 2305.1550 | | | | | | | | -213.66 | | | | | | | | 158 | | | | | | | | | - | | | | | | | 180 | | | | | | | | 1 | | | | | | | | --- | | | | | | | | R.LNAVESNFDSAESIAKAIGNAGK.V | | | | | |  | | | | | | | | | | | 2330.5217 | | | | | | 2329.5144 | | | | | | | 2328.9326 | | | | | | | | 250 | | | | | | | | 46 | | | | | | | | | - | | | | | | | 67 | | | | | | | | 1 | | | | | | | | --- | | | | | | | | K.SKDENSSDESSNGDSGNSNPFR.F | | | | | |  | | | | | | | | | | | 2345.7363 | | | | | | 2344.7290 | | | | | | | 2345.2339 | | | | | | | | -215.30 | | | | | | | | 75 | | | | | | | | | - | | | | | | | 97 | | | | | | | | 1 | | | | | | | | --- | | | | | | | | R.DVTSLIPVVSSPGTGLSFGNSRR.K | | | | | |  | | | | | | | | | | | 2361.7317 | | | | | | 2360.7244 | | | | | | | 2361.2540 | | | | | | | | -224.26 | | | | | | | | 300 | | | | | | | | | - | | | | | | | 322 | | | | | | | | 1 | | | | | | | | --- | | | | | | | | K.VAKSQIASLVADVFSNTAVAENK.V | | | | | |  | | | | | | | | | | | No match to: 700.2494, 701.2867, 705.2043, 713.2728, 716.2343, 724.3461, 730.2495, 734.3295, 742.2321, 744.2477, 750.3605, 763.2627, 768.3605, 779.2560, 803.2867, 822.2788, 823.2465, 833.2324, 842.3424, 850.3569, 856.3349, 860.9062, 864.3427, 868.3637, 876.8790, 882.3698, 900.3484, 929.2868, 948.3165, 948.3165, 957.2624, 968.3416, 987.3528, 1047.3473, 1051.4756, 1059.3685, 1069.3112, 1131.3969, 1151.4572, 1165.4576, 1172.4128, 1178.3850, 1182.3988, 1187.4501, 1188.3979, 1195.3604, 1196.3682, 1196.3682, 1218.3684, 1226.4048, 1227.4705, 1227.4705, 1234.3561, 1250.3961, 1260.4016, 1261.4227, 1280.4163, 1334.5791, 1394.4530, 1395.5129, 1456.4683, 1465.5016, 1498.4999, 1541.4913, 1543.5239, 1562.5037, 1607.4645, 1618.5455, 1619.5356, 1623.4927, 1679.4772, 1702.6371, 1702.6371, 1706.5231, 1808.6116, 1830.7042, 1830.7042, 2078.5608, 2085.6277, 2085.6277, 2101.6580, 2101.6580, 2111.6565, 2123.6172, 2238.7183, 2245.7598, 2336.6702, 2381.6580, 2885.9392, 2885.9392, 2945.9106 | | | | | | | | | | | | | | | | | | | | | | | | | | | | | | | | | | | | | | | | | | | | | | | | | | | | | | | | | | | | | | | | | | | | | | | | | | | | | | | | | | |  | | | **S34** [**Cs4g11860.1**](http://zhangyang-pc/mascot/cgi/protein_view.pl?file=../data/20140118/F012113.dat&hit=1) **Mass: 22278 Score: 256 Expect: 1.1e-021 Matches: 10** | | | | | | | | | | | | | | | | | | | | | | | | | | | | | | | | | | | | | | | | | | | | | | | | | | | | | | | | | | | | | | | | | | | | | | | | | | | | | | | | | | |  | | | Observed | | | | | Mr(expt) | | | | | | | Mr(calc) | | | | | | | | ppm | | | | | | | | Start | | | | | | | | |  | | | | | | | End | | | | | | | | Miss | | | | | | | | Ions | | | | | | | | Peptide | | | | | | | | | | | | | | | | | | 830.2763 | | | | | 829.2690 | | | | | | | 829.4909 | | | | | | | | -267.44 | | | | | | | | 23 | | | | | | | | | - | | | | | | | 29 | | | | | | | | 1 | | | | | | | | --- | | | | | | | | K.LAEEIKK.G | | | | | | | | | | | | | | | | | | 1062.2957 | | | | | 1061.2884 | | | | | | | 1061.4674 | | | | | | | | -168.63 | | | | | | | | 86 | | | | | | | | | - | | | | | | | 94 | | | | | | | | 0 | | | | | | | | --- | | | | | | | | R.FGMMAAQFK.A + 2 Oxidation (M) | | | | | | | | | | | | | | | | | | 1456.4016 | | | | | 1455.3943 | | | | | | | 1455.6379 | | | | | | | | -167.33 | | | | | | | | 164 | | | | | | | | | - | | | | | | | 179 | | | | | | | | 0 | | | | | | | | --- | | | | | | | | K.GGSPYGAGTFAGDGSR.Q | | | | | | | | | | | | | | | | | | 1456.4016 | | | | | 1455.3943 | | | | | | | 1455.6379 | | | | | | | | -167.33 | | | | | | | | 164 | | | | | | | | | - | | | | | | | 179 | | | | | | | | 0 | | | | | | | | 136 | | | | | | | | K.GGSPYGAGTFAGDGSR.Q | | | | | | | | | | | | | | | | | | 1737.5435 | | | | | 1736.5362 | | | | | | | 1736.9349 | | | | | | | | -229.51 | | | | | | | | 41 | | | | | | | | | - | | | | | | | 55 | | | | | | | | 0 | | | | | | | | --- | | | | | | | | K.LWQVPETLPEEVLGK.M | | | | | | | | | | | | | | | | | | 1737.5435 | | | | | 1736.5362 | | | | | | | 1736.9349 | | | | | | | | -229.51 | | | | | | | | 41 | | | | | | | | | - | | | | | | | 55 | | | | | | | | 0 | | | | | | | | --- | | | | | | | | K.LWQVPETLPEEVLGK.M | | | | | | | | | | | | | | | | | | 1754.5671 | | | | | 1753.5598 | | | | | | | 1753.8635 | | | | | | | | -173.14 | | | | | | | | 180 | | | | | | | | | - | | | | | | | 194 | | | | | | | | 0 | | | | | | | | --- | | | | | | | | R.QPTELELEQAFHQGK.H | | | | | | | | | | | | | | | | | | 2365.8008 | | | | | 2364.7935 | | | | | | | 2364.2399 | | | | | | | | 234 | | | | | | | | 41 | | | | | | | | | - | | | | | | | 61 | | | | | | | | 1 | | | | | | | | --- | | | | | | | | K.LWQVPETLPEEVLGKMSAPPK.S + Oxidation (M) | | | | | | | | | | | | | | | | | | 2591.8555 | | | | | 2590.8482 | | | | | | | 2591.2908 | | | | | | | | -170.79 | | | | | | | | 62 | | | | | | | | | - | | | | | | | 85 | | | | | | | | 0 | | | | | | | | --- | | | | | | | | K.SDVPIITPNELAEADGFVFGFPTR.F | | | | | | | | | | | | | | | | | | 2591.8555 | | | | | 2590.8482 | | | | | | | 2591.2908 | | | | | | | | -170.79 | | | | | | | | 62 | | | | | | | | | - | | | | | | | 85 | | | | | | | | 0 | | | | | | | | 92 | | | | | | | | K.SDVPIITPNELAEADGFVFGFPTR.F | | | | | | | | | | | | | | | | | | No match to: 700.2160, 701.1688, 712.1407, 713.2836, 727.2098, 733.0792, 749.2910, 797.2147, 823.2448, 842.3527, 848.2548, 850.2628, 855.2717, 860.9194, 877.2819, 914.2407, 914.2407, 928.2852, 930.2606, 947.3166, 978.3592, 995.2819, 998.3053, 1022.3409, 1035.3114, 1038.4935, 1059.3611, 1104.4045, 1116.3531, 1129.3387, 1170.4453, 1176.4041, 1178.4075, 1179.3949, 1187.4609, 1187.4609, 1204.4139, 1207.3849, 1209.4341, 1210.4177, 1210.4177, 1220.4017, 1221.4022, 1222.4055, 1222.4055, 1232.3947, 1235.4038, 1238.4064, 1244.4022, 1252.4147, 1261.4941, 1261.4941, 1277.4939, 1283.4769, 1289.4460, 1295.4930, 1307.4435, 1315.4012, 1372.4557, 1375.4468, 1380.4713, 1392.4601, 1414.4744, 1478.3811, 1480.4739, 1485.5159, 1497.5480, 1502.5142, 1516.5131, 1519.5350, 1551.5795, 1637.5510, 1655.5378, 1655.5378, 1660.6129, 1682.5958, 1716.5728, 1736.5962, 1741.5992, 1751.5663, 1753.6057, 1759.5415, 1766.5297, 1775.5712, 1789.5333, 1791.5432, 1804.5313, 1854.5675, 1880.6240, 1880.6240, 1918.6116, 1921.7159, 1934.6110, 1993.6471, 2007.5977, 2055.6582, 2072.6477, 2238.7698, 2324.7878, 2704.7117 | | | | | | | | | | | | | | | | | | | | | | | | | | | | | | | | | | | | | | | | | | | | | | | | | | | | | | | | | | | | | | | | | | | | | | | | | | | | | | | | | | |  | | | **S49** [**orange1.1t00001.2**](http://zhangyang-pc/mascot/cgi/protein_view.pl?file=../data/20140118/F012138.dat&hit=1) **Mass: 67301 Score: 247 Expect: 8.8e-021 Matches: 20** | | | | | | | | | | | | | | | | | | | | | | | | | | | | | | | | | | | | | | | | | | | | | | | | | | | | | | | | | | | | | | | | | | | | | | | | | | | | | | | | | | |  | | | Observed | | | | | | | Mr(expt) | | | | | | | | Mr(calc) | | | | | | | | ppm | | | | | | | | Start | | | | | | | | |  | | | | | | | End | | | | | | | | Miss | | | | | | | | Ions | | | | | | | Peptide | | | | | | | | | | | | | | | | 700.2354 | | | | | | | 699.2281 | | | | | | | | 699.3235 | | | | | | | | -136.39 | | | | | | | | 111 | | | | | | | | | - | | | | | | | 115 | | | | | | | | 1 | | | | | | | | --- | | | | | | | R.RECHR.T | | | | | | | | | | | | | | | | 748.2547 | | | | | | | 747.2474 | | | | | | | | 747.3875 | | | | | | | | -187.42 | | | | | | | | 279 | | | | | | | | | - | | | | | | | 286 | | | | | | | | 0 | | | | | | | | --- | | | | | | | K.LSSASAGR.E | | | | | | | | | | | | | | | | 773.3325 | | | | | | | 772.3252 | | | | | | | | 772.4807 | | | | | | | | -201.30 | | | | | | | | 342 | | | | | | | | | - | | | | | | | 348 | | | | | | | | 0 | | | | | | | | --- | | | | | | | K.LGLLTTR.Q | | | | | | | | | | | | | | | | 889.2952 | | | | | | | 888.2879 | | | | | | | | 888.4851 | | | | | | | | -221.98 | | | | | | | | 515 | | | | | | | | | - | | | | | | | 522 | | | | | | | | 0 | | | | | | | | --- | | | | | | | K.TVAQVCLR.W | | | | | | | | | | | | | | | | 965.3505 | | | | | | | 964.3432 | | | | | | | | 964.5229 | | | | | | | | -186.31 | | | | | | | | 349 | | | | | | | | | - | | | | | | | 356 | | | | | | | | 0 | | | | | | | | --- | | | | | | | R.QDLFITTK.L | | | | | | | | | | | | | | | | 1005.3713 | | | | | | | 1004.3640 | | | | | | | | 1004.5515 | | | | | | | | -186.67 | | | | | | | | 560 | | | | | | | | | - | | | | | | | 567 | | | | | | | | 0 | | | | | | | | --- | | | | | | | K.INQIPQHR.M | | | | | | | | | | | | | | | | 1005.3713 | | | | | | | 1004.3640 | | | | | | | | 1004.5515 | | | | | | | | -186.67 | | | | | | | | 560 | | | | | | | | | - | | | | | | | 567 | | | | | | | | 0 | | | | | | | | 52 | | | | | | | K.INQIPQHR.M | | | | | | | | | | | | | | | | 1085.3690 | | | | | | | 1084.3617 | | | | | | | | 1084.5553 | | | | | | | | -178.50 | | | | | | | | 432 | | | | | | | | | - | | | | | | | 441 | | | | | | | | 0 | | | | | | | | --- | | | | | | | K.FIGVSNFSSK.K | | | | | | | | | | | | | | | | 1091.3671 | | | | | | | 1090.3598 | | | | | | | | 1090.5546 | | | | | | | | -178.63 | | | | | | | | 407 | | | | | | | | | - | | | | | | | 415 | | | | | | | | 0 | | | | | | | | --- | | | | | | | K.EDLVPLDYK.G | | | | | | | | | | | | | | | | 1129.3690 | | | | | | | 1128.3617 | | | | | | | | 1128.6026 | | | | | | | | -213.44 | | | | | | | | 331 | | | | | | | | | - | | | | | | | 341 | | | | | | | | 0 | | | | | | | | --- | | | | | | | R.ALGEAIDEALK.L | | | | | | | | | | | | | | | | 1315.4309 | | | | | | | 1314.4236 | | | | | | | | 1314.6608 | | | | | | | | -180.43 | | | | | | | | 572 | | | | | | | | | - | | | | | | | 582 | | | | | | | | 0 | | | | | | | | --- | | | | | | | R.DEYIIPHGPFK.T | | | | | | | | | | | | | | | | 1315.4309 | | | | | | | 1314.4236 | | | | | | | | 1314.6608 | | | | | | | | -180.43 | | | | | | | | 572 | | | | | | | | | - | | | | | | | 582 | | | | | | | | 0 | | | | | | | | 60 | | | | | | | R.DEYIIPHGPFK.T | | | | | | | | | | | | | | | | 1337.3972 | | | | | | | 1336.3899 | | | | | | | | 1336.5540 | | | | | | | | -122.75 | | | | | | | | 416 | | | | | | | | | - | | | | | | | 426 | | | | | | | | 0 | | | | | | | | --- | | | | | | | K.GVWEAMEECQR.L | | | | | | | | | | | | | | | | 1345.4384 | | | | | | | 1344.4311 | | | | | | | | 1344.6959 | | | | | | | | -196.91 | | | | | | | | 287 | | | | | | | | | - | | | | | | | 299 | | | | | | | | 0 | | | | | | | | --- | | | | | | | R.EMPVIGLGTAVDK.K + Oxidation (M) | | | | | | | | | | | | | | | | 1353.3947 | | | | | | | 1352.3874 | | | | | | | | 1352.5489 | | | | | | | | -119.39 | | | | | | | | 416 | | | | | | | | | - | | | | | | | 426 | | | | | | | | 0 | | | | | | | | --- | | | | | | | K.GVWEAMEECQR.L + Oxidation (M) | | | | | | | | | | | | | | | | 1412.3505 | | | | | | | 1411.3432 | | | | | | | | 1411.6004 | | | | | | | | -182.20 | | | | | | | | 319 | | | | | | | | | - | | | | | | | 330 | | | | | | | | 0 | | | | | | | | --- | | | | | | | R.HFDTASEYGTER.A | | | | | | | | | | | | | | | | 1412.3505 | | | | | | | 1411.3432 | | | | | | | | 1411.6004 | | | | | | | | -182.20 | | | | | | | | 319 | | | | | | | | | - | | | | | | | 330 | | | | | | | | 0 | | | | | | | | 100 | | | | | | | R.HFDTASEYGTER.A | | | | | | | | | | | | | | | | 1480.5931 | | | | | | | 1479.5858 | | | | | | | | 1479.7318 | | | | | | | | -98.64 | | | | | | | | 210 | | | | | | | | | - | | | | | | | 223 | | | | | | | | 0 | | | | | | | | --- | | | | | | | K.NGAVDLSTGTVYQR.Y | | | | | | | | | | | | | | | | 1802.5840 | | | | | | | 1801.5767 | | | | | | | | 1802.0090 | | | | | | | | -239.90 | | | | | | | | 523 | | | | | | | | | - | | | | | | | 538 | | | | | | | | 1 | | | | | | | | --- | | | | | | | R.WIIEQGAIVVAKSFNK.E | | | | | | | | | | | | | | | | 1802.5840 | | | | | | | 1801.5767 | | | | | | | | 1802.0090 | | | | | | | | -239.90 | | | | | | | | 523 | | | | | | | | | - | | | | | | | 538 | | | | | | | | 1 | | | | | | | | --- | | | | | | | R.WIIEQGAIVVAKSFNK.E | | | | | | | | | | | | | | | | No match to: 713.2734, 716.2236, 734.3295, 750.3132, 757.2400, 768.3784, 842.3493, 850.3464, 851.3078, 855.3461, 868.3796, 882.3934, 904.3096, 946.3305, 948.3317, 962.3091, 967.2835, 988.3540, 1019.4293, 1021.3547, 1028.3419, 1028.3419, 1037.3549, 1039.3329, 1040.3357, 1040.3357, 1042.3506, 1053.3542, 1054.3518, 1056.3481, 1059.3572, 1070.3521, 1072.3059, 1121.3615, 1128.3455, 1128.3455, 1133.4570, 1151.4315, 1159.3942, 1164.3701, 1165.3903, 1171.4187, 1171.4187, 1175.3911, 1266.4117, 1269.4622, 1271.4054, 1303.4054, 1323.3629, 1330.5040, 1335.3490, 1342.5236, 1349.4290, 1351.3624, 1365.4191, 1387.4631, 1394.3936, 1400.4507, 1410.3649, 1411.3927, 1416.3787, 1418.3840, 1424.3650, 1426.3636, 1428.3789, 1429.4008, 1430.3309, 1434.4200, 1439.4301, 1442.2881, 1449.5219, 1449.5219, 1465.4962, 1606.5308, 1606.5308, 1738.6119, 1753.5504, 1754.5750, 1816.4802, 1818.5538, 1834.5510, 1840.5474, 2125.5425, 2201.6846, 2213.6704, 2229.6733, 2237.6997, 2238.7156, 2245.7380, 2366.6726 | | | | | | | | | | | | | | | | | | | | | | | | | | | | | | | | | | | | | | | | | | | | | | | | | | | | | | | | | | | | | | | | | | | | | | | | | | | | | | | | | | |  | | | **Unidentified protein spots** | | | | | | | | | | | | | | | | | | | | | | | | | | | | | | | | | | | | | | | | | | | | | | | | | | | | | | | | | | | | | | | | | | | | | | | | | | | | | | | | | | |  | | | **S33** [**orange1.1t05474.1**](http://zhangyang-pc/mascot/cgi/protein_view.pl?file=../data/20140118/F012099.dat&hit=1) **Mass: 16188 Score: 100 Expect: 4.4e-006 Matches: 4** | | | | | | | | | | | | | | | | | | | | | | | | | | | | | | | | | | | | | | | | | | | | | | | | | | | | | | | | | | | | | | | | | | | | | | | | | | | | | | | | | | |  | | | Observed | | | | | | | | Mr(expt) | | | | | | | | Mr(calc) | | | | | | | | ppm | | | | | | | | Start | | | | | | | | |  | | | | | | | End | | | | | | | | Miss | | | | | | | | Ions | | | | | | | Peptide | | | | | | | | | | | | | | | 1717.5122 | | | | | | | | 1716.5049 | | | | | | | | 1716.7744 | | | | | | | | -156.95 | | | | | | | | 54 | | | | | | | | | - | | | | | | | 69 | | | | | | | | 0 | | | | | | | | --- | | | | | | | K.EDFGGGHPDPNLTYAK.E | | | | | | | | | | | | | | | 2062.5457 | | | | | | | | 2061.5384 | | | | | | | | 2061.8512 | | | | | | | | -151.68 | | | | | | | | 80 | | | | | | | | | - | | | | | | | 99 | | | | | | | | 0 | | | | | | | | --- | | | | | | | K.SNTQDEPPEFGAAADGDADR.N | | | | | | | | | | | | | | | 2062.5457 | | | | | | | | 2061.5384 | | | | | | | | 2061.8512 | | | | | | | | -151.68 | | | | | | | | 80 | | | | | | | | | - | | | | | | | 99 | | | | | | | | 0 | | | | | | | | 91 | | | | | | | K.SNTQDEPPEFGAAADGDADR.N | | | | | | | | | | | | | | | 2286.7610 | | | | | | | | 2285.7537 | | | | | | | | 2285.1077 | | | | | | | | 283 | | | | | | | | 54 | | | | | | | | | - | | | | | | | 74 | | | | | | | | 1 | | | | | | | | --- | | | | | | | K.EDFGGGHPDPNLTYAKELVAR.M | | | | | | | | | | | | | | | No match to: 700.2706, 707.2587, 712.2003, 713.2952, 715.2586, 716.2549, 718.2430, 724.3601, 730.2466, 732.2208, 734.3301, 736.3039, 738.2301, 739.2308, 742.2588, 750.3540, 752.2744, 757.2701, 768.3979, 805.2564, 830.2831, 834.3554, 842.3691, 848.3343, 850.3944, 850.3944, 860.9332, 860.9332, 864.3468, 868.4036, 876.9055, 882.3740, 884.2613, 884.2613, 901.2990, 904.2778, 906.2793, 913.2704, 926.2950, 928.2787, 931.2861, 932.3112, 935.3071, 938.3024, 948.3705, 949.3268, 952.3404, 968.3388, 973.3536, 982.3850, 986.3065, 986.3065, 992.3482, 996.4033, 1000.3216, 1007.4761, 1012.3192, 1017.4294, 1031.3861, 1031.3861, 1043.4502, 1043.4502, 1046.3550, 1051.5286, 1057.3632, 1059.3948, 1077.0389, 1085.3771, 1107.3722, 1114.3818, 1119.4360, 1122.4109, 1126.3866, 1126.3866, 1139.4363, 1139.4363, 1143.4086, 1147.4639, 1148.4069, 1148.4069, 1151.4973, 1153.4417, 1161.4054, 1165.5122, 1168.3860, 1179.3834, 1193.3608, 1261.5297, 1267.4958, 1275.4238, 1276.4943, 1290.4783, 1302.4515, 1315.4399, 1334.6439, 1465.5441, 1505.5013, 1512.5178, 1547.4818, 1626.6062, 1685.6266, 1720.5830, 1839.6479, 2076.5574, 2111.7012, 2238.7925 | | | | | | | | | | | | | | | | | | | | | | | | | | | | | | | | | | | | | | | | | | | | | | | | | | | | | | | | | | | | | | | | | | | | | | | | | | | | | | | | | |  | | | | **S25** [**Cs7g11110.1**](http://zhangyang-pc/mascot/cgi/protein_view.pl?file=../data/20140118/F012124.dat&hit=1) **Mass: 66908 Score: 65 Expect: 0.015 Matches: 21** | | | | | | | | | | | | | | | | | | | | | | | | | | | | | | | | | | | | | | | | | | | | | | | | | | | | | | | | | | | | | | | | | | | | | | | | | | | | | | | | | |  | | | | Observed | | | | | | | | Mr(expt) | | | | | | | | Mr(calc) | | | | | | | | ppm | | | | | | | | Start | | | | | | | | |  | | | | | | | End | | | | | | | | Miss | | | | | | | | Ions | | | | | | | Peptide | | | | | | | | | | | | | | | 949.2776 | | | | | | | | 948.2704 | | | | | | | | 948.5062 | | | | | | | | -248.67 | | | | | | | | 160 | | | | | | | | | - | | | | | | | 167 | | | | | | | | 1 | | | | | | | | --- | | | | | | | R.TLTCRIDK.R | | | | | | | | | | | | | | | 949.2776 | | | | | | | | 948.2704 | | | | | | | | 948.5062 | | | | | | | | -248.67 | | | | | | | | 160 | | | | | | | | | - | | | | | | | 167 | | | | | | | | 1 | | | | | | | | --- | | | | | | | R.TLTCRIDK.R | | | | | | | | | | | | | | | 1111.4218 | | | | | | | | 1110.4145 | | | | | | | | 1110.5611 | | | | | | | | -131.95 | | | | | | | | 137 | | | | | | | | | - | | | | | | | 145 | | | | | | | | 0 | | | | | | | | --- | | | | | | | K.HFTIFGYAR.S | | | | | | | | | | | | | | | 1111.4218 | | | | | | | | 1110.4145 | | | | | | | | 1110.5611 | | | | | | | | -131.95 | | | | | | | | 137 | | | | | | | | | - | | | | | | | 145 | | | | | | | | 0 | | | | | | | | --- | | | | | | | K.HFTIFGYAR.S | | | | | | | | | | | | | | | 1127.4158 | | | | | | | | 1126.4085 | | | | | | | | 1126.6757 | | | | | | | | -237.13 | | | | | | | | 368 | | | | | | | | | - | | | | | | | 376 | | | | | | | | 1 | | | | | | | | --- | | | | | | | K.VLRSMRPLR.L | | | | | | | | | | | | | | | 1128.4458 | | | | | | | | 1127.4385 | | | | | | | | 1127.6186 | | | | | | | | -159.70 | | | | | | | | 521 | | | | | | | | | - | | | | | | | 530 | | | | | | | | 0 | | | | | | | | --- | | | | | | | R.LLLDAIEGER.R | | | | | | | | | | | | | | | 1128.4458 | | | | | | | | 1127.4385 | | | | | | | | 1127.6186 | | | | | | | | -159.70 | | | | | | | | 521 | | | | | | | | | - | | | | | | | 530 | | | | | | | | 0 | | | | | | | | 16 | | | | | | | R.LLLDAIEGER.R | | | | | | | | | | | | | | | 1151.4576 | | | | | | | | 1150.4503 | | | | | | | | 1150.5870 | | | | | | | | -118.79 | | | | | | | | 377 | | | | | | | | | - | | | | | | | 386 | | | | | | | | 0 | | | | | | | | --- | | | | | | | R.LEDVVTGQYK.S | | | | | | | | | | | | | | | 1284.5259 | | | | | | | | 1283.5186 | | | | | | | | 1283.7197 | | | | | | | | -156.64 | | | | | | | | 521 | | | | | | | | | - | | | | | | | 531 | | | | | | | | 1 | | | | | | | | --- | | | | | | | R.LLLDAIEGERR.L | | | | | | | | | | | | | | | 1284.5259 | | | | | | | | 1283.5186 | | | | | | | | 1283.7197 | | | | | | | | -156.64 | | | | | | | | 521 | | | | | | | | | - | | | | | | | 531 | | | | | | | | 1 | | | | | | | | 23 | | | | | | | R.LLLDAIEGERR.L | | | | | | | | | | | | | | | 1454.5795 | | | | | | | | 1453.5722 | | | | | | | | 1453.6653 | | | | | | | | -64.05 | | | | | | | | 148 | | | | | | | | | - | | | | | | | 159 | | | | | | | | 1 | | | | | | | | --- | | | | | | | K.MTDAELRNMVSR.T + 2 Oxidation (M) | | | | | | | | | | | | | | | 1500.5045 | | | | | | | | 1499.4972 | | | | | | | | 1499.6272 | | | | | | | | -86.67 | | | | | | | | 169 | | | | | | | | | - | | | | | | | 180 | | | | | | | | 1 | | | | | | | | --- | | | | | | | R.ENCDEKMDEFLK.R | | | | | | | | | | | | | | | 1889.5586 | | | | | | | | 1888.5513 | | | | | | | | 1888.0175 | | | | | | | | 283 | | | | | | | | 121 | | | | | | | | | - | | | | | | | 136 | | | | | | | | 0 | | | | | | | | --- | | | | | | | K.IFPALFALYYEGFLPK.H | | | | | | | | | | | | | | | 1892.5659 | | | | | | | | 1891.5586 | | | | | | | | 1890.9986 | | | | | | | | 296 | | | | | | | | 371 | | | | | | | | | - | | | | | | | 386 | | | | | | | | 1 | | | | | | | | --- | | | | | | | R.SMRPLRLEDVVTGQYK.S | | | | | | | | | | | | | | | 1893.5514 | | | | | | | | 1892.5441 | | | | | | | | 1892.9632 | | | | | | | | -221.39 | | | | | | | | 407 | | | | | | | | | - | | | | | | | 424 | | | | | | | | 0 | | | | | | | | --- | | | | | | | K.DSLTPTFAAAALFIDNAR.W | | | | | | | | | | | | | | | 1893.5514 | | | | | | | | 1892.5441 | | | | | | | | 1892.9632 | | | | | | | | -221.39 | | | | | | | | 407 | | | | | | | | | - | | | | | | | 424 | | | | | | | | 0 | | | | | | | | --- | | | | | | | K.DSLTPTFAAAALFIDNAR.W | | | | | | | | | | | | | | | 2309.7058 | | | | | | | | 2308.6985 | | | | | | | | 2308.2063 | | | | | | | | 213 | | | | | | | | 403 | | | | | | | | | - | | | | | | | 424 | | | | | | | | 1 | | | | | | | | --- | | | | | | | K.TVSKDSLTPTFAAAALFIDNAR.W | | | | | | | | | | | | | | | 2334.7827 | | | | | | | | 2333.7754 | | | | | | | | 2333.2896 | | | | | | | | 208 | | | | | | | | 210 | | | | | | | | | - | | | | | | | 229 | | | | | | | | 1 | | | | | | | | --- | | | | | | | R.VSNRLFYLSIPPNIFIDAVR.C | | | | | | | | | | | | | | | 2349.7317 | | | | | | | | 2348.7244 | | | | | | | | 2348.1253 | | | | | | | | 255 | | | | | | | | 48 | | | | | | | | | - | | | | | | | 69 | | | | | | | | 0 | | | | | | | | --- | | | | | | | R.NSHPNMVLMEDGAAVTTPTHVK.N | | | | | | | | | | | | | | | 2365.7144 | | | | | | | | 2364.7071 | | | | | | | | 2364.1202 | | | | | | | | 248 | | | | | | | | 48 | | | | | | | | | - | | | | | | | 69 | | | | | | | | 0 | | | | | | | | --- | | | | | | | R.NSHPNMVLMEDGAAVTTPTHVK.N + Oxidation (M) | | | | | | | | | | | | | | | 2381.6550 | | | | | | | | 2380.6477 | | | | | | | | 2380.1151 | | | | | | | | 224 | | | | | | | | 48 | | | | | | | | | - | | | | | | | 69 | | | | | | | | 0 | | | | | | | | --- | | | | | | | R.NSHPNMVLMEDGAAVTTPTHVK.N + 2 Oxidation (M) | | | | | | | | | | | | | | | No match to: 713.2859, 768.3652, 838.2671, 842.3570, 850.3370, 868.3843, 876.8864, 881.3398, 948.2418, 960.2825, 961.3143, 961.3143, 979.3434, 991.3334, 1007.3358, 1020.3256, 1033.3127, 1101.4430, 1106.3932, 1109.4148, 1125.4152, 1133.3997, 1155.3832, 1165.4597, 1179.4133, 1199.3889, 1202.3870, 1207.4320, 1209.4974, 1209.4974, 1223.4534, 1257.4897, 1264.4457, 1266.4662, 1267.4972, 1283.4928, 1298.4816, 1300.4854, 1306.5074, 1316.4507, 1332.4663, 1338.4635, 1433.5389, 1440.5869, 1446.6049, 1446.6049, 1456.5242, 1468.5691, 1625.5962, 1641.5537, 1642.5806, 1642.5806, 1656.5873, 1664.5844, 1680.5630, 1808.5751, 1810.6447, 1824.6088, 1829.5784, 1845.5349, 1857.5636, 1873.5239, 1904.5483, 1905.5403, 1909.5433, 1914.5931, 1921.5275, 1925.5488, 1937.5441, 1940.5585, 1959.5800, 2039.8419, 2159.6733, 2238.7688, 2311.8613, 2324.7422, 2325.7612, 2327.7300, 2327.7300, 2329.2451, 2338.6841, 2341.7634, 2344.7529, 2423.6846, 2435.6128, 2559.8450, 2629.6384, 3081.1829, 3082.2083 | | | | | | | | | | | | | | | | | | | | | | | | | | | | | | | | | | | | | | | | | | | | | | | | | | | | | | | | | | | | | | | | | | | | | | | | | | | | | | | | | | | |  | |  | **S53** [**Cs9g05910.3**](http://zhangyang-pc/mascot/cgi/protein_view.pl?file=../data/20140118/F012163.dat&hit=1) **Mass: 27982 Score: 43 Expect: 2.3 Matches: 12** | | | | | | | | | | | | | | | | | | | | | | | | | | | | | | | | | | | | | | | | | | | | | | | | | | | | | | | | | | | | | | | | | | | | | | |  | | | | | | | | | | | | | | Observed | | Mr(expt) | | | | | | | Mr(calc) | | | | | | | | ppm | | | | | | | | Start | | | | | | | |  | | | End | | | | | | | | | | | | | Miss | | | | | | | | Ions | | | | | | | | Peptide | | | | | | | | | | | | | | | | | | | | | 705.2510 | | 704.2438 | | | | | | | 704.3606 | | | | | | | | -165.83 | | | | | | | | 108 | | | | | | | | - | | | 112 | | | | | | | | | | | | | 1 | | | | | | | | --- | | | | | | | | K.DTWRK.N | | | | | | | | | | | | | | | | | | | | | 1565.4061 | | 1564.3988 | | | | | | | 1564.7820 | | | | | | | | -244.90 | | | | | | | | 132 | | | | | | | | - | | | 148 | | | | | | | | | | | | | 1 | | | | | | | | --- | | | | | | | | K.AKGVSAVGAAGFCWGGK.V | | | | | | | | | | | | | | | | | | | | | 1763.4877 | | 1762.4804 | | | | | | | 1762.9189 | | | | | | | | -248.71 | | | | | | | | 134 | | | | | | | | - | | | 152 | | | | | | | | | | | | | 1 | | | | | | | | --- | | | | | | | | K.GVSAVGAAGFCWGGKVAVK.L | | | | | | | | | | | | | | | | | | | | | 1887.5845 | | 1886.5772 | | | | | | | 1886.0302 | | | | | | | | 290 | | | | | | | | 201 | | | | | | | | - | | | 216 | | | | | | | | | | | | | 1 | | | | | | | | --- | | | | | | | | R.FDEILSAKPKFDHLVK.T | | | | | | | | | | | | | | | | | | | | | 1893.5737 | | 1892.5664 | | | | | | | 1892.0692 | | | | | | | | 263 | | | | | | | | 2 | | | | | | | | - | | | 18 | | | | | | | | | | | | | 0 | | | | | | | | --- | | | | | | | | M.MELILLTSLLLNFASSK.A | | | | | | | | | | | | | | | | | | | | | 1951.5852 | | 1950.5779 | | | | | | | 1950.9761 | | | | | | | | -204.08 | | | | | | | | 58 | | | | | | | | - | | | 74 | | | | | | | | | | | | | 0 | | | | | | | | --- | | | | | | | | K.AVLMISDIYGDEPPIYR.S | | | | | | | | | | | | | | | | | | | | | 1967.5872 | | 1966.5799 | | | | | | | 1966.9710 | | | | | | | | -198.82 | | | | | | | | 58 | | | | | | | | - | | | 74 | | | | | | | | | | | | | 0 | | | | | | | | --- | | | | | | | | K.AVLMISDIYGDEPPIYR.S + Oxidation (M) | | | | | | | | | | | | | | | | | | | | | 2073.6038 | | 2072.5965 | | | | | | | 2072.9911 | | | | | | | | -190.35 | | | | | | | | 26 | | | | | | | | - | | | 46 | | | | | | | | | | | | | 0 | | | | | | | | --- | | | | | | | | R.EPPPFCPTCGAGTVTELGGLK.A | | | | | | | | | | | | | | | | | | | | | 2075.5811 | | 2074.5738 | | | | | | | 2075.1197 | | | | | | | | -263.07 | | | | | | | | 180 | | | | | | | | - | | | 199 | | | | | | | | | | | | | 1 | | | | | | | | --- | | | | | | | | K.VPIAVLGAERDNGLPPAQMK.R | | | | | | | | | | | | | | | | | | | | | 2075.5811 | | 2074.5738 | | | | | | | 2075.1197 | | | | | | | | -263.07 | | | | | | | | 180 | | | | | | | | - | | | 199 | | | | | | | | | | | | | 1 | | | | | | | | --- | | | | | | | | K.VPIAVLGAERDNGLPPAQMK.R | | | | | | | | | | | | | | | | | | | | | 2114.5247 | | 2113.5174 | | | | | | | 2114.0520 | | | | | | | | -252.88 | | | | | | | | 211 | | | | | | | | - | | | 228 | | | | | | | | | | | | | 1 | | | | | | | | --- | | | | | | | | K.FDHLVKTYPGVCHGWTVR.Y | | | | | | | | | | | | | | | | | | | | | 3111.8979 | | 3110.8906 | | | | | | | 3110.5206 | | | | | | | | 119 | | | | | | | | 26 | | | | | | | | - | | | 56 | | | | | | | | | | | | | 1 | | | | | | | | --- | | | | | | | | R.EPPPFCPTCGAGTVTELGGLKAYVTGPPHSK.K | | | | | | | | | | | | | | | | | | | | | No match to: 734.3478, 750.3045, 842.3407, 944.3690, 964.3427, 970.3479, 987.3018, 987.3018, 998.3070, 999.3030, 999.3030, 1009.2723, 1014.3572, 1015.3209, 1018.3046, 1037.2922, 1053.3275, 1055.3342, 1055.3342, 1067.3502, 1071.3203, 1072.3575, 1116.3219, 1163.3477, 1163.3477, 1178.3318, 1260.4474, 1263.3696, 1320.4191, 1337.4387, 1380.3853, 1394.3822, 1397.3947, 1398.3757, 1398.3757, 1406.3524, 1408.3652, 1409.3655, 1410.3728, 1410.3728, 1422.3879, 1424.3815, 1426.3679, 1426.3679, 1436.4348, 1440.3666, 1444.3702, 1449.3804, 1465.4160, 1468.4155, 1477.4340, 1478.3899, 1481.4105, 1506.4022, 1508.3694, 1520.3503, 1531.5149, 1534.3966, 1567.3927, 1592.5192, 1607.6438, 1619.5363, 1633.5175, 1830.5116, 1832.6348, 1832.6348, 1842.6549, 1844.6357, 1845.6387, 1846.6497, 1847.2440, 1849.1807, 1858.6837, 1859.5753, 1862.6213, 1874.6478, 1875.5455, 1879.5206, 1881.6615, 1921.6642, 1936.6123, 1939.5834, 1939.5834, 1948.6672, 1955.5674, 1962.5730, 1971.5958, 1993.5938, 2097.5588, 2158.6594, 2297.6829, 2309.6560, 2357.7139, 3122.8879, 3123.9072, 3139.8718, 3377.8147, 3528.0071 | | | | | | | | | | | | | | | | | | | | | | | | | | | | | | | | | | | | | | | | | | | | | | | | | | | | | | | | | | | | | | | | | | | | | | | | | | | | | | | | | |  | | | | **S54** [**Cs9g05100.7**](http://zhangyang-pc/mascot/cgi/protein_view.pl?file=../data/20140118/F012106.dat&hit=1) **Mass: 32511 Score: 44 Expect: 1.8 Matches: 12** | | | | | | | | | | | | | | | | | | | | | | | | | | | | | | | | | | | | | | | | | | | | | | | | | | | | | | | | | | | | | | | | | | | | | | | | | | | | | | | | | |  | | | | Observed | | | | | | | | Mr(expt) | | | | | | | | Mr(calc) | | | | | | | | ppm | | | | | | | | Start | | | | | | | | |  | | | | | | | End | | | | | | | | Miss | | | | | | | | Ions | | | | | | | Peptide | | | | | | | | | | | | | | | 700.2736 | | | | | | | | 699.2663 | | | | | | | | 699.2390 | | | | | | | | 39.0 | | | | | | | | 234 | | | | | | | | | - | | | | | | | 239 | | | | | | | | 0 | | | | | | | | --- | | | | | | | K.MECSCK.G | | | | | | | | | | | | | | | 713.2922 | | | | | | | | 712.2850 | | | | | | | | 712.4595 | | | | | | | | -245.05 | | | | | | | | 175 | | | | | | | | | - | | | | | | | 180 | | | | | | | | 0 | | | | | | | | --- | | | | | | | R.NIVIVR.S | | | | | | | | | | | | | | | 716.2467 | | | | | | | | 715.2394 | | | | | | | | 715.2339 | | | | | | | | 7.72 | | | | | | | | 234 | | | | | | | | | - | | | | | | | 239 | | | | | | | | 0 | | | | | | | | --- | | | | | | | K.MECSCK.G + Oxidation (M) | | | | | | | | | | | | | | | 766.2606 | | | | | | | | 765.2533 | | | | | | | | 765.3439 | | | | | | | | -118.42 | | | | | | | | 279 | | | | | | | | | - | | | | | | | 285 | | | | | | | | 0 | | | | | | | | --- | | | | | | | R.MSSSAQR.D | | | | | | | | | | | | | | | 960.3368 | | | | | | | | 959.3295 | | | | | | | | 959.5036 | | | | | | | | -181.43 | | | | | | | | 89 | | | | | | | | | - | | | | | | | 97 | | | | | | | | 1 | | | | | | | | --- | | | | | | | K.KGIVSDGER.S | | | | | | | | | | | | | | | 1051.5371 | | | | | | | | 1050.5298 | | | | | | | | 1050.4586 | | | | | | | | 67.8 | | | | | | | | 258 | | | | | | | | | - | | | | | | | 267 | | | | | | | | 1 | | | | | | | | --- | | | | | | | K.GNKNCEVCGK.E | | | | | | | | | | | | | | | 1099.3989 | | | | | | | | 1098.3916 | | | | | | | | 1098.5418 | | | | | | | | -136.67 | | | | | | | | 149 | | | | | | | | | - | | | | | | | 158 | | | | | | | | 1 | | | | | | | | --- | | | | | | | R.TSGEQHKANK.G | | | | | | | | | | | | | | | 1151.5374 | | | | | | | | 1150.5301 | | | | | | | | 1150.5149 | | | | | | | | 13.2 | | | | | | | | 279 | | | | | | | | | - | | | | | | | 288 | | | | | | | | 1 | | | | | | | | --- | | | | | | | R.MSSSAQRDNR.R | | | | | | | | | | | | | | | 1172.4034 | | | | | | | | 1171.3961 | | | | | | | | 1171.6309 | | | | | | | | -200.40 | | | | | | | | 156 | | | | | | | | | - | | | | | | | 167 | | | | | | | | 1 | | | | | | | | --- | | | | | | | K.ANKGTAPTAVSR.S | | | | | | | | | | | | | | | 1241.3402 | | | | | | | | 1240.3329 | | | | | | | | 1240.5367 | | | | | | | | -164.27 | | | | | | | | 290 | | | | | | | | | - | | | | | | | 299 | | | | | | | | 0 | | | | | | | | --- | | | | | | | R.NHSQQTMHSR.S + Oxidation (M) | | | | | | | | | | | | | | | 1428.4448 | | | | | | | | 1427.4375 | | | | | | | | 1427.6939 | | | | | | | | -179.60 | | | | | | | | 60 | | | | | | | | | - | | | | | | | 72 | | | | | | | | 0 | | | | | | | | --- | | | | | | | R.TGRPSLQSQHSCK.G | | | | | | | | | | | | | | | 1453.5209 | | | | | | | | 1452.5136 | | | | | | | | 1452.8776 | | | | | | | | -250.55 | | | | | | | | 175 | | | | | | | | | - | | | | | | | 187 | | | | | | | | 1 | | | | | | | | --- | | | | | | | R.NIVIVRSVSLPTR.R | | | | | | | | | | | | | | | No match to: 705.2664, 711.2261, 717.2345, 723.2507, 724.3429, 729.2244, 731.2399, 733.2346, 734.3154, 736.3335, 738.2560, 742.2830, 743.2640, 744.2788, 747.2585, 757.2798, 758.2543, 768.3980, 784.2988, 834.3463, 842.3667, 848.3603, 850.3921, 850.3921, 856.3624, 856.3624, 868.4025, 870.3840, 882.4204, 908.2927, 910.3796, 916.3361, 924.3634, 932.1627, 935.3383, 938.3616, 948.2776, 968.3980, 973.3891, 982.4241, 993.3755, 995.4652, 996.4401, 1012.4005, 1017.4966, 1019.4949, 1032.3983, 1033.4631, 1043.3519, 1045.4083, 1059.4100, 1059.4100, 1070.2513, 1073.3066, 1086.3425, 1087.2252, 1088.2490, 1088.2490, 1102.2654, 1102.2654, 1110.2319, 1119.4026, 1122.4139, 1126.3877, 1131.4407, 1133.5530, 1142.3176, 1165.5520, 1165.5520, 1185.4388, 1205.4727, 1214.1268, 1228.3793, 1229.4077, 1279.4888, 1332.5114, 1334.6632, 1345.3988, 1363.4353, 1363.4353, 1370.4856, 1375.4390, 1375.4390, 1391.4276, 1424.5504, 1484.4752, 1753.5476, 1939.6606, 2002.7570, 2002.7570, 2220.7703, 2224.7395, 2238.7834, 2238.7834, 2245.8442, 2260.7715, 2435.8391, 3346.1062 | | | | | | | | | | | | | | | | | | | | | | | | | | | | | | | | | | | | | | | | | | | | | | | | | | | | | | | | | | | | | | | | | | | | | | | | | | | | | | | | | |  | | | | **S55** [**Cs2g22650.3**](http://zhangyang-pc/mascot/cgi/protein_view.pl?file=../data/20140118/F012149.dat&hit=1) **Mass: 22301 Score: 52 Expect: 0.29 Matches: 6** | | | | | | | | | | | | | | | | | | | | | | | | | | | | | | | | | | | | | | | | | | | | | | | | | | | | | | | | | | | | | | | | | | | | | | | | | | | | | | | | | |  | | | | Observed | | | | | | | | Mr(expt) | | | | | | | | Mr(calc) | | | | | | | | ppm | | | | | | | | Start | | | | | | | | |  | | | | | | | End | | | | | | | | Miss | | | | | | | | Ions | | | | | | | Peptide | | | | | | | | | | | | | | | 761.2936 | | | | | | | | 760.2864 | | | | | | | | 760.4331 | | | | | | | | -192.91 | | | | | | | | 108 | | | | | | | | | - | | | | | | | 114 | | | | | | | | 0 | | | | | | | | --- | | | | | | | R.TLTEGLK.G | | | | | | | | | | | | | | | 1120.3867 | | | | | | | | 1119.3794 | | | | | | | | 1119.5812 | | | | | | | | -180.22 | | | | | | | | 115 | | | | | | | | | - | | | | | | | 124 | | | | | | | | 0 | | | | | | | | --- | | | | | | | K.GDPTYLVVEK.D | | | | | | | | | | | | | | | 1120.3867 | | | | | | | | 1119.3794 | | | | | | | | 1119.5812 | | | | | | | | -180.22 | | | | | | | | 115 | | | | | | | | | - | | | | | | | 124 | | | | | | | | 0 | | | | | | | | 36 | | | | | | | K.GDPTYLVVEK.D | | | | | | | | | | | | | | | 1363.4437 | | | | | | | | 1362.4364 | | | | | | | | 1362.7031 | | | | | | | | -195.69 | | | | | | | | 115 | | | | | | | | | - | | | | | | | 126 | | | | | | | | 1 | | | | | | | | --- | | | | | | | K.GDPTYLVVEKDK.T | | | | | | | | | | | | | | | 1495.5035 | | | | | | | | 1494.4962 | | | | | | | | 1494.7507 | | | | | | | | -170.28 | | | | | | | | 189 | | | | | | | | | - | | | | | | | 200 | | | | | | | | 0 | | | | | | | | --- | | | | | | | K.VVFVPWTETDFR.T | | | | | | | | | | | | | | | 1772.6469 | | | | | | | | 1771.6396 | | | | | | | | 1771.9580 | | | | | | | | -179.70 | | | | | | | | 171 | | | | | | | | | - | | | | | | | 188 | | | | | | | | 0 | | | | | | | | --- | | | | | | | R.GPAPLSLALAHANIENGK.V | | | | | | | | | | | | | | | No match to: 722.1837, 830.2790, 842.3500, 937.2210, 949.2241, 971.2147, 1059.3435, 1101.2837, 1113.2805, 1142.3503, 1160.3226, 1160.3226, 1186.3184, 1230.3202, 1244.3380, 1246.3180, 1424.4445, 1436.4580, 1455.4783, 1473.4965, 1481.4684, 1497.4956, 1499.4943, 1499.4943, 1508.5004, 1509.4907, 1510.5004, 1511.4933, 1511.4933, 1521.4606, 1525.4773, 1527.4897, 1527.4897, 1533.4763, 1537.4615, 1541.4843, 1543.4844, 1549.4703, 1551.5011, 1570.5142, 1581.5063, 1591.5005, 1595.5175, 1607.4971, 1609.5072, 1613.4667, 1635.5095, 1637.5483, 1639.5145, 1652.5671, 1655.4958, 1655.4958, 1663.5309, 1665.5200, 1667.5018, 1675.5358, 1681.5394, 1687.5315, 1705.4603, 1707.5247, 1711.5264, 1729.5197, 1744.4863, 1745.4574, 1746.4934, 1758.6165, 1762.4415, 1762.4415, 1778.4548, 1779.4685, 1784.4819, 1794.6239, 1812.4283, 1869.4036, 2161.6309, 2179.6602, 2191.6643, 2206.6526, 2207.6545, 2218.6516, 2222.6733, 2224.6724, 2224.6724, 2234.6782, 2236.6677, 2236.6677, 2250.6672, 2252.6633, 2252.6633, 2256.6543, 2258.6550, 2263.6274, 2266.6301, 2274.5969, 2276.6443, 2277.6643, 2280.6877, 2291.6819, 2307.6685, 2320.6523, 2333.6626, 2340.6501, 2375.6538, 2388.7080 | | | | | | | | | | | | | | | | | | | | | | | | | | | | | | | | | | | | | | | | | | | | | | | | | | | | | | | | | | | | | | | | | | | | | | | | | | | | | | | | | |  | | | |
