# Supplementary material for: Low pH-responsive proteins revealed by a 2-DE based MS approach and related physiological responses in Citrus leaves
Source: BMC Plant Biol. 2018 Sep 12;18:188. doi: 10.1186/s12870-018-1413-3 (PMC6134590; doi:10.1186/s12870-018-1413-3)
Supplement: Supplementary file 5 — Figure S2. Close-up views of 22 DAP spots in pH 2.5, pH 3 and pH 6-treated C. grandis and C. sinensis leaves. (PDF 132 kb) [file 12870_2018_1413_MOESM5_ESM.pdf]

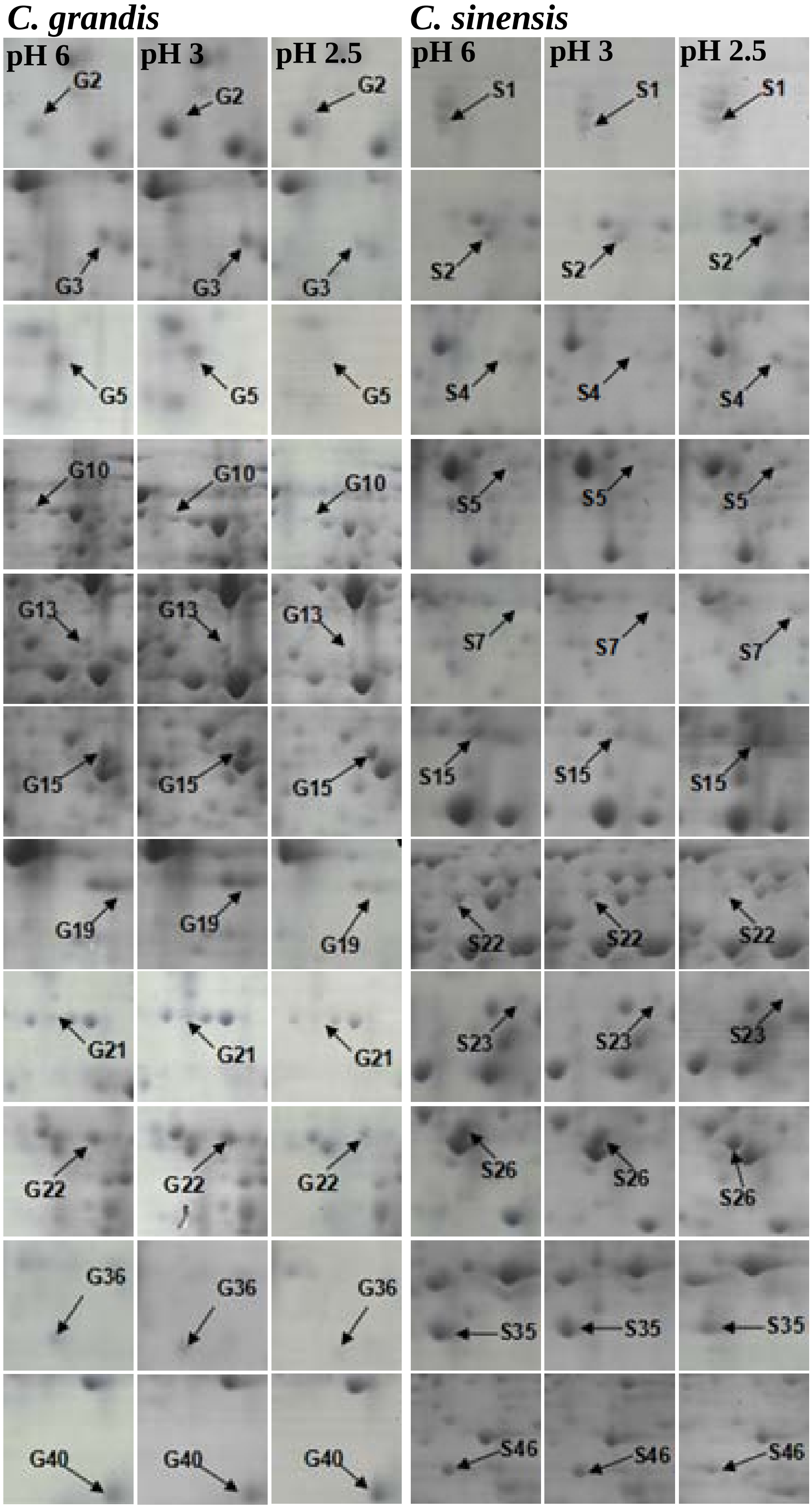

Additional file 5: Figure S2. Close-up views of 22 DAP spots in pH 2.5, pH 3 and pH 6-treated *C. grandis* and *C. sinensis* leaves.
