# Supplementary material for: Low pH-responsive proteins revealed by a 2-DE based MS approach and related physiological responses in Citrus leaves
Source: BMC Plant Biol. 2018 Sep 12;18:188. doi: 10.1186/s12870-018-1413-3 (PMC6134590; doi:10.1186/s12870-018-1413-3)
Supplement: Supplementary file 4 — Table S3. Master list of proteins identified in MALDI TOF/TOF MS from pH 2.5 and/or pH 3-treated C. grandis leaves using 2DE and DIGE experiments. (DOC 1467 kb) [file 12870_2018_1413_MOESM4_ESM.doc]

**Additional file 4: Table S3.** Master list of proteins identified in MALDI TOF/TOF MS from pH 2.5 and/or pH 3-treated *C. grandis* leaves using 2DE and DIGE experiments

**G2	Cs8g15520.1	Mass: 25986	Score: 341	Expect: 3.5e-030	Matches: 8**

Observed

Mr(expt)

Mr(calc)

ppm

Start

End

Miss

Ions

Peptide

1426.4572

1425.4499

1425.6961

-172.64

209

-

223

0

---

K.GGHELSLTTGNAGGR.L

1426.4572

1425.4499

1425.6961

-172.64

209

-

223

0

119

K.GGHELSLTTGNAGGR.L

1471.4786

1470.4713

1470.7355

-179.59

196

-

208

0

---

R.AFVVHELEDDLGK.G

1471.4786

1470.4713

1470.7355

-179.59

196

-

208

0

99

R.AFVVHELEDDLGK.G

2429.7693

2428.7620

2429.2035

-181.72

91

-

114

0

---

K.GTSNVEGVVTLTQEDGGPTTVNVR.V

2429.7693

2428.7620

2429.2035

-181.72

91

-

114

0

106

K.GTSNVEGVVTLTQEDGGPTTVNVR.V

2878.9070

2877.8997

2878.4210

-181.08

196

-

223

1

---

R.AFVVHELEDDLGKGGHELSLTTGNAGGR.L

3571.1296

3570.1223

3570.8027

-190.53

160

-

195

0

---

R.HAGDLGNIVANANGVAEATIVDNQISLDGPNTVVGR.A

No match to: 700.2584, 713.2726, 716.2284, 724.3541, 730.2378, 734.3450, 744.2559, 750.3771, 768.3785, 771.1887, 807.2369, 834.3326, 842.3459, 847.2881, 848.3259, 850.3731, 860.9101, 864.3612, 868.3791, 876.8760, 882.3904, 900.3349, 919.2824, 982.3683, 994.2555, 996.3411, 999.3221, 1017.4691, 1042.3378, 1051.5051, 1059.3768, 1059.3768, 1082.3655, 1082.3655, 1092.3390, 1104.3420, 1106.3240, 1107.3440, 1113.2900, 1118.3114, 1132.3739, 1151.5059, 1165.3821, 1179.3936, 1179.3936, 1194.3756, 1265.4199, 1277.4412, 1279.4238, 1302.5253, 1308.4166, 1315.4122, 1320.3665, 1332.2899, 1334.6251, 1343.3771, 1365.4049, 1365.4049, 1373.4183, 1379.4542, 1383.4258, 1421.4351, 1432.4399, 1434.5314, 1440.4758, 1448.4552, 1453.4961, 1475.5081, 1479.4954, 1485.4929, 1491.4590, 1493.4697, 1507.4858, 1516.4463, 1528.4972, 1657.5095, 1657.5095, 1687.6573, 1699.5352, 1707.4841, 1707.4841, 1716.5538, 1873.6299, 1908.4839, 1993.6353, 2002.6897, 2063.7576, 2220.7122, 2238.7144, 2245.7666, 2305.7141, 2324.7927, 2383.5244, 2383.5244, 2411.7676, 2427.7412, 2443.7832, 2451.7263, 2810.8071, 2903.8455, 2941.9072, 3311.6968

**G11	Cs5g05240.1	Mass: 58874	Score: 409	Expect: 5.6e-037	Matches: 27**

Observed

Mr(expt)

Mr(calc)

ppm

Start

End

Miss

Ions

Peptide

740.3214

739.3141

739.4592

-196.30

527

-

533

0

---

K.AVVQPVK.N

775.2892

774.2819

774.4276

-188.13

367

-

373

0

---

R.IVGDPFK.N

804.2551

803.2479

803.3926

-180.16

345

-

350

0

---

R.TYVHER.V

812.2047

811.1974

811.3534

-192.26

116

-

121

0

---

K.MTPYER.S + Oxidation (M)

819.2758

818.2685

818.5126

-298.21

390

-

395

1

---

K.VLRYIR.S

847.2523

846.2450

846.4083

-192.96

449

-

455

0

---

K.ETDDVIR.R

931.3598

930.3525

930.5287

-189.33

366

-

373

1

---

R.RIVGDPFK.N

932.3259

931.3186

931.4797

-172.96

157

-

164

0

---

K.SEVPMVVR.L + Oxidation (M)

994.3230

993.3157

993.4920

-177.42

74

-

81

0

---

K.TFPVYDPR.T

994.3230

993.3157

993.4920

-177.42

74

-

81

0

61

K.TFPVYDPR.T

1010.3692

1009.3619

1009.5597

-195.88

462

-

471

0

---

R.FGLAAGVFTK.S

1028.3114

1027.3041

1027.4862

-177.23

351

-

358

0

---

R.VYDEFVEK.A

1028.3114

1027.3041

1027.4862

-177.23

351

-

358

0

21

R.VYDEFVEK.A

1122.3889

1121.3816

1121.5717

-169.46

447

-

455

1

---

K.FKETDDVIR.R

1129.2716

1128.2643

1128.4539

-168.04

472

-

481

0

---

K.SADTANTMMR.A + 2 Oxidation (M)

1267.4802

1266.4729

1266.7085

-185.95

460

-

471

1

---

K.TRFGLAAGVFTK.S

1368.5063

1367.4990

1367.7449

-179.75

515

-

526

0

---

K.GIYSLNAYLQVK.A

1368.5063

1367.4990

1367.7449

-179.75

515

-

526

0

54

K.GIYSLNAYLQVK.A

1433.4917

1432.4844

1432.7310

-172.13

60

-

73

0

---

K.NLINGQFVDAASGK.T

1505.5327

1504.5254

1504.8137

-191.55

230

-

243

0

---

K.SAEQTPLTALYLAK.L

1813.5485

1812.5412

1812.8642

-178.18

374

-

389

0

---

K.NGVEQGPQIDPEQFQK.V

1813.5485

1812.5412

1812.8642

-178.18

374

-

389

0

61

K.NGVEQGPQIDPEQFQK.V

1887.5875

1886.5802

1886.8857

-161.92

82

-

99

0

---

R.TAEVIANVAEGDAEDIDR.A

1887.5875

1886.5802

1886.8857

-161.92

82

-

99

0

115

R.TAEVIANVAEGDAEDIDR.A

1948.6702

1947.6629

1947.9068

-125.21

396

-

414

1

---

R.SGIESNATLECGGDRLGNR.G

2121.5481

2120.5408

2120.0031

254

462

-

481

1

---

R.FGLAAGVFTKSADTANTMMR.A + 2 Oxidation (M)

3406.0771

3405.0698

3405.7027

-185.84

244

-

277

0

---

K.LFHEAGLPPGVLNVVSGYGPTAGAALASHMDVDK.L + Oxidation (M)

No match to: 734.3541, 768.3721, 776.2678, 780.2289, 803.2929, 828.2039, 830.2676, 834.2203, 842.3535, 850.2790, 868.3406, 910.2814, 914.2458, 1021.3557, 1021.3557, 1032.3807, 1038.3022, 1050.3104, 1059.3999, 1062.3005, 1170.4435, 1178.3834, 1185.4364, 1187.4595, 1190.3839, 1190.3839, 1206.3624, 1212.3691, 1261.5021, 1261.5021, 1280.4730, 1334.3796, 1340.4327, 1352.4332, 1352.4332, 1363.4535, 1390.4232, 1420.4836, 1434.4978, 1444.5029, 1447.5219, 1448.5283, 1465.5117, 1465.5117, 1474.4530, 1478.5221, 1487.4901, 1497.5048, 1516.4896, 1518.4633, 1537.5092, 1581.4254, 1619.5699, 1665.4878, 1673.6003, 1707.5304, 1792.5797, 1797.4996, 1814.5475, 1825.5809, 1835.5411, 1851.5194, 1869.5717, 1901.5649, 1964.6548, 1993.6375, 2155.7603, 2177.7891, 2185.6013, 2193.7451, 2201.6013, 2245.7104, 2555.7705, 2566.7834, 2568.7688, 2571.8003, 2582.7512, 2588.7581, 2813.9258, 3234.1125, 3343.1409, 3422.0652, 3844.1367

**G40	Cs4g01600.1	Mass: 21504	Score: 264 Expect: 1.8e-022	Matches: 11**

Observed

Mr(expt)

Mr(calc)

ppm

Start

End

Miss

Ions

Peptide

778.2257

777.2184

777.3691

-193.79

6

-

11

0

---

K.EMEVVR.G + Oxidation (M)

906.2953

905.2881

905.4640

-194.33

5

-

11

1

---

K.KEMEVVR.G + Oxidation (M)

925.2989

924.2917

924.4552

-176.94

65

-

73

0

---

R.GSIEGTAYK.A

936.3630

935.3557

935.3985

-45.72

56

-

63

0

---

R.NETWSDGK.R

1255.3932

1254.3859

1254.6033

-173.26

22

-

31

0

---

R.WYEIASFPSR.N

1575.4999

1574.4926

1574.7689

-175.43

42

-

55

0

---

R.ATYTLNEDGTVHVR.N

1575.4999

1574.4926

1574.7689

-175.43

42

-

55

0

106

R.ATYTLNEDGTVHVR.N

1831.5000

1830.4927

1830.8458

-192.84

130

-

144

0

---

R.EPHMDEAIYNQLVEK.A + Oxidation (M)

1831.5000

1830.4927

1830.8458

-192.84

130

-

144

0

38

R.EPHMDEAIYNQLVEK.A + Oxidation (M)

1969.5072

1968.4999

1968.8548

-180.27

158

-

175

0

---

R.TPQSDNPPEAEESPQDTK.G

1969.5072

1968.4999

1968.8548

-180.27

158

-

175

0

94

R.TPQSDNPPEAEESPQDTK.G

No match to: 713.2759, 716.2217, 734.3472, 750.3727, 768.3825, 822.2938, 834.2966, 838.2955, 842.3496, 848.2856, 850.2905, 850.2905, 856.3152, 864.3185, 868.3699, 882.3850, 948.3575, 952.2330, 964.3598, 968.2863, 986.3332, 996.3673, 998.3385, 1014.3242, 1043.3306, 1051.5153, 1055.3292, 1059.3549, 1069.3479, 1071.3392, 1096.3119, 1096.3119, 1108.3087, 1108.3087, 1123.3431, 1124.3237, 1124.3237, 1125.3406, 1148.3164, 1232.3323, 1237.5012, 1240.3588, 1242.3646, 1251.3621, 1254.3640, 1259.3851, 1259.3851, 1263.3678, 1265.3739, 1267.3860, 1268.3777, 1270.3667, 1271.3812, 1271.3812, 1281.3584, 1287.3794, 1287.3794, 1293.3671, 1294.3673, 1296.3802, 1299.3763, 1301.3838, 1306.3654, 1309.3788, 1311.3929, 1312.3846, 1315.3900, 1319.3933, 1330.3925, 1337.3964, 1339.3962, 1341.3931, 1351.3896, 1354.3986, 1355.3949, 1368.3925, 1369.3998, 1383.3735, 1385.3866, 1395.4102, 1398.4178, 1400.4203, 1409.4647, 1418.4122, 1425.4293, 1434.4199, 1437.4309, 1450.4440, 1464.4915, 1531.4606, 1573.4879, 1589.5033, 1597.4766, 1767.5330, 1813.5269, 1847.4971, 1853.5040, 1991.5118, 2238.7451

**G15	orange1.1t00331.1	Mass: 28141	Score: 561	Expect: 3.5e-052	Matches: 25**

Observed

Mr(expt)

Mr(calc)

ppm

Start

End

Miss

Ions

Peptide

720.2728

719.2655

719.4000

-186.96

158

-

163

0

---

K.MVSVLR.E + Oxidation (M)

869.2968

868.2895

868.4443

-178.22

118

-

124

0

---

R.GTFIEFR.S

869.2968

868.2895

868.4443

-178.22

118

-

124

0

27

R.GTFIEFR.S

877.3829

876.3757

876.5293

-175.34

151

-

157

0

---

K.IHNIRPK.M

1025.3711

1024.3638

1024.5454

-177.24

117

-

124

1

---

K.RGTFIEFR.S

1101.3018

1100.2945

1100.4774

-166.21

143

-

150

1

---

R.DEFERYDK.I

1160.4158

1159.4085

1159.6019

-166.79

125

-

135

0

---

R.SGMLNISPIGR.N + Oxidation (M)

1160.4158

1159.4085

1159.6019

-166.79

125

-

135

0

35

R.SGMLNISPIGR.N + Oxidation (M)

1283.4918

1282.4845

1282.7146

-179.34

148

-

157

1

---

R.YDKIHNIRPK.M

1316.4719

1315.4646

1315.7348

-205.32

39

-

52

0

---

K.VVTVGVVGGSDLSK.I

1326.3882

1325.3809

1325.6108

-173.40

24

-

34

0

---

K.AATPQMLEFMR.E + 2 Oxidation (M)

1326.3882

1325.3809

1325.6108

-173.40

24

-

34

0

11

K.AATPQMLEFMR.E + 2 Oxidation (M)

1389.3759

1388.3686

1388.5957

-163.50

213

-

224

0

---

K.GGNDHEIFESER.T

1389.3759

1388.3686

1388.5957

-163.50

213

-

224

0

95

K.GGNDHEIFESER.T

1454.4546

1453.4473

1453.7057

-177.76

23

-

34

1

---

R.KAATPQMLEFMR.E + 2 Oxidation (M)

1454.4546

1453.4473

1453.7057

-177.76

23

-

34

1

13

R.KAATPQMLEFMR.E + 2 Oxidation (M)

1759.5004

1758.4931

1758.7890

-168.20

196

-

209

0

---

R.YLDDFNEIHFFGDK.T

1759.5004

1758.4931

1758.7890

-168.20

196

-

209

0

66

R.YLDDFNEIHFFGDK.T

1781.5001

1780.4928

1780.8016

-173.40

210

-

224

1

---

K.TYKGGNDHEIFESER.T

1786.6847

1785.6774

1785.9625

-159.63

6

-

22

0

---

K.QGLLALFDVDGTLTAPR.K

1914.7524

1913.7451

1914.0575

-163.18

6

-

23

1

---

K.QGLLALFDVDGTLTAPRK.A

1914.7524

1913.7451

1914.0575

-163.18

5

-

22

1

122

R.KQGLLALFDVDGTLTAPR.K

2198.6919

2197.6846

2198.0532

-167.67

60

-

78

0

---

K.TVIDEYDYVFSENGLVAHK.D

2198.6919

2197.6846

2198.0532

-167.67

60

-

78

0

94

K.TVIDEYDYVFSENGLVAHK.D

2498.7568

2497.7495

2498.1965

-178.93

60

-

81

1

---

K.TVIDEYDYVFSENGLVAHKDGK.L

No match to: 700.2770, 712.1834, 713.2831, 716.2706, 724.3705, 726.2626, 727.3040, 734.3593, 736.3626, 750.3876, 768.3866, 782.3458, 807.2438, 819.2497, 830.2356, 834.3568, 836.3689, 842.3614, 848.3771, 850.3849, 860.9175, 864.3835, 866.3709, 868.3932, 876.8901, 882.4010, 891.2703, 956.3688, 956.3688, 968.3895, 982.4093, 996.4103, 1017.4868, 1019.5131, 1051.5095, 1059.3909, 1096.4480, 1117.5167, 1145.4778, 1151.5022, 1165.5179, 1176.4081, 1179.4167, 1237.3792, 1261.5266, 1265.4709, 1274.4688, 1300.6151, 1315.4230, 1320.3817, 1329.4044, 1334.6482, 1342.3833, 1358.3948, 1361.4836, 1371.3563, 1373.4124, 1403.3835, 1411.3536, 1434.5990, 1448.6494, 1465.5140, 1469.4845, 1470.4537, 1486.4601, 1488.4843, 1493.4597, 1619.6339, 1707.4781, 1731.7318, 1741.5197, 1747.6648, 1768.6678, 1769.6672, 1769.6672, 1797.4938, 1896.7457, 1936.6799, 1937.7263, 2153.7185, 2164.6260, 2191.7368, 2220.6731, 2284.7712, 2678.8057

**G1	Cs5g21010.1	Mass: 36014	Score: 435	Expect: 1.4e-039	Matches: 18**

Observed

Mr(expt)

Mr(calc)

ppm

Start

End

Miss

Ions

Peptide

958.3704

957.3631

957.5859

-232.58

29

-

37

0

---

K.IITGSINLK.V

1051.5287

1050.5214

1050.4651

53.6

1

-

10

0

---

-.MAGEEAVSNK.Q + Oxidation (M)

1127.4183

1126.4110

1126.5982

-166.15

194

-

203

1

---

K.EPDLDAALKR.C

1176.4789

1175.4716

1175.6624

-162.27

279

-

288

0

---

K.FLELVMPAIK.E + Oxidation (M)

1176.4789

1175.4716

1175.6624

-162.27

279

-

288

0

31

K.FLELVMPAIK.E + Oxidation (M)

1207.4244

1206.4171

1206.6100

-159.89

220

-

229

0

---

K.MLDAVLLNMR.I + 2 Oxidation (M)

1207.4244

1206.4171

1206.6100

-159.89

220

-

229

0

6

K.MLDAVLLNMR.I + 2 Oxidation (M)

1223.3412

1222.3339

1222.5295

-159.97

183

-

192

0

---

K.FGFDDAFNYK.K

1223.3412

1222.3339

1222.5295

-159.97

183

-

192

0

74

K.FGFDDAFNYK.K

1351.4534

1350.4461

1350.6245

-132.03

183

-

193

1

---

K.FGFDDAFNYKK.E

1465.5488

1464.5415

1464.6674

-85.92

181

-

192

1

---

K.NKFGFDDAFNYK.K

1465.5488

1464.5415

1464.7977

-174.87

11

-

23

0

64

K.QVILSNYVTGFPK.E

1477.5461

1476.5388

1476.7711

-157.32

292

-

304

0

---

K.LVYVEDIAEGLEK.A

1477.5461

1476.5388

1476.7711

-157.32

292

-

304

0

63

K.LVYVEDIAEGLEK.A

1558.6011

1557.5938

1557.8515

-165.41

305

-

320

0

---

K.APSALVGIFTGQNVGK.Q

1558.6011

1557.5938

1557.8515

-165.41

305

-

320

0

116

K.APSALVGIFTGQNVGK.Q

1818.5162

1817.5089

1817.8083

-164.69

264

-

278

0

---

R.MEGFLAGDFYHQYPK.F + Oxidation (M)

1818.5162

1817.5089

1817.8083

-164.69

264

-

278

0

40

R.MEGFLAGDFYHQYPK.F + Oxidation (M)

No match to: 700.2690, 704.2212, 705.2413, 707.2320, 709.1974, 711.2167, 713.2833, 716.2500, 725.2481, 728.2086, 730.2128, 734.3367, 739.2895, 744.2532, 750.3774, 752.2736, 756.2936, 758.3029, 768.3915, 782.3188, 784.2661, 800.2969, 811.2751, 833.3158, 834.3202, 842.3589, 850.3851, 860.9235, 860.9235, 868.3940, 876.8823, 882.4098, 900.2544, 914.3863, 931.3023, 937.3165, 946.3077, 982.3767, 996.4159, 1041.3713, 1045.3910, 1059.3905, 1104.4121, 1107.3931, 1123.3889, 1142.4016, 1147.4451, 1151.5237, 1165.5132, 1173.3927, 1187.4523, 1205.4161, 1254.4142, 1254.4142, 1262.3618, 1292.4073, 1334.6158, 1337.4473, 1338.4395, 1340.4420, 1350.5112, 1352.4131, 1356.4189, 1367.4897, 1372.4346, 1382.4697, 1405.4652, 1431.4459, 1444.6056, 1448.5496, 1448.5496, 1458.5344, 1463.3606, 1470.5333, 1472.5270, 1487.5310, 1499.5339, 1580.6003, 1596.5344, 1613.5764, 1805.5878, 1806.5342, 1834.5319, 1840.5012, 1843.5128, 1907.7206, 2077.7324, 2108.7417, 2238.7729, 2245.8325, 2281.7236, 2395.8235

**G3	Cs3g26690.1	Mass: 38994	Score: 472	Expect: 2.8e-043	Matches: 18**

Observed

Mr(expt)

Mr(calc)

ppm

Start

End

Miss

Ions

Peptide

716.2437

715.2364

715.3864

-209.77

106

-

113

0

---

K.ATAAPGTK.N

999.4085

998.4012

998.5760

-175.09

332

-

340

0

---

K.GELIGEILR.Y

999.4085

998.4012

998.5760

-175.09

332

-

340

0

56

K.GELIGEILR.Y

1024.3372

1023.3299

1023.5178

-183.57

196

-

203

0

---

K.VPHFSFYK.S

1024.3372

1023.3299

1023.5178

-183.57

196

-

203

0

41

K.VPHFSFYK.S

1090.4351

1089.4278

1089.6182

-174.75

141

-

150

0

---

K.LVVVEFAASR.S

1090.4351

1089.4278

1089.6182

-174.75

141

-

150

0

71

K.LVVVEFAASR.S

1095.3640

1094.3567

1094.5543

-180.49

281

-

290

0

---

R.QMAGTVVFAR.M + Oxidation (M)

1256.4133

1255.4060

1255.6271

-176.06

157

-

166

0

---

K.IYPFMVDLSR.Q + Oxidation (M)

1256.4133

1255.4060

1255.6271

-176.06

157

-

166

0

32

K.IYPFMVDLSR.Q + Oxidation (M)

1444.4594

1443.4521

1443.6994

-171.27

124

-

135

0

---

K.VHSIEEFDEALR.L

1444.4594

1443.4521

1443.6994

-171.27

124

-

135

0

106

K.VHSIEEFDEALR.L

1572.5405

1571.5332

1571.7943

-166.13

123

-

135

1

---

K.KVHSIEEFDEALR.L

1572.5405

1571.5332

1571.7943

-166.13

123

-

135

1

80

K.KVHSIEEFDEALR.L

1695.5870

1694.5797

1694.8702

-171.38

305

-

318

0

---

R.DMNVVEVPTFLFIR.D + Oxidation (M)

1695.5870

1694.5797

1694.8702

-171.38

305

-

318

0

57

R.DMNVVEVPTFLFIR.D + Oxidation (M)

1707.4535

1706.4462

1706.9203

-277.73

141

-

156

1

---

K.LVVVEFAASRSLDSSK.I

1772.5251

1771.5178

1772.0084

-276.83

2

-

17

0

---

M.TEIFIIDISVVSALPR.S

No match to: 700.2673, 713.2794, 722.1815, 730.2371, 734.3583, 744.2649, 750.3591, 757.2369, 768.3747, 782.2908, 834.3474, 842.3534, 848.3660, 850.3741, 860.9062, 864.3270, 868.3798, 876.8751, 882.3931, 895.2725, 964.3554, 982.3767, 996.3937, 1017.4969, 1021.3726, 1033.4750, 1046.3352, 1051.5071, 1059.3796, 1078.3462, 1078.3462, 1080.3219, 1094.3407, 1107.3643, 1111.3569, 1131.4830, 1145.3672, 1151.5149, 1165.5045, 1168.3452, 1179.4030, 1191.3564, 1208.3832, 1261.4924, 1269.4054, 1272.4094, 1285.4121, 1315.4199, 1320.3732, 1334.6326, 1365.4257, 1434.5883, 1443.4349, 1448.6620, 1458.4889, 1465.4617, 1617.7433, 1630.5286, 1631.6285, 1656.3319, 1657.3712, 1677.5905, 1711.5940, 1717.5980, 1718.5962, 1734.6149, 1736.6194, 1736.6194, 1751.6033, 1758.5801, 1764.3160, 1862.5466, 1880.5518, 1880.5518, 1894.5751, 1902.5322, 1918.4962, 1921.5717, 2002.6934, 2006.6556, 2007.6376, 2021.6542, 2023.6287, 2028.5398, 2041.5994, 2069.6028, 2070.5806, 2086.6108, 2238.7520, 2245.7861, 2865.8542, 2880.8953

**G4	Cs3g26690.1	Mass: 38994	Score: 477	Expect: 8.8e-044	Matches: 20**

Observed

Mr(expt)

Mr(calc)

ppm

Start

End

Miss

Ions

Peptide

716.2258

715.2186

715.3864

-234.68

106

-

113

0

---

K.ATAAPGTK.N

819.2924

818.2851

818.4902

-250.59

271

-

277

0

---

K.VYPTVIK.L

969.4468

968.4395

968.6270

-193.58

255

-

263

0

---

K.LIVLDVGLK.H

999.3947

998.3874

998.5760

-188.85

332

-

340

0

---

K.GELIGEILR.Y

999.3947

998.3874

998.5760

-188.85

332

-

340

0

64

K.GELIGEILR.Y

1024.3290

1023.3217

1023.5178

-191.58

196

-

203

0

---

K.VPHFSFYK.S

1024.3290

1023.3217

1023.5178

-191.58

196

-

203

0

45

K.VPHFSFYK.S

1090.4233

1089.4160

1089.6182

-185.58

141

-

150

0

---

K.LVVVEFAASR.S

1090.4233

1089.4160

1089.6182

-185.58

141

-

150

0

66

K.LVVVEFAASR.S

1095.3580

1094.3507

1094.5543

-185.97

281

-

290

0

---

R.QMAGTVVFAR.M + Oxidation (M)

1240.4165

1239.4092

1239.6322

-179.85

157

-

166

0

---

K.IYPFMVDLSR.Q

1256.4001

1255.3928

1255.6271

-186.57

157

-

166

0

---

K.IYPFMVDLSR.Q + Oxidation (M)

1256.4001

1255.3928

1255.6271

-186.57

157

-

166

0

35

K.IYPFMVDLSR.Q + Oxidation (M)

1444.4406

1443.4333

1443.6994

-184.29

124

-

135

0

---

K.VHSIEEFDEALR.L

1444.4406

1443.4333

1443.6994

-184.29

124

-

135

0

106

K.VHSIEEFDEALR.L

1572.5228

1571.5155

1571.7943

-177.39

123

-

135

1

---

K.KVHSIEEFDEALR.L

1572.5228

1571.5155

1571.7943

-177.39

123

-

135

1

69

K.KVHSIEEFDEALR.L

1695.5715

1694.5642

1694.8702

-180.53

305

-

318

0

---

R.DMNVVEVPTFLFIR.D + Oxidation (M)

1695.5715

1694.5642

1694.8702

-180.53

305

-

318

0

59

R.DMNVVEVPTFLFIR.D + Oxidation (M)

2185.5825

2184.5752

2184.0079

260

167

-

185

1

---

R.QCNDVEFILVMGDESEKTK.E

No match to: 700.2618, 706.2083, 713.2712, 718.2479, 722.1859, 730.2368, 734.3452, 750.3646, 766.2291, 768.3748, 783.2524, 830.2741, 842.3458, 850.3357, 868.3586, 882.3831, 910.2728, 914.2306, 917.2914, 926.2791, 930.2622, 996.3539, 997.3397, 1013.4084, 1014.3662, 1021.3534, 1031.3840, 1046.3174, 1050.3188, 1051.5044, 1059.3699, 1062.3215, 1078.3347, 1078.3347, 1081.3488, 1094.3363, 1100.3347, 1111.3589, 1112.3602, 1116.3596, 1130.2281, 1132.2357, 1151.4849, 1158.3555, 1165.5159, 1170.4263, 1187.4550, 1187.4550, 1191.4728, 1192.4288, 1208.3700, 1223.4261, 1238.3905, 1261.4884, 1272.4094, 1278.3704, 1315.4077, 1334.6229, 1426.4542, 1443.4318, 1448.4706, 1458.4675, 1465.4972, 1466.4531, 1467.3893, 1482.4052, 1501.4725, 1550.3373, 1552.3451, 1586.5283, 1593.3953, 1607.6246, 1629.5400, 1631.5981, 1631.5981, 1640.3735, 1647.5730, 1655.5626, 1657.3690, 1673.3750, 1677.5950, 1678.4972, 1680.5837, 1711.5780, 1717.5613, 1741.6166, 1764.3092, 2044.4556, 2238.7283, 2245.7849

**G8	Cs6g20130.1	Mass: 16564	Score: 130	Expect: 4.4e-009	Matches: 7**

Observed

Mr(expt)

Mr(calc)

ppm

Start

End

Miss

Ions

Peptide

734.3413

733.3340

733.3792

-61.64

42

-

47

0

---

K.SVEIMR.K

750.2686

749.2613

749.3742

-150.55

42

-

47

0

---

K.SVEIMR.K + Oxidation (M)

900.2456

899.2383

899.4137

-194.99

49

-

55

0

---

K.FSEQYAR.R

900.2456

899.2383

899.4137

-194.99

49

-

55

0

52

K.FSEQYAR.R

1028.3239

1027.3166

1027.5087

-186.90

48

-

55

1

---

R.KFSEQYAR.R

1028.3239

1027.3166

1027.5087

-186.90

48

-

55

1

61

R.KFSEQYAR.R

1217.3444

1216.3371

1216.5547

-178.82

56

-

65

1

---

R.RSDTFFCVDK.S

No match to: 700.2629, 705.2559, 706.2382, 713.2698, 715.2387, 716.2253, 718.2557, 724.3585, 726.2866, 730.2380, 736.3320, 738.2181, 744.2430, 748.2553, 749.3140, 752.2490, 768.3777, 771.2825, 791.3453, 799.2283, 809.2622, 816.3262, 834.3373, 842.3430, 850.3688, 850.3688, 860.3959, 860.9014, 868.3763, 882.3780, 882.3780, 914.2485, 922.2396, 927.4097, 949.2712, 951.3479, 961.2793, 968.3924, 971.4518, 982.3659, 994.3376, 996.3760, 1010.3178, 1011.3022, 1017.4449, 1020.3666, 1027.2816, 1042.3373, 1045.3439, 1050.3080, 1051.5021, 1054.3230, 1059.3688, 1059.3688, 1063.3251, 1072.3121, 1099.3475, 1110.3297, 1111.4023, 1117.3582, 1126.3530, 1128.3884, 1149.4733, 1151.4908, 1165.4965, 1165.4965, 1168.3181, 1182.3563, 1183.3702, 1183.3702, 1202.3845, 1249.4037, 1251.4547, 1270.3673, 1272.3665, 1274.3545, 1274.3545, 1277.3273, 1282.3982, 1284.4207, 1290.3190, 1315.4006, 1331.4110, 1334.6104, 1424.5022, 1448.5938, 1492.4829, 1642.5667, 1936.5740, 1978.4531, 1994.4573, 2002.7032, 2002.7032, 2085.4875, 2219.6479, 2220.6904, 2238.7297, 2238.7297, 2245.7903, 2327.7217, 2423.6926, 2810.7815, 3346.0188

**G23	Cs3g19790.1	Mass: 55582	Score: 625	Expect: 1.4e-058	Matches: 31**

Observed

Mr(expt)

Mr(calc)

ppm

Start

End

Miss

Ions

Peptide

866.2891

865.2818

865.4181

-157.47

291

-

297

0

---

R.EVAEQYK.G

924.2925

923.2852

923.4501

-178.57

263

-

270

0

---

K.FFNSPNAK.A

936.2750

935.2677

935.4349

-178.65

462

-

469

0

---

K.TVPYEGDR.T

936.2750

935.2677

935.4349

-178.65

462

-

469

0

48

K.TVPYEGDR.T

987.3210

986.3138

986.5032

-192.08

140

-

149

0

---

K.QSGPASAEIK.S

1087.3866

1086.3793

1086.5670

-172.68

205

-

215

0

---

R.GESSVTGPVVR.L

1087.3866

1086.3793

1086.5670

-172.68

205

-

215

0

38

R.GESSVTGPVVR.L

1108.3704

1107.3631

1107.5560

-174.15

98

-

106

0

---

K.ELASQYEIR.G

1108.3704

1107.3631

1107.5560

-174.15

98

-

106

0

58

K.ELASQYEIR.G

1136.3860

1135.3787

1135.5761

-173.79

129

-

138

0

---

R.EADGIVEYLK.K

1165.4822

1164.4749

1164.5775

-88.08

462

-

471

1

---

K.TVPYEGDRTK.E

1240.3617

1239.3544

1239.5619

-167.37

150

-

161

0

---

K.SAEDASSFIGEK.K

1363.4258

1362.4185

1362.6415

-163.67

472

-

482

0

---

K.EDIVDFIENNR.D

1363.4258

1362.4185

1362.6415

-163.67

472

-

482

0

61

K.EDIVDFIENNR.D

1396.3785

1395.3712

1395.6055

-167.87

189

-

200

0

---

R.SDYEFGHTQNAK.L

1396.3785

1395.3712

1395.6055

-167.87

189

-

200

0

45

R.SDYEFGHTQNAK.L

1481.4659

1480.4586

1480.7045

-166.06

366

-

378

0

---

K.SEPIPEENNEPVK.V

1566.5298

1565.5225

1565.8889

-234.00

201

-

215

1

---

K.LLPRGESSVTGPVVR.L

1592.5245

1591.5172

1591.7842

-167.71

470

-

482

1

---

R.TKEDIVDFIENNR.D

1592.5245

1591.5172

1591.7842

-167.71

470

-

482

1

95

R.TKEDIVDFIENNR.D

1606.5314

1605.5241

1605.7634

-149.04

472

-

484

1

---

K.EDIVDFIENNRDK.A

1609.5381

1608.5308

1608.7995

-166.98

365

-

378

1

---

R.KSEPIPEENNEPVK.V

1620.5785

1619.5712

1619.8519

-173.25

73

-

88

0

---

K.AASELSSHDPPVVLAK.V

1644.5796

1643.5723

1643.8599

-174.96

216

-

228

0

---

R.LFKPFDELFVDFK.D

1644.5796

1643.5723

1643.8599

-174.96

216

-

228

0

22

R.LFKPFDELFVDFK.D

1738.5698

1737.5625

1737.8607

-171.60

379

-

394

0

---

K.VLVADSLQDMVFNSGK.N + Oxidation (M)

2078.6448

2077.6375

2077.9916

-170.38

89

-

106

1

---

K.VDANEEANKELASQYEIR.G

2078.6448

2077.6375

2077.9916

-170.38

89

-

106

1

84

K.VDANEEANKELASQYEIR.G

2706.7920

2705.7847

2706.2602

-175.71

433

-

456

0

---

K.FDATANDIPGDTFEVQGYPTVFFR.S

2706.7920

2705.7847

2706.2602

-175.71

433

-

456

0

71

K.FDATANDIPGDTFEVQGYPTVFFR.S

2802.9224

2801.9151

2802.4229

-181.17

238

-

262

0

---

K.FVEESSIPIVTVFNSDANNHPFVIK.F

No match to: 700.2592, 705.2570, 713.2816, 716.2394, 724.3676, 734.3492, 736.3723, 750.3817, 768.3865, 783.2683, 824.3604, 832.2752, 834.3301, 838.9246, 842.3487, 850.3671, 854.8953, 856.3649, 860.9121, 864.3389, 868.3865, 876.8826, 882.4029, 894.3539, 921.3901, 946.2894, 964.3408, 967.2966, 982.3778, 996.3842, 999.3238, 1009.3062, 1021.3257, 1051.5238, 1055.3519, 1059.3834, 1065.8861, 1090.3768, 1122.3901, 1145.3790, 1151.5146, 1163.3590, 1210.3542, 1222.3506, 1234.3854, 1297.4558, 1334.6473, 1345.4257, 1355.4427, 1373.4924, 1377.4120, 1398.3917, 1410.3982, 1418.3701, 1426.3961, 1444.4966, 1448.5673, 1488.5059, 1580.5792, 1580.5792, 1605.5331, 1617.5347, 1631.5352, 1642.5413, 1647.5527, 1650.6394, 1659.5469, 1698.5177, 1802.6604, 1832.6158, 1835.6041, 1874.6747, 1886.6763, 1902.6603, 2002.7186, 2144.6992, 2220.7214, 2238.7419, 2245.8113

**G39	Cs3g18360.1	Mass: 36075	Score: 422	Expect: 2.8e-038	Matches: 23**

Observed

Mr(expt)

Mr(calc)

ppm

Start

End

Miss

Ions

Peptide

745.2340

744.2267

744.3667

-188.11

123

-

128

0

---

R.QAYHAR.F

800.2763

799.2690

799.4188

-187.43

287

-

293

0

---

R.NSVPLDR.A

1023.2967

1022.2894

1022.4669

-173.55

68

-

75

0

---

K.ELTNDFER.V

1036.3516

1035.3443

1035.5237

-173.18

226

-

233

0

---

K.DEYLDLLR.A

1036.3516

1035.3443

1035.5237

-173.18

226

-

233

0

62

K.DEYLDLLR.A

1047.3328

1046.3255

1046.4993

-166.01

257

-

266

0

---

K.QGTDEGALTR.V

1108.3336

1107.3263

1107.5196

-174.53

183

-

191

0

---

K.AYNDEDLIR.I

1108.3336

1107.3263

1107.5196

-174.53

183

-

191

0

64

K.AYNDEDLIR.I

1158.5227

1157.5154

1157.7172

-174.30

148

-

157

0

---

K.LLLPLVTAYR.Y

1158.5227

1157.5154

1157.7172

-174.30

148

-

157

0

79

K.LLLPLVTAYR.Y

1235.4343

1234.4270

1234.6306

-164.86

88

-

98

1

---

R.DAFLANEATKR.W

1263.4509

1262.4436

1262.6619

-172.84

199

-

209

0

---

K.AQINATLNQYK.N

1286.6053

1285.5980

1285.8122

-166.54

147

-

157

1

---

R.KLLLPLVTAYR.Y

1466.4775

1465.4702

1465.7049

-160.09

64

-

75

1

---

K.SLDKELTNDFER.V

1516.4551

1515.4478

1515.7093

-172.50

51

-

63

0

---

R.QTYADTYGEDLLK.S

1738.4792

1737.4719

1737.7594

-165.46

132

-

146

0

---

K.SLEEDVGYHTNGDFR.K

1738.4792

1737.4719

1737.7594

-165.46

132

-

146

0

82

K.SLEEDVGYHTNGDFR.K

1805.4937

1804.4864

1804.8115

-180.13

210

-

225

0

---

K.NVYGNDIDQDLEADPK.D

1866.5486

1865.5413

1865.8544

-167.79

132

-

147

1

---

K.SLEEDVGYHTNGDFRK.L

1866.5486

1865.5413

1865.8544

-167.79

132

-

147

1

53

K.SLEEDVGYHTNGDFRK.L

2301.6650

2300.6577

2300.1682

213

2

-

22

1

---

M.STLTVPAQVPSVTEDCEQLRK.A

2490.9636

2489.9563

2490.3733

-167.45

148

-

169

1

---

K.LLLPLVTAYRYEGDEVNITLAK.S

2822.8462

2821.8389

2822.3246

-172.09

210

-

233

1

---

K.NVYGNDIDQDLEADPKDEYLDLLR.A

No match to: 712.2611, 728.2079, 734.3343, 768.3830, 840.2792, 868.3738, 882.3950, 968.3657, 978.3624, 997.3488, 1015.3862, 1030.3062, 1059.3774, 1064.3904, 1064.3904, 1121.3789, 1125.3287, 1128.3535, 1137.3715, 1249.4457, 1261.4731, 1342.5945, 1374.4985, 1449.4924, 1461.4905, 1461.4905, 1465.5010, 1475.4929, 1477.4952, 1480.5566, 1487.4999, 1499.4327, 1544.6260, 1556.4344, 1564.6271, 1721.4749, 1736.5342, 1752.5193, 1759.6407, 1777.5836, 1783.8263, 1784.2188, 1784.9736, 1785.6469, 1815.5273, 1818.5637, 1827.5225, 1834.5964, 1842.5465, 1844.5240, 1848.5405, 1849.5455, 1874.6422, 1876.6283, 1876.6283, 1892.5652, 1906.6105, 2152.7185, 2158.7742, 2158.7742, 2168.7585, 2169.7573, 2170.7656, 2170.7656, 2179.8198, 2181.7188, 2185.7793, 2186.7700, 2192.7439, 2200.7498, 2209.7502, 2271.7493, 2275.8650, 2288.6592, 2298.8257, 2307.8164, 2308.8716, 2310.4966, 2311.6814, 2323.7778, 2339.8428, 2357.7700, 2397.8184, 2399.8052, 2415.7266, 2677.8975, 3003.0210

**G36	Cs5g06840.1	Mass: 25512	Score: 467	Expect: 8.8e-043	Matches: 13**

Observed

Mr(expt)

Mr(calc)

ppm

Start

End

Miss

Ions

Peptide

916.3169

915.3096

915.4774

-183.28

185

-

193

0

---

R.GDLAVQTGR.N

916.3169

915.3096

915.4774

-183.28

185

-

193

0

50

R.GDLAVQTGR.N

943.3845

942.3772

942.5498

-183.16

98

-

106

0

---

R.GLVGEIISR.F

943.3845

942.3772

942.5498

-183.16

98

-

106

0

57

R.GLVGEIISR.F

1004.2760

1003.2687

1003.4610

-191.66

125

-

132

0

---

K.DLAEEHYK.D

1192.4211

1191.4138

1191.6288

-180.40

133

-

142

0

---

K.DLNSKPFFPK.L

1192.4211

1191.4138

1191.6288

-180.40

133

-

142

0

60

K.DLNSKPFFPK.L

1495.4719

1494.4646

1494.7175

-169.19

194

-

207

1

---

R.NVVHGSDSPENGKR.E

1495.4719

1494.4646

1494.7175

-169.19

194

-

207

1

96

R.NVVHGSDSPENGKR.E

1667.6028

1666.5955

1666.8890

-176.06

169

-

184

0

---

K.LIGSTDPLQAEPGTIR.G

1667.6028

1666.5955

1666.8890

-176.06

169

-

184

0

106

K.LIGSTDPLQAEPGTIR.G

1795.6804

1794.6731

1794.9839

-173.16

168

-

184

1

---

R.KLIGSTDPLQAEPGTIR.G

1795.6804

1794.6731

1794.9839

-173.16

168

-

184

1

76

R.KLIGSTDPLQAEPGTIR.G

No match to: 700.2636, 705.2198, 713.2774, 716.2185, 724.3438, 730.2541, 734.3520, 744.2436, 750.3709, 752.2985, 768.3785, 808.2391, 817.2513, 824.3417, 834.3267, 842.3502, 848.3605, 850.3646, 860.9109, 864.3581, 868.3756, 870.3786, 876.8808, 878.3042, 882.3943, 890.2632, 896.3150, 896.3150, 906.3101, 907.2824, 908.3121, 908.3121, 924.3116, 930.2889, 951.3475, 965.3520, 968.3380, 974.2646, 999.2775, 1017.4360, 1018.2946, 1032.3308, 1033.3540, 1042.2992, 1051.4994, 1054.3475, 1059.3882, 1062.3510, 1072.3640, 1074.3682, 1081.4084, 1086.3766, 1115.3491, 1133.4825, 1136.3107, 1151.5045, 1165.5333, 1214.3989, 1230.3824, 1272.4041, 1298.4432, 1315.4089, 1324.3926, 1334.6576, 1336.4014, 1340.4010, 1352.4071, 1448.6251, 1649.5837, 1681.6147, 1689.5729, 1705.5490, 1895.5706, 1901.5940, 1913.5890, 1929.5754, 1940.5808, 2002.6844, 2143.5662, 2206.7205, 2206.7205, 2220.7078, 2223.6611, 2238.7322, 2245.7632, 2252.7063, 2254.6506, 2255.6909, 2269.6592, 2270.6436, 2270.6436, 2284.6428, 2286.6355, 2293.6152, 2304.6340, 2308.5962, 2666.8162

**G5	Cs5g04880.1	Mass: 54916	Score: 236	Expect: 1.1e-019	Matches: 12**

Observed

Mr(expt)

Mr(calc)

ppm

Start

End

Miss

Ions

Peptide

713.2866

712.2793

712.4483

-237.15

20

-

25

1

---

R.EPVLKK.R

727.3130

726.3057

726.4388

-183.24

335

-

341

0

---

R.LGPVVSR.G

1181.4070

1180.3997

1180.5836

-155.79

236

-

247

0

---

K.IAFTGSNATGSR.I

1214.4712

1213.4639

1213.6707

-170.33

136

-

146

0

---

K.APVSLPLENFK.S

1332.5565

1331.5492

1331.7197

-128.04

335

-

346

1

---

R.LGPVVSRGQYEK.V

1332.5565

1331.5492

1331.7197

-128.04

335

-

346

1

---

R.LGPVVSRGQYEK.V

1626.6226

1625.6153

1625.8777

-161.36

302

-

315

0

---

R.LLVHESIAAEFLER.M

1626.6226

1625.6153

1625.8777

-161.36

302

-

315

0

101

R.LLVHESIAAEFLER.M

1781.5376

1780.5303

1780.8115

-157.90

405

-

420

0

---

K.AFSTEDEAIELANDTR.Y

1781.5376

1780.5303

1780.8115

-157.90

405

-

420

0

116

K.AFSTEDEAIELANDTR.Y

1882.6593

1881.6520

1882.0420

-207.20

248

-

266

0

---

R.IMAAAAPMVKPVSLELGGK.S

3069.1218

3068.1145

3068.6072

-160.55

174

-

204

0

---

K.VAPALAAGCAAILKPSELASVTCLELAEVCR.E

No match to: 700.2808, 718.2123, 724.3835, 734.3641, 735.3209, 736.3508, 750.3821, 768.3910, 782.3248, 834.3590, 836.3410, 842.3594, 848.3683, 850.3802, 860.9177, 864.3798, 868.3897, 876.8877, 882.4032, 896.2329, 948.2961, 968.3651, 982.3789, 996.4106, 1013.3716, 1017.4843, 1029.3805, 1051.5192, 1057.3268, 1059.3840, 1084.3387, 1099.3016, 1099.3016, 1149.3850, 1151.4703, 1158.4072, 1161.3904, 1165.4899, 1178.3964, 1179.4064, 1182.4082, 1187.4729, 1188.4098, 1194.3920, 1196.4071, 1261.5073, 1276.4463, 1284.3945, 1284.3945, 1296.4689, 1315.4449, 1381.5203, 1381.5203, 1434.5924, 1453.5210, 1465.5245, 1479.5126, 1509.5215, 1553.4923, 1592.5525, 1592.5525, 1619.5327, 1638.5475, 1640.5867, 1648.6024, 1680.5634, 1705.5602, 1706.5686, 1715.6355, 1727.6426, 1727.6426, 1732.6431, 1733.6211, 1742.6018, 1744.6567, 1744.6567, 1791.4510, 1795.5591, 1803.5725, 1833.5593, 1855.7125, 1917.5811, 1922.5884, 1938.5854, 1938.5854, 1952.6149, 1979.7153, 2014.6783, 2029.6769, 2085.6697, 2164.7561, 2189.7878, 2251.8337, 2254.6887, 2342.7832, 2383.5679, 2705.7014, 2885.9739

**G7	Cs8g16040.1	Mass: 61464	Score: 215	Expect: 1.4e-017	Matches: 23**

Observed

Mr(expt)

Mr(calc)

ppm

Start

End

Miss

Ions

Peptide

700.2739

699.2666

699.4643

-282.64

386

-

391

1

---

R.IAQLKK.E

716.2579

715.2507

715.4089

-221.20

18

-

23

1

---

K.QGSLRR.R

733.2368

732.2295

732.3555

-171.97

318

-

324

0

---

K.APGFGER.R

798.3062

797.2989

797.4508

-190.39

175

-

181

0

---

R.ARPIEGR.D

1043.4017

1042.3944

1042.5771

-175.23

89

-

98

0

---

K.VVNDGVTIAR.A

1090.4087

1089.4014

1089.4938

-84.79

471

-

479

1

---

K.DKLEDADER.L

1090.4087

1089.4014

1089.5818

-165.58

251

-

259

0

77

K.LIVEFENAR.V

1198.4922

1197.4849

1197.6717

-155.98

64

-

75

0

---

K.LSDAVGLTLGPR.G

1204.4125

1203.4052

1203.6136

-173.09

78

-

88

0

---

R.NVVLDEFGSPK.V

1290.4337

1289.4264

1289.6099

-142.28

421

-

432

0

---

K.VGAATETELEDR.K

1409.5264

1408.5191

1408.7674

-176.24

163

-

174

1

---

K.TVHGLVEELEKR.A

1479.4812

1478.4739

1478.7405

-180.30

238

-

250

0

---

R.GYISPQFVTNPEK.L

1485.4290

1484.4217

1484.6518

-154.99

392

-

404

0

---

K.ELAETDSVYDSEK.L

1539.4797

1538.4724

1538.9032

-279.90

272

-

284

1

---

K.DIIPLLEKTTQLR.A

1564.4891

1563.4818

1563.7603

-178.06

527

-

540

0

---

K.YENMLQAGVIDPAK.V + Oxidation (M)

1711.6884

1710.6811

1710.9992

-185.90

142

-

158

1

---

K.LGLLSVTSGANPVSLKR.G

1770.6042

1769.5969

1769.8981

-170.18

99

-

115

0

---

R.AIELADPMENAGAALIR.E + Oxidation (M)

1770.6042

1769.5969

1769.8981

-170.18

99

-

115

0

54

R.AIELADPMENAGAALIR.E + Oxidation (M)

1904.6527

1903.6454

1903.9487

-159.27

368

-

385

1

---

K.DSTTIIADAASKDEIQAR.I

2099.7417

2098.7344

2099.1045

-176.29

55

-

75

1

---

R.AAMQAGIDKLSDAVGLTLGPR.G + Oxidation (M)

2114.6738

2113.6665

2114.1219

-215.40

412

-

432

1

---

K.LSGGVAVIKVGAATETELEDR.K

2272.8550

2271.8477

2272.2427

-173.82

488

-

510

0

---

K.ALVAPASLIAHNAGVEGEVVVEK.V

2954.0549

2953.0476

2953.5661

-175.56

441

-

470

0

---

K.NATFAAIEEGIVPGGGAALVHLSDHVPAIK.D

No match to: 712.1229, 713.2859, 718.2404, 719.2248, 724.3748, 734.3671, 736.3717, 747.2726, 750.3824, 768.3898, 782.3236, 834.3646, 842.3455, 850.3798, 860.9222, 864.2708, 864.2708, 868.3862, 882.3987, 964.3394, 977.3329, 982.3872, 987.3248, 987.3248, 996.4064, 999.3276, 1005.3707, 1017.4639, 1021.3235, 1036.3712, 1051.5155, 1055.3638, 1059.3942, 1104.4075, 1125.3831, 1125.3831, 1131.5198, 1137.3844, 1137.3844, 1140.4008, 1151.5271, 1153.3962, 1163.3750, 1165.4956, 1214.4243, 1249.3966, 1255.4946, 1255.4946, 1260.4287, 1265.4646, 1315.4539, 1325.4565, 1334.5864, 1392.4252, 1395.5164, 1398.4076, 1398.4076, 1405.4425, 1410.3977, 1410.3977, 1416.5531, 1424.4160, 1426.4005, 1426.4005, 1432.3896, 1444.4388, 1460.4829, 1477.4524, 1506.4069, 1517.4912, 1534.5270, 1552.5493, 1553.5444, 1617.5482, 1735.5800, 1748.6147, 1766.6920, 1786.6158, 1869.6552, 1878.5773, 1882.6575, 1893.6422, 1982.6494, 2183.6721, 2238.7383, 2257.6340, 2305.7227

**G10	Cs8g16040.1	Mass: 61464	Score: 783	Expect: 2.2e-074	Matches: 37**

Observed

Mr(expt)

Mr(calc)

ppm

Start

End

Miss

Ions

Peptide

716.2187

715.2114

715.4089

-276.05

18

-

23

1

---

K.QGSLRR.R

731.2310

730.2237

730.3609

-187.86

380

-

385

0

---

K.DEIQAR.I

733.2185

732.2112

732.3555

-196.97

318

-

324

0

---

K.APGFGER.R

798.3038

797.2966

797.4508

-193.37

175

-

181

0

---

R.ARPIEGR.D

802.3210

801.3137

801.4596

-182.07

260

-

266

0

---

R.VLVTDQK.I

847.2408

846.2335

846.3719

-163.52

473

-

479

0

---

K.LEDADER.L

898.4000

897.3927

897.5647

-191.68

309

-

317

0

---

R.GILNVAAIK.A

920.3228

919.3155

919.4433

-139.02

55

-

63

0

---

R.AAMQAGIDK.L + Oxidation (M)

940.3966

939.3893

939.5641

-185.96

272

-

279

0

---

K.DIIPLLEK.T

1043.3998

1042.3925

1042.5771

-177.06

89

-

98

0

---

K.VVNDGVTIAR.A

1043.3998

1042.3925

1042.5771

-177.06

89

-

98

0

32

K.VVNDGVTIAR.A

1090.3846

1089.3773

1089.4938

-106.91

471

-

479

1

---

K.DKLEDADER.L

1090.3846

1089.3773

1089.5818

-187.69

251

-

259

0

80

K.LIVEFENAR.V

1196.3416

1195.3343

1195.6271

-244.88

571

-

582

0

---

K.TPVAAPPQGLMV.- + Oxidation (M)

1198.4691

1197.4618

1197.6717

-175.27

64

-

75

0

---

K.LSDAVGLTLGPR.G

1198.4691

1197.4618

1197.6717

-175.27

64

-

75

0

86

K.LSDAVGLTLGPR.G

1204.4080

1203.4007

1203.6136

-176.83

78

-

88

0

---

R.NVVLDEFGSPK.V

1253.4668

1252.4595

1252.6663

-165.07

163

-

173

0

---

K.TVHGLVEELEK.R

1290.4010

1289.3937

1289.6099

-167.64

421

-

432

0

---

K.VGAATETELEDR.K

1290.4010

1289.3937

1289.6099

-167.64

421

-

432

0

50

K.VGAATETELEDR.K

1409.5365

1408.5292

1408.7674

-169.07

163

-

174

1

---

K.TVHGLVEELEKR.A

1409.5365

1408.5292

1408.7674

-169.07

163

-

174

1

96

K.TVHGLVEELEKR.A

1418.4872

1417.4799

1417.7049

-158.67

421

-

433

1

---

K.VGAATETELEDRK.L

1479.4866

1478.4793

1478.7405

-176.65

238

-

250

0

---

R.GYISPQFVTNPEK.L

1479.4866

1478.4793

1478.7405

-176.65

238

-

250

0

77

R.GYISPQFVTNPEK.L

1485.4084

1484.4011

1484.6518

-168.86

392

-

404

0

---

K.ELAETDSVYDSEK.L

1539.4590

1538.4517

1538.9032

-293.35

272

-

284

1

---

K.DIIPLLEKTTQLR.A

1555.6118

1554.6045

1554.8981

-188.81

142

-

157

0

---

K.LGLLSVTSGANPVSLK.R

1564.5134

1563.5061

1563.7603

-162.52

527

-

540

0

---

K.YENMLQAGVIDPAK.V + Oxidation (M)

1636.5074

1635.5001

1635.7700

-165.00

121

-

137

0

---

K.TNDSAGDGTTTASVLAR.E

1770.5885

1769.5812

1769.8981

-179.05

99

-

115

0

---

R.AIELADPMENAGAALIR.E + Oxidation (M)

1770.5885

1769.5812

1769.8981

-179.05

99

-

115

0

83

R.AIELADPMENAGAALIR.E + Oxidation (M)

1904.6313

1903.6240

1903.9487

-170.51

368

-

385

1

---

K.DSTTIIADAASKDEIQAR.I

1904.6313

1903.6240

1903.9487

-170.51

368

-

385

1

126

K.DSTTIIADAASKDEIQAR.I

2272.8513

2271.8440

2272.2427

-175.44

488

-

510

0

---

K.ALVAPASLIAHNAGVEGEVVVEK.V

2290.7190

2289.7117

2290.1362

-185.36

186

-

208

0

---

K.AVATISAGNDDLIGTMIADAIDK.V + Oxidation (M)

2954.0378

2953.0305

2953.5661

-181.35

441

-

470

0

---

K.NATFAAIEEGIVPGGGAALVHLSDHVPAIK.D

No match to: 713.2830, 730.2479, 744.2673, 750.3577, 755.2446, 768.3771, 781.2563, 819.2359, 824.3093, 842.3491, 850.3591, 856.3311, 860.9039, 864.2515, 864.2515, 868.3715, 876.8828, 882.3929, 886.2358, 895.2583, 944.2961, 956.2986, 966.2819, 982.3936, 987.3071, 999.3242, 1012.2650, 1051.5157, 1059.3840, 1075.3763, 1077.3844, 1080.3977, 1104.4033, 1112.3688, 1145.3481, 1151.4923, 1158.3878, 1165.4917, 1177.4629, 1226.3989, 1231.3857, 1242.3734, 1260.4070, 1297.4600, 1315.4060, 1334.6168, 1353.4281, 1395.5208, 1398.4479, 1425.4797, 1444.5107, 1448.5646, 1465.4967, 1472.4467, 1499.4635, 1501.4697, 1517.4547, 1531.5009, 1706.5527, 1725.4684, 1752.6212, 1784.6095, 1786.5985, 1786.5985, 1800.5989, 1804.5254, 1882.6029, 2002.7074, 2032.7078, 2220.7288, 2238.7253, 2245.7964, 2294.8103

**G12	Cs7g31800.2	Mass: 41356	Score: 219	Expect: 5.6e-018	Matches: 10**

Observed

Mr(expt)

Mr(calc)

ppm

Start

End

Miss

Ions

Peptide

895.2608

894.2535

894.4083

-173.06

253

-

259

0

---

R.VYDDEVR.K

1145.3616

1144.3543

1144.5513

-172.07

279

-

288

0

---

K.EAAPTFEQPR.M

1145.3616

1144.3543

1144.5513

-172.07

279

-

288

0

42

K.EAAPTFEQPR.M

1159.3867

1158.3794

1158.6397

-224.66

261

-

272

0

---

K.WISGVGVGSIGK.S

1690.5369

1689.5296

1689.8322

-179.07

219

-

233

1

---

K.GIFRNDNVADDDIVK.L

1882.6532

1881.6459

1881.9625

-168.23

234

-

250

0

---

K.LVDTFPGQSIDFFGALR.A

1882.6532

1881.6459

1881.9625

-168.23

234

-

250

0

97

K.LVDTFPGQSIDFFGALR.A

2078.6843

2077.6770

2078.0466

-177.85

294

-

310

1

---

K.LLEYGNMIVQEQENVKR.V + Oxidation (M)

2088.8298

2087.8225

2088.1619

-162.54

179

-

197

0

---

R.VPIIVTGNDFSTLYAPLIR.D

2088.8298

2087.8225

2088.1619

-162.54

179

-

197

0

64

R.VPIIVTGNDFSTLYAPLIR.D

No match to: 713.2684, 716.2421, 734.3401, 752.2342, 768.3810, 814.1975, 842.3608, 868.3729, 882.4075, 944.2968, 956.2959, 956.2959, 963.3311, 972.2886, 994.3743, 1051.5020, 1059.3687, 1085.3618, 1085.3618, 1121.3568, 1123.3406, 1127.3640, 1142.4020, 1153.3762, 1165.3579, 1170.3796, 1252.4119, 1255.4312, 1297.4707, 1303.4691, 1331.3336, 1347.4485, 1359.4413, 1366.4258, 1373.5073, 1429.4910, 1436.4648, 1478.5861, 1487.4612, 1512.5328, 1531.5444, 1534.5465, 1546.6028, 1547.5107, 1549.4891, 1549.4891, 1566.5372, 1599.4833, 1605.4617, 1633.4670, 1635.5021, 1638.4252, 1640.4850, 1640.4850, 1642.5375, 1645.4799, 1656.4277, 1667.4594, 1677.5027, 1680.4933, 1685.4968, 1701.5861, 1702.5591, 1707.5765, 1707.5765, 1717.5497, 1719.5759, 1719.5759, 1724.5319, 1725.4762, 1729.5081, 1733.5764, 1735.5762, 1741.5175, 1742.4890, 1742.4890, 1746.6271, 1756.4908, 1772.6194, 1796.6610, 1890.6281, 1921.6068, 1938.6040, 1960.6411, 2002.7197, 2014.7263, 2026.6613, 2070.6602, 2093.7175, 2133.6204, 2172.7292, 2224.6929, 2238.7590, 2245.8081, 2378.7588, 2833.7156, 2845.7258, 2858.7664, 3346.0859, 3353.9937

**G19	Cs1g25510.1	Mass: 40452	Score: 289	Expect: 5.6e-025	Matches: 23**

Observed

Mr(expt)

Mr(calc)

ppm

Start

End

Miss

Ions

Peptide

706.2465

705.2392

705.3810

-201.00

83

-

88

0

---

K.TPYIGR.C

717.1873

716.1800

716.3203

-195.86

231

-

235

0

---

K.MFFEK.H + Oxidation (M)

733.2208

732.2135

732.3555

-193.87

270

-

275

0

---

K.APENFR.L

761.2751

760.2678

760.4079

-184.24

15

-

21

0

---

K.STSLPTR.T

807.2797

806.2724

806.4286

-193.70

276

-

282

0

---

R.LDFAVSR.E

808.2418

807.2345

807.3619

-157.79

201

-

206

0

---

K.EMLMPR.D + 2 Oxidation (M)

827.2483

826.2410

826.4007

-193.24

292

-

297

0

---

K.MYIQTR.M + Oxidation (M)

963.3049

962.2976

962.5773

-290.63

133

-

140

1

---

K.NAKPHKLR.L

990.3287

989.3215

989.4930

-173.37

268

-

275

1

---

K.EKAPENFR.L

990.3287

989.3215

989.4930

-173.37

268

-

275

1

27

K.EKAPENFR.L

1265.4691

1264.4618

1264.6088

-116.21

352

-

361

1

---

K.KAEQWNVEVY.-

1332.3705

1331.3632

1331.6736

-233.11

226

-

235

1

---

R.GFLWKMFFEK.H

1332.3705

1331.3632

1331.7119

-261.80

1

-

14

0

---

-.MAAVSAAVSLPTSK.S

1334.6489

1333.6416

1333.5795

46.6

312

-

322

0

---

K.DNTYVYMCGLR.G

1378.4779

1377.4706

1377.7140

-176.63

165

-

176

0

---

R.LVYTNENGEIVK.G

1378.4779

1377.4706

1377.7140

-176.63

165

-

176

0

60

R.LVYTNENGEIVK.G

1387.4598

1386.4525

1386.6991

-177.80

119

-

132

0

---

K.EGQSIGVIADGVDK.N

1444.4097

1443.4024

1443.7578

-246.13

194

-

206

1

---

K.ITGPVGKEMLMPR.D + Oxidation (M)

1534.5499

1533.5426

1533.8151

-177.63

164

-

176

1

---

K.RLVYTNENGEIVK.G

1630.4906

1629.4833

1629.7886

-187.31

141

-

156

0

---

R.LYSIASSALGDFGDSK.T

1630.4906

1629.4833

1629.7886

-187.31

141

-

156

0

57

R.LYSIASSALGDFGDSK.T

1960.6688

1959.6615

1960.0088

-177.18

207

-

225

0

---

R.DPNATVIMLATGTGIAPFR.G + Oxidation (M)

1960.6688

1959.6615

1960.0088

-177.18

207

-

225

0

70

R.DPNATVIMLATGTGIAPFR.G + Oxidation (M)

No match to: 700.2706, 705.2263, 713.2787, 716.2275, 720.2023, 728.2145, 730.2331, 736.3216, 739.1966, 743.2169, 744.2319, 750.3738, 768.3817, 788.2403, 818.2833, 823.2494, 824.2140, 836.2983, 842.2335, 843.2367, 848.3446, 850.3733, 850.3733, 868.3853, 882.3925, 915.2844, 919.3251, 921.2852, 968.3346, 982.3663, 996.3538, 1009.3056, 1017.4924, 1025.3049, 1040.3184, 1051.5146, 1059.3815, 1060.3750, 1078.2784, 1083.3149, 1131.4263, 1149.4019, 1151.4988, 1157.3239, 1165.5138, 1168.3632, 1179.3123, 1213.4131, 1250.4025, 1251.4114, 1255.3988, 1268.3535, 1285.3860, 1285.3860, 1297.3684, 1297.3684, 1302.3522, 1313.3821, 1314.3557, 1316.3696, 1316.3696, 1335.3654, 1364.3762, 1377.4489, 1402.4082, 1407.3634, 1412.3723, 1422.3848, 1423.3278, 1423.3278, 1429.3993, 1439.3398, 1448.6292, 1451.4576, 1453.4292, 1461.3695, 1465.4642, 1477.3800, 1497.4242, 1641.4918, 1657.4630, 1695.4209, 1713.5165, 1723.4058, 1940.5822, 1976.6636, 2238.7222

**G13	orange1.1t00475.2	Mass: 46723	Score: 507	Expect: 8.8e-047	Matches: 24**

Observed

Mr(expt)

Mr(calc)

ppm

Start

End

Miss

Ions

Peptide

701.1923

700.1850

700.3140

-184.16

239

-

245

0

---

K.DGAEGPR.G

718.2424

717.2352

717.3810

-203.26

161

-

166

0

---

R.APVEFR.Q

803.2599

802.2526

802.4072

-192.69

348

-

354

0

---

K.ELADDIK.Q

857.2883

856.2810

856.4403

-186.00

167

-

174

0

---

R.QVGSPDVR.G

863.3001

862.2928

862.4548

-187.92

323

-

329

0

---

K.LLFAEDR.K

863.3001

862.2928

862.4548

-187.92

323

-

329

0

27

K.LLFAEDR.K

961.4054

960.3981

960.5756

-184.80

179

-

186

0

---

K.RPVSIVYK.G

961.4054

960.3981

960.5756

-184.80

179

-

186

0

39

K.RPVSIVYK.G

976.3185

975.3112

975.5025

-196.15

187

-

195

0

---

K.GDDKPGFLK.K

991.3791

990.3719

990.5498

-179.63

323

-

330

1

---

K.LLFAEDRK.Q

991.3791

990.3719

990.5498

-179.63

322

-

329

1

48

K.KLLFAEDR.K

1253.3805

1252.3732

1252.5935

-175.88

381

-

392

0

---

K.ADDSPEAVPQPK.V

1386.4763

1385.4690

1385.7150

-177.54

148

-

160

0

---

K.LGDDASAQLLDIR.A

1386.4763

1385.4690

1385.7150

-177.54

148

-

160

0

130

K.LGDDASAQLLDIR.A

1563.4999

1562.4926

1562.7940

-192.87

331

-

343

0

---

K.QTLQQVDEFLNTK.V

1618.5264

1617.5191

1617.8362

-196.00

393

-

408

0

---

K.VEAAADPPPQVNSVPK.T

1618.5264

1617.5191

1617.8362

-196.00

393

-

408

0

42

K.VEAAADPPPQVNSVPK.T

1626.4780

1625.4707

1625.8388

-226.40

246

-

259

1

---

R.GWMNSGLPWIPPKK.A + Oxidation (M)

1691.5909

1690.5836

1690.8890

-180.60

330

-

343

1

---

R.KQTLQQVDEFLNTK.V

1691.5909

1690.5836

1690.8890

-180.60

330

-

343

1

67

R.KQTLQQVDEFLNTK.V

1694.5767

1693.5694

1693.8927

-190.83

201

-

214

1

---

K.FKEPENTTLFILDK.F

1952.6361

1951.6288

1951.9891

-184.57

215

-

232

0

---

K.FDGNSELVAELVTINGFK.N

2339.7368

2338.7295

2339.1685

-187.67

422

-

443

0

---

R.SLSPYPAYPDLKPPTSPTPSQP.-

2339.7368

2338.7295

2339.1685

-187.67

422

-

443

0

77

R.SLSPYPAYPDLKPPTSPTPSQP.-

No match to: 700.2639, 705.2549, 713.2765, 716.2350, 724.3577, 730.2481, 732.2554, 734.3388, 736.3046, 738.2609, 740.2270, 743.2256, 750.3654, 752.2514, 768.3773, 772.2490, 834.3333, 840.2606, 842.3470, 850.3674, 860.9117, 868.3783, 877.3016, 882.3908, 895.2550, 895.2550, 899.3832, 907.2543, 907.2543, 921.2610, 923.2574, 929.2533, 934.3309, 944.2871, 950.2648, 956.2862, 982.3786, 987.3187, 996.3968, 1007.4456, 1008.3046, 1045.3601, 1051.5061, 1057.4070, 1059.3845, 1062.3832, 1092.3253, 1099.3689, 1116.3308, 1118.3588, 1126.3346, 1145.3558, 1151.5000, 1155.3688, 1165.5240, 1168.4313, 1193.3849, 1225.3854, 1242.4470, 1256.4204, 1315.4171, 1334.6322, 1337.4750, 1353.4364, 1361.3768, 1377.4348, 1408.4434, 1416.4338, 1424.4459, 1434.5474, 1530.4612, 1546.4894, 1546.4894, 1557.4915, 1568.4758, 1640.5171, 1649.5775, 1707.4722, 1713.5588, 1725.4633, 1882.6345, 1992.6482, 2002.6804, 2238.7253, 2245.7986, 2361.7292

**G16	Cs3g01420.1	Mass: 32572	Score: 321	Expect: 3.5e-028	Matches: 12**

Observed

Mr(expt)

Mr(calc)

ppm

Start

End

Miss

Ions

Peptide

833.2992

832.2919

832.4477

-187.10

182

-

188

0

---

K.VLMDALR.V + Oxidation (M)

834.2952

833.2880

833.4395

-181.89

237

-

243

0

---

K.GFELVNR.E

962.3669

961.3596

961.5345

-181.89

236

-

243

1

---

K.KGFELVNR.E

962.3669

961.3596

961.5345

-181.89

236

-

243

1

26

K.KGFELVNR.E

1137.3561

1136.3488

1136.5462

-173.65

204

-

213

0

---

K.EANSESFVVR.T

1137.3561

1136.3488

1136.5462

-173.65

204

-

213

0

95

K.EANSESFVVR.T

1342.4890

1341.4817

1341.7140

-173.11

274

-

285

0

---

K.VTTPDDLLIAER.I

1342.4890

1341.4817

1341.7140

-173.11

274

-

285

0

94

K.VTTPDDLLIAER.I

1621.5374

1620.5301

1620.8148

-175.61

260

-

273

0

---

K.HPVYITEGSYTNIK.V

1621.5374

1620.5301

1620.8148

-175.61

260

-

273

0

85

K.HPVYITEGSYTNIK.V

1782.5771

1781.5698

1781.9047

-187.94

244

-

259

0

---

R.EGLEVTDDVSIVEHLK.H

2072.6987

2071.6914

2071.9660

-132.51

113

-

130

0

---

K.EIVVVCDPSYSDIFEETK.E

No match to: 700.2760, 707.2309, 713.2799, 716.2305, 718.2790, 724.3565, 730.2553, 734.3534, 736.3542, 738.2530, 744.2589, 750.3721, 752.2745, 766.2789, 768.3773, 797.2555, 809.2018, 823.2485, 824.2433, 836.3052, 842.3527, 848.3306, 850.3683, 860.9131, 863.2739, 864.3542, 868.3655, 876.8796, 882.3938, 917.2946, 937.2870, 943.3040, 954.3421, 996.3950, 1017.4672, 1019.4868, 1051.5104, 1059.3821, 1090.4164, 1099.3789, 1119.3568, 1126.3589, 1128.3551, 1131.4467, 1133.4908, 1151.4808, 1156.3497, 1165.5009, 1172.4707, 1172.4707, 1213.4535, 1263.4377, 1263.4377, 1265.4750, 1298.4292, 1312.4669, 1315.4165, 1334.6316, 1340.4130, 1353.4539, 1361.4857, 1392.4612, 1392.4612, 1414.4485, 1430.5363, 1434.5994, 1444.4872, 1448.6007, 1458.4941, 1480.5420, 1517.4401, 1517.4401, 1533.4336, 1559.4810, 1603.5470, 1603.5470, 1617.6890, 1620.5692, 1633.5504, 1635.5500, 1643.5367, 1659.5146, 1665.5138, 1669.5735, 1677.5533, 1718.5499, 1722.5366, 1722.5366, 1744.5433, 1809.6219, 1937.7053, 2002.7051, 2059.7603, 2071.7314, 2087.7432, 2220.7646, 2238.7483, 2245.8054

**G17	orange1.1t02542.1	Mass: 35354	Score: 454	Expect: 1.8e-041	Matches: 15**

Observed

Mr(expt)

Mr(calc)

ppm

Start

End

Miss

Ions

Peptide

868.3709

867.3636

867.4338

-80.93

92

-

99

0

---

K.YTADLSAK.F

1022.3524

1021.3451

1021.5192

-170.45

249

-

257

0

---

K.DSPKPPPER.I

1293.3940

1292.3867

1292.6765

-224.14

239

-

248

1

---

K.ENEKWVTFIK.D

1293.3940

1292.3867

1292.6765

-224.14

239

-

248

1

---

K.ENEKWVTFIK.D

1377.4655

1376.4582

1376.7300

-197.38

200

-

213

0

---

K.SNILATSAATGFPK.F

1416.4570

1415.4497

1415.6681

-154.26

146

-

157

1

---

R.VVPKDHDDSNYK.L

1707.6493

1706.6420

1706.9203

-163.04

105

-

121

0

---

K.GSFTVVLSGGSLIDSLR.K

1707.6493

1706.6420

1706.9203

-163.04

105

-

121

0

133

K.GSFTVVLSGGSLIDSLR.K

1835.7321

1834.7248

1835.0153

-158.28

105

-

122

1

---

K.GSFTVVLSGGSLIDSLRK.L

1835.7321

1834.7248

1835.0153

-158.28

105

-

122

1

100

K.GSFTVVLSGGSLIDSLRK.L

1863.6180

1862.6107

1862.9262

-169.32

75

-

91

0

---

K.NVQVFDSEEDLAVSLAK.Y

1863.6180

1862.6107

1862.9262

-169.32

75

-

91

0

67

K.NVQVFDSEEDLAVSLAK.Y

1964.7596

1963.7523

1964.0579

-155.56

103

-

121

1

---

K.EKGSFTVVLSGGSLIDSLR.K

1964.7596

1963.7523

1964.0579

-155.56

103

-

121

1

117

K.EKGSFTVVLSGGSLIDSLR.K

2707.9075

2706.9002

2707.3866

-179.65

214

-

238

0

---

K.FDLMLLGMGPDGHIASLFPGHPLLK.E + 2 Oxidation (M)

No match to: 700.2577, 712.1247, 713.2888, 716.2328, 718.1935, 720.1879, 734.3641, 736.3738, 750.3810, 768.3930, 797.3093, 809.3114, 842.3633, 848.3756, 850.3873, 864.3613, 882.4027, 999.3306, 1051.5215, 1059.4001, 1145.3909, 1165.4750, 1271.3971, 1276.3833, 1277.4076, 1281.3997, 1281.3997, 1287.3821, 1288.3975, 1289.4037, 1291.3948, 1292.3881, 1299.3948, 1301.3988, 1302.4155, 1304.3879, 1305.3928, 1305.3928, 1308.4108, 1309.4065, 1312.4227, 1317.4048, 1319.4069, 1321.3912, 1321.3912, 1325.4099, 1327.4066, 1330.4009, 1331.4176, 1333.4036, 1337.3763, 1340.3918, 1343.3798, 1359.4025, 1371.4524, 1388.4381, 1392.4110, 1399.4165, 1403.3885, 1410.4058, 1410.4058, 1424.4253, 1424.4253, 1433.4309, 1448.4277, 1459.4573, 1506.4525, 1617.5580, 1635.5717, 1649.5696, 1661.5756, 1677.5659, 1689.6530, 1699.5355, 1728.6625, 1729.6368, 1771.6223, 1777.6169, 1789.6393, 1799.6571, 1800.6836, 1812.6788, 1825.6213, 1840.6821, 1845.6659, 1852.6974, 1857.6906, 1885.6265, 1951.6382, 1954.7014, 2004.8013, 2092.8328, 2237.8071, 2277.7944, 2643.9707

**G18	orange1.1t02644.1	Mass: 61710	Score: 342	Expect: 2.8e-030	Matches: 19**

Observed

Mr(expt)

Mr(calc)

ppm

Start

End

Miss

Ions

Peptide

730.2338

729.2265

729.2639

-51.37

589

-

594

0

---

K.DMYQAS.- + Oxidation (M)

785.2905

784.2832

784.4079

-159.00

331

-

337

0

---

K.HLDATTK.A

891.3461

890.3389

890.4134

-83.69

401

-

407

0

---

R.LNEWDSK.V

914.2443

913.2370

913.4188

-199.08

549

-

556

0

---

K.HMQAQAGR.S + Oxidation (M)

915.2585

914.2512

914.4246

-189.65

498

-

506

0

---

K.YGGATAGYR.T

915.2585

914.2512

914.4246

-189.65

498

-

506

0

52

K.YGGATAGYR.T

1045.3746

1044.3673

1044.5815

-205.03

420

-

429

1

---

R.GATAILEDKK.K

1161.4202

1160.4129

1160.6189

-177.51

120

-

130

0

---

R.LNFGLAAEQAK.S

1352.4115

1351.4042

1351.6628

-191.32

354

-

365

0

---

K.IPVPMPPSHSMK.S + 2 Oxidation (M)

1634.5906

1633.5833

1633.8675

-173.94

249

-

262

0

---

K.QILSTETNYVPITR.G

1634.5906

1633.5833

1633.8675

-173.94

249

-

262

0

61

K.QILSTETNYVPITR.G

1645.6149

1644.6076

1644.8947

-174.54

288

-

302

0

---

K.AVPNLQLEHGLAVER.V

1645.6149

1644.6076

1644.8947

-174.54

288

-

302

0

82

K.AVPNLQLEHGLAVER.V

1725.5785

1724.5712

1724.8733

-175.13

120

-

135

1

---

R.LNFGLAAEQAKSEGYK.V

1727.6173

1726.6100

1726.9287

-184.55

507

-

522

0

---

R.TMLDALIPAAAVLQER.L + Oxidation (M)

1727.6173

1726.6100

1726.9287

-184.55

507

-

522

0

16

R.TMLDALIPAAAVLQER.L + Oxidation (M)

1740.5673

1739.5600

1739.9417

-219.37

168

-

187

0

---

K.IAGAAAAAGLSLADVAAEAK.R

2095.6614

2094.6541

2095.0222

-175.67

431

-

449

0

---

K.YYPLNDAAETVNEIGASIR.R

2095.6614

2094.6541

2095.0222

-175.67

431

-

449

0

95

K.YYPLNDAAETVNEIGASIR.R

No match to: 700.2607, 705.2355, 711.2388, 713.2690, 716.2242, 724.2744, 734.3215, 736.2856, 750.3837, 752.2648, 768.3757, 776.2624, 777.3000, 778.2907, 823.2609, 834.3058, 842.3448, 848.3488, 850.3600, 860.8984, 866.2808, 868.3755, 876.8778, 882.3833, 907.2569, 919.3688, 928.2754, 1015.3675, 1017.4698, 1051.5021, 1059.3677, 1106.3347, 1113.4268, 1126.3531, 1131.4005, 1144.4290, 1144.4290, 1151.4863, 1165.5132, 1167.4166, 1178.3435, 1215.3572, 1232.3527, 1261.4709, 1264.4628, 1275.3784, 1295.4187, 1307.4087, 1315.4312, 1323.4139, 1329.3947, 1330.4767, 1334.6370, 1335.3640, 1337.4048, 1344.4574, 1364.4790, 1448.5472, 1465.5052, 1467.4893, 1479.5042, 1499.4838, 1600.5802, 1603.5162, 1607.5114, 1617.5695, 1617.5695, 1658.6125, 1662.6260, 1673.5353, 1701.5779, 1713.5820, 1713.5820, 1729.6052, 1743.6061, 1765.5490, 1791.5187, 1795.6154, 1905.6306, 1910.5313, 1926.7478, 1926.7478, 2002.6820, 2069.6680, 2069.6680, 2238.7275, 2245.7949, 2267.7261, 2330.6597, 2474.6353, 2585.8359

**G9	Cs5g22920.1	Mass: 35083	Score: 718	Expect: 7e-068	Matches: 22**

Observed

Mr(expt)

Mr(calc)

ppm

Start

End

Miss

Ions

Peptide

908.2256

907.2184

907.3898

-188.94

112

-

117

0

---

R.EFMFYR.N + Oxidation (M)

908.2256

907.2184

907.3898

-188.94

112

-

117

0

30

R.EFMFYR.N + Oxidation (M)

991.4175

990.4103

990.5862

-177.62

98

-

106

0

---

R.TALAFVTLR.A

991.4175

990.4103

990.5862

-177.62

98

-

106

0

70

R.TALAFVTLR.A

1310.4521

1309.4448

1309.7176

-208.30

233

-

244

0

---

K.LLLVTLGEHGCR.Y

1310.4521

1309.4448

1309.7176

-208.30

233

-

244

0

20

K.LLLVTLGEHGCR.Y

1334.5248

1333.5175

1333.7466

-171.76

42

-

56

0

---

K.APGGAPANVAIAVAR.L

1334.5248

1333.5175

1333.7466

-171.76

42

-

56

0

124

K.APGGAPANVAIAVAR.L

1418.4841

1417.4768

1417.7201

-171.60

166

-

178

0

---

K.EAGALLSYDPNLR.L

1418.4841

1417.4768

1417.7201

-171.60

166

-

178

0

110

K.EAGALLSYDPNLR.L

1514.4813

1513.4740

1513.7260

-166.47

279

-

291

0

---

K.LVDDQSVLEDEPR.L

1514.4813

1513.4740

1513.7260

-166.47

279

-

291

0

108

K.LVDDQSVLEDEPR.L

1538.4913

1537.4840

1537.7512

-173.73

204

-

217

0

---

K.VSDVELEFLTGSDK.I

1631.5031

1630.4958

1630.8025

-188.05

67

-

81

0

---

K.LGDDEFGHMLAGILK.E + Oxidation (M)

1631.5031

1630.4958

1630.8025

-188.05

67

-

81

0

27

K.LGDDEFGHMLAGILK.E + Oxidation (M)

1649.4703

1648.4630

1648.7441

-170.49

82

-

97

0

---

K.ENGVSADGINFDQGAR.T

1649.4703

1648.4630

1648.7441

-170.49

82

-

97

0

137

K.ENGVSADGINFDQGAR.T

1736.6544

1735.6471

1735.9720

-187.12

311

-

327

0

---

K.GAIPALPTESEALNLLK.G

1864.7101

1863.7028

1864.0305

-175.81

311

-

329

1

---

K.GAIPALPTESEALNLLKGA.-

2224.7742

2223.7669

2224.1521

-173.19

118

-

136

0

---

R.NPSADMLLRPDELNLELIR.S + Oxidation (M)

2224.7742

2223.7669

2224.1521

-173.19

118

-

136

0

35

R.NPSADMLLRPDELNLELIR.S + Oxidation (M)

2602.8757

2601.8684

2602.3028

-166.91

252

-

278

0

---

R.GAVDAFHVNTVDTTGAGDAFVGALLGK.L

No match to: 705.2572, 713.2651, 716.2553, 718.2530, 724.2889, 734.3297, 736.2927, 768.3716, 816.2971, 826.2809, 842.3516, 844.2564, 850.3716, 860.9196, 864.3233, 868.3602, 876.8862, 882.3866, 890.2260, 907.2247, 924.2315, 930.2258, 987.3185, 1016.3519, 1051.5060, 1059.3832, 1060.3508, 1072.3999, 1117.3945, 1144.4403, 1163.3936, 1181.3486, 1211.5059, 1215.3706, 1276.5323, 1298.4558, 1298.4558, 1307.4412, 1314.4570, 1324.4498, 1326.4576, 1330.5029, 1332.4526, 1340.4550, 1348.4514, 1367.5212, 1380.4438, 1383.4617, 1386.4585, 1400.4927, 1410.4143, 1417.4781, 1440.4613, 1462.4467, 1512.4392, 1527.4833, 1528.4742, 1536.4830, 1560.4708, 1606.4889, 1623.5017, 1632.4816, 1648.4764, 1654.4819, 1661.5099, 1669.4926, 1671.4786, 1684.6292, 1685.6404, 1734.6292, 1753.5643, 1773.5601, 1775.5822, 1776.6140, 1778.7031, 1791.5378, 1792.5581, 1910.5483, 1913.6359, 1933.6589, 1953.6868, 2069.7068, 2160.8333, 2206.8347, 2238.7588, 2240.7688, 2245.7651, 2436.8235

**G33	Cs3g11320.1	Mass: 33497	Score: 279	Expect: 5.6e-024	Matches: 15**

Observed

Mr(expt)

Mr(calc)

ppm

Start

End

Miss

Ions

Peptide

910.3376

909.3303

909.5032

-190.09

28

-

35

0

---

K.DALVSHLR.S

959.3428

958.3356

958.5083

-180.27

211

-

219

0

---

R.ESPTSAIVR.F

1138.4105

1137.4032

1137.5410

-121.09

162

-

171

0

---

K.SMTEMPLIGK.N + 2 Oxidation (M)

1291.4655

1290.4582

1290.6819

-173.34

15

-

27

0

---

K.IIAGADSFGAELK.D

1291.4655

1290.4582

1290.6819

-173.34

15

-

27

0

64

K.IIAGADSFGAELK.D

1368.4054

1367.3981

1367.6285

-168.46

275

-

284

0

---

K.YYEETEFFIK.W

1368.4054

1367.3981

1367.6285

-168.46

275

-

284

0

12

K.YYEETEFFIK.W

1391.3854

1390.3781

1390.7027

-233.37

1

-

14

0

---

-.MAENAAAAAPHPLK.I

1407.4274

1406.4201

1406.6976

-197.24

1

-

14

0

---

-.MAENAAAAAPHPLK.I + Oxidation (M)

2017.6378

2016.6305

2016.8914

-129.37

175

-

193

1

---

K.LDSDSSSSTCSICCLVKNR.E

2022.6532

2021.6459

2021.9847

-167.56

255

-

272

0

---

R.FDLTVGDYLFTPAGDVHR.V

2022.6532

2021.6459

2021.9847

-167.56

255

-

272

0

104

R.FDLTVGDYLFTPAGDVHR.V

2335.7681

2334.7608

2335.1822

-180.44

222

-

243

0

---

K.AGSVEPAHHHTFGHDLVVLQGK.K

2335.7681

2334.7608

2335.1822

-180.44

222

-

243

0

62

K.AGSVEPAHHHTFGHDLVVLQGK.K

2472.7432

2471.7359

2472.1656

-173.81

36

-

58

0

---

R.SLNIDVEDLGTSDYYSIGAEVGR.R

No match to: 700.2437, 702.2580, 713.2776, 716.2172, 724.2935, 734.3440, 736.3585, 750.3613, 759.2291, 761.1887, 768.3776, 783.2504, 830.2931, 834.3552, 842.3524, 848.3290, 850.3640, 856.3309, 868.3787, 882.3892, 996.3987, 1051.4980, 1055.3260, 1057.4290, 1059.3827, 1132.3257, 1132.3257, 1145.5059, 1151.5237, 1154.3181, 1165.5139, 1170.2958, 1241.5242, 1267.4918, 1294.5037, 1299.4680, 1300.5172, 1302.4597, 1309.4398, 1311.3973, 1315.4314, 1334.4696, 1335.5134, 1352.4453, 1353.4680, 1353.4680, 1367.4857, 1374.4153, 1375.3727, 1375.3727, 1383.5582, 1390.3871, 1397.3506, 1411.3826, 1419.4757, 1435.5021, 1481.5439, 1485.4355, 1494.6064, 1496.4934, 1529.4727, 1542.5154, 1547.4811, 1547.4811, 1559.4861, 1559.4861, 1562.5127, 1568.4797, 1569.4808, 1575.4946, 1581.4863, 1584.4780, 1585.4642, 1586.4860, 1586.4860, 1607.4757, 1608.4745, 1623.4720, 1624.4464, 1648.4413, 1653.4211, 1666.4219, 1673.5131, 1683.6787, 1926.7067, 1927.6591, 1940.5850, 2002.6937, 2034.5621, 2068.7993, 2103.4805, 2238.7544, 2245.8252, 2306.5063, 2357.7317

**G26	Cs7g16620.3	Mass: 63790	Score: 344	Expect: 1.8e-030	Matches: 21**

Observed

Mr(expt)

Mr(calc)

ppm

Start

End

Miss

Ions

Peptide

716.2550

715.2477

715.4228

-244.73

396

-

402

1

---

K.IEAAGKK.K

730.2540

729.2467

729.4385

-262.92

152

-

158

1

---

K.DGGIIKK.I

817.2847

816.2774

816.4341

-191.97

474

-

480

0

---

K.VLDLDSR.N

850.3723

849.3650

849.4345

-81.74

78

-

84

1

---

R.DRSTPFK.F

858.3259

857.3186

857.4607

-165.69

33

-

40

0

---

K.EIGNQGLK.K

993.2835

992.2762

992.4426

-167.67

539

-

546

0

---

K.FYGNMFAK.M + Oxidation (M)

1042.3417

1041.3344

1041.5091

-167.68

508

-

516

0

---

K.ALEIDPDNR.D

1076.4316

1075.4243

1075.6026

-165.75

85

-

94

0

---

K.FTLGQGNVIK.G

1389.4293

1388.4220

1388.6420

-158.38

322

-

333

0

---

K.TDEEQVIDGLDR.A

1389.4293

1388.4220

1388.6420

-158.38

322

-

333

0

56

K.TDEEQVIDGLDR.A

1435.4630

1434.4557

1434.6991

-169.60

171

-

182

0

---

K.DLDEVLVNYEAR.L

1435.4630

1434.4557

1434.6991

-169.60

171

-

182

0

81

K.DLDEVLVNYEAR.L

1479.4386

1478.4313

1478.6678

-159.89

310

-

321

0

---

K.GHSEEEQLFEFK.T

1479.4386

1478.4313

1478.6678

-159.89

310

-

321

0

63

K.GHSEEEQLFEFK.T

1548.6150

1547.6077

1547.8712

-170.19

221

-

234

0

---

K.VLLAVKPQYGFGEK.G

1548.6150

1547.6077

1547.8712

-170.19

221

-

234

0

69

K.VLLAVKPQYGFGEK.G

1595.4235

1594.4162

1594.6675

-157.56

428

-

440

0

---

K.YIEYDTSFGDEEK.K

1607.5071

1606.4998

1606.7627

-163.61

309

-

321

1

---

K.KGHSEEEQLFEFK.T

1629.4803

1628.4730

1628.7174

-150.05

547

-

561

1

---

K.MSMFGSAESNKAEPK.E + Oxidation (M)

1723.4882

1722.4809

1722.7624

-163.41

428

-

441

1

---

K.YIEYDTSFGDEEKK.Q

1723.4882

1722.4809

1722.7624

-163.41

428

-

441

1

45

K.YIEYDTSFGDEEKK.Q

No match to: 700.2836, 705.2487, 713.2893, 724.2870, 733.2599, 734.3605, 736.3695, 738.2682, 744.2639, 750.3787, 768.3768, 796.3329, 804.2846, 834.3391, 842.3580, 848.3700, 864.3558, 868.3895, 882.3882, 918.2862, 935.3091, 958.3588, 968.3693, 1017.3170, 1017.4957, 1031.3522, 1051.5211, 1059.3882, 1080.3580, 1085.3851, 1117.4124, 1126.3888, 1131.4729, 1132.3749, 1151.5015, 1153.4191, 1165.5071, 1169.4464, 1173.4457, 1197.4656, 1206.5197, 1228.4310, 1257.3839, 1265.4684, 1289.4924, 1301.5516, 1329.3976, 1329.3976, 1334.6334, 1342.4093, 1345.4054, 1358.4077, 1373.4896, 1376.4181, 1412.5111, 1449.5127, 1457.4867, 1465.4996, 1473.4523, 1487.4637, 1487.4637, 1499.4403, 1500.4843, 1556.4862, 1557.5099, 1560.5801, 1570.5918, 1576.5729, 1585.5138, 1596.4829, 1597.4922, 1603.5107, 1603.5107, 1617.5283, 1625.5579, 1660.5472, 1665.5631, 1675.4683, 1680.5621, 1680.5621, 2002.7118, 2080.7095, 2112.6267, 2181.8022, 2220.7512, 2238.7598, 2238.7598, 2245.8301, 3555.9128

**G21	Cs2g28260.1	Mass: 47994	Score: 231	Expect: 3.5e-019	Matches: 18**

Observed

Mr(expt)

Mr(calc)

ppm

Start

End

Miss

Ions

Peptide

741.2817

740.2745

740.4181

-193.96

102

-

108

0

---

K.DPGALLR.Y

799.2753

798.2681

798.4236

-194.76

139

-

145

0

---

K.ALDPVER.N

856.3444

855.3371

855.4814

-168.63

197

-

204

1

---

R.DAVAPKQK.E

933.3337

932.3265

932.4967

-182.56

109

-

116

0

---

R.YALPIDNK.A

1311.3583

1310.3510

1310.5714

-168.13

281

-

290

0

---

R.HFYDGMEIQR.A + Oxidation (M)

1311.3583

1310.3510

1310.5714

-168.13

281

-

290

0

26

R.HFYDGMEIQR.A + Oxidation (M)

1374.4730

1373.4657

1373.7112

-178.69

318

-

329

0

---

R.TIPLEIMVEGEK.S + Oxidation (M)

1442.4669

1441.4596

1441.6846

-156.06

349

-

361

0

---

K.LPFNAFGTMAMAR.D + Oxidation (M)

1458.4283

1457.4210

1457.6795

-177.34

349

-

361

0

---

K.LPFNAFGTMAMAR.D + 2 Oxidation (M)

1458.4283

1457.4210

1457.6795

-177.34

349

-

361

0

8

K.LPFNAFGTMAMAR.D + 2 Oxidation (M)

1614.5825

1613.5752

1613.8512

-171.00

120

-

133

0

---

R.EVQKPLEDITDSLK.I

1615.5334

1614.5261

1614.7849

-160.25

379

-

393

0

---

K.ESELTPSNANILDGR.Y

1615.5334

1614.5261

1614.7849

-160.25

379

-

393

0

78

K.ESELTPSNANILDGR.Y

1687.5581

1686.5508

1686.8505

-177.63

330

-

344

0

---

K.SPFYGATLEELGLYK.A

1761.5295

1760.5222

1760.8251

-171.98

180

-

194

0

---

K.LEAGMDELQQIVEDR.D + Oxidation (M)

1761.5295

1760.5222

1760.8251

-171.98

180

-

194

0

80

K.LEAGMDELQQIVEDR.D + Oxidation (M)

2261.7739

2260.7666

2261.1692

-178.04

260

-

280

0

---

R.IVLDGYNAPVTAGNFVDLVER.H

2562.7222

2561.7149

2562.1762

-180.04

291

-

315

0

---

R.ADGFVVQTGDPEGPAEGFIDPSTEK.T

No match to: 700.2732, 705.2539, 709.1780, 713.2792, 716.2357, 720.2275, 724.3592, 727.2435, 730.2440, 734.3525, 736.3644, 738.2206, 744.2607, 750.3787, 752.2302, 768.3810, 782.3211, 797.2047, 834.3474, 836.3652, 842.3511, 848.3671, 850.3818, 860.9135, 864.3754, 868.3872, 876.8837, 882.3991, 948.2862, 968.3787, 973.3612, 982.4026, 996.4164, 1017.5000, 1019.5108, 1033.5100, 1037.3237, 1045.3804, 1051.5221, 1059.3933, 1059.3933, 1074.3442, 1099.3660, 1126.3523, 1133.5166, 1140.3734, 1151.5231, 1165.5269, 1165.5269, 1179.4005, 1179.4005, 1232.3967, 1235.3357, 1247.3716, 1253.4269, 1265.4707, 1279.5293, 1287.4333, 1293.4178, 1293.4178, 1308.4497, 1315.4135, 1323.4283, 1325.4092, 1327.3588, 1332.3533, 1334.6530, 1343.3445, 1383.4567, 1424.4972, 1434.6310, 1448.6477, 1464.4823, 1474.4236, 1479.4965, 1500.4833, 1597.5251, 1601.5164, 1607.4214, 1645.7279, 1645.7279, 1697.5354, 1717.7017, 1775.5037, 1777.5389, 1791.4513, 2002.7051, 2220.7415, 2238.7480, 2238.7480, 2245.8162, 2260.7139

**G30	orange1.1t02270.1	Mass: 64142	Score: 261	Expect: 3.5e-022	Matches: 16**

Observed

Mr(expt)

Mr(calc)

ppm

Start

End

Miss

Ions

Peptide

744.2564

743.2491

743.3813

-177.94

257

-

263

1

---

K.DAPADKK.A

789.2225

788.2153

788.3664

-191.75

404

-

409

0

---

K.DQIEER.M

893.2684

892.2611

892.4250

-183.68

271

-

279

0

---

K.SEATSGSVR.D

893.2684

892.2611

892.4250

-183.68

271

-

279

0

20

K.SEATSGSVR.D

901.3300

900.3227

900.4916

-187.59

321

-

328

0

---

K.ELAEQIAK.D

917.2912

916.2839

916.4614

-193.63

403

-

409

1

---

K.KDQIEER.M

936.3074

935.3001

935.3468

-49.95

502

-

509

0

---

K.DEEDSEGR.T

1053.4338

1052.4265

1052.5614

-128.19

583

-

591

0

---

K.LNSQHDVLK.L

1133.4435

1132.4362

1132.4753

-34.54

228

-

237

0

---

K.NCQHASQMAK.G + Oxidation (M)

1293.4015

1292.3942

1292.6262

-179.43

542

-

553

0

---

K.NTALHYAAGYGR.K

1293.4015

1292.3942

1292.6262

-179.43

542

-

553

0

110

K.NTALHYAAGYGR.K

1747.4774

1746.4701

1746.7656

-169.16

493

-

509

1

---

K.NALAAGADKDEEDSEGR.T

1747.4774

1746.4701

1746.7656

-169.16

493

-

509

1

99

K.NALAAGADKDEEDSEGR.T

2375.6699

2374.6626

2375.1236

-194.08

415

-

436

0

---

K.EDPSLKPILDEIESGGPSAMMK.Y + 2 Oxidation (M)

2562.7012

2561.6939

2562.1764

-188.30

380

-

402

0

---

R.LGNALMQDPSMSHMLESLTNPTK.K + 3 Oxidation (M)

2616.7632

2615.7559

2616.3026

-208.95

413

-

436

1

---

K.IKEDPSLKPILDEIESGGPSAMMK.Y + 2 Oxidation (M)

No match to: 700.2610, 702.2298, 705.2632, 707.2354, 709.2148, 713.2781, 715.2764, 716.2407, 724.3572, 730.2418, 734.3500, 736.3601, 741.2261, 750.3786, 752.2852, 757.2159, 768.3761, 782.3008, 801.2773, 812.2508, 834.3209, 842.3479, 848.3500, 850.3675, 850.3675, 860.9150, 864.3554, 868.3772, 882.3908, 968.3676, 982.3808, 996.4149, 1017.4785, 1019.4859, 1033.4994, 1035.4292, 1045.3726, 1051.5027, 1059.3721, 1080.3385, 1126.3569, 1151.5145, 1165.5233, 1205.4260, 1232.3940, 1250.3533, 1253.4111, 1270.4285, 1276.3781, 1311.3644, 1315.4037, 1324.4152, 1324.4152, 1331.3752, 1334.6302, 1337.4032, 1340.4111, 1346.4105, 1350.4106, 1361.4412, 1361.4412, 1393.4349, 1399.4089, 1424.4561, 1428.4076, 1434.6030, 1443.4219, 1448.5846, 1450.4215, 1452.4331, 1458.4276, 1468.3811, 1474.4294, 1508.4355, 1508.4355, 1524.4230, 1530.4465, 1552.4186, 1601.4760, 1615.5217, 1645.7102, 1761.5107, 1761.5107, 1873.5621, 1873.5621, 1889.5760, 1940.5581, 2002.6785, 2054.6973, 2220.7300, 2238.7305, 2238.7305, 2245.7891, 2261.7478

**G31	orange1.1t02270.1	Mass: 64142	Score: 228	Expect: 7e-019	Matches: 13**

Observed

Mr(expt)

Mr(calc)

ppm

Start

End

Miss

Ions

Peptide

725.2192

724.2120

724.3180

-146.43

437

-

441

0

---

K.YWNDK.D

747.2034

746.1962

746.3633

-223.90

250

-

256

0

---

R.MASPPTK.D + Oxidation (M)

789.2158

788.2085

788.3664

-200.26

404

-

409

0

---

K.DQIEER.M

893.2659

892.2587

892.4250

-186.42

271

-

279

0

---

K.SEATSGSVR.D

901.2859

900.2786

900.4916

-236.53

321

-

328

0

---

K.ELAEQIAK.D

917.2804

916.2731

916.4614

-205.42

403

-

409

1

---

K.KDQIEER.M

1053.3994

1052.3921

1052.5614

-160.87

583

-

591

0

---

K.LNSQHDVLK.L

1133.4854

1132.4781

1132.4753

2.46

228

-

237

0

---

K.NCQHASQMAK.G + Oxidation (M)

1293.4133

1292.4060

1292.6262

-170.30

542

-

553

0

---

K.NTALHYAAGYGR.K

1293.4133

1292.4060

1292.6262

-170.30

542

-

553

0

120

K.NTALHYAAGYGR.K

1747.4784

1746.4711

1746.7656

-168.59

493

-

509

1

---

K.NALAAGADKDEEDSEGR.T

1747.4784

1746.4711

1746.7656

-168.59

493

-

509

1

84

K.NALAAGADKDEEDSEGR.T

2375.6714

2374.6641

2375.1236

-193.45

415

-

436

0

---

K.EDPSLKPILDEIESGGPSAMMK.Y + 2 Oxidation (M)

No match to: 700.2405, 711.2048, 713.2641, 716.2208, 719.1986, 722.1701, 730.2087, 734.3451, 735.1886, 739.2263, 740.2201, 745.2122, 750.3498, 751.2816, 753.2571, 762.2017, 768.3782, 773.1928, 779.2115, 797.2233, 810.2872, 838.2718, 842.3466, 848.3204, 850.3827, 868.3701, 882.3925, 924.2654, 982.3931, 991.2980, 996.3818, 1007.4313, 1014.2742, 1017.4886, 1033.4984, 1045.3969, 1051.5157, 1059.3837, 1062.3376, 1074.3341, 1074.3341, 1105.3558, 1151.5179, 1153.3944, 1165.5214, 1179.3997, 1185.3542, 1198.3474, 1203.3622, 1209.3401, 1237.3750, 1253.3934, 1253.3934, 1270.4220, 1270.4220, 1276.3785, 1311.3831, 1315.3890, 1324.4233, 1324.4233, 1331.3602, 1334.6378, 1336.3842, 1340.3962, 1350.4242, 1359.4357, 1361.4412, 1384.3983, 1428.4093, 1450.4459, 1452.4446, 1468.3738, 1508.4353, 1508.4353, 1524.4346, 1552.3715, 1575.5162, 1589.4358, 1589.4358, 1601.4287, 1615.4470, 1617.7043, 1640.4285, 1640.4285, 1672.4479, 1753.4670, 1791.4602, 1799.5475, 1863.5592, 1873.5771, 1873.5771, 1889.5647, 2002.6814, 2220.7424, 2238.7305, 2245.8064, 2704.8149

**G20	Cs3g26510.1	Mass: 17810	Score: 105	Expect: 1.4e-006	Matches: 11**

Observed

Mr(expt)

Mr(calc)

ppm

Start

End

Miss

Ions

Peptide

744.2561

743.2488

743.3813

-178.27

127

-

132

0

---

K.TPEEIR.K

750.3632

749.3559

749.4436

-116.91

133

-

138

1

---

R.KTFNIK.N

754.1598

753.1525

753.3731

-292.79

56

-

61

0

---

K.VIEYCK.K

872.3123

871.3050

871.4763

-196.58

127

-

133

1

---

K.TPEEIRK.T

882.4025

881.3952

881.4680

-82.65

56

-

62

1

---

K.VIEYCKK.H

1020.3679

1019.3606

1019.4771

-114.28

73

-

82

0

---

R.ATSGVDDDLK.A

1020.3679

1019.3606

1019.4771

-114.28

73

-

82

0

---

R.ATSGVDDDLK.A

1493.4015

1492.3942

1492.6318

-159.14

139

-

150

0

---

K.NDFTPEEEEEVR.R

1649.4606

1648.4533

1648.7329

-169.55

139

-

151

1

---

K.NDFTPEEEEEVRR.E

1649.4606

1648.4533

1648.7329

-169.55

139

-

151

1

60

K.NDFTPEEEEEVRR.E

2224.6570

2223.6497

2224.0383

-174.71

11

-

31

0

---

K.SSDGEAFEVDEAVALESQTIK.H

No match to: 700.2577, 705.2280, 708.2020, 713.2798, 716.2351, 720.2312, 724.2986, 727.2654, 730.2343, 734.3627, 736.3405, 738.2438, 743.2705, 747.2406, 768.3770, 774.2431, 782.3159, 784.2810, 834.3439, 842.3448, 845.3112, 850.3626, 850.3626, 860.9199, 864.3716, 868.3774, 876.8948, 904.3289, 907.2959, 908.3089, 913.3179, 914.3041, 937.2958, 941.2899, 941.2899, 944.3052, 948.3198, 956.2873, 958.2897, 968.3400, 974.3444, 982.3984, 993.2627, 996.3659, 997.3550, 1005.2515, 1017.4992, 1024.3171, 1033.4987, 1042.3330, 1051.5167, 1059.3932, 1059.3932, 1060.3790, 1071.3252, 1081.2885, 1095.3162, 1103.3525, 1113.3031, 1115.3820, 1118.4050, 1126.3618, 1131.5040, 1151.4977, 1151.4977, 1165.5331, 1165.5331, 1179.3961, 1179.3961, 1249.4462, 1265.4634, 1273.4127, 1300.6392, 1308.4414, 1331.3988, 1334.6361, 1383.4951, 1448.6831, 1457.4673, 1479.4784, 1497.4572, 1507.4252, 1515.5664, 1617.8011, 1632.4442, 1663.4972, 2002.7117, 2143.7505, 2143.7505, 2220.7170, 2238.7542, 2238.7542, 2245.8030, 2261.7224, 2383.5513, 2540.7170, 2914.0173, 3311.7131, 3346.0637

**G29	Cs1g17350.1	Mass: 82844	Score: 311	Expect: 3.5e-027	Matches: 12**

Observed

Mr(expt)

Mr(calc)

ppm

Start

End

Miss

Ions

Peptide

720.3068

719.2995

719.4330

-185.52

643

-

648

0

---

R.ISVFVR.E

737.2567

736.2494

736.3425

-126.51

169

-

175

0

---

K.GICETSK.D

865.1893

864.1821

864.4011

-253.43

768

-

776

0

---

R.GIQGSVSSM.-

1106.3456

1105.3383

1105.5074

-152.91

649

-

658

0

---

R.EPVSSDICTR.A

1260.4215

1259.4142

1259.6146

-159.08

349

-

360

0

---

R.EFPADAILGDGR.I

1260.4215

1259.4142

1259.6146

-159.08

349

-

360

0

89

R.EFPADAILGDGR.I

1395.5387

1394.5314

1394.7558

-160.87

468

-

479

0

---

K.LSQYPTATIVFR.G

1395.5387

1394.5314

1394.7558

-160.87

468

-

479

0

107

K.LSQYPTATIVFR.G

1447.4426

1446.4353

1446.6739

-164.94

153

-

165

0

---

R.HSFSDSTLSDVPR.R

1447.4426

1446.4353

1446.6739

-164.94

153

-

165

0

104

R.HSFSDSTLSDVPR.R

2064.5178

2063.5105

2063.8629

-170.76

195

-

211

0

---

R.GYESYMERPIDETDESK.S + Oxidation (M)

2305.6880

2304.6807

2305.0723

-169.89

593

-

614

0

---

K.DLASGEESTPFIHGAGHVDPNR.A

No match to: 700.2690, 713.2818, 727.2613, 734.3506, 750.3817, 758.2020, 768.3876, 807.2546, 828.2529, 834.3441, 836.2941, 842.3605, 848.3371, 850.3718, 860.9262, 868.3879, 882.3996, 899.4080, 905.3029, 957.3249, 973.3546, 982.3702, 993.3378, 999.3498, 1017.4884, 1033.3453, 1037.3569, 1051.5153, 1059.3959, 1107.3875, 1113.3163, 1118.3313, 1125.3661, 1132.3575, 1151.5177, 1165.4060, 1165.4060, 1179.4061, 1179.4061, 1184.3975, 1193.4279, 1201.4103, 1234.4656, 1234.4656, 1242.4065, 1263.4381, 1265.4489, 1268.4542, 1270.4435, 1277.4866, 1308.4446, 1316.4120, 1320.3722, 1320.3722, 1329.4287, 1332.3065, 1334.5927, 1336.4215, 1340.4637, 1345.4630, 1357.4972, 1362.4620, 1365.4182, 1365.4182, 1371.4269, 1373.4429, 1377.3854, 1381.4244, 1383.4543, 1390.4547, 1393.5334, 1401.4274, 1417.4799, 1432.4348, 1434.5316, 1450.4318, 1475.5198, 1479.5034, 1480.4883, 1491.5007, 1493.4952, 1493.4952, 1507.4930, 1606.6161, 1681.5905, 1685.5698, 1699.5614, 1707.4994, 1707.4994, 1716.5914, 1740.4696, 1920.5120, 1993.6576, 2047.7236, 2080.5322, 2368.6584, 2383.5554, 2716.6160

**G24	Cs1g08770.1	Mass: 46457	Score: 425	Expect: 1.4e-038	Matches: 27**

Observed

Mr(expt)

Mr(calc)

ppm

Start

End

Miss

Ions

Peptide

732.2768

731.2695

731.4581

-257.88

386

-

391

0

---

R.YVILPK.D

791.2533

790.2460

790.3973

-191.44

233

-

239

0

---

K.YLGEGPR.M

816.3411

815.3339

815.4865

-187.16

109

-

115

0

---

R.ILSTINR.E

820.2728

819.2655

819.4273

-197.41

301

-

307

0

---

K.VIMATNR.A + Oxidation (M)

895.3347

894.3274

894.4923

-184.38

187

-

194

0

---

K.QIGIDPPR.G

980.2754

979.2682

979.4359

-171.28

269

-

277

0

---

R.FDAQTGADR.E

986.3486

985.3414

985.5233

-184.59

325

-

332

0

---

K.IEFPLPDR.R

992.3536

991.3464

991.5339

-189.10

224

-

232

0

---

R.VVGSEFVQK.Y

1114.4313

1113.4240

1113.6182

-174.39

324

-

332

1

---

R.KIEFPLPDR.R

1114.4313

1113.4240

1113.6182

-174.39

324

-

332

1

65

R.KIEFPLPDR.R

1142.4243

1141.4170

1141.6244

-181.63

325

-

333

1

---

K.IEFPLPDRR.Q

1158.4462

1157.4389

1157.6445

-177.56

195

-

206

0

---

R.GVLLYGPPGTGK.T

1271.4845

1270.4772

1270.6782

-158.17

212

-

223

0

---

K.AVANHTTAAFIR.V

1289.3839

1288.3766

1288.5976

-171.46

403

-

412

0

---

K.KPDTDFEFYK.-

1289.3839

1288.3766

1288.5976

-171.46

403

-

412

0

58

K.KPDTDFEFYK.-

1394.5387

1393.5314

1393.7677

-169.56

308

-

320

0

---

R.ADTLDPALLRPGR.L

1394.5387

1393.5314

1393.7677

-169.56

308

-

320

0

61

R.ADTLDPALLRPGR.L

1420.5872

1419.5799

1419.8198

-168.92

116

-

128

0

---

R.ELLKPSASVALHR.H

1678.5837

1677.5764

1677.8726

-176.51

173

-

186

0

---

R.EAVELPLTHHELYK.Q

1956.5480

1955.5407

1955.9557

-212.15

364

-

382

0

---

K.ISAAEIAAICQEAGMHAVR.K + Oxidation (M)

2028.7196

2027.7123

2028.0527

-167.85

250

-

268

0

---

K.ENAPAIIFIDEVDAIATAR.F

2028.7196

2027.7123

2028.0527

-167.85

250

-

268

0

87

K.ENAPAIIFIDEVDAIATAR.F

2140.6001

2139.5928

2139.9630

-172.99

346

-

363

0

---

K.MNLSDEVDLEDYVSRPDK.I + Oxidation (M)

2140.6001

2139.5928

2139.9630

-172.99

346

-

363

0

47

K.MNLSDEVDLEDYVSRPDK.I + Oxidation (M)

2153.6697

2152.6624

2153.0528

-181.31

44

-

60

1

---

R.QLEFIDIQEEYVKDEQK.N

2239.7158

2238.7085

2239.0865

-168.79

282

-

300

0

---

R.ILMELLNQMDGFDQTVNVK.V + 2 Oxidation (M)

2245.8149

2244.8076

2244.2015

270

212

-

232

1

---

K.AVANHTTAAFIRVVGSEFVQK.Y

No match to: 713.2753, 716.2305, 730.2531, 734.3483, 768.3815, 809.2080, 834.3279, 842.3502, 849.2746, 850.3465, 860.9135, 868.3826, 876.8757, 878.3077, 882.3953, 965.2894, 974.3907, 1004.3964, 1011.2872, 1017.4487, 1032.3768, 1041.3547, 1051.5077, 1056.4227, 1059.3920, 1066.3708, 1099.3416, 1125.4418, 1132.3735, 1151.4976, 1165.5028, 1172.4683, 1172.4683, 1179.3890, 1243.5244, 1252.4908, 1256.4677, 1263.4362, 1263.4362, 1270.5112, 1270.5112, 1275.4169, 1286.4879, 1292.4514, 1315.4152, 1330.4448, 1351.4413, 1376.5146, 1377.5048, 1384.4557, 1399.4895, 1402.5767, 1416.5354, 1418.5300, 1434.5510, 1462.5016, 1566.4443, 1625.6085, 1633.5487, 1696.5490, 1827.5933, 1858.6461, 1922.6531, 1938.6506, 1962.4960, 1972.5763, 1974.4886, 1974.4886, 1988.5085, 1990.4901, 2043.7079, 2076.6323, 2107.6978, 2107.6978, 2117.5735, 2121.6709, 2123.7031, 2128.6523, 2136.6702, 2156.6099, 2175.6372, 2238.7390, 3346.0557

**G6	Cs8g03250.1	Mass: 44641	Score: 463	Expect: 2.2e-042	Matches: 23**

Observed

Mr(expt)

Mr(calc)

ppm

Start

End

Miss

Ions

Peptide

718.3230

717.3157

717.4537

-192.36

208

-

213

0

---

R.GIFLLR.K

757.3426

756.3353

756.4858

-198.90

201

-

207

0

---

K.VTGLVLR.G

846.3351

845.3279

845.5487

-261.16

208

-

214

1

---

R.GIFLLRK.K

870.3183

869.3110

869.4606

-172.09

70

-

76

0

---

K.ELPELNR.N

904.3486

903.3413

903.5178

-195.35

137

-

143

0

---

R.IILFDQR.G

904.3486

903.3413

903.5178

-195.35

137

-

143

0

58

R.IILFDQR.G

985.3233

984.3160

984.4876

-174.27

238

-

245

0

---

R.DLIPENER.S

985.3233

984.3160

984.4876

-174.27

238

-

245

0

48

R.DLIPENER.S

1106.2977

1105.2904

1105.4869

-177.74

129

-

136

0

---

R.FFDPDFYR.I

1106.2977

1105.2904

1105.4869

-177.74

129

-

136

0

35

R.FFDPDFYR.I

1108.3582

1107.3509

1107.6149

-238.31

329

-

338

0

---

R.HINATIVQGR.Y

1262.3761

1261.3688

1261.5880

-173.74

128

-

136

1

---

R.RFFDPDFYR.I

1262.3761

1261.3688

1261.5880

-173.74

128

-

136

1

35

R.RFFDPDFYR.I

1340.4196

1339.4123

1339.6408

-170.57

291

-

302

0

---

R.GEDDIFSLAFAR.I

1340.4196

1339.4123

1339.6408

-170.57

291

-

302

0

93

R.GEDDIFSLAFAR.I

1346.4429

1345.4356

1345.6990

-195.73

16

-

27

0

---

R.TFSLPHTSTLSR.L

1496.4948

1495.4875

1495.7419

-170.09

290

-

302

1

---

K.RGEDDIFSLAFAR.I

1574.5974

1573.5901

1573.8001

-133.44

30

-

43

0

---

R.FLPSSHQNFTLAGR.K

1581.4824

1580.4751

1580.7430

-169.48

256

-

269

1

---

R.LNSDDKETQYAAAR.A

1581.4824

1580.4751

1580.7430

-169.48

256

-

269

1

116

R.LNSDDKETQYAAAR.A

1756.5912

1755.5839

1755.9083

-184.74

77

-

91

0

---

R.NLYPYVEPYSTGILK.V

1969.6332

1968.6259

1968.9581

-168.73

312

-

328

0

---

K.GFFPSDSFLLDNIDNIR.H

2332.7515

2331.7442

2332.0926

-149.39

57

-

76

1

---

K.SEPELIDMMESGKELPELNR.N + Oxidation (M)

No match to: 700.2594, 713.2754, 724.3579, 733.2913, 734.3168, 735.3193, 749.2839, 750.3202, 768.3824, 817.2625, 840.2794, 842.3476, 850.3854, 858.3550, 860.9192, 864.3256, 868.3866, 882.3877, 889.3261, 920.3516, 926.3361, 942.3195, 956.2999, 966.2922, 978.3674, 979.2972, 996.3660, 998.2906, 1007.3217, 1010.2905, 1010.2905, 1015.3788, 1026.2997, 1051.5095, 1059.3761, 1117.4189, 1121.3593, 1128.3309, 1144.2930, 1145.3439, 1151.4888, 1157.3496, 1160.3745, 1165.4971, 1179.3492, 1195.3951, 1211.3748, 1211.3748, 1219.4584, 1223.3757, 1233.3596, 1239.4926, 1239.4926, 1244.3959, 1249.3776, 1265.3872, 1269.3938, 1315.4319, 1331.3917, 1334.5422, 1348.4073, 1357.5363, 1357.5363, 1362.4133, 1374.4896, 1378.3866, 1384.3884, 1427.5093, 1457.5101, 1470.4498, 1476.5085, 1480.5947, 1487.4631, 1504.4797, 1544.6134, 1562.4963, 1648.4520, 1778.5916, 1794.5692, 1831.5487, 1894.6530, 1986.7224, 2238.7378, 2245.8093, 2354.7563, 2371.7017, 3775.1292

**G25	Cs9g05680.1	Mass: 47830	Score: 181	Expect: 3.5e-014	Matches: 16**

Observed

Mr(expt)

Mr(calc)

ppm

Start

End

Miss

Ions

Peptide

752.2050

751.1977

751.3501

-202.71

387

-

392

0

---

K.GYLEDR.R

899.3892

898.3819

898.5600

-198.19

329

-

336

0

---

K.AILNLSLR.H

899.3892

898.3819

898.5600

-198.19

329

-

336

0

60

K.AILNLSLR.H

907.2441

906.2368

906.4043

-184.77

56

-

63

0

---

K.SEDSTVNR.L

966.3277

965.3204

965.4818

-167.13

170

-

178

0

---

R.AAEIFSNSK.V

1361.3733

1360.3660

1360.6007

-172.50

339

-

350

0

---

K.EHISAYGEGNER.R

1361.3733

1360.3660

1360.6007

-172.50

339

-

350

0

82

K.EHISAYGEGNER.R

1561.5717

1560.5644

1560.8511

-183.69

99

-

112

0

---

K.TISKPVEHPSELPK.W

1626.4847

1625.4774

1625.7546

-170.49

337

-

350

1

---

R.HKEHISAYGEGNER.R

1762.6321

1761.6248

1761.9036

-158.24

64

-

78

0

---

R.LEDLLNLDITPYTDK.I

1762.6321

1761.6248

1761.9036

-158.24

64

-

78

0

---

R.LEDLLNLDITPYTDK.I

1776.5269

1775.5196

1775.8227

-170.67

356

-

371

0

---

K.HETASIDSFSWGVANR.G

1823.6029

1822.5956

1822.9287

-182.74

79

-

94

0

---

K.IIAEYIWIGGTGIDMR.S + Oxidation (M)

1963.5327

1962.5254

1962.9478

-215.16

2

-

17

1

---

M.AQILAPSMQWQMRMPK.Y + 3 Oxidation (M)

2577.8518

2576.8445

2577.3115

-181.18

179

-

200

0

---

K.VSAEVPWFGIEQEYTLLQQNVK.W

2650.7935

2649.7862

2650.2973

-192.86

56

-

78

1

---

K.SEDSTVNRLEDLLNLDITPYTDK.I

No match to: 712.1075, 713.1240, 714.1192, 715.1585, 734.3098, 842.3577, 850.3393, 859.2280, 921.3613, 989.2675, 1066.2677, 1088.3335, 1095.3090, 1343.3505, 1373.3970, 1470.4695, 1642.5602, 1758.6071, 1761.6849, 1764.6082, 1765.0138, 1770.5391, 1772.6074, 1774.6074, 1780.5195, 1780.5195, 1788.5615, 1790.4961, 1792.5137, 1792.5137, 1796.6296, 1808.5082, 1808.5082, 1812.5751, 1822.5798, 1826.6055, 1827.5958, 1827.5958, 1836.6243, 1839.5983, 1839.5983, 1843.6051, 1855.5950, 1855.5950, 1861.5481, 1864.5331, 1871.5693, 1875.5623, 1877.5836, 1879.5969, 1895.5879, 1902.5487, 1911.5625, 1920.5730, 1923.5780, 1930.5782, 1933.5995, 1935.6055, 1936.6106, 1937.5863, 1948.5780, 1951.5725, 1953.5470, 1961.5720, 1971.6320, 1983.6842, 1987.6565, 1989.6521, 1991.6309, 2052.7002, 2230.7070, 2444.7346, 2508.8625, 2528.8313, 2580.7522, 2582.8140, 2585.8733, 2593.8110, 2595.8184, 2598.7871, 2599.8374, 2599.8374, 2603.8516, 2604.7876, 2613.7778, 2615.7637, 2619.8142, 2633.8230, 2674.8503, 2679.6462, 2681.7793, 2987.7954, 2993.8381, 3010.7869

**G34	orange1.1t02649.1	Mass: 45976	Score: 189	Expect: 5.6e-015	Matches: 16**

Observed

Mr(expt)

Mr(calc)

ppm

Start

End

Miss

Ions

Peptide

734.2947

733.2875

733.3680

-109.81

350

-

355

0

---

R.LCEELK.D

798.3206

797.3133

797.4759

-203.96

97

-

103

0

---

K.VINVTPR.Y

1051.4894

1050.4821

1050.6410

-151.24

22

-

30

1

---

R.RPGLRLPSR.I

1129.3849

1128.3776

1128.4943

-103.43

298

-

306

1

---

K.MMKSEFNDK.D

1131.4147

1130.4074

1130.5502

-126.30

386

-

394

0

---

R.MQQEAIQQR.K

1147.3615

1146.3542

1146.5451

-166.51

386

-

394

0

---

R.MQQEAIQQR.K + Oxidation (M)

1147.3615

1146.3542

1146.5451

-166.51

386

-

394

0

12

R.MQQEAIQQR.K + Oxidation (M)

1151.4628

1150.4555

1150.5223

-58.05

194

-

204

1

---

R.KMGAAVGQDCR.L + Oxidation (M)

1398.3748

1397.3675

1397.6034

-168.77

360

-

370

0

---

K.MHNFSSIEDFR.G + Oxidation (M)

1398.3748

1397.3675

1397.6034

-168.77

360

-

370

0

49

K.MHNFSSIEDFR.G + Oxidation (M)

1439.4868

1438.4795

1438.7377

-179.46

151

-

162

0

---

R.ILIASIMEEYNK.A + Oxidation (M)

1474.4774

1473.4701

1473.7133

-165.03

225

-

237

0

---

K.MTPNITDITEPAR.V + Oxidation (M)

1723.5829

1722.5756

1722.8577

-163.72

371

-

385

0

---

R.GASIEYFTTHTELVR.M

1723.5829

1722.5756

1722.8577

-163.72

371

-

385

0

96

R.GASIEYFTTHTELVR.M

2108.5913

2107.5840

2108.0493

-220.73

242

-

262

0

---

R.SGSEGVSAINTIMSVMGIDLK.T

2124.6062

2123.5989

2124.0442

-209.66

242

-

262

0

---

R.SGSEGVSAINTIMSVMGIDLK.T + Oxidation (M)

No match to: 700.2815, 711.2873, 713.2816, 716.2334, 724.3191, 730.2415, 736.3731, 746.2815, 750.3379, 760.2396, 768.3801, 842.3585, 850.3828, 853.2745, 856.3207, 868.3859, 882.4046, 887.3354, 899.3664, 914.2502, 940.4214, 945.3402, 960.3082, 996.3842, 999.4285, 999.4285, 1017.4714, 1059.3857, 1079.3455, 1096.3292, 1099.3470, 1106.3596, 1106.3596, 1118.3613, 1118.3613, 1134.3492, 1139.4419, 1155.3833, 1155.3833, 1163.3578, 1165.4009, 1165.5298, 1172.4508, 1174.4280, 1228.4001, 1251.3923, 1253.4125, 1263.4219, 1265.4105, 1286.5103, 1302.5140, 1315.4308, 1332.4457, 1333.4376, 1334.6439, 1360.4689, 1361.4269, 1376.4785, 1402.4846, 1414.4006, 1418.5537, 1429.4519, 1430.4735, 1435.4456, 1448.6111, 1487.4807, 1490.4814, 1498.5177, 1536.5139, 1553.5287, 1568.5438, 1576.5251, 1591.5791, 1626.5193, 1656.5306, 1666.5383, 1666.5383, 1697.5079, 1737.5535, 1773.5742, 1778.7369, 1780.6399, 1793.7205, 1793.7205, 1810.5747, 1813.5786, 1823.5610, 1858.5126, 1933.6571, 2028.6168, 2028.6168, 2238.7600, 2245.8208, 2518.6729

**G27	Cs5g07120.1	Mass: 54347	Score: 371	Expect: 3.5e-033	Matches: 26**

Observed

Mr(expt)

Mr(calc)

ppm

Start

End

Miss

Ions

Peptide

762.2759

761.2687

761.4032

-176.67

440

-

447

0

---

K.GSVSVTGR.T

777.2411

776.2339

776.3851

-194.74

194

-

200

0

---

K.AMVDVAR.E + Oxidation (M)

779.2550

778.2477

778.3973

-192.19

172

-

178

0

---

R.AGAIYER.K

849.2694

848.2621

848.4578

-230.71

478

-

484

0

---

R.LYGLPMR.V

865.2922

864.2850

864.4527

-194.09

478

-

484

0

---

R.LYGLPMR.V + Oxidation (M)

865.2922

864.2850

864.4527

-194.09

478

-

484

0

---

R.LYGLPMR.V + Oxidation (M)

892.2526

891.2453

891.4120

-187.03

350

-

356

0

---

R.IDMVENR.L + Oxidation (M)

907.2712

906.2639

906.4923

-251.93

172

-

179

1

---

R.AGAIYERK.Y

907.2712

906.2639

906.5022

-262.91

428

-

436

0

2

K.TTTGSVTLK.L

938.2759

937.2687

937.4392

-181.96

247

-

254

0

---

R.EDAIEYAK.K

1007.3585

1006.3512

1006.5335

-181.09

156

-

163

1

---

K.DLKEEFVK.D

1041.3525

1040.3452

1040.5291

-176.68

401

-

409

0

---

K.YAELVYAGR.W

1041.3525

1040.3452

1040.5291

-176.68

401

-

409

0

75

K.YAELVYAGR.W

1104.3478

1103.3405

1103.6274

-259.89

478

-

486

1

---

R.LYGLPMRVR.A

1131.4012

1130.3939

1130.5819

-166.25

136

-

145

1

---

K.ELDGLEEKAK.A

1413.4241

1412.4168

1412.6493

-164.58

416

-

427

0

---

R.ESIDAFMENITK.T + Oxidation (M)

1639.6278

1638.6205

1638.8940

-166.89

329

-

344

0

---

K.LSPASLLAELNEIGGR.H

1639.6278

1638.6205

1638.8940

-166.89

329

-

344

0

102

K.LSPASLLAELNEIGGR.H

1767.7074

1766.7001

1766.9890

-163.48

328

-

344

1

---

K.KLSPASLLAELNEIGGR.H

2028.6002

2027.5929

2028.0098

-205.58

2

-

21

0

---

M.AQSTAVSPCSSINLAFHAPK.R

2399.7166

2398.7093

2399.3577

-270.21

95

-

116

1

---

R.LNKVVLAYSGGLDTSVIVPWLR.E

2400.6821

2399.6748

2399.3577

132

95

-

116

1

---

R.LNKVVLAYSGGLDTSVIVPWLR.E

2400.6821

2399.6748

2399.3577

132

95

-

116

1

11

R.LNKVVLAYSGGLDTSVIVPWLR.E

2417.7034

2416.6961

2417.1135

-172.69

456

-

477

0

---

R.QDISSFESGQIYDQADAAGFIR.L

2417.7034

2416.6961

2417.1135

-172.69

456

-

477

0

112

R.QDISSFESGQIYDQADAAGFIR.L

2694.9131

2693.9058

2694.3752

-174.22

364

-

388

0

---

R.GVYETPGGTILFSAVQELESLTLDR.E

No match to: 700.2548, 703.2288, 708.2720, 713.2814, 716.2288, 732.2547, 734.3425, 744.2643, 752.2618, 768.3840, 801.3013, 818.2658, 836.3095, 837.2699, 842.3527, 847.2752, 848.2852, 851.2840, 868.3364, 875.2654, 881.2946, 882.3552, 889.2632, 891.3022, 899.3038, 901.2492, 913.2598, 918.2619, 919.2631, 919.2631, 933.2932, 935.2690, 946.2776, 948.3072, 959.2774, 961.2698, 962.3052, 963.3279, 973.2728, 982.3583, 987.2949, 992.3511, 992.3511, 999.4151, 1003.3149, 1027.3434, 1051.5009, 1054.2944, 1059.3824, 1063.3351, 1075.3558, 1080.4183, 1083.3499, 1099.3181, 1099.3181, 1102.3610, 1115.3289, 1132.3622, 1151.4949, 1165.5062, 1262.4447, 1267.4021, 1280.4109, 1450.4967, 1460.5198, 1494.5571, 1510.5183, 1547.5223, 1599.6051, 1629.6028, 1629.6028, 1693.5656, 1778.7449, 1933.6443, 2002.6924, 2060.7510, 2221.6997, 2238.7471, 2245.7588, 2387.6863, 2405.7483, 2412.7983, 2424.6675, 3346.0544

**G28	Cs5g35290.1	Mass: 55622	Score: 124	Expect: 1.8e-008	Matches: 15**

Observed

Mr(expt)

Mr(calc)

ppm

Start

End

Miss

Ions

Peptide

729.2753

728.2680

728.4181

-206.04

146

-

151

0

---

R.NVDILR.D

848.3234

847.3161

847.4116

-112.70

161

-

166

0

---

K.YFYDIK.D

972.3143

971.3071

971.4712

-168.99

333

-

340

0

---

K.ASFLSEYR.F

972.3143

971.3071

971.4712

-168.99

333

-

340

0

27

K.ASFLSEYR.F

1059.3755

1058.3682

1058.5080

-132.03

474

-

482

1

---

R.NFPGRMGHK.E + Oxidation (M)

1059.3755

1058.3682

1058.5080

-132.03

474

-

482

1

---

R.NFPGRMGHK.E + Oxidation (M)

1200.4482

1199.4409

1199.6914

-208.80

402

-

412

0

---

K.VPTFLVPATQK.V

1255.4106

1254.4033

1254.5914

-149.95

273

-

283

0

---

K.SMEFVGTTVER.L

1271.3843

1270.3770

1270.5864

-164.76

273

-

283

0

---

K.SMEFVGTTVER.L + Oxidation (M)

1562.5540

1561.5467

1561.7776

-147.85

333

-

345

1

---

K.ASFLSEYRFDISK.L

1804.6445

1803.6372

1803.9513

-174.08

58

-

74

1

---

R.RPATTGSVKNAMTLTEK.I

1817.6509

1816.6436

1816.9359

-160.89

128

-

142

0

---

K.IVIIPDHYIFTSDER.A

1817.6509

1816.6436

1816.9359

-160.89

128

-

142

0

72

K.IVIIPDHYIFTSDER.A

1903.6709

1902.6636

1903.0666

-211.75

255

-

272

0

---

K.DLILQIIGEISVAGATYK.S

2614.8440

2613.8367

2614.2915

-173.95

483

-

507

0

---

K.EGQIYLASPYTAAASALTGYVTDPR.E

No match to: 700.2366, 706.2413, 712.2117, 715.2012, 716.2691, 717.1974, 734.3604, 736.3145, 738.2540, 744.2758, 750.3605, 752.2631, 757.2064, 768.3420, 769.3027, 771.2178, 779.1965, 795.2817, 807.2783, 820.2463, 834.3319, 842.3569, 850.3608, 850.3608, 859.2609, 860.9213, 860.9213, 868.3891, 870.3799, 876.8954, 876.8954, 892.8635, 900.2742, 902.2623, 904.2543, 910.2908, 913.2837, 931.2993, 939.3157, 953.3544, 958.2753, 969.3615, 982.3481, 996.3933, 1000.3646, 1008.3207, 1012.3239, 1017.4783, 1019.4296, 1025.3212, 1035.3888, 1037.3865, 1040.3490, 1042.3363, 1045.3718, 1051.5214, 1073.4351, 1090.3676, 1103.3331, 1103.3331, 1107.3722, 1119.3409, 1128.4340, 1131.4520, 1140.3998, 1151.5181, 1165.5073, 1176.4470, 1244.3774, 1263.4437, 1279.4766, 1312.4795, 1315.4354, 1331.3940, 1334.6279, 1379.4933, 1437.3503, 1437.3503, 1447.4869, 1448.5576, 1486.5535, 1519.4930, 1571.4404, 1573.5735, 1582.5337, 1617.7378, 1662.4557, 1662.4557, 1839.6365, 2140.7180, 2218.8206, 2218.8206, 2238.7493, 2308.7400, 3969.1658

**G22	orange1.1t04488.3	Mass: 26964	Score: 318	Expect: 7e-028	Matches: 16**

Observed

Mr(expt)

Mr(calc)

ppm

Start

End

Miss

Ions

Peptide

780.2469

779.2396

779.3966

-201.45

213

-

218

0

---

K.FHVSYK.G

856.3496

855.3423

855.5178

-205.09

77

-

84

0

---

K.NLGLNVVK.A

1037.3075

1036.3002

1036.4825

-175.89

240

-

248

0

---

R.RPTTEEASF.-

1037.3075

1036.3002

1036.4825

-175.89

240

-

248

0

50

R.RPTTEEASF.-

1254.4280

1253.4207

1253.5573

-108.95

1

-

10

0

---

-.MYISSCTFFR.F

1254.4280

1253.4207

1253.5573

-108.95

1

-

10

0

---

-.MYISSCTFFR.F

1275.4570

1274.4497

1274.6904

-188.81

65

-

76

0

---

R.LGALLDTMNALK.N + Oxidation (M)

1383.5110

1382.5037

1382.7405

-171.25

108

-

119

1

---

R.KVDDPEVLEAIR.L

1383.5110

1382.5037

1382.7405

-171.25

108

-

119

1

75

R.KVDDPEVLEAIR.L

1663.7372

1662.7299

1663.0144

-171.08

221

-

235

0

---

K.AIIKPLQQVLANSLR.Y

1663.7372

1662.7299

1663.0144

-171.08

221

-

235

0

89

K.AIIKPLQQVLANSLR.Y

2050.8574

2049.8501

2050.2038

-172.50

170

-

188

0

---

R.SLLLVETADRPGLLVDLVK.I

2050.8574

2049.8501

2050.2038

-172.50

170

-

188

0

54

R.SLLLVETADRPGLLVDLVK.I

2226.6628

2225.6555

2226.0553

-179.60

150

-

169

0

---

K.QQVDVDIATHISVFDDGPDR.S

2303.7642

2302.7569

2303.1533

-172.10

44

-

64

0

---

K.VIIDLDSDPDATIVEITFGDR.L

2376.7700

2375.7627

2376.2060

-186.56

189

-

210

0

---

K.IITDINVNVESGEFDTEGLLAK.A

No match to: 700.2200, 705.2159, 708.2005, 713.2681, 716.2149, 724.2786, 727.2380, 730.1984, 732.2271, 734.3342, 736.3325, 747.2466, 748.2296, 750.3503, 752.2765, 764.2342, 768.3751, 770.2541, 799.2215, 807.3038, 816.2631, 830.2815, 838.2994, 842.3466, 850.3710, 852.2493, 864.3120, 868.3710, 882.3956, 917.3578, 922.2468, 926.3633, 948.2483, 1051.3330, 1051.5106, 1059.3610, 1081.3759, 1116.3788, 1127.3488, 1139.3995, 1141.4534, 1151.5028, 1165.4977, 1187.4165, 1189.4459, 1202.4177, 1205.4417, 1205.4417, 1209.4280, 1221.4391, 1221.4391, 1227.4210, 1236.4210, 1239.3936, 1243.4017, 1277.4243, 1315.4132, 1359.4210, 1366.3116, 1397.5044, 1404.3409, 1517.6156, 1627.5497, 1645.6237, 1750.5391, 1750.5391, 1764.5588, 1786.6056, 1787.6318, 1787.6318, 1801.6450, 1802.6443, 2002.7070, 2072.8289, 2111.5928, 2120.7280, 2124.6831, 2124.6831, 2129.6272, 2138.6978, 2142.7729, 2146.6594, 2150.5764, 2162.5928, 2184.5947, 2198.5515, 2201.5889, 2209.6584, 2214.5298, 2220.6702, 2223.5969, 2238.7139, 2245.8074, 2289.7485

**G32	Cs2g12010.1	Mass: 23324	Score: 113	Expect: 2.2e-007	Matches: 12**

Observed

Mr(expt)

Mr(calc)

ppm

Start

End

Miss

Ions

Peptide

700.2557

699.2485

699.3050

-80.88

1

-

5

0

---

-.MGYWK.S + Oxidation (M)

734.3553

733.3480

733.3428

7.06

24

-

31

0

---

K.AAAAEACK.S

768.3820

767.3747

767.3966

-28.57

2

-

7

1

---

M.GYWKSK.V

777.2626

776.2553

776.4068

-195.20

95

-

101

0

---

K.IEFPGSK.A

1152.4331

1151.4258

1151.6074

-157.68

126

-

135

0

---

K.VSTFIVTEEK.A

1294.4991

1293.4918

1293.6088

-90.43

38

-

47

1

---

K.EEINKEFEEK.K

1437.5037

1436.4964

1436.7398

-169.42

55

-

67

0

---

K.VVEIYEASSAEIK.T

1437.5037

1436.4964

1436.7398

-169.42

55

-

67

0

37

K.VVEIYEASSAEIK.T

1712.5975

1711.5902

1711.8974

-179.44

110

-

125

0

---

K.FGAASVPGPVFFIFEK.V

1973.5970

1972.5897

1973.0105

-213.27

185

-

205

0

---

K.TEPAEAAAAPAPTEPLPAAAK.E

1973.5970

1972.5897

1973.0105

-213.27

185

-

205

0

36

K.TEPAEAAAAPAPTEPLPAAAK.E

2962.8618

2961.8545

2962.3891

-180.44

136

-

165

0

---

K.AAEPATAAAGEETKPEQETTSSSTAAVTEK.D

No match to: 701.2270, 713.2816, 716.2367, 718.2376, 724.3214, 730.2325, 736.3554, 750.3839, 783.2701, 806.2847, 834.3463, 842.3496, 850.3773, 860.9152, 864.3626, 868.3696, 876.8807, 882.3879, 933.3317, 952.3168, 952.3168, 974.3699, 996.3758, 1017.4791, 1051.5063, 1059.3828, 1126.4071, 1131.4167, 1132.3329, 1132.3329, 1145.5031, 1147.4176, 1151.5089, 1154.3293, 1165.5153, 1176.3510, 1188.4386, 1217.3995, 1222.4232, 1241.5181, 1257.4589, 1279.4712, 1293.4575, 1296.4667, 1300.4934, 1315.4196, 1334.4681, 1353.4669, 1353.4669, 1365.4896, 1374.4412, 1375.3855, 1375.3855, 1381.4772, 1391.3950, 1397.3711, 1416.4174, 1432.4207, 1433.4106, 1459.4417, 1481.5319, 1487.5219, 1547.4844, 1547.4844, 1550.4895, 1559.4993, 1566.5146, 1568.4938, 1569.4772, 1581.5059, 1586.4926, 1586.4926, 1591.4570, 1592.5304, 1608.4774, 1624.4592, 1635.6270, 1645.4563, 1673.5048, 1723.5881, 1725.5782, 1734.5857, 1812.6259, 1987.5709, 1987.5709, 1995.6288, 2002.6776, 2011.6116, 2022.6339, 2068.7910, 2097.6404, 2103.4797, 2220.7075, 2238.7339, 2238.7339, 2245.7852, 2306.5081, 2691.8694

**G35	orange1.1t03395.1	Mass: 53195	Score: 568	Expect: 7e-053	Matches: 25**

Observed

Mr(expt)

Mr(calc)

ppm

Start

End

Miss

Ions

Peptide

702.2564

701.2491

701.4072

-225.33

162

-

167

0

---

R.ILVSDR.A

781.2736

780.2663

780.4130

-188.01

189

-

195

0

---

K.SFIGTTR.R

836.3024

835.2951

835.4076

-134.63

451

-

457

0

---

R.NYSDLPK.G

863.3027

862.2955

862.4549

-184.83

242

-

248

0

---

K.YTPDVLR.E

863.3027

862.2955

862.4549

-184.83

242

-

248

0

9

K.YTPDVLR.E

998.4471

997.4399

997.6172

-177.76

322

-

331

0

---

R.VVGDLIGVVK.A

1019.2991

1018.2918

1018.4542

-159.46

218

-

225

0

---

R.HMDTFPQK.L + Oxidation (M)

1035.3433

1034.3360

1034.5509

-207.67

196

-

205

1

---

R.RGIGPAYSSK.V

1045.3794

1044.3721

1044.5498

-170.13

209

-

217

1

---

R.NGIRVCDLR.H

1157.4541

1156.4468

1156.6452

-171.47

226

-

236

0

---

K.LDLLLSDAAAR.F

1157.4541

1156.4468

1156.6452

-171.47

226

-

236

0

52

K.LDLLLSDAAAR.F

1477.5038

1476.4965

1476.7460

-168.94

242

-

253

1

---

K.YTPDVLREEVEK.Y

1486.5724

1485.5651

1485.8443

-187.86

421

-

433

0

---

K.SFPADLLLLEQLK.V

1490.5149

1489.5076

1489.7637

-171.92

356

-

369

0

---

R.LAGQEFGTTTGRPR.R

1490.5149

1489.5076

1489.7637

-171.92

356

-

369

0

75

R.LAGQEFGTTTGRPR.R

1610.6191

1609.6118

1609.8828

-168.31

83

-

96

0

---

K.LVDILAEHFDIVAR.C

1610.6191

1609.6118

1609.8828

-168.31

83

-

96

0

93

K.LVDILAEHFDIVAR.C

1720.5389

1719.5316

1719.8217

-168.66

337

-

353

0

---

R.VGSGPFPTEIFGQGGDR.L

1720.5389

1719.5316

1719.8217

-168.66

337

-

353

0

92

R.VGSGPFPTEIFGQGGDR.L

1810.6172

1809.6099

1809.9162

-169.23

168

-

182

0

---

R.AHLLFDFHQEIDGLR.E

1810.6172

1809.6099

1809.9162

-169.23

168

-

182

0

82

R.AHLLFDFHQEIDGLR.E

1882.7107

1881.7034

1882.0200

-168.22

466

-

483

0

---

R.IEELVGVPIDYIGVGPGR.D

1882.7107

1881.7034

1882.0200

-168.22

466

-

483

0

101

R.IEELVGVPIDYIGVGPGR.D

2220.7585

2219.7512

2219.0932

297

261

-

279

0

---

R.LEPFITDTVHFMNESIAQK.R

2235.6843

2234.6770

2235.0882

-183.95

261

-

279

0

---

R.LEPFITDTVHFMNESIAQK.R + Oxidation (M)

No match to: 700.2631, 705.2255, 710.1918, 713.2795, 716.2302, 724.2930, 729.2039, 730.2438, 734.3404, 742.2374, 752.2572, 756.2220, 758.2451, 768.3767, 771.2484, 783.2930, 793.2260, 806.2579, 819.2515, 834.2850, 842.3508, 850.3423, 860.9156, 868.3721, 874.2703, 876.8835, 882.3981, 902.2629, 907.2933, 911.2828, 960.2743, 962.3129, 962.3129, 973.2865, 979.3026, 982.3618, 996.3945, 999.4271, 1017.4240, 1020.3575, 1024.3042, 1032.3541, 1041.3469, 1051.5105, 1053.3617, 1059.3892, 1080.4193, 1126.3817, 1141.4081, 1150.3790, 1151.4537, 1160.3817, 1162.3774, 1165.5415, 1170.4401, 1210.3617, 1237.4105, 1237.4105, 1251.3971, 1265.4791, 1270.5276, 1315.4243, 1337.4156, 1346.4583, 1365.4763, 1384.4539, 1453.5088, 1472.4879, 1489.4799, 1501.5148, 1625.5936, 1629.6027, 1650.5303, 1728.4794, 1734.5477, 1746.4471, 1749.4971, 1809.5977, 1967.6384, 1979.6388, 1979.6388, 1995.6331, 2002.6641, 2238.7324, 2245.7795

**G37	Cs1g01370.1	Mass: 19611	Score: 213	Expect: 2.2e-017	Matches: 7**

Observed

Mr(expt)

Mr(calc)

ppm

Start

End

Miss

Ions

Peptide

834.3549

833.3476

833.4317

-100.89

172

-

179

0

---

K.SKPLASTM.-

850.3870

849.3797

849.4266

-55.15

172

-

179

0

---

K.SKPLASTM.- + Oxidation (M)

978.3368

977.3295

977.4930

-167.29

82

-

90

0

---

R.GNLDIFSGR.G

978.3368

977.3295

977.4930

-167.29

82

-

90

0

66

R.GNLDIFSGR.G

1964.6740

1963.6667

1963.9851

-162.12

42

-

61

0

---

K.GGTDSIIGVTLYDAVGDGVR.I

1964.6740

1963.6667

1963.9851

-162.12

42

-

61

0

134

K.GGTDSIIGVTLYDAVGDGVR.I

2290.7114

2289.7041

2289.0524

285

62

-

81

0

---

R.ISNLEAWGGLMGPGYNYFER.G + Oxidation (M)

No match to: 700.2775, 708.2272, 712.1321, 713.2885, 716.2540, 724.3710, 726.2598, 728.2593, 730.2529, 734.3665, 736.3604, 744.2751, 750.3964, 762.2719, 768.3920, 782.3456, 791.2546, 797.2468, 814.2911, 821.2802, 842.3650, 848.3586, 854.9100, 860.9133, 864.3837, 868.3951, 876.8925, 882.4039, 913.3148, 960.3301, 982.3804, 992.3567, 994.3010, 996.4034, 1000.3199, 1017.5009, 1045.3159, 1051.5358, 1059.3988, 1074.4093, 1077.4495, 1077.4495, 1102.3258, 1151.5277, 1165.4961, 1179.4142, 1234.4977, 1259.5012, 1259.5012, 1263.4714, 1315.4562, 1320.3981, 1334.6426, 1412.4567, 1419.4052, 1434.6149, 1476.4244, 1492.4244, 1526.6477, 1527.0806, 1528.1458, 1529.4445, 1530.0367, 1551.4520, 1568.4786, 1579.4130, 1618.4115, 1620.4353, 1620.4353, 1634.6215, 1636.3656, 1636.3656, 1652.4458, 1707.5226, 1809.6254, 1946.6868, 1963.6655, 1978.6866, 1986.6531, 2002.6100, 2241.7197, 2241.7197, 2257.6978, 2287.7034, 2293.6877, 2302.6809, 2304.6577, 2305.6824, 2305.6824, 2310.6831, 2319.6487, 2321.6858, 2321.6858, 2335.6814, 2337.6724, 2337.6724, 2341.6682, 2376.6641, 2430.8606, 2444.8735, 2452.8813, 2466.8577, 2687.9626

**G38	Cs1g01370.1	Mass: 19611	Score: 222	Expect: 2.8e-018	Matches: 7**

Observed

Mr(expt)

Mr(calc)

ppm

Start

End

Miss

Ions

Peptide

834.3577

833.3505

833.4317

-97.45

172

-

179

0

---

K.SKPLASTM.-

850.3892

849.3819

849.4266

-52.63

172

-

179

0

---

K.SKPLASTM.- + Oxidation (M)

978.3324

977.3251

977.4930

-171.78

82

-

90

0

---

R.GNLDIFSGR.G

978.3324

977.3251

977.4930

-171.78

82

-

90

0

72

R.GNLDIFSGR.G

1964.6687

1963.6614

1963.9851

-164.82

42

-

61

0

---

K.GGTDSIIGVTLYDAVGDGVR.I

1964.6687

1963.6614

1963.9851

-164.82

42

-

61

0

137

K.GGTDSIIGVTLYDAVGDGVR.I

2289.6804

2288.6731

2289.0524

-165.70

62

-

81

0

---

R.ISNLEAWGGLMGPGYNYFER.G + Oxidation (M)

No match to: 700.2718, 703.2318, 707.3468, 713.2867, 724.3550, 727.3124, 730.2510, 734.3613, 736.3669, 742.2683, 744.2461, 750.3837, 757.2962, 768.3925, 814.2994, 842.3572, 848.3516, 854.8954, 864.4165, 868.3886, 876.9016, 882.4005, 891.2390, 914.2407, 960.3401, 968.4088, 982.3561, 996.3989, 1007.5012, 1017.5056, 1051.5219, 1059.4012, 1065.8990, 1074.4003, 1077.4353, 1101.2808, 1102.3235, 1131.5358, 1133.4868, 1151.5229, 1165.4960, 1179.4106, 1259.4913, 1320.3864, 1334.6442, 1476.3894, 1492.4004, 1493.4596, 1528.1898, 1529.4319, 1529.4319, 1530.0052, 1578.3701, 1580.4031, 1618.4265, 1620.4194, 1620.4194, 1634.6100, 1636.3732, 1636.3732, 1707.5189, 1809.6190, 1838.6454, 1920.6758, 1946.6694, 1986.6587, 2229.7061, 2231.6763, 2241.7148, 2241.7148, 2257.7319, 2275.6926, 2279.6672, 2287.6848, 2291.6934, 2293.6880, 2293.6880, 2303.6797, 2305.6736, 2305.6736, 2309.6794, 2317.7217, 2320.6775, 2321.6665, 2321.6665, 2326.6787, 2330.6138, 2334.6226, 2336.6880, 2337.6697, 2337.6697, 2340.6565, 2344.6458, 2351.6621, 2353.6335, 2365.6504, 2377.6636, 2393.6167, 2431.8186, 2473.7583, 2667.8323, 2677.8274, 2681.8198

**G14	Cs6g18900.1	Mass: 27496	Score: 371 Expect: 3.5e-033	Matches: 13**

Observed

Mr(expt)

Mr(calc)

ppm

Start

End

Miss

Ions

Peptide

763.2590

762.2517

762.4024

-197.70

94

-

100

0

---

R.GSPAYLR.L

937.3303

936.3230

936.4916

-180.09

119

-

126

0

---

K.LYTGDLQK.R

1034.3522

1033.3449

1033.5305

-179.53

92

-

100

1

---

R.DRGSPAYLR.L

1094.3973

1093.3900

1093.5848

-178.12

204

-

211

0

---

K.LFYTFYLK.G

1094.3973

1093.3900

1093.5848

-178.12

204

-

211

0

55

K.LFYTFYLK.G

1119.4502

1118.4429

1118.6488

-184.06

195

-

203

0

---

K.LQQLVFPFK.L

1119.4502

1118.4429

1118.6488

-184.06

195

-

203

0

60

K.LQQLVFPFK.L

1528.4985

1527.4912

1527.7681

-181.25

80

-

91

0

---

K.VQELHVYEINER.D

1528.4985

1527.4912

1527.7681

-181.25

80

-

91

0

83

K.VQELHVYEINER.D

1942.6997

1941.6924

1942.0524

-185.34

101

-

118

0

---

R.LSQKPVNSLGDLVPFSNK.L

1942.6997

1941.6924

1942.0524

-185.34

101

-

118

0

74

R.LSQKPVNSLGDLVPFSNK.L

2461.8726

2460.8653

2461.3217

-185.41

215

-

238

0

---

K.DLPEVLLGKPVDPHPGVEPSPAAK.A

2461.8726

2460.8653

2461.3217

-185.41

215

-

238

0

68

K.DLPEVLLGKPVDPHPGVEPSPAAK.A

No match to: 700.2628, 705.2597, 713.2766, 716.2376, 718.2423, 720.2256, 724.3626, 725.2352, 726.2354, 730.2502, 734.3474, 736.3655, 744.2527, 748.2528, 750.3654, 768.3791, 770.3360, 782.3351, 802.2609, 834.3477, 836.3392, 838.3519, 842.3492, 848.3427, 850.3746, 856.3502, 860.9169, 864.3607, 868.3837, 882.3990, 932.3789, 945.3411, 966.3185, 982.3723, 996.4004, 1017.4865, 1021.3496, 1045.3735, 1051.5013, 1059.3781, 1099.4008, 1113.3575, 1116.3562, 1126.3583, 1131.4415, 1141.4307, 1151.5026, 1157.4047, 1163.4210, 1165.4877, 1170.3401, 1186.4159, 1237.5009, 1265.4552, 1288.3174, 1297.4121, 1297.4121, 1306.4100, 1315.4169, 1334.6274, 1404.5084, 1428.4124, 1434.4532, 1434.4532, 1448.4966, 1456.4421, 1465.5044, 1486.5007, 1497.5028, 1497.5028, 1510.4774, 1511.5007, 1533.5049, 1542.5240, 1542.5240, 1550.4784, 1566.4579, 1585.5143, 1613.5548, 1634.3872, 1964.6714, 1980.6718, 2002.6685, 2159.6887, 2220.7102, 2238.7346, 2238.7346, 2245.7817, 2249.7168, 2266.6057, 2475.8816, 2526.6050, 2541.6699, 2557.6648, 2567.7363, 2648.5498, 2666.7283

**G41	Cs1g10270.1	Mass: 68638	Score: 635	Expect: 1.4e-059	Matches: 40**

Observed

Mr(expt)

Mr(calc)

ppm

Start

End

Miss

Ions

Peptide

749.2579

748.2506

748.3868

-181.97

2

-

8

0

---

M.PSVYGAR.L

833.2548

832.2475

832.3926

-174.38

617

-

623

0

---

R.ALEDETR.-

871.3398

870.3325

870.4923

-183.58

215

-

222

0

---

R.TPRPVSSK.L

883.3080

882.3008

882.4599

-180.38

578

-

584

0

---

R.LGDLFYR.L

883.3080

882.3008

882.4599

-180.38

578

-

584

0

36

R.LGDLFYR.L

885.2886

884.2813

884.4392

-178.53

385

-

391

0

---

R.LASFYER.A

906.3033

905.2960

905.4607

-181.87

116

-

123

0

---

R.SGDVYIPR.G

1002.2817

1001.2744

1001.4454

-170.73

18

-

25

0

---

K.ESEYGYVR.K

1158.3984

1157.3911

1157.5829

-165.68

607

-

616

0

---

K.LHEDLTAGFR.A

1163.4397

1162.4324

1162.5659

-114.79

133

-

141

0

---

K.DTLWEFQPK.K

1255.4866

1254.4793

1254.6932

-170.45

223

-

234

0

---

K.LAADTPLLTGQR.V

1255.4866

1254.4793

1254.6932

-170.45

223

-

234

0

59

K.LAADTPLLTGQR.V

1286.4689

1285.4616

1285.6779

-168.20

606

-

616

1

---

K.KLHEDLTAGFR.A

1335.5178

1334.5105

1334.6805

-127.37

204

-

214

0

---

K.SFTMLQAWPVR.T

1335.5178

1334.5105

1334.7306

-164.91

48

-

59

0

80

R.VGHDNLIGEIIR.L

1396.5220

1395.5147

1395.7398

-161.26

178

-

190

0

---

K.VTYVAPAGQYSLK.D

1471.5029

1470.4956

1470.7566

-177.43

489

-

501

0

---

R.EDDLNEIVQLVGK.D

1505.5457

1504.5384

1504.8137

-182.93

191

-

203

1

---

K.DTVLELEFQGVKK.S

1561.5531

1560.5458

1560.7930

-158.36

312

-

326

0

---

R.TTLVANTSNMPVAAR.E + Oxidation (M)

1595.6094

1594.6021

1594.8389

-148.44

561

-

575

1

---

K.GAGMDGQKITYTLIK.H

1705.4843

1704.4770

1704.8988

-247.37

446

-

459

1

---

R.KHFPSVNWLISYSK.Y

1733.5931

1732.5858

1732.8672

-162.36

327

-

341

0

---

R.EASIYTGITIAEYFR.D

1733.5931

1732.5858

1732.8672

-162.36

327

-

341

0

77

R.EASIYTGITIAEYFR.D

1747.5951

1746.5878

1746.8828

-168.87

590

-

605

1

---

K.FEDPAEGEPALVAKFK.K

1773.6277

1772.6204

1772.9209

-169.50

546

-

560

0

---

R.NIIHFYNLANQAVEK.G

1773.6277

1772.6204

1772.9209

-169.50

546

-

560

0

100

R.NIIHFYNLANQAVEK.G

1795.6050

1794.5977

1794.8610

-146.70

368

-

384

0

---

R.LAEMPADSGYPAYLAAR.L

1811.5592

1810.5519

1810.8559

-167.89

368

-

384

0

---

R.LAEMPADSGYPAYLAAR.L + Oxidation (M)

1811.5592

1810.5519

1810.8559

-167.89

368

-

384

0

57

R.LAEMPADSGYPAYLAAR.L + Oxidation (M)

2056.6973

2055.6900

2056.0265

-163.66

517

-

533

1

---

K.LLREDYLAQNAFTPYDK.F

2094.6111

2093.6038

2093.9429

-161.94

9

-

25

1

---

R.LTTFEDEEKESEYGYVR.K

2094.6111

2093.6038

2093.9429

-161.94

9

-

25

1

49

R.LTTFEDEEKESEYGYVR.K

2096.7041

2095.6968

2096.1113

-197.75

484

-

501

1

---

R.EVLQREDDLNEIVQLVGK.D

2108.6304

2107.6231

2107.1749

213

215

-

234

1

---

R.TPRPVSSKLAADTPLLTGQR.V

2180.6936

2179.6863

2180.0606

-171.67

27

-

47

0

---

K.VSGPVVIADGMNGAAMYELVR.V + 2 Oxidation (M)

2308.7629

2307.7556

2308.1555

-173.26

26

-

47

1

---

R.KVSGPVVIADGMNGAAMYELVR.V + 2 Oxidation (M)

2454.7346

2453.7273

2454.1379

-167.32

460

-

479

0

---

K.YSTALESFYEQFDPDFINIR.T

2512.6897

2511.6824

2512.1284

-177.53

283

-

304

0

---

R.GNEMAEVLMDFPQLTMTLPDGR.E + 3 Oxidation (M)

2735.8716

2734.8643

2735.3324

-171.11

60

-

84

0

---

R.LEGDSATIQVYEETAGLMVNDPVLR.T + Oxidation (M)

2799.1223

2798.1150

2798.5807

-166.39

85

-

110

0

---

R.THKPLSVELGPGILGNIFDGIQRPLK.T

No match to: 713.2837, 748.2730, 761.2521, 768.3864, 787.1963, 820.2923, 842.3474, 850.3742, 857.2091, 868.3793, 873.2145, 882.3998, 961.3730, 969.3325, 996.4005, 1051.5170, 1059.3823, 1061.3335, 1165.3921, 1179.4063, 1198.4027, 1234.4656, 1280.4257, 1320.3851, 1334.5774, 1355.4552, 1367.4441, 1367.4441, 1383.4453, 1395.5308, 1419.4830, 1434.5370, 1465.5171, 1493.4969, 1495.5089, 1524.4884, 1531.5641, 1532.5946, 1560.5643, 1562.5861, 1564.5616, 1564.5616, 1566.5461, 1593.5367, 1606.5748, 1614.4919, 1617.5117, 1623.5458, 1699.5593, 1707.4946, 1721.4578, 1757.6091, 1793.5983, 1827.5626, 1838.6077, 1884.7168, 1920.7349, 1920.7349, 1968.6620, 1982.6779, 1993.6448, 2045.7139, 2075.6689, 2091.6790, 2097.7551, 2116.6948, 2162.8052, 2196.6873, 2222.6851, 2883.0366

**G42	Cs4g06170.1	Mass: 26791	Score: 228	Expect: 7e-019	Matches: 11**

Observed

Mr(expt)

Mr(calc)

ppm

Start

End

Miss

Ions

Peptide

758.2650

757.2577

757.4082

-198.70

128

-

135

0

---

R.SGPAASLR.V

824.3503

823.3431

823.4473

-126.62

182

-

188

1

---

K.TSIKTMK.A + Oxidation (M)

1033.4520

1032.4447

1032.4811

-35.21

40

-

48

0

---

R.CALSPSNWR.E

1251.3903

1250.3830

1250.5891

-164.79

140

-

152

0

---

R.AVAQYASDAGNGK.M

1251.3903

1250.3830

1250.5891

-164.79

140

-

152

0

53

R.AVAQYASDAGNGK.M

1252.3929

1251.3856

1251.5224

-109.26

153

-

163

0

---

K.MASDDVDQCLR.A

1291.4611

1290.4538

1290.6819

-176.75

164

-

174

0

---

R.ALEDLDSLFLR.A

1291.4611

1290.4538

1290.6819

-176.75

164

-

174

0

82

R.ALEDLDSLFLR.A

1719.4491

1718.4418

1718.7483

-178.29

222

-

237

0

---

R.SADDDTEAQVSDPEIK.Q

1719.4491

1718.4418

1718.7483

-178.29

222

-

237

0

67

R.SADDDTEAQVSDPEIK.Q

1733.4784

1732.4711

1732.8856

-239.19

136

-

152

1

---

R.VNIRAVAQYASDAGNGK.M

No match to: 700.2706, 705.2614, 709.2115, 713.2799, 716.2378, 721.2127, 724.3599, 727.2545, 730.2469, 734.3505, 736.3616, 737.2169, 738.2374, 743.2367, 744.2590, 750.3749, 765.2429, 768.3791, 834.3457, 842.3496, 848.3632, 850.3796, 860.9082, 864.3562, 868.3840, 876.8757, 880.3066, 882.3940, 889.3081, 905.2825, 916.3026, 917.2768, 926.3369, 951.4175, 968.3938, 982.3984, 996.4191, 1005.3741, 1007.4392, 1017.4896, 1019.5023, 1042.3809, 1045.3866, 1051.5189, 1059.3893, 1059.3893, 1093.4169, 1099.3833, 1115.3956, 1126.3525, 1131.5115, 1151.5105, 1151.5105, 1155.3619, 1165.5367, 1165.5367, 1186.3507, 1188.3597, 1188.3597, 1189.5022, 1231.3344, 1231.3344, 1237.5242, 1265.4895, 1273.4136, 1290.4280, 1295.4088, 1300.6262, 1305.4816, 1313.4377, 1315.4246, 1334.6510, 1351.5090, 1424.4679, 1428.4025, 1434.6000, 1448.6515, 1477.5336, 1486.5092, 1487.5526, 1489.4799, 1489.4799, 1490.0690, 1538.4419, 1578.4877, 1580.4750, 1588.5370, 1596.4094, 1596.4094, 1612.4518, 1617.7383, 1695.5510, 1731.7450, 1741.4381, 1874.5247, 1995.6013, 2002.6918, 2238.7292, 2245.7869

**G43	Cs8g19010.1	Mass: 55648	Score: 285	Expect: 1.4e-024	Matches: 20**

Observed

Mr(expt)

Mr(calc)

ppm

Start

End

Miss

Ions

Peptide

774.2946

773.2873

773.4283

-182.27

463

-

470

0

---

K.AGTLPSTK.A

786.3234

785.3162

785.4759

-203.42

452

-

458

0

---

K.VQIATVR.G

814.3585

813.3512

813.4596

-133.18

488

-

494

1

---

K.EELPKAK.A

1060.3629

1059.3556

1059.5349

-169.21

326

-

335

0

---

K.VFTDPSAPAR.R

1158.4205

1157.4132

1157.6404

-196.26

459

-

470

1

---

R.GQAKAGTLPSTK.A

1199.4514

1198.4441

1198.6306

-155.55

497

-

506

0

---

K.QINESKPEVR.K

1209.4315

1208.4242

1208.6401

-178.62

434

-

444

0

---

K.FSSQITTAVQK.S

1381.5088

1380.5015

1380.7361

-169.89

129

-

142

0

---

R.AGVPELGAAQELAR.L

1381.5088

1380.5015

1380.7361

-169.89

129

-

142

0

108

R.AGVPELGAAQELAR.L

1398.4550

1397.4477

1397.7626

-225.30

495

-

506

1

---

K.AKQINESKPEVR.K

1421.4543

1420.4470

1420.6834

-166.39

337

-

349

0

---

R.VDELFSAIAEDGR.R

1555.5004

1554.4931

1554.8730

-244.28

445

-

458

1

---

K.STDQIPKVQIATVR.G

1577.5200

1576.5127

1576.7845

-172.37

337

-

350

1

---

R.VDELFSAIAEDGRR.A

1592.5319

1591.5246

1591.7955

-170.15

99

-

115

0

---

K.DSGTVFVAGATGQAGVR.I

1592.5319

1591.5246

1591.7955

-170.15

99

-

115

0

118

K.DSGTVFVAGATGQAGVR.I

1694.5293

1693.5220

1693.8159

-173.47

158

-

173

0

---

R.LNAVESNFDSAESIAK.A

1720.6112

1719.6039

1719.8904

-166.58

98

-

115

1

---

R.KDSGTVFVAGATGQAGVR.I

2189.7573

2188.7500

2189.1328

-174.87

75

-

96

0

---

R.DVTSLIPVVSSPGTGLSFGNSR.R

2329.5996

2328.5923

2328.9326

-146.13

46

-

67

1

---

K.SKDENSSDESSNGDSGNSNPFR.F

2330.5400

2329.5327

2328.9326

258

46

-

67

1

---

K.SKDENSSDESSNGDSGNSNPFR.F

No match to: 700.2555, 713.2782, 716.2363, 730.2609, 734.3560, 736.3361, 744.2600, 750.3782, 752.2747, 768.3785, 781.2808, 831.2681, 842.3545, 850.3771, 868.3795, 882.3973, 914.2417, 928.2817, 967.2828, 986.3009, 1017.3343, 1017.3343, 1021.3505, 1051.5109, 1059.3777, 1069.3135, 1082.3943, 1090.4180, 1107.3088, 1157.4178, 1157.4178, 1165.5122, 1182.4119, 1187.4497, 1191.4789, 1196.3944, 1249.4088, 1261.4856, 1287.4618, 1287.4618, 1302.4972, 1303.4664, 1305.4419, 1305.4419, 1315.4174, 1320.4033, 1320.4033, 1322.4564, 1334.6267, 1352.4399, 1355.5165, 1363.4562, 1395.4950, 1429.4441, 1438.4800, 1447.4974, 1448.5314, 1465.5076, 1465.5076, 1479.5093, 1502.4841, 1526.5081, 1546.4648, 1547.4839, 1564.4956, 1564.4956, 1569.4648, 1614.5105, 1675.4775, 1679.5665, 1714.6071, 1715.6057, 1731.6333, 1733.5908, 1791.5322, 1799.6232, 1823.6246, 1835.5603, 1851.5662, 1954.6659, 1967.6478, 2047.6956, 2069.6812, 2122.8132, 2186.7151, 2186.7151, 2202.7195, 2238.7312, 2245.7971, 2271.7839

**G44	Cs6g06590.1	Mass: 35985	Score: 485	Expect: 1.4e-044	Matches: 14**

Observed

Mr(expt)

Mr(calc)

ppm

Start

End

Miss

Ions

Peptide

824.2385

823.2313

823.3824

-183.57

106

-

112

0

---

R.SEQYAAR.G

1017.4020

1016.3947

1016.5615

-164.04

83

-

93

0

---

K.STVLVTGAGGR.T

1051.3959

1050.3886

1050.5379

-142.13

67

-

76

1

---

R.SCVSKTEAVK.V

1109.3550

1108.3477

1108.5261

-160.91

104

-

112

1

---

K.ERSEQYAAR.G

1219.3914

1218.3841

1218.5728

-154.83

270

-

279

0

---

K.DDELLQTETR.T

1361.4703

1360.4630

1360.6987

-173.19

124

-

136

0

---

K.IGGADDLFIGDIR.D

1361.4703

1360.4630

1360.6987

-173.19

124

-

136

0

136

K.IGGADDLFIGDIR.D

1619.5634

1618.5561

1618.8203

-163.16

303

-

318

0

---

K.AFDLASKPEGTGTPTK.D

1809.6392

1808.6319

1808.9308

-165.24

236

-

251

0

---

K.AEQYLADSGIPYTIIR.A

1809.6392

1808.6319

1808.9308

-165.24

236

-

251

0

105

K.AEQYLADSGIPYTIIR.A

1858.6658

1857.6585

1857.9684

-166.76

264

-

279

1

---

R.ELLVGKDDELLQTETR.T

1858.6658

1857.6585

1857.9684

-166.76

264

-

279

1

96

R.ELLVGKDDELLQTETR.T

1937.7090

1936.7017

1937.0258

-167.30

235

-

251

1

---

R.KAEQYLADSGIPYTIIR.A

1937.7090

1936.7017

1937.0258

-167.30

235

-

251

1

116

R.KAEQYLADSGIPYTIIR.A

No match to: 705.2506, 713.2800, 715.2759, 716.2498, 734.3343, 750.3840, 768.3779, 800.2768, 842.3538, 844.2980, 848.3271, 851.3404, 864.3274, 868.3834, 870.3200, 882.4000, 886.2862, 914.2660, 917.3580, 927.3296, 956.3554, 960.3884, 966.3532, 979.3872, 1005.3981, 1005.3981, 1019.4063, 1042.3258, 1052.3241, 1052.3241, 1059.3815, 1098.4004, 1133.4769, 1133.4769, 1147.4121, 1149.4037, 1151.4552, 1160.4373, 1164.4012, 1164.4012, 1186.3995, 1211.3823, 1214.3562, 1261.4946, 1274.4698, 1279.4564, 1286.4657, 1291.4294, 1293.4353, 1309.4210, 1309.4210, 1315.4105, 1317.4377, 1318.4867, 1331.3854, 1334.5841, 1339.4515, 1343.4744, 1353.4507, 1357.4340, 1366.4646, 1383.4459, 1411.6094, 1467.5901, 1476.4935, 1511.5767, 1537.6177, 1564.5033, 1591.5315, 1639.6115, 1747.5966, 1818.6031, 1829.5876, 1831.5879, 1840.6456, 1872.6748, 1897.6022, 1915.6278, 1920.6619, 1959.6592, 2008.4995, 2024.5394, 2033.6543, 2044.7134, 2089.6458, 2164.5774, 2173.6853, 2220.7817, 2238.7473, 2245.8098, 2699.7974, 2705.7900, 2717.7891, 2717.7891, 2733.7847, 2810.7888

**Unidentified protein spots**

**G45	Cs3g17220.3	Mass: 108105	Score: 52	Expect: 0.31	Matches: 28**

Observed

Mr(expt)

Mr(calc)

ppm

Start

End

Miss

Ions

Peptide

702.2223

701.2150

701.3609

-208.01

883

-

887

0

---

R.WNQVR.A

704.2481

703.2408

703.3289

-125.32

594

-

598

0

---

K.QHYEK.K

705.2408

704.2335

704.3089

-107.05

2

-

7

0

---

M.ENSEAR.D

708.2352

707.2280

707.2908

-88.88

807

-

811

0

---

R.QMEER.A + Oxidation (M)

750.3831

749.3758

749.3932

-23.21

358

-

363

1

---

K.YANRAR.N

836.3325

835.3252

835.3494

-28.89

1

-

7

0

---

-.MENSEAR.D

850.3738

849.3665

849.4457

-93.23

874

-

880

1

---

R.ERVFSGR.G

850.3738

849.3665

849.4457

-93.23

874

-

880

1

---

R.ERVFSGR.G

902.3151

901.3078

901.5167

-231.72

737

-

743

1

---

R.QKMVLQR.K

910.2906

909.2833

909.4378

-169.91

118

-

124

0

---

K.VMDNIFR.R + Oxidation (M)

918.3494

917.3421

917.5117

-184.81

737

-

743

1

---

R.QKMVLQR.K + Oxidation (M)

919.3270

918.3197

918.4341

-124.58

807

-

813

1

---

R.QMEERAR.M

935.3046

934.2973

934.4290

-140.98

807

-

813

1

---

R.QMEERAR.M + Oxidation (M)

982.3978

981.3905

981.5535

-166.08

139

-

146

0

---

R.VSFIEIFK.E

1017.4890

1016.4817

1016.5185

-36.19

812

-

820

1

---

R.ARMANEVAR.L

1017.4890

1016.4817

1016.5185

-36.19

812

-

820

1

---

R.ARMANEVAR.L

1031.3387

1030.3314

1030.5593

-221.13

814

-

822

1

---

R.MANEVARLK.E

1033.4608

1032.4535

1032.5134

-58.02

812

-

820

1

---

R.ARMANEVAR.L + Oxidation (M)

1033.4608

1032.4535

1032.5134

-58.02

812

-

820

1

---

R.ARMANEVAR.L + Oxidation (M)

1099.3413

1098.3340

1098.5604

-206.08

434

-

442

0

---

R.LTCQHLTQR.A

1122.3220

1121.3147

1121.4924

-158.39

722

-

729

1

---

R.RNEYEMHK.L + Oxidation (M)

1122.3220

1121.3147

1121.4924

-158.39

722

-

729

1

---

R.RNEYEMHK.L + Oxidation (M)

1131.4855

1130.4782

1130.5931

-101.64

325

-

335

0

---

R.LLQDSLGGNSK.T

1273.3953

1272.3880

1272.6860

-234.12

163

-

174

0

---

K.GESASVLKPVMR.V

1300.5894

1299.5821

1299.6129

-23.66

199

-

209

1

---

R.SKEEMASFLSR.G + Oxidation (M)

1315.4303

1314.4230

1314.7044

-214.03

883

-

893

1

---

R.WNQVRALTEAK.N

1334.6396

1333.6323

1333.6053

20.3

374

-

384

1

---

R.DPMAAQMQRMR.S

1334.6396

1333.6323

1333.6053

20.3

374

-

384

1

---

R.DPMAAQMQRMR.S

No match to: 700.2640, 706.2611, 709.2167, 713.2762, 716.2341, 718.2539, 724.3614, 726.2275, 730.2441, 734.3544, 736.3590, 744.2529, 758.2341, 768.3789, 796.2441, 818.2691, 834.3326, 842.3501, 848.3589, 856.3661, 856.3661, 860.9037, 864.3617, 864.3617, 868.3801, 882.3947, 905.2896, 948.3041, 962.3455, 968.3686, 976.3100, 978.3273, 989.3456, 996.4068, 1007.4813, 1014.3043, 1019.5012, 1021.4126, 1045.3746, 1049.3677, 1051.5148, 1054.4198, 1059.3729, 1059.3729, 1076.3605, 1088.3398, 1110.3302, 1117.5004, 1126.3481, 1128.3776, 1133.5026, 1138.3665, 1147.4856, 1151.5133, 1151.5133, 1155.4015, 1165.5258, 1165.5258, 1179.4110, 1251.4871, 1263.4270, 1265.4863, 1281.4337, 1293.4391, 1331.3862, 1374.4397, 1390.4376, 1397.3558, 1413.3542, 1428.4019, 1434.6257, 1448.6288, 1457.4349, 1617.7446, 1717.7310, 1731.7675, 1900.8499, 2002.7205, 2208.6272, 2224.6821, 2238.7317, 2245.8022

**G46	Cs3g23180.1	Mass: 51385	Score: 55	Expect: 0.14	Matches: 2**

Observed

Mr(expt)

Mr(calc)

ppm

Start

End

Miss

Ions

Peptide

1863.5121

1862.5048

1862.8786

-200.62

244

-

259

0

---

K.VVTIDDYEDVPLNDEK.A

1863.5121

1862.5048

1862.8786

-200.62

244

-

259

0

55

K.VVTIDDYEDVPLNDEK.A

No match to: 700.2565, 704.2446, 705.2422, 709.1955, 713.2699, 716.2240, 724.3415, 725.2165, 730.2406, 734.3389, 736.3312, 738.2195, 744.2455, 750.3586, 765.2398, 768.3731, 818.2397, 834.3342, 837.3390, 842.3415, 850.3697, 856.3628, 860.8986, 864.3411, 868.3726, 876.8754, 882.3773, 957.3049, 968.3575, 982.3522, 991.2958, 1002.3482, 1003.2994, 1017.4819, 1021.3439, 1037.2961, 1045.3635, 1049.3439, 1051.4834, 1051.4834, 1055.3625, 1059.3639, 1062.3286, 1062.3286, 1066.2926, 1074.3192, 1074.3192, 1090.3079, 1094.3729, 1108.3634, 1126.3452, 1131.3383, 1149.3822, 1150.4196, 1151.4686, 1163.3589, 1165.4597, 1172.4093, 1194.3672, 1198.3385, 1202.3722, 1208.3694, 1231.3251, 1253.3945, 1253.3945, 1263.4111, 1270.4146, 1270.4146, 1315.3949, 1331.4047, 1334.6177, 1353.4528, 1359.4070, 1375.3662, 1383.4650, 1400.4111, 1437.4063, 1448.4923, 1470.4545, 1482.4071, 1547.4852, 1550.4026, 1589.4210, 1589.4210, 1601.4166, 1601.4166, 1605.4076, 1616.4133, 1617.4254, 1619.4366, 1639.5012, 1841.4930, 1855.6060, 1885.5131, 1940.5616, 2002.6564, 2002.6564, 2220.7195, 2238.7146, 2238.7146, 2245.7778, 2260.7173, 2423.6926, 2435.7593, 2567.6990, 2694.8140, 2751.7861, 3346.0518

**G47	Cs8g03090.1	Mass: 17581	Score: 51	Expect: 0.34	Matches: 8**

Observed

Mr(expt)

Mr(calc)

ppm

Start

End

Miss

Ions

Peptide

1007.4671

1006.4598

1006.5270

-66.72

27

-

35

0

---

K.AQMGFNVLK.T

1019.4925

1018.4852

1018.5658

-79.15

69

-

78

0

---

R.LNSSASSLLK.L

1021.3826

1020.3753

1020.5352

-156.68

98

-

105

1

---

R.SLRFEQNK.R

1036.3762

1035.3689

1035.5171

-143.11

11

-

18

1

---

K.HMYLNKSK.A + Oxidation (M)

1060.4031

1059.3958

1059.5713

-165.59

132

-

140

0

---

R.AILSPEQFR.I

1060.4031

1059.3958

1059.5713

-165.59

132

-

140

0

24

R.AILSPEQFR.I

1151.5248

1150.5175

1150.6169

-86.33

26

-

35

1

---

R.KAQMGFNVLK.T + Oxidation (M)

2647.7854

2646.7781

2646.4017

142

45

-

68

0

---

R.TFSSILSNLNFIHSPPTTTLLTSR.L

No match to: 700.2780, 705.2593, 708.2370, 709.2510, 713.2873, 716.2519, 718.2835, 724.3668, 730.2644, 734.3596, 736.3704, 744.2662, 750.3829, 752.2457, 768.3913, 782.3137, 810.3052, 816.3093, 824.3510, 834.3582, 836.3536, 842.3541, 848.3543, 850.3867, 850.3867, 854.9030, 856.3602, 860.9221, 864.3477, 864.3477, 868.3933, 876.8903, 882.4088, 907.2958, 914.2717, 916.3246, 951.4023, 968.3953, 982.4063, 986.4075, 996.4221, 1010.3499, 1014.3141, 1017.4860, 1033.4705, 1034.3386, 1045.3828, 1051.5277, 1053.3416, 1054.3328, 1059.3915, 1117.4279, 1126.3750, 1131.4592, 1133.5143, 1142.3530, 1154.4097, 1165.5438, 1165.5438, 1169.3887, 1205.3639, 1216.3885, 1315.4299, 1330.4116, 1334.6558, 1334.6558, 1343.4622, 1373.5175, 1398.4208, 1398.4208, 1414.4258, 1418.4807, 1434.6106, 1435.5413, 1435.5413, 1448.6570, 1455.5425, 1455.5425, 1460.4476, 1464.4613, 1484.4598, 1493.4716, 1592.5284, 1617.7871, 1620.6172, 1869.5641, 2002.7302, 2002.7302, 2181.8064, 2220.7493, 2224.7090, 2238.7605, 2238.7605, 2245.8242, 2423.7180, 2434.7871, 2635.7808, 2666.8169, 2694.8220, 2751.8223, 2810.8223, 3346.0830

**G48	Cs7g03150.1	Mass: 15554	Score: 55	Expect: 0.13	Matches: 7**

Observed

Mr(expt)

Mr(calc)

ppm

Start

End

Miss

Ions

Peptide

966.3702

965.3629

965.4454

-85.44

77

-

84

0

---

K.YGNLEENK.Y

1016.3113

1015.3040

1015.4611

-154.66

131

-

140

0

---

K.SGGGYGDYLK.T

1179.4290

1178.4217

1178.6295

-176.30

2

-

11

1

---

M.DSLFSKLQNK.E

1257.4778

1256.4705

1256.6724

-160.68

63

-

76

0

---

K.VAGAAENLVAAGSK.Y

1471.4821

1470.4748

1470.6990

-152.45

77

-

88

1

---

K.YGNLEENKYVDK.A

1471.4821

1470.4748

1470.6990

-152.45

77

-

88

1

29

K.YGNLEENKYVDK.A

1665.5100

1664.5027

1664.7576

-153.12

27

-

41

0

---

K.EHHQPSNAELMASAK.C + Oxidation (M)

No match to: 700.2888, 707.2370, 709.2520, 711.2604, 713.2908, 716.2637, 719.2225, 721.1816, 723.1858, 724.3825, 725.3257, 727.2878, 730.2575, 734.3659, 736.3805, 744.2617, 750.3944, 768.3969, 782.3380, 834.3664, 836.3755, 838.3805, 842.3428, 848.3798, 850.3947, 850.3947, 854.9094, 854.9094, 860.9272, 860.9272, 864.3825, 868.4053, 876.8998, 882.4172, 901.2526, 951.4724, 968.4064, 982.4246, 982.4246, 996.4357, 996.4357, 999.3975, 1007.5199, 1017.5069, 1019.5194, 1033.5262, 1037.3650, 1049.9434, 1051.5426, 1053.2556, 1059.4048, 1059.8918, 1065.9076, 1065.9076, 1081.8796, 1117.4707, 1131.5247, 1133.5345, 1140.3733, 1147.5315, 1151.5411, 1165.5527, 1165.5527, 1227.5282, 1229.5756, 1234.4773, 1241.4454, 1249.4559, 1251.5326, 1265.5397, 1276.9045, 1279.5568, 1290.6295, 1300.6451, 1311.4447, 1315.4459, 1320.3947, 1331.4200, 1334.6748, 1334.6748, 1340.4716, 1347.4871, 1365.4397, 1434.6643, 1448.6849, 1453.4919, 1493.4741, 1548.6226, 1617.8047, 1707.5182, 1717.7736, 1731.8044, 1867.6093, 2383.5940, 2678.8342, 2704.8303, 2763.8857, 2797.7864, 2831.8394, 2943.8096, 2943.8096, 2959.7810, 3142.8938
